# Chemoselective Hydrogenation of α,β-Unsaturated Ketones Catalyzed by a Manganese(I) Hydride Complex

**DOI:** 10.1021/acs.orglett.4c00277

**Published:** 2024-05-13

**Authors:** Kartick Dey, Graham de Ruiter

**Affiliations:** ‡ Schulich Faculty of Chemistry, Technion − Israel Institute of Technology, Technion City, 3200008 Haifa, Israel

## Abstract

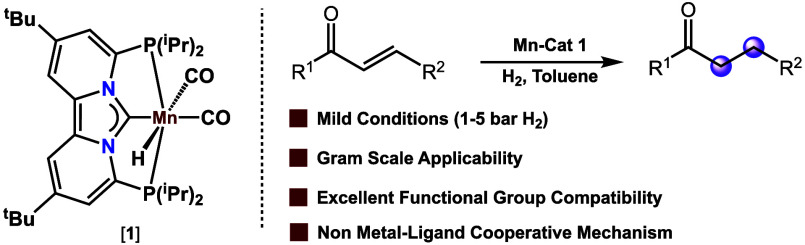

Here, we report the chemoselective hydrogenation of α,β-unsaturated ketones catalyzed by a well-defined Mn(I) PC_NHC_P pincer complex [(PC_NHC_P)Mn(CO)_2_H] (**1**). The reaction is compatible with a wide variety of functional groups that include halides, esters, amides, nitriles, nitro, alkynes, and alkenes, and for most substrates occurs readily at ambient hydrogen pressure (1–2 bar). Mechanistic studies and deuterium labeling experiments reveal a non-cooperative mechanism, which is further discussed in this report.

With growing emphasis on developing sustainable methodologies in chemical synthesis, continuing efforts have been devoted to developing atom economical reactions that utilize earth-abundant rather precious metals.^1^ As a result, in the past decade, iron^2^ and cobalt^3^ have generally been used as hydrogenation catalysts in a variety of transformations. Manganese, on the other hand, has less frequently been utilized as a hydrogenation catalyst despite being the third most abundant transition metal. Only in 2016, the first report emerged describing the activity of manganese in the hydrogenation of ketones, aldehydes, and nitriles.^4^ The hydrogenation of polar C=O and C=N bonds by manganese is well documented by Beller,^5^ Kirchner,^6^ Sortais,^7^ Milstein,^8^ Kempe,^9^ Pidko,^10^ and others.^11^ The manganese catalyzed hydrogenation of olefins, however, is less well explored with only a few examples reported in the literature.^12^

One of the main challenges of olefin hydrogenation is achieving high chemoselectivity for the desired functionality in the presence of other reducible functional groups. For example, the selective C=C bond hydrogenation of α,β-unsaturated ketones suffers either from reduction of the ketone or from over-reduction to the aliphatic alcohol. Highly chemoselective 1,4-hydrogenation of α,β-unsaturated ketones has been reported, but is primarily achieved via transfer hydrogenation utilizing precious metals.^13^ Other methodologies for selective 1,4-reduction (e.g., hydroboration/hydrosilylation) have also been reported,^14^ but only a few reports have focused on transfer-^15^ and/or direct hydrogenation with earth-abundant metals.^16^ However, these methodologies frequently require elevated hydrogen pressures (30–50 bar) or additives. Akin to many other earth-abundant metal hydrogenation catalysts,^17^ their supporting ligand allows for metal–ligand cooperativity (MLC) aiding in the facile activation of H_2_ (Figure 1A). Hydrogenation catalysts that do not rely on such cooperativity are quite rare and typically require harsh reaction conditions, leaving ample opportunity for development.

**Figure 1:**
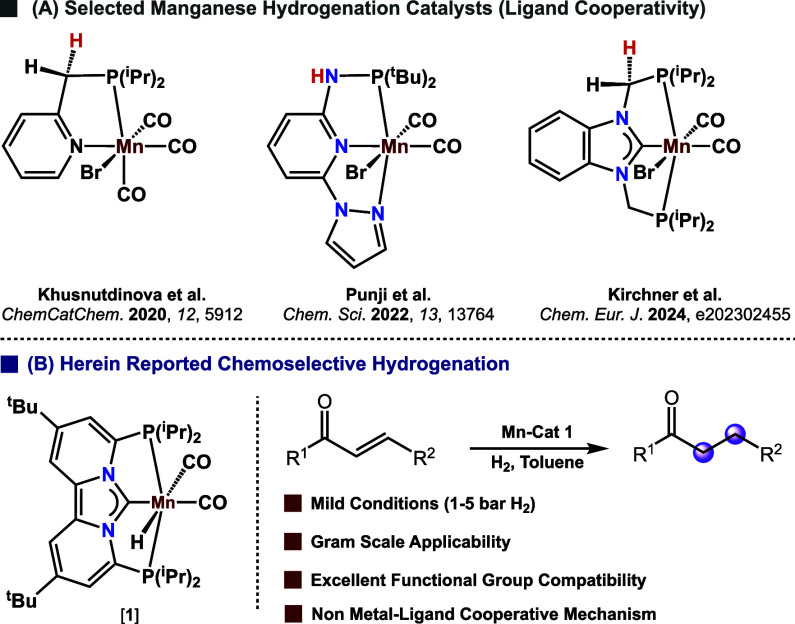
(A) Representative examples of manganese hydrogenation catalysts that use the metal–ligand cooperativity concept. (B) Herein reported Mn(I) PC_NHC_P hydride pincer catalyst (**1**) for the highly chemoselective hydrogenation of α,β-unsaturated ketones.

Recently, Punji and co-workers reported a mild methodology for the chemoselective C=C bond hydrogenation of α,β-unsaturated ketones with a (PNN) Mn(I) pincer complex (Figure 1A).^16a^ Although low hydrogen pressures could be used (i.e., 5 bar), easily reducible substrates, such as esters, amides, and aliphatic ketones, were not compatible with their reported protocol. As such, the Cp*Rh(III)H_2_ complex reported by Norton and co-workers is still the state-of-the-art in the hydrogenation of α,β-unsaturated ketones.^18^

Recently, we reported the α-methylation of ketones catalyzed by a cationic manganese(I) PC_NHC_P pincer complex, [(PC_NHC_P)Mn(CO)_3_]^+^.^19^ Our mechanistic studies indicated that initial hydride transfer from a Mn(I)-hydride complex, is responsible for the hydrogenation of the *in situ* formed α,β-unsaturated ketone.^19^ Inspired by the reactivity of the [(PC_NHC_P)Mn(CO)_2_H] complex (**1**), we envisioned that α,β-unsaturated ketones could, perhaps, also be directly hydrogenated with H_2_ in a chemoselective manner. Here we report the chemoselective 1,4-reduction of α,β-unsaturated ketones under ambient pressures of hydrogen (mostly 1–2 bar H_2_) without the need for any additional additives (Figure 1B). The herein developed methodology shows a broad tolerance toward sensitive functional groups such as esters, amides, nitriles, alkenes, and alkynes. The versatility and applicability of herein reported methodology was demonstrated by the gram-scale hydrogenation of 4-chlorochalcone.

We started with optimization of the reaction conditions by using (E)-chalcone ((*E*)-1,3-diphenylprop-2-en-1-one) as the model substrate and our previously reported PC_NHC_P Mn(I) hydride complex (**1**) as the catalyst.^19^ Upon evaluation of a wide range of reaction parameters (see Supporting Information for more information), we determined that a system composed of 5 mol % of PC_NHC_P Mn(I) hydride catalyst (**1**), at a temperature of 110 °C, with a hydrogen pressure of 2 bar, in toluene-d_8_, resulted in almost quantitative conversion of (*E*)-chalcone to its corresponding hydrogenated product. During this process, neither reduction of the ketone nor over-reduction to the aliphatic alcohol was observed. Control experiments indicated that, in the absence of catalyst **1**, or in the presence of other manganese complexes, only little hydrogenation was observed (Table S1, entries 13–19). However, as will be evident from the scope, it was sometimes necessary to adjust the hydrogen pressure or reaction time to achieve near quantitative conversion to the hydrogenated products.

With the optimized conditions established, the substrate scope of the herein reported hydrogenation reaction was investigated. For clarity we have grouped the substrates according to the hydrogen pressure that was used (Table 1; 1–5 bar).

**Table 1:**
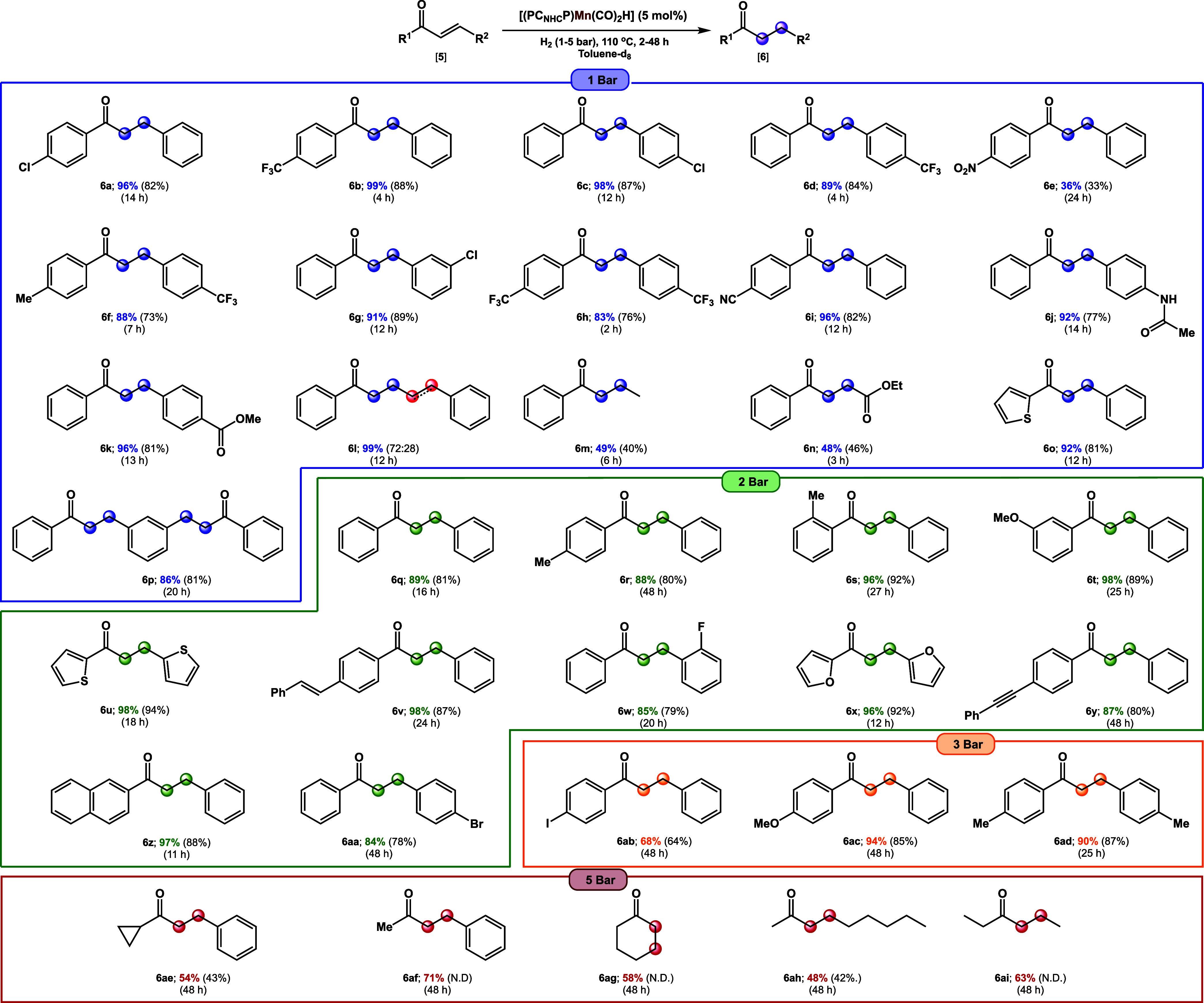
Substrate Scope of the Hydrogenation of α,β-Unsaturated Ketones Catalyzed by Complex **1**^a^

a Reaction conditions: α,β-unsaturated ketone (0.05–0.2 mmol) and manganese catalyst **1** (0.005–0.01 mmol, 5 mol %) in toluene-d_8_ (0.4 mL) with a hydrogen pressure of 1–5 bar, at 110 °C at the specified amount of time (see Supporting Information for further details). For substrate **5p**, 10 mol % catalyst was used. Yields were determined by ^1^H NMR spectroscopy of the crude reaction mixture, containing the hydrogenated products with 1,3,5-trimethoxybenzene as internal standard. Yields reported in brackets are isolated yields which have been averaged from two independent catalytic runs. For product **6ae**, yield was determined by GC analysis.

What is immediately evident from Table 1 is that, at ambient pressures (i.e., 1 bar), electron-deficient chalcones are more readily hydrogenated than electron-rich ones. For instance, chalcones bearing electron-withdrawing groups (e.g., −NO_2_, −CF_3_, −Cl, −CN) at the *para* position of either the 1- or 3-phenyl substituents are all hydrogenated efficiently and typically within 12 h (Table 1; **6a**–**6h**). Changing the substitution pattern in those substrates does not seem to influence the reaction considerably. Even electron-withdrawing groups that are susceptible to reduction (e.g., −CN, −COOR, and −CONHR) are well tolerated and only result in the selective hydrogenation of the C=C double bond of the α,β-unsaturated ketone (Table 1; **6e** and **6i**–**6k**). Similarly, changing the 3-phenyl substituents in chalcone to a -styrenyl (**6l**), -methyl (**6m**), or -acyl (**6n**) substituent resulted in overall selective hydrogenation of the C=C double bond, although for products **6m** and **6n** reduced yields were observed (Table 1). For **6l**, complete hydrogenation was also observed with a ratio of 28:72, with respect to selective α,β-hydrogenation The only outlier in this series is 2-cinnamoylthiophene, which is a more electron-rich substrate compared to (*E*)-chalcone. Notwithstanding, the overall trend seems to indicate that chalcones bearing electron-withdrawing substituents tend to be hydrogenated more readily. Such a hypothesis is consistent with the work of Brookhart and co-workers, who observed similar electronic effects in the β-alkyl migration in *para*-substituted styrenes from a Pd–Me complex.^20^

Indeed, substrates that are either similar or slightly more electron-rich than (*E*)-chalcone require a higher hydrogen pressure of 2 bar (Table 1; compare **6q** and **6z**). To illustrate, chalcones with electron-donating substituents (e.g., −Me or −OMe) at various positions among the phenyl ring (except *p*-OMe) all require increased hydrogen pressures and longer reaction times (Table 1; **6r**–**6t**). Even at these higher pressures (2 bar), reducible substituents such as alkenes and alkynes remain unaffected by our hydrogenation protocol (Table 1; **6v** and **6y**). Under these conditions, heteroaromatic substituents are also well tolerated, and their hydrogenated products **6u** and **6x** can be isolated in good yields. Only 2′-fluorochalcone is herein an outlier, as it also requires slightly elevated hydrogen pressures to achieve reasonable levels of C=C bond hydrogenation. The trend indicating that more electron-rich chalcones require higher hydrogen pressures is also validated by the fact that chalcones containing a methoxy group, or two methyl groups, in the para-position of the 1- or 3-phenyl substituent (Table 1; **6ac** and **6ad**) require about 48 h and 3 bar of hydrogen to reach >90% hydrogenation of the C=C double bond. Aliphatic α,β-unsaturated ketones require an even higher hydrogen pressure (5 bar) with only moderate conversion and yields of the hydrogenated products (Table 1; **6ae**–**6ai**).

Overall, the herein reported methodology present clear advantages compared to the state-of-the-art.^16a,18^ First and foremost, the hydrogen pressures are greatly reduced, where now only 1 or 2 bar of hydrogen are already sufficient to hydrogenate most substrates (Table 1). Second, contrary to the excellent report by Punji and co-workers,^16a^ reducible substituents such as esters and amides are well tolerated. Even aliphatic α,β-unsaturated ketones are suitable substrates, which proved to be highly problematic in their studies. In addition, external additives such as base are not required to facilitate the reaction, providing an overall reaction medium where simple filtration over silica is sufficient to isolate the products. Moreover, our methodology can easily be extended into gram-scale reactions (Scheme S3). Taking these points into consideration, our report is akin to the hallmark study reported by Norton and co-workers with rhodium,^18^ demonstrating an overall wide substrate scope, and simple reaction conditions.

With the substrate scope established, we focused our attention on elucidating the mechanism of the hydrogenation reaction because metal–ligand cooperativity is not possible in our system. Since only one coordination is available in complex **1**, we started our investigation by determining whether ligand exchange of CO with H_2_ is possible at elevated temperatures. However, heating PC_NHC_P Mn(I) complex **1** to 140 °C in the presence of 5 bar of H_2_ did not lead to any change in the ^1^H or ^31^P NMR spectrum of complex **1** (Figures S3–S5), ruling out the formation of a manganese poly hydride or H_2_-adduct.^21^ Now envisioning an outer-sphere mechanism for hydrogen/hydride transfer,^12b,22^ we performed a stoichiometric reaction between manganese catalyst **1** and α,β-unsaturated ketone **5h** (1.15 equiv). Monitoring the reaction by ^1^H NMR spectroscopy showed indeed the rapid formation of a new species, with a concomitant decrease of the hydride resonance at −8.53 ppm (Figure S6). Although this species could be characterized by NMR spectroscopy (Figures S6–S15), its reactivity toward H_2_ and its instability in the absence of H_2_ prevented us from obtaining X-ray quality crystals, even at low temperatures. Nonetheless, based on isotope labeling experiments (*vide infra*), we putatively assign this species as a manganese-enolate. Indeed, exposure of this intermediate species to an atmosphere of D_2_ (see Supporting Information for experimental details) generates the Mn-D catalyst (^31^P NMR: 140.1 ppm: *J*_P-D_ = 7.3 Hz, see Figure S13) concomitant with the release of the saturated ketone that is exclusively deuterated at the α-position (Figure S10). The minor amount of deuteration observed at the β-position results from the excess enone (*ca*. 0.3 equiv) that was present and was subsequently hydrogenated when the D_2_ gas was introduced. Control experiments indicated that the deuteration at the α-position does not result from manganese catalyzed H/D exchange of the formed ketone with D_2_ (Figures S16–S20 and Schemes S7–S8), suggesting that the observed α-deuteration is the result of heterolytic cleavage of D_2_ from the putative manganese-enolate intermediate.

Taken together, these data indicate that reaction is initiated by outer-sphere hydride transfer from the catalyst to the α,β-unsaturated ketone via **Pro-1**, resulting in the formation of an equilibrium mixture of the manganese enolate (Scheme 1; **Int**-**1A**/**1B**). Subsequent heterolytic H_2_ cleavage by the in situ formed manganese enolate **Int**-**1A** regenerates the catalyst and produces the saturated ketone. These data are further corroborated by the large kinetic isotope effect (*k*_H_/*k*_D_ > 3) observed upon the hydrogenation of **5c** in the presence of D_2_ (Scheme S4). A catalytic cycle depicting these mechanistic steps is shown in Scheme 1.

**Scheme 1:**
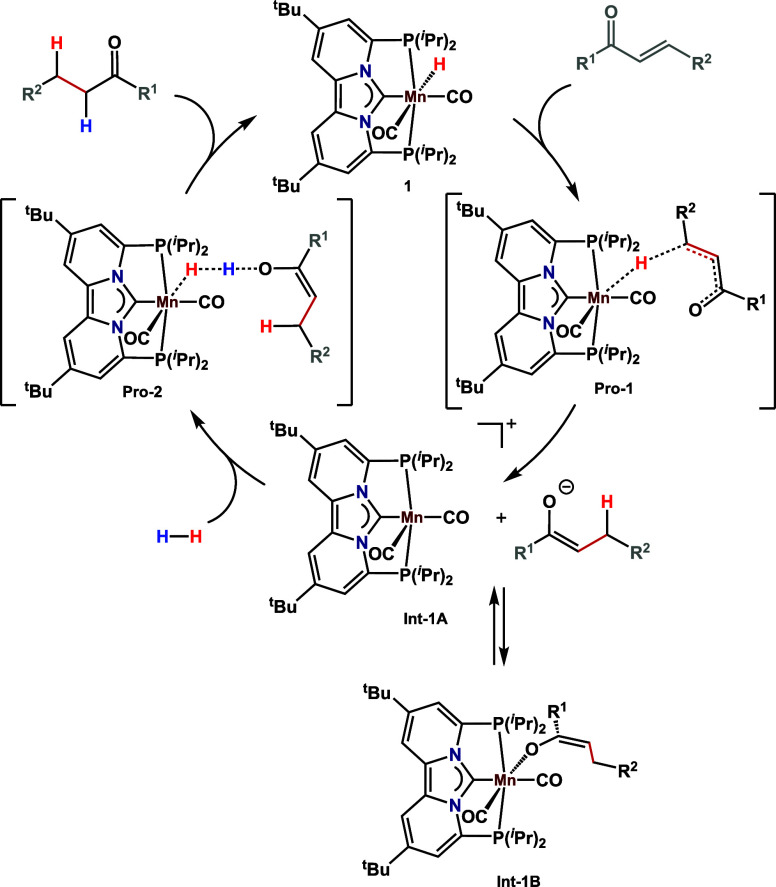
Plausible Mechanism for the Hydrogenation of α,β-Unsaturated Ketones Catalyzed by Complex **1**

In summary, we have developed a mild and efficient protocol for the highly chemoselective hydrogenation of the C=C double bond of α,β-unsaturated ketones. Our methodology is compatible with a wide array of sensitive functional groups such as halides, nitro, esters, amides, alkenes, alkynes, and even aliphatic α,β-unsaturated ketones, albeit in lower yields. Aldehydes unfortunately are still not compatible, as they readily reduce to the primary alcohol. Our studies also indicated a clear electronic effect, where more electron-poor substrates are hydrogenated at lower pressures and with shorter reaction times. Moreover, our mechanistic studies indicated an outer-sphere mechanism for hydride transfer, which is followed by heterolytic cleavage of H_2_, by the *in situ* formed manganese-enolate. Overall, the methodology produces saturated ketones in good yields in a simple protocol, generally requiring only 1 or 2 bar of hydrogen pressure.

## Data Availability

The data underlying this is study are available at the published article and its Supporting Information.

## Supporting Information Available

Additional experimental details, characterization data, and isotope labeling studies (PDF)
