# Supplementary material for: Chemoselective Hydrogenation of α,β-Unsaturated Ketones Catalyzed by a Manganese(I) Hydride Complex
Source: Org Lett. 2024 May 13;26(20):4173–7. doi: 10.1021/acs.orglett.4c00277 (PMC11129310; doi:10.1021/acs.orglett.4c00277)
Supplement: Supplementary file 1 — ol4c00277_si_001.pdf [file ol4c00277_si_001.pdf]

Supporting Information

Chemoselective Hydrogenation of α, β-Unsaturated Ketones Catalyzed by a Manganese (I) Hydride Complex

Kartick Dey,[a] and Graham de Ruiter,*[a]

[a]Schulich Faculty of Chemistry, Technion – Israel Institute of Technology; Technion City, Haifa 3200008, Israel.

Email: graham@technion.ac.il

Table of Contents:

General Procedures……………………………………………………………………..S3

Synthesis and characterization of enone substrates…………………………………….S3

Optimization of reaction conditions…………………………………………………….S6

General procedure of hydrogenation of enones………………………………………...S9

Characterization data of hydrogenated enones………………………………………..S10

Gram scale hydrogenation of enone 5c………………………………………………..S21

Mechanistic Studies…………………………………………………………………….S22

NMR and mass spectra of selected enone substrates…………………………………..S41

NMR spectra of crude and isolated products………………………………………… S50

Yield analysis of product 6ag by Gas Chromatography…………………………...….S104

References……………………………………………………………………………….S113

General Procedures:

All reactions were performed at room temperature either by using Schlenk techniques or by using a N_2-filled Glovebox unless otherwise specified. Glassware was oven dried at 150 °C for at least 2 h prior to use and were allowed to cool under vacuum. All reagents were used as received unless mentioned otherwise. Commercially available chemicals reported herein were purchased from Sigma-Aldrich, Alfa Aesar, Acros Orgaics and Thermo Scientific. Enones, **5m**, **5n**, and **5af-5ai** were purchased from Sigma Aldrich and used as received. Manganese hydride complex **1** $[(PC_{NHC}P)Mn(CO)_2H]$ was synthesized according to our previously reported procedure.[1] Anhydrous unstabilized tetrahydrofuran (THF), dichloromethane, and diethyl ether (Et_2O) were purchased from Sigma-Aldrich and and purified by filtering over activated alumina under air-free conditions. Anhydrous toluene was purchased from Sigma-Aldrich, dried over calcium hydride, degassed by three freeze−pump−thaw cycles, and vacuum-transferred prior to use. The ^1H, ^{13}C, ^{19}F, and ^{31}P NMR spectra were recorded on Bruker either a AVANCE 200 MHz or 300 MHz NMR spectrometer, or an a AVANCE II NMR 400 MHz spectrometer, or on a AVANCE III 600 MHz NMR spectrometer at room temperature unless mentioned otherwise. All chemical shifts (δ) are reported in ppm and coupling constants (*J*) are given in Hz. The ^1H and $^{13}C\{1H\}$ NMR spectra were referenced using residual H-impurities in the deuterated solvents. Deuterated solvents (Toluene-d_8 and $CDCl_3$) were purchased from Cambridge Isotope Laboratories, dried over calcium hydride, degassed by three freeze−pump−thaw cycles, and vacuum-transferred prior to use. Positive Atmospheric Pressure Chemical Ionization (MS $APCI^+$) and high-resolution mass spectrometry (HRMS) were recorded on a Bruker maXis impact solid probe. Gas chromatography was performed on a Agilent 7820A GC system using dodecane as internal standard.

Synthesis and characterization of enone substrates:

Substrates **5a**–**5j**, **5l**, **5o**–**5u**, **5w**–**5x**, and **5z**–**5ae** were synthesized according to a reported literature procedures, whose general outline is detailed below.[2] Enone **5k** was prepared according to a different procedure,[3] while substrates **5v**[4] and **5y**[5] were synthesized according to literature procedures starting from (*E*)-1-(4-bromophenyl)-3-phenylprop-2-en-1-one which was synthesized following the general procedure outlined below:

General procedure: An oven dried 100 mL round bottom flask was charged with NaOH (500 mg, 12.5 mmol) and water (40 mL). The mixture was stirred until complete dissolution of NaOH. Hereafter, a mixture of the corresponding ketone derivative (10 mmol) and aldehyde derivative (10 mmol) in ethanol (40 mL) was slowly added to the aqueous NaOH solution under continuous stirring. The reaction mixture was stirred overnight at room temperature. After completion of reaction, the work-up of the reaction proceeded via two different pathways: (1) if no acid sensitive functionality is present, the reaction mixture was neutralized using HCl (2 M) until pH of the reaction mixture reached 7. If the product precipitated from the reaction mixture, it was collected by filtration, and the crude product was washed repeatedly with distilled water (5 × 50 ml). If however, the product did not precipitate, the enone (e.g., **5s** and **5t**) was extracted using ethyl acetate (3 × 50 ml). The organics were subsequently pooled together, washed with brine, dried over Na_2SO_4, and was purified by column chromatography (SiO_2, ethyl acetate/hexane). (2) if acid sensitive functional groups are present, the product mixture was not neutralized. Instead, it was repeatedly washed with distilled water until the pH of the eluted water appeared as 7. The solid products from both cases were dried in open air and were recrystallized from hot ethanol.

Characterization of synthesized enones:

Synthesized enones **5a–5g, 5i–5l, 5o, 5q–5s**, and **5v–5ae** were characterized by ^{1}H, ^{13}C, and ^{19}F NMR spectroscopy and matched with literature reported data,[2-5] while the characterization data for substrates **5h**, **5p**, **5t**, and **5u** are reported below:

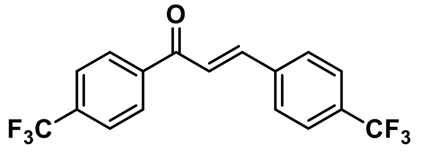

(*E*)-1,3-bis(4-(trifluoromethyl)phenyl)prop-2-en-1-one (5h): Synthesized according to general procedure described above. The crude product was purified by recrystallization from hot ethanol. Isolated as white crystalline solid (prism). Isolated Yield: 1226 mg (36%). **^{1}H NMR** (400 MHz, $CDCl_3$) δ (ppm): 8.12 (d, *J* = 8.3 Hz, 2H), 7.84 (d, *J* = 15.8 Hz, 1H), 7.79 (d, *J* = 8.3 Hz, 2H), 7.76 (d, *J* = 8.3 Hz, 2H), 7.69 (d, *J* = 8.3 Hz, 2H), 7.56 (d, *J* = 15.8 Hz, 1H). **^{13}C{^{1}H} NMR** (101 MHz, $CDCl_3$) δ (ppm): 189.3, 144.1, 140.7, 138.0, 134.5 (q, *J* = 32.7 Hz), 132.4 (q, *J* = 32.7 Hz), 129.0, 128.8, 126.1 (q, *J* = 3.7 Hz), 125.9 (q, *J* = 3.6 Hz), 125.2 (d, *J* = 16.6 Hz), 123.8, 122.5 (d, *J* = 17.1 Hz). **^{19}F NMR** (377 MHz, $CDCl_3$) δ (ppm): -62.89, -63.06. **HRMS (APCI$^+$, m/z):** calculated for $[C_{17}H_{11}F_6O]^+$, 345.0709; found, 345.0734.

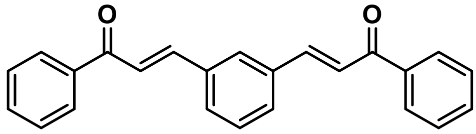

(2*E*,2'*E*)-3,3'-(1,3-phenylene)bis(1-phenylprop-2-en-1-one) (5p): Synthesized according to general procedure described above. The crude product was purified by recrystallization from hot ethanol. Isolated as white crystalline solid (prism). Isolated Yield: 1510 mg (45%). **^{1}H NMR** (400 MHz, $CDCl_3$) δ (ppm): 8.04 (d, *J* = 7.4 Hz, 4H), 7.88 (s, 1H), 7.83 (d, *J* = 15.7 Hz, 2H), 7.69 (dd, *J* = 7.7, 0.9 Hz, 2H), 7.60 (dd, *J* = 15.1, 6.5 Hz, 4H), 7.55 – 7.45 (m, 5H).**^{13}C{^{1}H} NMR** (101 MHz, $CDCl_3$) δ (ppm): 190.4, 143.9, 138.1, 135.8, 133.1, 130.2, 129.7, 128.8, 128.7, 128.4, 123.1. **HRMS (APCI$^+$, m/z):** calculated for $[C_{24}H_{19}O_2]^+$, 339.1380; found, 339.1397.

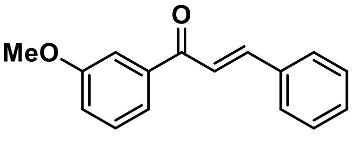

(*E*)-1-(3-methoxyphenyl)-3-phenylprop-2-en-1-one (5t): Synthesized according to general procedure described above. The crude product was purified by column chromatography (SiO_2; ethyl acetate:hexane, 5:95 (v/v)). Isolated as yellow oil. Isolated Yield: 930 mg (39%).**^{1}H NMR** (400 MHz, $CDCl_3$) δ (ppm): 7.82 (d, *J* = 15.7 Hz, 1H), 7.65 (dd, *J* = 6.3, 2.9 Hz, 2H), 7.61 (d, *J* = 7.6 Hz, 1H), 7.55 (brs, 1H), 7.52 (d, *J* = 15.7 Hz, 1H), 7.46 – 7.38 (m, 4H), 7.14 (dd, *J* = 8.2, 2.6 Hz, 1H), 3.89 (s, 3H).**^{13}C{^{1}H} NMR** (101 MHz, $CDCl_3$) δ (ppm): 190.4, 160.0, 145.0, 139.7, 135.0, 130.7, 129.7, 129.1, 128.6, 122.3, 121.2, 119.4, 113.0, 55.6. **HRMS (APCI$^+$, m/z):** calculated d for $[C_{16}H_{15}O_2]^+$, 239.1067; found, 239.1061.

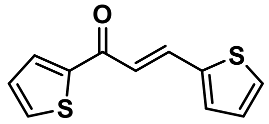

(*E*)-1,3-di(thiophen-2-yl)prop-2-en-1-one (5u): Synthesized according to general procedure described above. The crude product was purified by recrystallization from hot ethanol. Isolated as light yellow crystalline solid (needles). Isolated Yield: 2095 mg (95%). **^{1}H NMR** (400 MHz, $CDCl_3$) δ (ppm): 7.97 (d, *J* = 15.2 Hz, 1H), 7.84 (d, *J* = 3.8 Hz, 1H), 7.67 (d, *J* = 4.9 Hz, 1H), 7.43 (d, *J* = 5.0 Hz, 1H), 7.37 (d, *J* = 3.6 Hz, 1H), 7.24 – 7.15 (m, 2H), 7.12 – 7.07 (m, 1H).**^{13}C{^{1}H} NMR** (101 MHz, $CDCl_3$) δ (ppm): 181.7, 145.6, 140.3, 136.6, 134.0, 132.3, 131.8, 129.0, 128.5, 128.4, 120.6. **HRMS (APCI$^+$, m/z):** calculated d for $[C_{11}H_9OS_2]^+$, 221.0089; found, 221.0115.

Optimization of reaction conditions:

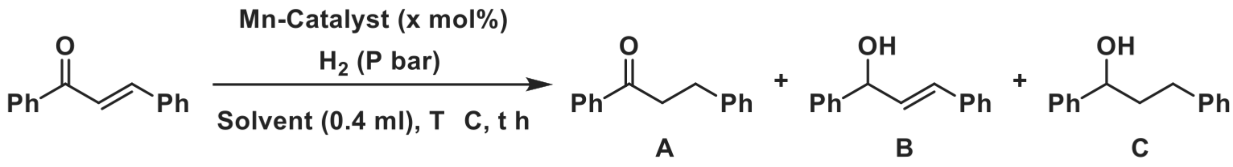

Scheme S1: General procedure for optimization of the reaction conditions for the chemoselective hydrogenation of enones.

Inside a N_2-filled glovebox, a J. Young NMR tube suitable for intermediate pressure (up to 10.5 bar) was charged with *trans*-chalcone (20.82 mg, 0.1 mmol), Mn catalyst (x mol%) and 0.4 ml of specified solvent was added into the NMR tube (**Table 1**). The tube was sealed and taken out of the glovebox. Hereafter, the tube was degassed by freeze-pump-thaw cycles (3×) using Schlenk techniques and connected to an intermediate pressure hydrogen line. The line was purged by subsequent evacuation and filling with H_2 (3×). The tube was opened and was filled with hydrogen at desired pressure. Next, the tube was sealed again and placed on a preheated oil bath (T °C). In case a deuterated solvent was used, the reaction progress was monitored by ^{1}H-NMR spectroscopy at room temperature (maximum reaction time was 48 h). Upon completion of the reaction, the reaction mixture was cooled to room temperature and the contents of the J. Young tube were transferred to a 20 ml vial. The tube was washed repeatedly (5 × 1 mL) with an ethyl acetate/hexane (10/90; v/v) mixture, and to the combined organics was added 1,3,5-trimethoxybenzene (0.33 equiv.) as internal standard. Then, the combined organic phases filtered through a short alumina pad (4 cm; 7 mm O.D) followed by repeated washing with 10% ethyl acetate in hexane as eluent (15 mL) to separate the organics from the manganese catalyst. Hereafter, the solvent was evaporated under reduced pressure and the ^{1}H NMR was recorded to analyze the NMR yield and product ratio.

Table S1: Optimization of the reaction conditions

Entry	Mn-Catalyst	Cat loading (x mol%)	Solvent	Temp. (°C)	Pressure (bar)	Time (h)	Yield of A (%)	Yield of B (%)	Yield of C (%)
1.	**1**	3	Tol-d8	140	1	48	40	ND	<1
2.	**1**	3	Tol-d8	140	2	10	95	ND	<1
3.	**1**	1	Tol-d8	140	2	48	42	ND	<1
4.	**1**	2	Tol-d8	140	2	30	87	ND	<1
5.	**1**	3	Tol-d8	110	2	30	64	ND	ND
6.	**1**	*5*	*Tol-d8*	*110*	*2*	*16*	*94*	ND	*ND*
7.	**1**	5	C_6D_6	110	2	16	66	ND	ND
8.	**1**	5	THF-d8	110	2	16	50	ND	ND
9[a]	**1**	5	CH_3CN	110	2	16	23	ND	ND
10.	**1**	5	Hexane	110	2	16	6	ND	<1
11.	**1**	5	Et_2O	50	2	16	3	ND	<1
12.	**1**	5	CH_2Cl_2	50	2	16	9	ND	ND
13.	-	-	Tol-d8	110	2	16	ND	ND	ND
14.	**2**	5	Tol-d8	110	2	16	5	ND	ND
15[b].	**2**	5	CH_2Cl_2	50	2	16	ND	ND	ND
16.	**3**	5	Tol-d8	110	2	16	<1	ND	ND
17.	**4**	5	Tol-d8	110	2	16	3	ND	ND
18[c].	**3**	5	Tol-d8	110	2	16	3	ND	1
19[c].	**4**	5	Tol-d8	110	2	16	24	ND	3

[a]Acetonitrile activation was observed. [b]Because of insolubility of complex **2** in toluene-d8, the reaction was performed in CH_2Cl_2.[c]10 mol% KO^{t}Bu was used to activate the catalyst.

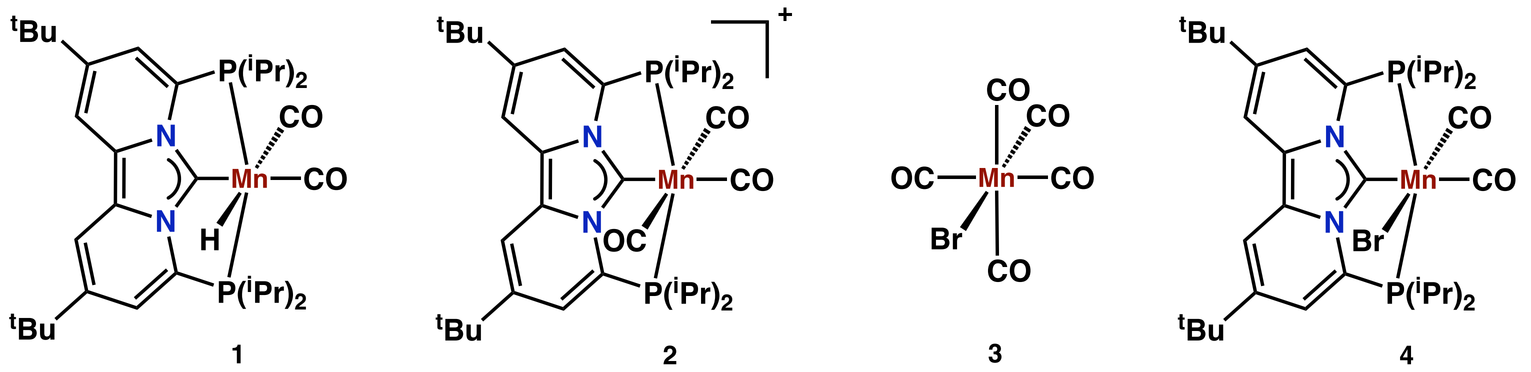

Chart S1. Manganese catalysts used in this study (see Table S1).

General procedure of hydrogenation of enones:

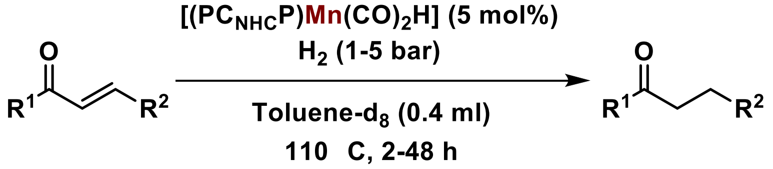

Scheme S2: General procedure for chemoselective hydrogenation of enones**.**

Inside a N_2-filled glovebox, a J. Young NMR tube suitable for intermediate pressure (up to 10.5 bar) was charged with substrate (0.1/0.2 mmol, or 0.05 mmol), manganese hydride catalyst **1** [(PC$_{NHC}$P)Mn(CO)$_2$H] (5 mol%; 0.1/0.2 mL from a 50 mM stock solution of **1** in toluene), and toluene-d$_8$ to make the total volume of 0.4 mL. The tube was sealed and was taken out of the glovebox. Hereafter, the tube was degassed by freeze-pump-thaw cycles (3×) using Schlenk techniques and connected to an intermediate pressure hydrogen line. The line was purged by subsequent evacuation and filling with H_2 (3×). The tube was opened and was filled with hydrogen at desired pressure (1-5 bar). Next, the tube was sealed again and placed on a preheated oil bath at 110 °C. The reaction progress was monitored by ^{1}H-NMR spectroscopy at room temperature. Either (i) after >90% substrate consumption or (ii) after a maximum of 48 h, the reaction mixture was cooled to room temperature, and the contents of the J. Young tube were transferred to a 20 ml vial. The J. Young tube was repeatedly rinsed with dichloromethane (5 × 0.6 mL). Then, the reaction mixture was filtered through a short alumina pad (4 cm; 7 mm O.D) using a gradient of either ethyl acetate/hexane mixture or 0-2 % methanol in dichloromethane (depending on the polarity and solubility of the product) to separate the organic products from the manganese catalyst. The complete elution of the organic products from the alumina pad was confirmed by thin layer chromatography analysis. Hereafter, the solvent was evaporated under reduced pressure and the crude reaction mixture was purified by silica gel column. For every substrate, reactions were run in duplicate. In one reaction, 1,3,5-trimethoxybenzene (0.33 equiv.) was added as internal standard to the crude reaction mixture after filtration over alumina to determine the yield by ^{1}H-NMR spectroscopy. For substrate 5ae the yield was determined by Gas Chromatography. Isolated yields have been averaged from two independent catalysis runs.

Characterization data of hydrogenated enones

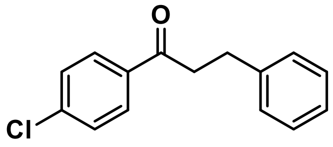

1-(4-chlorophenyl)-3-phenylpropan-1-one (6a): Conditions, 0.1 mmol scale with 1 bar H_2. Isolated as a white amorphous solid by column chromatography (SiO_2; ethyl acetate:hexane, 1:99 (v/v)). Yield: 20.1 mg (82%). **^{1}H NMR** (400 MHz, $CDCl_3$) δ (ppm): 7.89 (d, *J* = 8.6 Hz, 2H), 7.42 (d, *J* = 8.5 Hz, 2H), 7.32 – 7.28 (m, 2H), 7.22 (dd, *J* = 16.2, 7.4 Hz, 3H), 3.27 (t, *J* = 7.7 Hz, 2H), 3.06 (t, *J* = 7.7 Hz, 2H). **^{13}C{^{1}H} NMR** (101 MHz, $CDCl_3$) δ (ppm): 198.1, 141.2, 139.6, 135.2, 129.6, 129.0, 128.7, 128.5, 126.3, 40.5, 30.1. The NMR data was in accordance with the literature.[6]

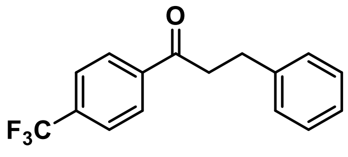

3-phenyl-1-(4-(trifluoromethyl)phenyl)propan-1-one (6b): Conditions, 0.1 mmol scale with 1 bar H_2. Isolated as a pale yellow oil by column chromatography (SiO_2; ethyl acetate:hexane, 1:99 (v/v)). Yield: 24.5 mg (88%). **^{1}H NMR** (400 MHz, $CDCl_3$) δ (ppm): 8.04 (d, *J* = 8.2 Hz, 2H), 7.71 (d, *J* = 8.2 Hz, 2H), 7.33 -7.29 (m, 2H), 7.26 – 7.20 (m, 3H), 3.32 (t, *J* = 7.6 Hz, 2H), 3.08 (t, *J* = 7.6 Hz, 2H). **^{13}C{^{1}H} NMR** (101 MHz, $CDCl_3$) δ (ppm): 198.3, 141.0, 139.5, 134.5 (q, *J* = 32.6 Hz), 128.7, 128.5, 128.5, 126.4, 125.8 (q, *J* = 3.7 Hz), 123.7 (q, *J* = 273.6 Hz), 40.9, 30.0. **^{19}F NMR** (377 MHz, $CDCl_3$) δ (ppm): -63.05. The NMR data was in accordance with the literature.[6]

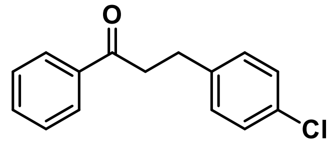

3-(4-chlorophenyl)-1-phenylpropan-1-one (6c): Conditions, 0.1 mmol scale with 1 bar H_2. Isolated as a white amorphous solid by column chromatography (SiO_2; ethyl acetate:hexane, 1:99 (v/v)). Yield: 21.2 mg (87%). **^{1}H NMR** (400 MHz, $CDCl_3$) δ (ppm): 7.95 (d, *J* = 7.4 Hz, 2H), 7.57 (t, *J* = 7.4 Hz, 1H), 7.46 (t, *J* = 7.6 Hz, 2H), 7.26 (d, *J* = 8.4 Hz, 2H), 7.19 (d, *J* = 8.3 Hz, 2H), 3.29 (t, *J* = 7.5 Hz, 2H), 3.05 (t, *J* = 7.5 Hz, 2H). **^{13}C{^{1}H} NMR** (101 MHz, $CDCl_3$) δ (ppm): 199.0, 139.9, 136.9, 133.3, 132.0, 130.0, 128.8, 128.7, 128.1, 40.3, 29.5. The NMR data was in accordance with the literature.[6]

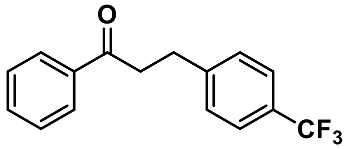

1-phenyl-3-(4-(trifluoromethyl)phenyl)propan-1-one (6d): Conditions, 0.1 mmol scale with 1 bar H_2. Isolated as a pale yellow oil by column chromatography (SiO_2; ethyl acetate:hexane, 1:99 (v/v)). Yield: 23.3 mg (84%). **^{1}H NMR** (400 MHz, $CDCl_3$) δ (ppm): 7.99 – 7.93 (m, 2H), 7.61 – 7.50 (m, 3H), 7.46 (t, *J* = 7.6 Hz, 2H), 7.38 (d, *J* = 8.0 Hz, 2H), 3.33 (t, *J* = 7.5 Hz, 2H), 3.14 (t, *J* = 7.5 Hz, 2H). **^{13}C{^{1}H} NMR** (101 MHz, $CDCl_3$) δ (ppm): 198.7, 145.6, 136.7, 133.4, 129.1, 128.9, 128.8, 128.8, 128.4,128.1, 125.5 (q, *J* = 3.8 Hz). 124.4 (q, *J* = 271.6 Hz), 39.9, 29.9. **^{19}F NMR** (377 MHz, $CDCl_3$) δ (ppm): -62.30. The NMR data was in accordance with the literature.[6]

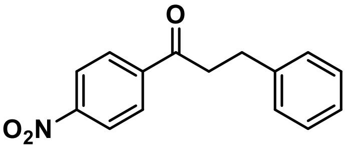

1-(4-nitrophenyl)-3-phenylpropan-1-one (6e): Conditions, 0.1 mmol scale with 1 bar H_2. Isolated as a yellow amorphous solid by column chromatography (SiO_2; ethyl acetate:hexane, 2:98 (v/v)). Yield: 8.4 mg (33%). **^{1}H NMR** (400 MHz, $CDCl_3$) δ (ppm): 8.30 (d, *J* = 8.8 Hz, 2H), 8.09 (d, *J* = 8.8 Hz, 2H), 7.31 (t, *J* = 7.4 Hz, 2H), 7.27 – 7.19 (m, 3H), 3.35 (t, *J* = 7.5 Hz, 2H), 3.09 (t, *J* = 7.5 Hz, 2H). **^{13}C{^{1}H} NMR** (101 MHz, $CDCl_3$) δ (ppm): 197.8, 150.4, 141.3, 140.7, 129.2, 128.8, 128.5, 126.5, 124.0, 41.2, 30.0. The NMR data was in accordance with the literature.[7]

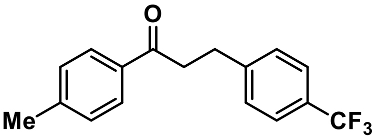

1-(p-tolyl)-3-(4-(trifluoromethyl)phenyl)propan-1-one (6f): Conditions, 0.1 mmol scale with 1 bar H_2. Isolated as a white amorphous solid by column chromatography (SiO_2; ethyl acetate:hexane, 1:99 (v/v)). Yield: 21.3 mg (73%). **^{1}H NMR** (400 MHz, $CDCl_3$) δ (ppm): 7.85 (d, *J* = 8.1 Hz, 2H), 7.54 (d, *J* = 8.0 Hz, 2H), 7.36 (d, *J* = 8.0 Hz, 2H), 7.25 (d, *J* = 8.0 Hz, 2H), 3.29 (t, *J* = 7.5 Hz, 2H), 3.12 (t, *J* = 7.5 Hz, 2H), 2.40 (s, 3H). **^{13}C{^{1}H} NMR** (101 MHz, $CDCl_3$) δ (ppm): 198.4, 145.7, 144.2, 134.3, 129.5, 128.9, 128.7, 128.4, 128.3, 125.8, 125.5 (q, *J* = 3.8 Hz), 123.1, 39.9, 29.9, 21.8. **^{19}F NMR** (377 MHz, $CDCl_3$) δ (ppm): -62.30. The NMR data was in accordance with the literature.[8]

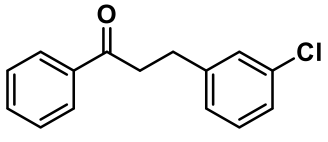

3-(3-chlorophenyl)-1-phenylpropan-1-one (6g): Conditions, 0.1 mmol scale with 1 bar H_2. Isolated as a white amorphous solid by column chromatography (SiO_2; ethyl acetate:hexane, 1:70 (v/v)). Yield: 21.8 mg (89%). ^{1}**H NMR** (400 MHz, $CDCl_3$) δ (ppm): 7.97 (d, *J* = 7.4 Hz, 2H), 7.58 (t, *J* = 7.4 Hz, 1H), 7.47 (t, *J* = 7.6 Hz, 2H), 7.26 (s, 1H), 7.23 (d, *J* = 7.4 Hz, 1H), 7.21 – 7.17 (m, 1H), 7.15 (d, *J* = 7.2 Hz, 1H), 3.31 (t, *J* = 7.6 Hz, 2H), 3.06 (t, *J* = 7.6 Hz, 2H). ^{13}**C{^{1}H} NMR** (101 MHz, $CDCl_3$) δ (ppm): 198.8, 143.5, 136.8, 134.3, 133.3, 129.9, 128.8, 128.7, 128.1, 126.8, 126.5, 40.1, 29.8. The NMR data was in accordance with the literature.[9]

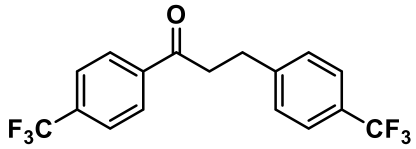

1,3-bis(4-(trifluoromethyl)phenyl)propan-1-one (6h): Conditions, 0.1 mmol scale with 1 bar H_2. Isolated as a white amorphous solid by column chromatography (SiO_2; ethyl acetate:hexane, 1:99 (v/v)). Yield: 26.3 mg (76%). ^{1}**H NMR** (400 MHz, $CDCl_3$) δ (ppm): 8.05 (d, *J* = 8.1 Hz, 2H), 7.73 (d, *J* = 8.2 Hz, 2H), 7.56 (d, *J* = 8.0 Hz, 2H), 7.37 (d, *J* = 8.0 Hz, 2H), 3.35 (t, *J* = 7.4 Hz, 2H), 3.15 (t, *J* = 7.4 Hz, 2H). ^{13}**C{^{1}H} NMR** (101 MHz, $CDCl_3$) δ (ppm): 197.7, 145.1, 139.4, 134.7 (q, *J* = 32.6 Hz), 129.0, 128.9, 128.7, 128.5, 128.4, 128.3, 125.9 (q, *J* = 3.7 Hz), 125.6 (q, *J* = 3.8 Hz), 125.0, 123.0, 122.3, 40.3, 29.7. ^{19}**F NMR** (377 MHz, $CDCl_3$) δ (ppm): -62.39, -63.13. The NMR data was in accordance with the literature.[10]

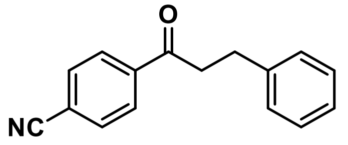

4-(3-phenylpropanoyl)benzonitrile (6i): Conditions, 0.1 mmol scale with 1 bar H_2. Isolated as a white amorphous solid by column chromatography (SiO_2; ethyl acetate:hexane, 10:90 (v/v)). Yield: 19.3 mg (82%). ^{1}**H NMR** (400 MHz, $CDCl_3$) δ (ppm): 8.03 (d, *J* = 8.4 Hz, 2H), 7.76 (d, *J* = 8.4 Hz, 2H), 7.31 (t, *J* = 7.4 Hz, 2H), 7.23 (dd, *J* = 12.9, 7.0 Hz, 3H), 3.32 (t, *J* = 7.5 Hz, 2H), 3.08 (t, *J* = 7.5 Hz, 2H). ^{13}**C{^{1}H} NMR** (101 MHz, $CDCl_3$) δ (ppm): 197.9, 140.8, 139.8, 132.6, 128.7, 128.5, 128.5, 126.5, 118.0, 116.5, 40.9, 29.9. The NMR data was in accordance with the literature.[11]

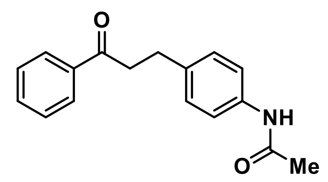

N-(4-(3-oxo-3-phenylpropyl)phenyl)acetamide (6j): Conditions, 0.1 mmol scale with 1 bar H_2. Isolated as a white amorphous solid by column chromatography (SiO_2; ethyl acetate:hexane, 30:70 (v/v)). Yield: 20.6 mg (77%). **^{1}H NMR** (400 MHz, $CDCl_3$) δ (ppm): 7.95 (d, *J* = 7.4 Hz, 2H), 7.56 (t, *J* = 7.4 Hz, 1H), 7.46 (t, *J* = 7.7 Hz, 2H), 7.42 (d, *J* = 8.3 Hz, 2H), 7.26 (s, 1H), 7.20 (d, *J* = 8.2 Hz, 2H), 3.28 (t, *J* = 7.6 Hz, 2H), 3.03 (t, *J* = 7.6 Hz, 2H), 2.16 (s, 3H). **^{13}C{^{1}H} NMR** (101 MHz, $CDCl_3$) δ (ppm): 199.4, 168.4, 137.4, 136.9, 136.1, 133.2, 129.1, 128.8, 128.2, 120.4, 40.5, 29.6, 24.7. The NMR data was in accordance with the literature.[7]

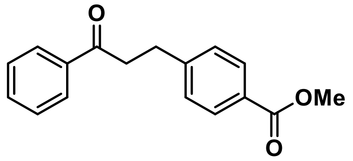

Methyl 4-(3-oxo-3-phenylpropyl)benzoate (6k): Conditions, 0.1 mmol scale with 1 bar H_2. Isolated as a white amorphous solid by column chromatography (SiO_2; ethyl acetate:hexane, 4:96 (v/v)). Yield: 21.7 mg (81%). **^{1}H NMR** (400 MHz, $CDCl_3$) δ (ppm): 8.00 – 7.92 (m, 4H), 7.56 (t, *J* = 7.4 Hz, 1H), 7.46 (t, *J* = 7.6 Hz, 2H), 7.32 (d, *J* = 8.1 Hz, 2H), 3.90 (s, 3H), 3.33 (t, *J* = 7.5 Hz, 2H), 3.13 (t, *J* = 7.5 Hz, 2H). **^{13}C{^{1}H} NMR** (101 MHz, $CDCl_3$) δ (ppm): 198.8, 167.2, 146.9, 136.7, 133.4, 130.0, 128.8, 128.6, 128.2, 128.1, 52.2, 39.9, 30.1. The NMR data was in accordance with the literature.[3]

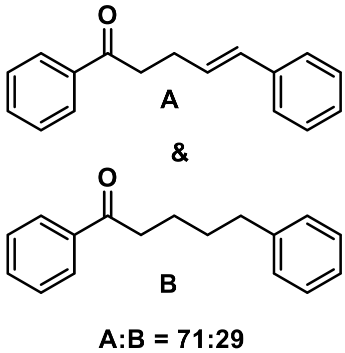

A:B = 71:29
(Isolated mixture)

(E)-1,5-diphenylpent-4-en-1-one and 1,5-diphenylpentan-1-one (6l): Conditions, 0.1 mmol scale with 1 bar H_2. Isolated as a colorless oil by column chromatography (SiO_2; ethyl acetate:hexane, 10:90 (v/v)). Yield: 22.7 mg (96%). Isolated as 71:29 (A:B) mixture. **^{1}H NMR** (400 MHz, $CDCl_3$) δ (ppm): 8.01 – 7.89 (m, 2H, Ar-H), 7.55 (q, *J* = 7.2 Hz, 1H, Ar-H), 7.45 (dd, *J* = 15.1, 7.6 Hz, 2H, Ar-H), 7.36 – 7.14 (m, 5H, Ar-H), 6.46 (d, *J* = 15.9 Hz, 0.71H), 6.29 (dt, *J* = 15.8, 6.8 Hz, 0.71H), 3.15 (t, *J* = 7.4 Hz, 1.42H), 2.98 (t, *J* = 7.1 Hz, 0.58H), 2.70 - 2.60 (m, 1.90H), 1.84 – 1.65 (m, 1.16H). **^{13}C{^{1}H} NMR** (101 MHz, $CDCl_3$) δ (ppm): 200.4, 199.5, 142.4, 137.6, 137.1, 137.0, 133.2, 133.1, 130.9, 129.3, 128.8, 128.7, 128.6, 128.5, 128.4, 128.2, 128.2, 127.2, 126.2, 125.9, 38.5, 38.4, 35.9, 31.2, 27.6, 24.1. The NMR data was in accordance with the literature.[6, 12]

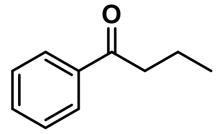

1-phenylbutan-1-one (6m): Conditions, 0.1 mmol scale with 1 bar H_2. Isolated as a pale yellow oil by preparative thin layer chromatography (SiO_2; ethyl acetate:hexane, 15:85 (v/v)). Yield: 5.9 mg (40%). **^{1}H NMR** (400 MHz, $CDCl_3$) δ (ppm): 7.96 (d, J = 7.3 Hz, 2H), 7.55 (t, J = 7.3 Hz, 1H), 7.46 (t, J = 7.6 Hz, 2H), 2.95 (t, J = 7.3 Hz, 2H), 1.82 – 1.72 (m, 2H), 1.01 (t, J = 7.4 Hz, 3H). **^{13}C{^{1}H} NMR** (101 MHz, $CDCl_3$) δ (ppm): 200.6, 137.2, 133.0, 128.7, 128.2, 40.6, 17.9, 14.0. The NMR data was in accordance with the literature.[13]

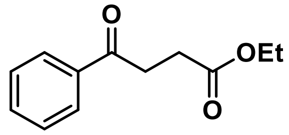

Ethyl 4-oxo-4-phenylbutanoate (6n): Conditions, 0.1 mmol scale with 1 bar H_2. Isolated as a colorless oil by column chromatography (SiO_2; ethyl acetate:hexane, 4:96 (v/v)). Yield: 9.5 mg (46%). **^{1}H NMR** (400 MHz, $CDCl_3$) δ (ppm): 8.02 – 7.94 (m, 2H), 7.57 (t, J = 7.4 Hz, 1H), 7.46 (t, J = 7.6 Hz, 2H), 4.16 (q, J = 7.1 Hz, 2H), 3.31 (t, J = 6.6 Hz, 2H), 2.75 (t, J = 6.6 Hz, 2H), 1.26 (t, J = 7.1 Hz, 3H). **^{13}C{^{1}H} NMR** (101 MHz, $CDCl_3$) δ (ppm): 198.3, 173.0, 136.7, 133.3, 128.7, 128.2, 60.8, 33.5, 28.4, 14.3. The NMR data was in accordance with the literature.[14]

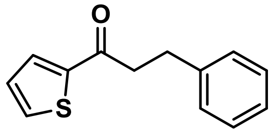

3-phenyl-1-(thiophen-2-yl)propan-1-one (6o): Conditions, 0.1 mmol scale with 1 bar H_2. Isolated as a colorless oil by column chromatography (SiO_2; ethyl acetate:hexane, 2:98 (v/v)). Yield: 17.5 mg (81%). **^{1}H NMR** (400 MHz, $CDCl_3$) δ (ppm): 7.71 – 7.66 (m, 1H), 7.65 – 7.59 (m, 1H), 7.34 – 7.27 (m, 2H), 7.25 (d, J = 7.0 Hz, 2H), 7.21 (t, J = 7.1 Hz, 1H), 7.11 (dd, J = 4.7, 4.0 Hz, 1H), 3.27 – 3.20 (m, 2H), 3.07 (t, J = 7.7 Hz, 2H). **^{13}C{^{1}H} NMR** (101 MHz, $CDCl_3$) δ (ppm): 192.3, 144.2, 141.1, 133.7, 132.0, 128.7, 128.5, 128.2, 126.3, 41.3, 30.5. The NMR data was in accordance with the literature.[7]

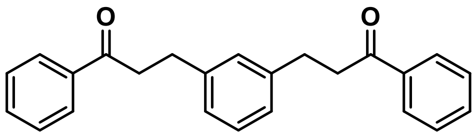

3,3'-(1,3-phenylene)bis(1-phenylpropan-1-one) (6p): Conditions, 0.05 mmol scale with 1 bar H_2. Isolated as a colorless oil by column chromatography (SiO_2; ethyl acetate:hexane, 3:97 (v/v)). Yield: 13.9 mg (81%). **^{1}H NMR** (400 MHz, $CDCl_3$) δ (ppm): 7.96 (d, *J* = 7.3 Hz, 4H), 7.56 (t, *J* = 7.4 Hz, 2H), 7.46 (t, *J* = 7.6 Hz, 4H), 7.28 – 7.22 (m, 1H), 7.15 (s, 1H), 7.12 (d, *J* = 7.6 Hz, 2H), 3.34 – 3.25 (m, 4H), 3.06 (t, *J* = 7.7 Hz, 4H). **^{13}C{^{1}H} NMR** (101 MHz, $CDCl_3$) δ (ppm): 199.4, 141.7, 137.0, 133.2, 128.9, 128.7, 128.2, 126.4, 40.6, 30.2. The NMR data was in accordance with the literature.[15]

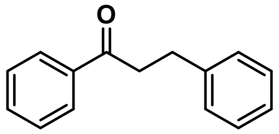

1,3-diphenylpropan-1-one (6q): Conditions, 0.1 mmol scale with 2 bar H_2. Isolated as a white amorphous solid by column chromatography (SiO_2; ethyl acetate:hexane, 1:99 (v/v)). Yield: 17.1 mg (81%). **^{1}H NMR** (400 MHz, $CDCl_3$) δ (ppm): 7.96 – 7.94 (m, 2H), 7.55 (t, *J* = 7.4 Hz, 1H), 7.44 (t, *J* = 7.6 Hz, 2H), 7.32 – 7.23 (m, 4H), 7.20 (t, *J* = 7.0 Hz , 1H), 3.32 – 3.28 (m, 2H), 3.08 – 3.05 (m, 2H).**^{13}C{^{1}H} NMR** (101 MHz, $CDCl_3$) δ (ppm): 199.3, 141.4, 136.9, 133.2, 128.7, 128.6, 128.5, 128.1, 126.2, 40.6, 30.2. The NMR data were in accordance with the literature.[6]

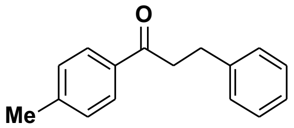

3-phenyl-1-(p-tolyl)propan-1-one (6r): Conditions, 0.1 mmol scale with 2 bar H_2. Isolated as a colorless oil by column chromatography (SiO_2; ethyl acetate:hexane, 1:99 (v/v)). Yield: 17.9 mg (80%). **^{1}H NMR** (400 MHz, $CDCl_3$) δ (ppm): 7.86 (d, *J* = 8.2 Hz, 2H), 7.32 – 7.28 (m, 2H), 7.26 -7.19 (m, 5H), 3.29 – 3.25 (m, 2H), 3.08 – 3.04 (m, 2H), 2.40 (s, 3H). **^{13}C{^{1}H} NMR** (101 MHz, $CDCl_3$) δ (ppm): 199.0, 143.9, 141.5, 134.4, 129.4, 128.6, 128.5, 128.3, 126.2, 40.5, 30.3, 21.7. The NMR data was in accordance with the literature.[6]

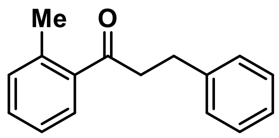

3-phenyl-1-(o-tolyl)propan-1-one (6s): Conditions, 0.1 mmol scale with 2 bar H_2. Isolated as a pale yellow oil by column chromatography (SiO_2; ethyl acetate:hexane, 1:99 (v/v)). Yield: 20.6 mg (92%). **^{1}H NMR** (400 MHz, $CDCl_3$) δ (ppm): 7.59 (d, *J* = 7.5 Hz, 1H), 7.34 (t, *J* = 7.4 Hz, 1H), 7.28 (t, *J* = 7.4 Hz, 2H), 7.25 – 7.15 (m, 5H), 3.21 (t, *J* = 7.6 Hz, 2H), 3.03 (t, *J* = 7.6 Hz, 2H), 2.46 (s, 3H). **^{13}C{^{1}H} NMR** (101 MHz, $CDCl_3$) δ (ppm): 203.5, 141.3, 138.2, 138.0, 132.1, 131.4, 128.6, 128.5, 128.5, 126.2, 125.8, 43.3, 30.4, 21.4. The NMR data was in accordance with the literature.[6]

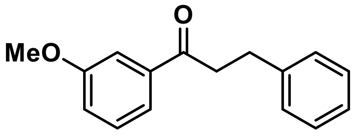

1-(3-methoxyphenyl)-3-phenylpropan-1-one (6t): Conditions, 0.1 mmol scale with 2 bar H_2. Isolated as a colorless oil by column chromatography (SiO_2; ethyl acetate:hexane, 2:98 (v/v)). Yield: 21.3 mg (89%). **^{1}H NMR** (400 MHz, $CDCl_3$) δ (ppm): 7.53 (d, *J* = 7.7 Hz, 1H), 7.48 (s, 1H), 7.35 (t, *J* = 7.9 Hz, 1H), 7.32 – 7.27 (m, 2H), 7.25 (d, *J* = 6.9 Hz, 2H), 7.21 (t, *J* = 7.1 Hz, 1H), 7.10 (dd, *J* = 8.2, 2.1 Hz, 1H), 3.84 (s, 3H), 3.29 (t, *J* = 7.7 Hz, 2H), 3.06 (t, *J* = 7.7 Hz, 2H). **^{13}C{^{1}H} NMR** (101 MHz, $CDCl_3$) δ (ppm):199.2, 160.0, 141.4, 138.3, 129.7, 128.7, 128.6, 126.3, 120.8, 119.7, 112.4, 55.6, 40.7, 30.3. The NMR data was in accordance with the literature.[16]

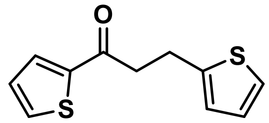

1,3-di(thiophen-2-yl)propan-1-one (6u): Conditions, 0.1 mmol scale with 2 bar H_2. Isolated as a colorless oil by column chromatography (SiO_2; ethyl acetate:hexane, 2:98 (v/v)). Yield: 21.0 mg (94%). **^{1}H NMR** (400 MHz, $CDCl_3$) δ (ppm): 7.72 (d, *J* = 3.8 Hz, 1H), 7.64 (d, *J* = 4.9 Hz, 1H), 7.13 (t, *J* = 4.2 Hz, 2H), 6.92 (dd, *J* = 4.9, 3.7 Hz, 1H), 6.86 (d, *J* = 3.2 Hz, 1H), 3.30 (s, 4H). **^{13}C{^{1}H} NMR** (101 MHz, $CDCl_3$) δ (ppm): 191.6, 144.1, 143.6, 133.8, 132.0, 128.2, 127.0, 124.9, 123.6, 41.3, 24.5. The NMR data was in accordance with the literature.[10]

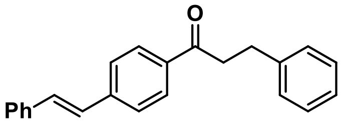

(E)-3-phenyl-1-(4-styrylphenyl)propan-1-one (6v): Conditions, 0.1 mmol scale with 2 bar H_2. Isolated as a white amorphous solid by column chromatography (SiO_2; dichloromethane). Yield: 27.2 mg (87%). **^{1}H NMR** (400 MHz, $CDCl_3$) δ (ppm): 7.96 (d, J = 8.3 Hz, 2H), 7.58 (d, J = 8.3 Hz, 2H), 7.54 (d, J = 7.5 Hz, 2H), 7.39 (t, J = 7.5 Hz, 2H), 7.35 – 7.18 (m, 7H), 7.13 (d, J = 16.3 Hz, 1H), 3.35 – 3.27 (m, 2H), 3.09 (t, J = 7.7 Hz, 2H). **^{13}C{^{1}H} NMR** (101 MHz, $CDCl_3$) δ (ppm): 198.7, 142.1, 141.5, 136.8, 135.8, 131.6, 128.9, 128.7, 128.7, 128.6, 128.5, 127.6, 126.9, 126.7, 126.3, 40.6, 30.3. The NMR data was in accordance with the literature.[7]

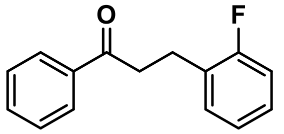

3-(2-fluorophenyl)-1-phenylpropan-1-one (6w): Conditions, 0.1 mmol scale with 2 bar H_2. Isolated as a pale yellow oil by column chromatography (SiO_2; ethyl acetate:hexane, 1:99 (v/v)). Yield: 18.0 mg (79%). **^{1}H NMR** (400 MHz, $CDCl_3$) δ (ppm): 7.97 (d, J = 7.3 Hz, 2H), 7.56 (t, J = 7.4 Hz, 1H), 7.46 (t, J = 7.6 Hz, 2H), 7.28 (t, J = 6.7 Hz, 1H), 7.20 (td, J = 7.4, 1.4 Hz, 1H), 7.11 – 6.98 (m, 2H), 3.31 (t, J = 7.6 Hz, 2H), 3.10 (t, J = 7.6 Hz, 2H). **^{13}C{^{1}H} NMR** (101 MHz, $CDCl_3$) δ (ppm): 199.1, 161.4 (d, J_{C-F} = 244.8 Hz), 136.9, 133.2, 131.1 (d, J_{C-F} = 5.0 Hz), 128.7, 128.2, 128.2 (d, J_{C-F} = 15.7 Hz), 128.1 (d, J_{C-F} = 8.1 Hz), 124.2 (d, J_{C-F} = 3.5 Hz), 115.4 (d, J_{C-F} = 21.9 Hz), 39.0 (d, J_{C-F} = 1.1 Hz), 24.1 (d, J_{C-F} = 2.5 Hz). **^{19}F NMR** (377 MHz, $CDCl_3$) δ (ppm): -118.41. The NMR data was in accordance with the literature.[17]

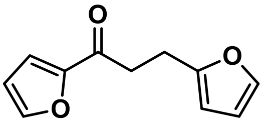

1,3-di(furan-2-yl)propan-1-one (6x): Conditions, 0.1 mmol scale with 2 bar H_2. Isolated as a colorless oil by column chromatography (SiO_2; ethyl acetate:hexane, 2:98 (v/v)). Yield: 17.7 mg (92%). **^{1}H NMR** (400 MHz, $CDCl_3$) δ (ppm): 7.58 (d, J = 0.8 Hz, 1H), 7.30 (d, J = 0.9 Hz, 1H), 7.19 (d, J = 3.5 Hz, 1H), 6.53 (dd, J = 3.5, 1.6 Hz, 1H), 6.29 – 6.24 (m, 1H), 6.03 (d, J = 3.0 Hz, 1H), 3.21 – 3.15 (m, 2H), 3.09 – 3.03 (m, 2H). **^{13}C{^{1}H} NMR** (101 MHz, $CDCl_3$) δ (ppm): 188.0, 154.5, 152.6, 146.5, 141.3, 117.2, 112.4, 110.3, 105.5, 36.8, 22.4. The NMR data was in accordance with the literature.[18]

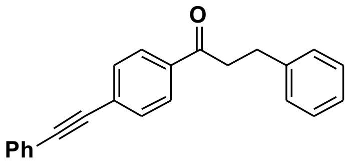

3-phenyl-1-(4-(phenylethyl)phenyl)propan-1-one (6y): Conditions, 0.1 mmol scale with 2 bar H_2. Isolated as a white amorphous solid by column chromatography (SiO_2; ethyl acetate:hexane, 1:99 (v/v)). Yield: 24.8 mg (80%). **^{1}H NMR** (400 MHz, $CDCl_3$) δ (ppm): 7.94 (d, *J* = 8.4 Hz, 2H), 7.60 (d, *J* = 8.4 Hz, 2H), 7.58 – 7.52 (m, 2H), 7.41 – 7.35 (m, 3H), 7.34 – 7.29 (m, 2H), 7.28 – 7.25 (m, 2H), 7.22 (t, *J* = 7.1 Hz, 1H), 3.31 (t, *J* = 7.7 Hz, 2H), 3.08 (t, *J* = 7.7 Hz, 2H). **^{13}C{^{1}H} NMR** (101 MHz, $CDCl_3$) δ (ppm): 198.6, 141.3, 136.0, 131.9, 131.9, 129.0, 128.7, 128.6, 128.6, 128.3, 128.1, 126.3, 122.8, 92.8, 88.7, 40.6, 30.2. The NMR data was in accordance with the literature.[7]

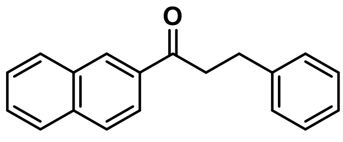

1-(naphthalen-2-yl)-3-phenylpropan-1-one (6z): Conditions, 0.1 mmol scale with 2 bar H_2. Isolated as a white amorphous solid by column chromatography (SiO_2; ethyl acetate:hexane, 1:99 (v/v)). Yield: 22.9 mg (88%). **^{1}H NMR** (400 MHz, $CDCl_3$) δ (ppm): 8.45 (s, 1H), 8.03 (d, *J* = 8.6 Hz, 1H), 7.93 (d, *J* = 8.0 Hz, 1H), 7.90 – 7.84 (m, 2H), 7.59 (t, *J* = 7.4 Hz, 1H), 7.54 (t, *J* = 7.4 Hz, 1H), 7.35 – 7.26 (m, 4H), 7.25 – 7.19 (m, 1H), 3.48 – 3.39 (m, 2H), 3.13 (t, *J* = 7.7 Hz, 2H).. **^{13}C{^{1}H} NMR** (101 MHz, $CDCl_3$) δ (ppm): 199.3, 141.5, 135.7, 134.3, 132.6, 129.8, 129.7, 128.7, 128.6 (3 x C), 127.9, 126.9, 126.3, 123.9, 40.7, 30.4. The NMR data was in accordance with the literature.[6]

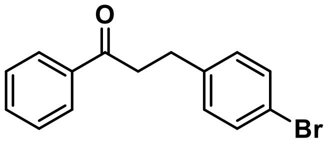

3-(4-bromophenyl)-1-phenylpropan-1-one (6aa): Conditions, 0.1 mmol scale with 2 bar H_2. Isolated as a white amorphous solid by column chromatography (SiO_2; ethyl acetate:hexane, 1:99 (v/v)). Yield: 22.5 mg (78%). **^{1}H NMR** (400 MHz, $CDCl_3$) δ (ppm): 7.95 (d, *J* = 7.3 Hz, 2H), 7.59 – 7.54 (m, 1H), 7.46 (t, *J* = 7.7 Hz, 2H), 7.41 (d, *J* = 8.3 Hz, 2H), 7.13 (d, *J* = 8.3 Hz, 2H), 3.28 (t, *J* = 7.5 Hz, 2H), 3.03 (t, *J* = 7.5 Hz, 2H).**^{13}C{^{1}H} NMR** (101 MHz, $CDCl_3$) δ (ppm): 199.0, 140.4, 136.9, 133.3, 131.7, 130.4, 128.8, 128.1, 120.0, 40.2, 29.6. The NMR data was in accordance with the literature.[19]

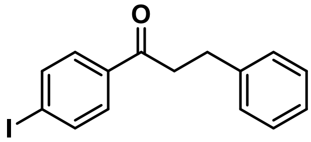

1-(4-iodophenyl)-3-phenylpropan-1-one (6ab): Conditions, 0.1 mmol scale with 3 bar H_2. Isolated as a white amorphous solid by column chromatography (SiO_2; ethyl acetate:hexane, 1:99 (v/v)). Yield: 21.5 mg (64%). **^{1}H NMR** (400 MHz, $CDCl_3$) δ (ppm): 7.84 – 7.79 (m, 2H), 7.68 – 7.64 (m, 2H), 7.33 – 7.28 (m, 2H), 7.26 – 7.19 (m, 3H), 3.29 – 3.22 (m, 2H), 3.09 – 3.02 (m, 2H)..**^{13}C{^{1}H} NMR** (101 MHz, $CDCl_3$) δ (ppm): 198.6, 141.2, 138.1, 136.2, 129.6, 128.7, 128.5, 126.4, 101.2, 40.5, 30.1. The NMR data was in accordance with the literature[6].

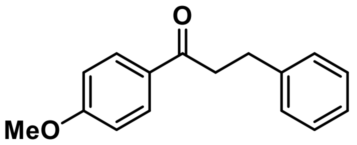

1-(4-methoxyphenyl)-3-phenylpropan-1-one (6ac): Conditions, 0.1 mmol scale with 3 bar H_2. Isolated as a white amorphous solid by column chromatography (SiO_2; ethyl acetate:hexane, 2:98 (v/v)). Yield: 20.4 mg (85%). **^{1}H NMR** (400 MHz, $CDCl_3$) δ (ppm): 7.98 – 7.92 (m, 2H), 7.34 – 7.24 (m, 4H), 7.24 – 7.18 (m, 1H), 6.96 – 6.89 (m, 2H), 3.87 (s, 3H), 3.30 – 3.22 (m, 2H), 3.10 – 3.02 (m, 2H).**^{13}C{^{1}H} NMR** (101 MHz, $CDCl_3$) δ (ppm): 198.0, 163.6, 141.6, 130.4, 130.1, 128.6, 128.6, 126.2, 113.8, 55.6, 40.2, 30.5. The NMR data was in accordance with the literature[6].

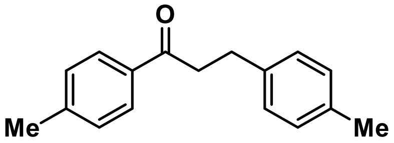

1,3-di-p-tolylpropan-1-one (6ad): Conditions, 0.1 mmol scale with 3 bar H_2. Isolated as a white amorphous solid by column chromatography (SiO_2; ethyl acetate:hexane, 1:99 (v/v)). Yield: 20.8 mg (87%). **^{1}H NMR** (400 MHz, $CDCl_3$) δ (ppm): 7.87 (d, J = 8.1 Hz, 2H), 7.25 (d, J = 8.0 Hz, 2H), 7.15 (d, J = 8.0 Hz, 2H), 7.11 (d, J = 8.0 Hz, 2H), 3.30 – 3.21 (m, 2H), 3.06 – 2.98 (m, 2H), 2.41 (s, 3H), 2.33 (s, 3H). **^{13}C{^{1}H} NMR** (101 MHz, $CDCl_3$) δ (ppm): 199.1, 143.9, 138.4, 135.7, 134.5, 129.4, 129.3, 128.4, 128.3, 40.6, 29.9, 21.7, 21.1. The NMR data was in accordance with the literature.[10]

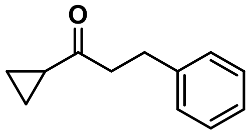

1-cyclopropyl-3-phenylpropan-1-one (6ae): Conditions, 0.2 mmol scale with 5 bar H_2. Isolated as a colorless oil by preparative thin layer chromatography (SiO_2; ethyl acetate:hexane, 15:85 (v/v)). Yield: 15.0 mg (43%). **^{1}H NMR** (400 MHz, $CDCl_3$) δ (ppm): 7.32 – 7.25 (m, 2H), 7.23 – 7.16 (m, 3H), 2.98

– 2.84 (m, 4H), 1.95 – 1.88 (m, 1H), 1.06 – 0.99 (m, 2H), 0.89 – 0.82 (m, 2H).**^{13}C{^{1}H} NMR** (101 MHz, $CDCl_3$) δ (ppm): 210.2, 141.4, 128.6, 128.5, 126.2, 45.1, 30.1, 20.7, 10.9. The NMR data was in accordance with the literature.[20]

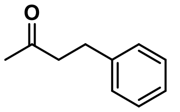

4-phenylbutan-2-one (6af): Conditions, 0.1 mmol scale with 5 bar H_2. Not isolated, NMR Yield (71%). **^{1}H NMR** (400 MHz, $CDCl_3$) δ (ppm): 7.30 – 7.23 (m, 2H), 7.18 (t, J = 6.9 Hz, 3H), 2.88 (t, J = 7.5 Hz, 2H), 2.74 (t, J = 7.5 Hz, 2H), 2.12 (s, 3H). **^{13}C{^{1}H} NMR** (101 MHz, $CDCl_3$) δ (ppm): 208.0, 141.1, 128.6, 128.4, 126.2, 45.2, 30.1, 29.8. The NMR data was in accordance with the literature.[7]

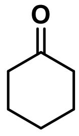

Cyclohexanone (6ag): Conditions, 0.2 mmol scale with 5 bar H_2. Not isolated, GC Yield (58%). GC calibration and calculation of yield have been discussed in last last section of the supporting information.

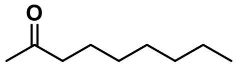

Nonan-2-one (6ah): Conditions, 0.2 mmol scale with 5 bar H_2. Isolated as a colorless liquid by column chromatography (SiO_2; ethyl acetate:hexane, 1:99 (v/v)). Yield: 12 mg (42%). **^{1}H NMR** (400 MHz, $CDCl_3$) δ (ppm): 2.41 (t, J = 7.5 Hz, 2H), 2.13 (s, 3H), 1.61 – 1.51 (m, 2H), 1.34 – 1.20 (m, 8H), 0.87 (t, J = 6.9 Hz, 3H).**^{13}C{^{1}H} NMR** (101 MHz, $CDCl_3$) δ (ppm): 209.6, 44.0, 31.8, 30.0, 29.3, 29.2, 24.0, 22.7, 14.2. The NMR data was in accordance with the literature.[21]

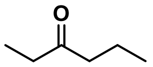

H**exan-3-one (6ai):** Conditions, 0.2 mmol scale with 5 bar H_2. Because of low boiling product (123 °C), only NMR yield was recorded with residual solvents (trace toluene-d_8 and dichloromethane) in the product mixture (with 0.33 equiv. of 1,3,5-Trimethoxybenzene as internal standard). The crude ^{1}H NMR data (in $CDCl_3$) (**Figure S138**) was in accordance with the literature.[22] From another duplicate run, Isolated product (SiO_2; dichloromethane) NMR was recorded in toluene-d_8. Isolated yield was not determined as it contains residual toluene-d_8 and excess dichloromethane. **^{1}H NMR** (400 MHz, Toluene-d_8) δ (ppm): 1.93 – 1.81 (m, 4H), 1.50 – 1.39 (m, 2H), 0.89 (t, J = 7.3 Hz, 3H), 0.76 (t, J = 7.4 Hz, 3H).**^{13}C{^{1}H} NMR** (101 MHz, Toluene-d_8) δ (ppm): 208.4, 44.0, 35.5, 17.5, 13.9, 7.9.

Gram scale hydrogenation of enone 5c:

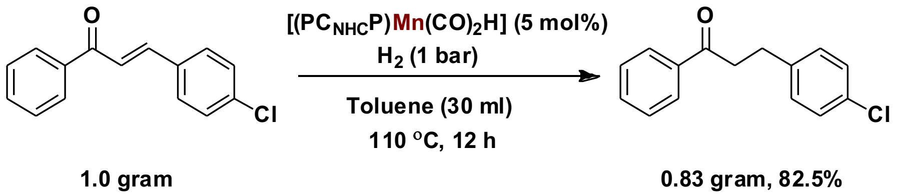

Scheme S3: Gram scale hydrogenation of (*E*)-3-(4-chlorophenyl)-1-phenylprop-2-en-1-one.

Inside a N_2-filled glovebox, to a solution of (*E*)-3-(4-chlorophenyl)-1-phenylprop-2-en-1-one (1g, 4.12 mmol) in toluene (30 mL) in flame-dried Schlenk tube with J. Young valve was added [(PC$_{NHC}$P)Mn(CO)$_2$H] (**1**; 129 mg, 0.206 mmol, 5 mol%). The flask was sealed and degassed by three freeze-pump-thaw cycles using Schlenk techniques and connected to an intermediate pressure hydrogen line. The line was purged by subsequent evacuation and filling with H_2 (3×). The tube was opened and was filled with hydrogen at a pressure of 1 bar. Hereafter, the flask was placed on a preheated oil bath at 110 °C for 12h. After 12h, the reaction mixture was cooled down to room temperature and filtered over a short alumina pad to separate the organic products from the manganese catalyst. The residual alumina was washed with dichloromethane (300 mL), and complete elution of the organic products was confirmed by TLC analysis. Next, the solvent was evaporated under reduced pressure and the crude reaction mixture was purified by flash chromatography (SiO_2; ethyl acetate:hexane, 5:95 (v/v)) to afford an off-white solid. **Isolated yield**: 830 mg (3.39 mmol, 82.5%). NMR data is identical to that of **6c**.

Mechanistic Studies

Hydrogenation catalysis with enone substrate 5c and D_2:

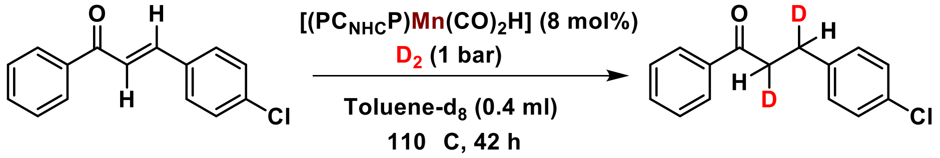

Scheme S4: Hydrogenation catalysis with enone substrate **5c** and D_2.

Inside a N_2-filled glovebox, a J. Young NMR tube suitable for intermediate pressure (up to 10.5 bar) was charged with of (*E*)-3-(4-chlorophenyl)-1-phenylprop-2-en-1-one (24.27 mg, 0.1 mmol), [(PC$_{NHC}$P)Mn(CO)$_2$H] (**1**, 5.1 mg, 0.008 mmol, 8 mol%), and toluene-d$_8$ (0.4 mL). The tube was sealed and was taken out of the glovebox. Hereafter, the tube was degassed by freeze-pump-thaw cycles (3×) using Schlenk techniques and connected to an intermediate pressure deuterium gas (D_2) line. The line was purged by subsequent evacuation and filling with D_2 (3×). The tube was opened and was filled with deuterium gas at the desired pressure (1 bar). Next, the tube was sealed again and placed on a preheated oil bath at 110 °C. The reaction progress was monitored by ^{1}H-NMR spectroscopy at room temperature by comparing the relative integration of the specific resonances associated with the reactant and product in the crude ^{1}H-NMR spectrum. After 96% conversion (**Figure S1**; 42 h) the reaction mixture was cooled to room temperature, and the solvent was evaporated under reduced pressure, whereafter the deuterium (^{2}H)-NMR was recorded using 600 µl toluene and 5 µl of toluene-d8 (**Figure S2**).

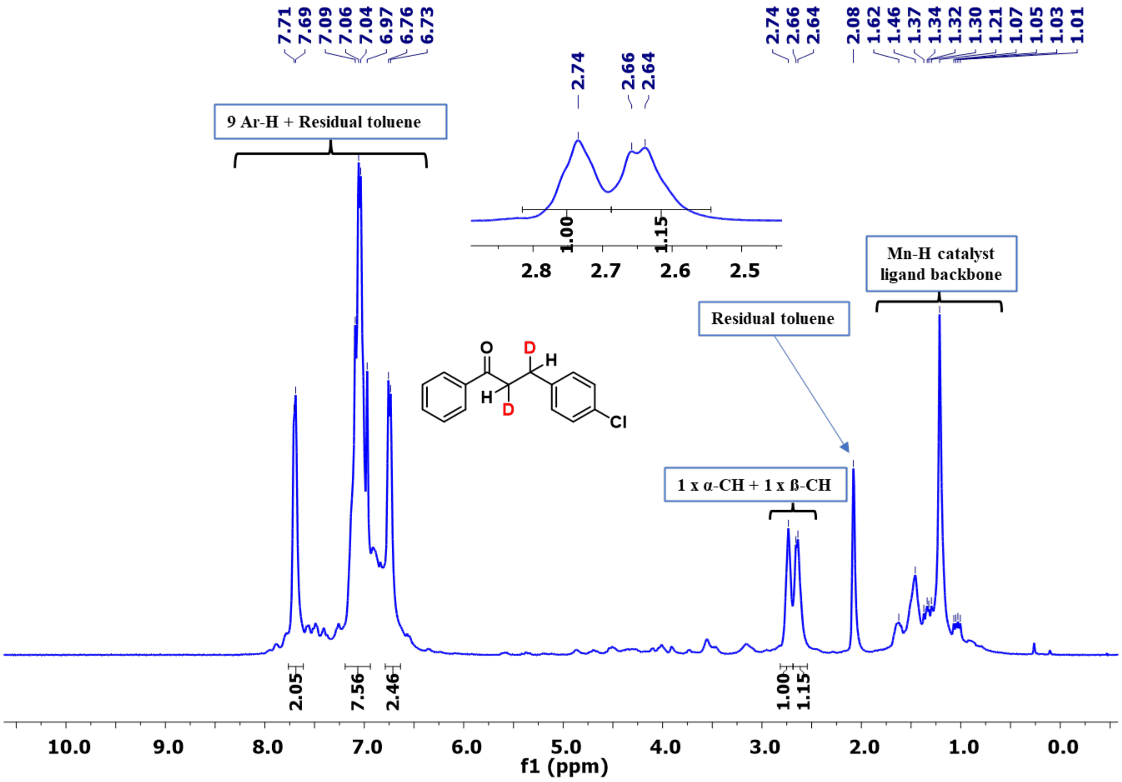

Figure S1: ^{1}H NMR spectrum (300 MHz) after 42 h of reaction at 110 °C in toluene-d8

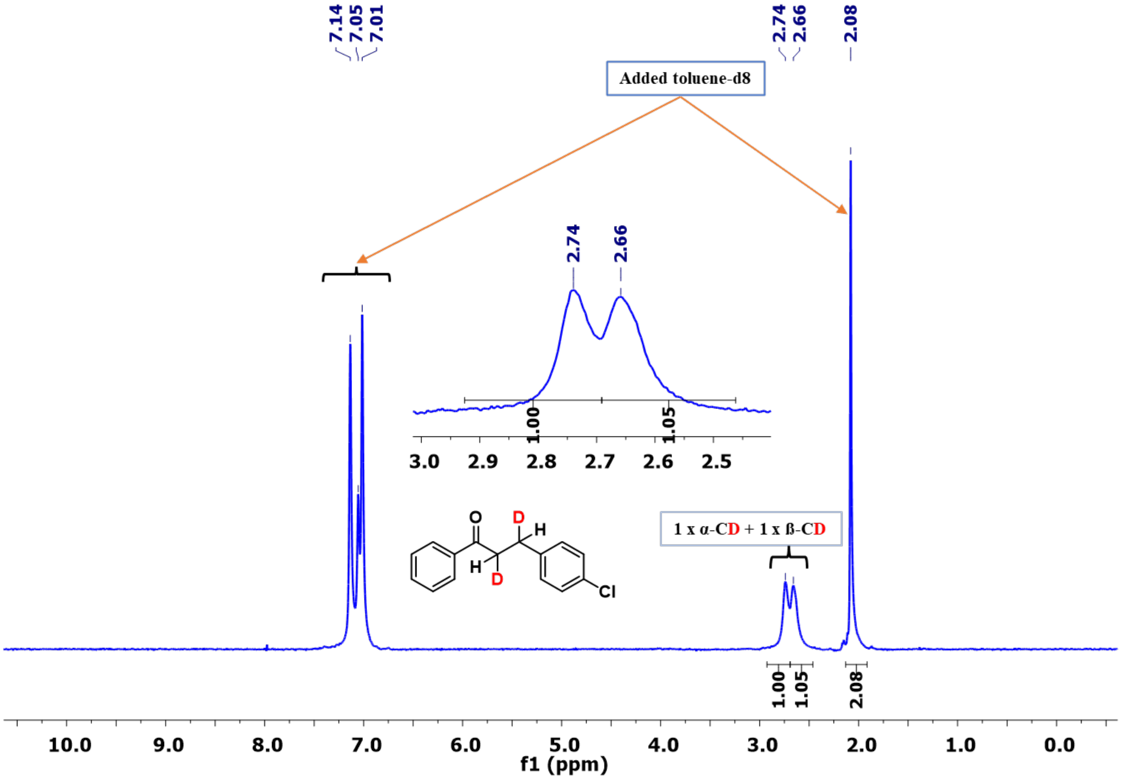

Figure S2: Deuterium (^{2}H) NMR spectrum (46 MHz, 600 µL toluene with 5 µL toluene-d_8 solvent) of the crude reaction mixture after catalysis with D_2

Reaction of [($PC_{NHC}P$)Mn(CO)$_2$H] and H_2 in absence of enone substrate:

Inside a N_2-filled glovebox, a J. Young NMR tube suitable for intermediate pressure (up to 10.5 bar) was charged with [($PC_{NHC}P$)Mn(CO)$_2$H] (**1**, 6.25 mg, 0.01 mmol, 8), and toluene-d_8 (0.4 mL). The tube was sealed and was taken out of the glovebox. Hereafter, the tube was degassed by freeze-pump-thaw cycles (3×) using Schlenk techniques and connected to an intermediate pressure hydrogen line. The line was purged by subsequent evacuation and filling with H_2 (3×). The tube was opened and was filled with hydrogen gas at the desired pressure (5 bar). Next, the tube was sealed and kept at room temperature for 30 minutes. Hereafter, the tube was heated at 110 °C for 24 h, followed by heating at 140 °C for 18 h. At every stage, the ^1H and ^{31}P NMR spectra were recorded to gauge if any new manganese hydride/dihydrogen species (**Figure S3** to **Figure S5**).

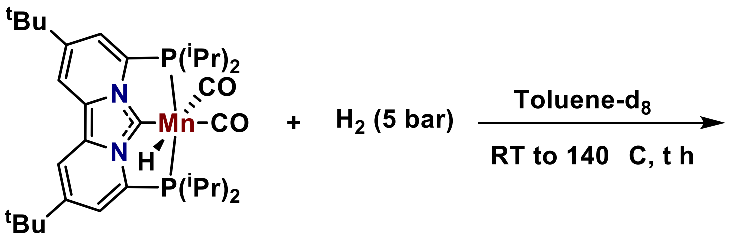

Scheme S5: Reaction of [(PCP)Mn(CO)$_2$H] and 5 bar H_2 in absence of enone substrate.

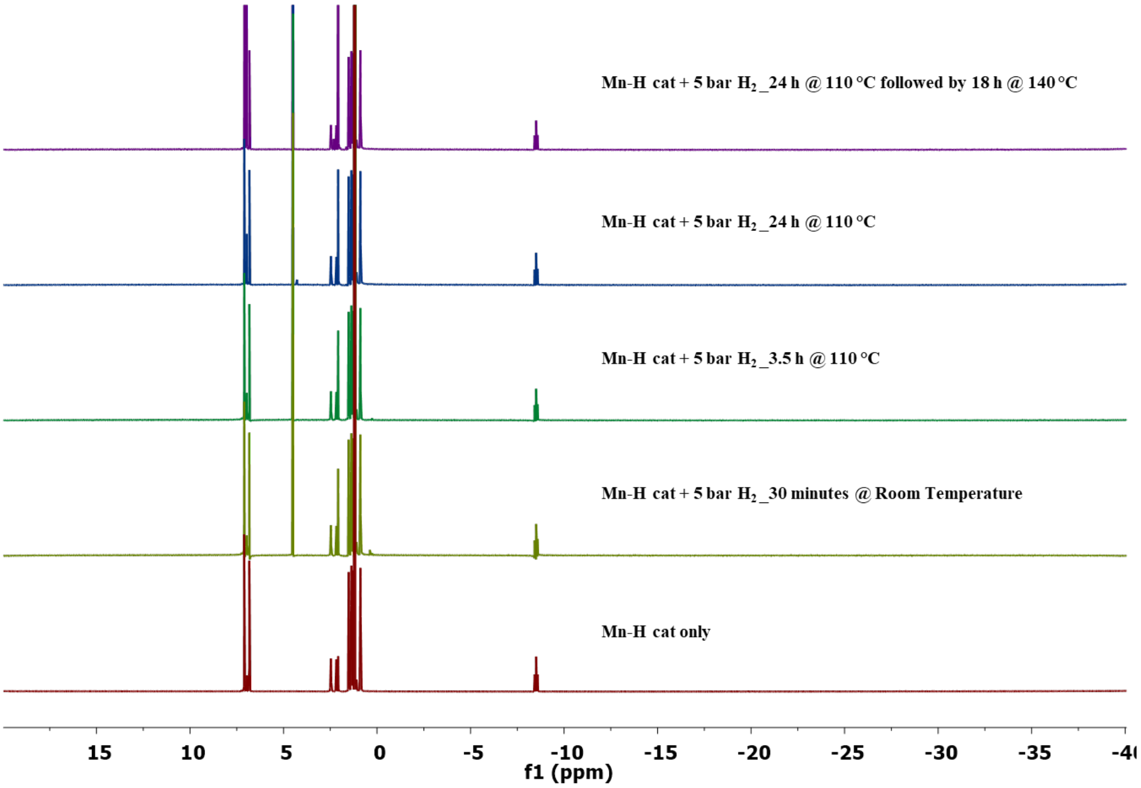

Figure S3: Stacked ^{1}H NMR (600 MHz) spectra (20 ppm to −40 ppm) of **1** in the presence of H_2, after heating at different temperatures in toluene-d_8

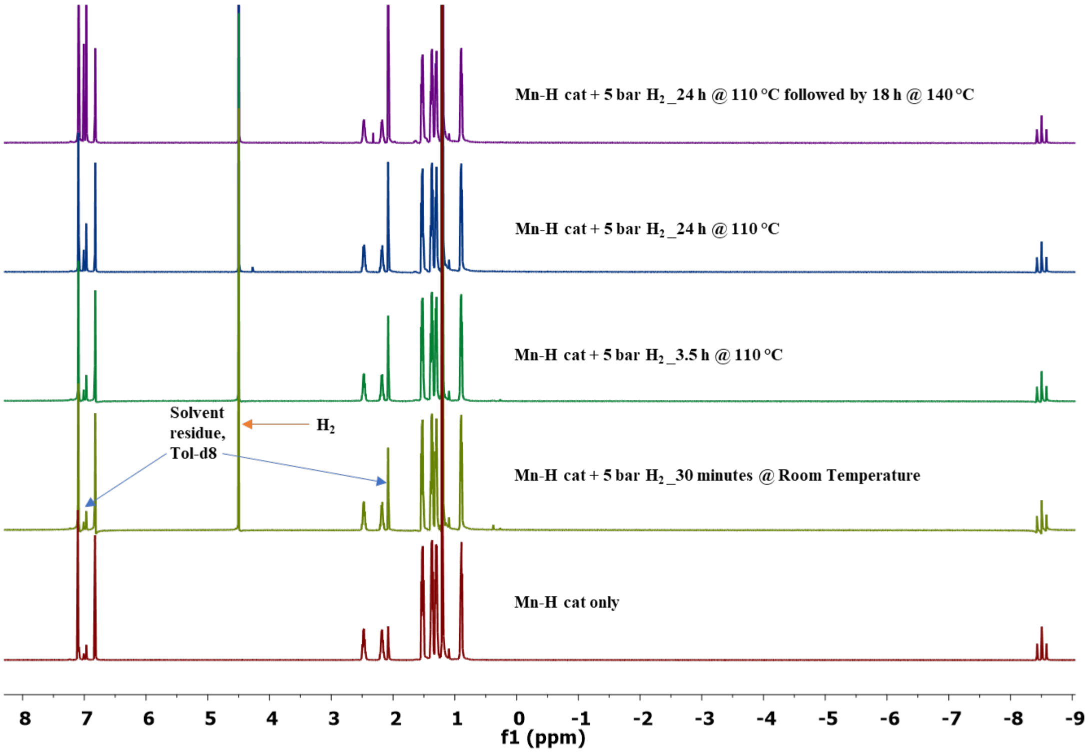

Figure S4: Stacked ^{1}H NMR (600 MHz) spectra (8 ppm to −9 ppm) of 1 in the presence of H_2, after heating at different temperatures in toluene-d_8

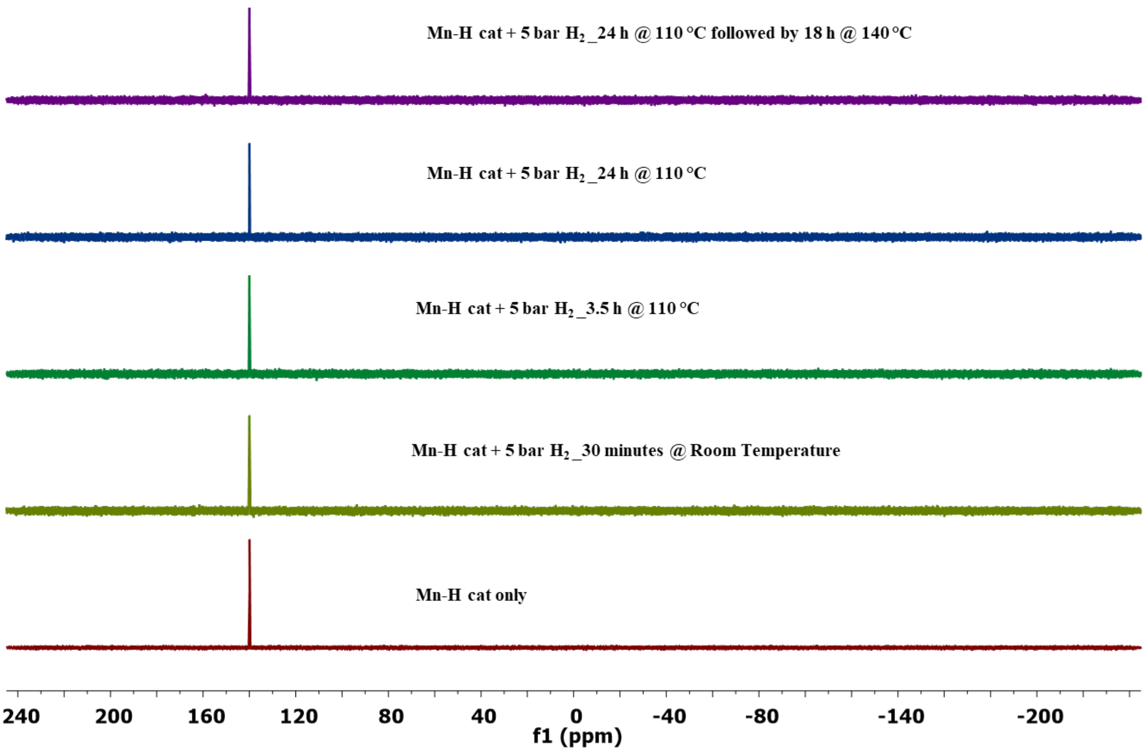

Figure S5: Stacked ^{31}P NMR (243 MHz) spectra (250 ppm to −250 ppm) of **1** in the presence of H_2, after heating at different temperatures in toluene-d_8.

Stoichiometric reaction of [(PC$_{NHC}$P)Mn(CO)$_2$H] and enone substrate 5h and exposure of the intermediate species to D_2 atmosphere:

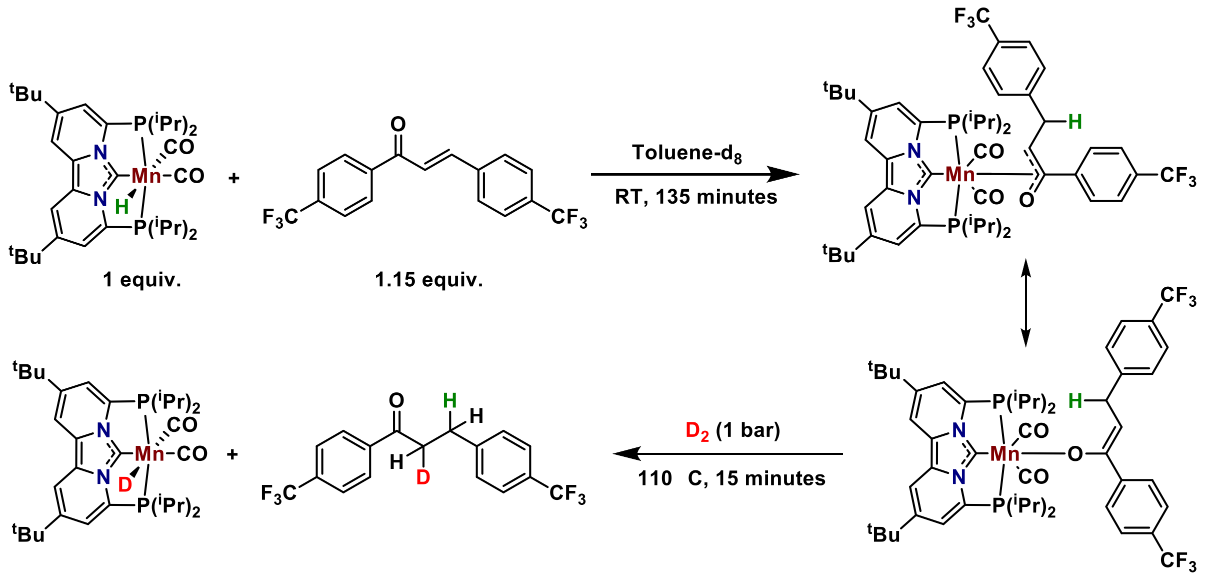

Scheme S6: Stoichiometric reaction of [(PC$_{NHC}$P)Mn(CO)$_2$H] with enone substrate **5h** and exposure of the intermediate species to D_2 atmosphere.

Inside a N_2-filled glovebox, a J. Young NMR tube suitable for intermediate pressure (up to 10.5 bar) was charged with [(PC$_{NHC}$P)Mn(CO)$_2$H] (**1**, 18.7 mg, 0.03 mmol), chalcone **5h** (11.9 mg, 0.0345 mmol, 1.15 equiv.), and toluene-d$_8$ (0.4 mL). The tube was sealed and was taken out of the glovebox. Hereafter, the tube was degassed by freeze-pump-thaw cycles (3×) using Schlenk techniques. For 135 minutes the tube was kept at room temperature and shaken every 10 minutes to ensure proper mixing of the contents. Analysis of the ^{1}H NMR spectrum after 135 minutes showed the formation of a new species (**Figure S7**) and ca. 80% consumption. Hereafter, the J. Young tube, was connected to an intermediate pressure hydrogen line. The line was purged by subsequent evacuation and filling with D_2 (3×). The tube was opened and was filled with deuterium gas at the desired pressure (1 bar). Hereafter, the tube was heated at 110 °C for 15 minutes, resulting in formation of the partially deuterated **6h** and [(PC$_{NHC}$P)Mn(CO)$_2$D] (**1-D**). The ^{1}H (**Figure S6** to **Figure S9**), ^{19}F (**Figure S14** to **Figure S15**) and ^{31}P (**Figure S11** to **Figure S13**) NMR spectra were recorded at different stages of the reaction and stacked for comparison. After completion of the reaction, the solvent was evaporated and the deuterium (^{2}H)-NMR was recorded in 600 µL toluene and 5 µL of toluene-d$_8$ (**Figure S10**).

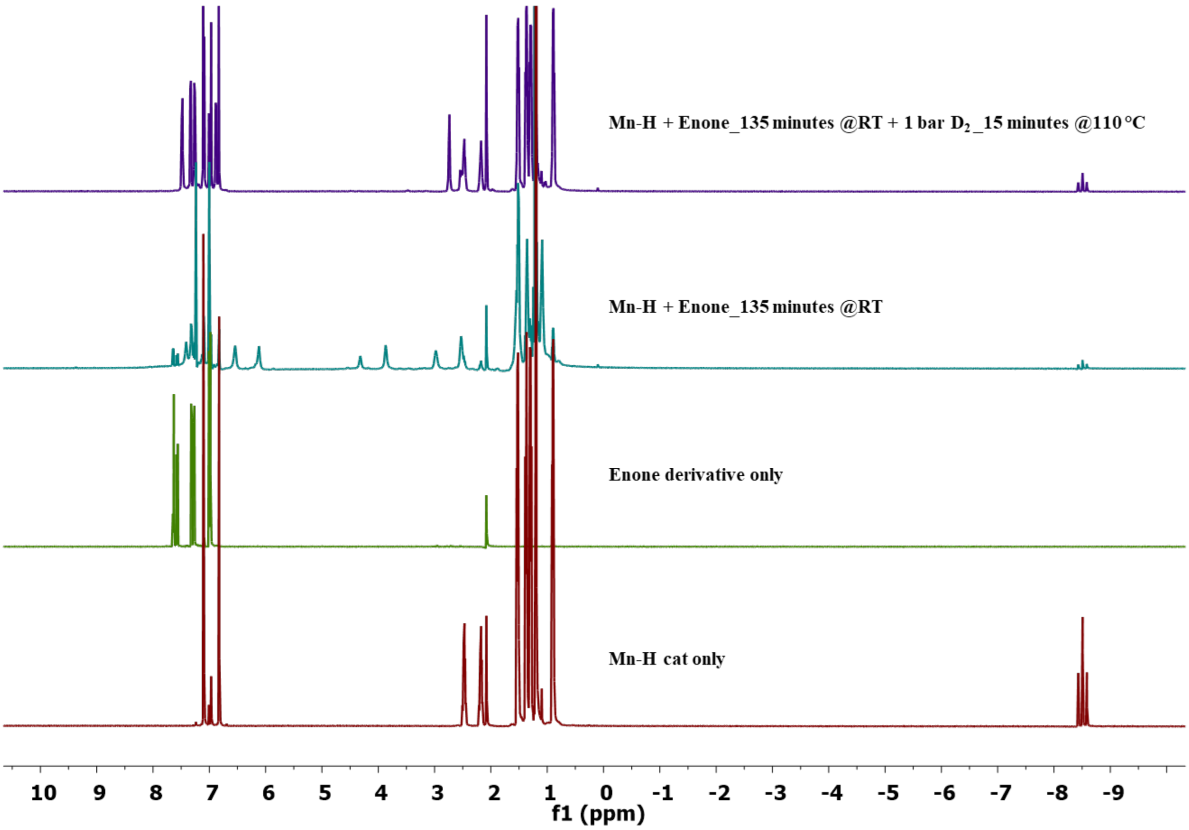

Figure S6: Stacked ^{1}H NMR (600 MHz) spectra (10 ppm to −10 ppm) at different stages during reaction recorded in toluene-d_8.

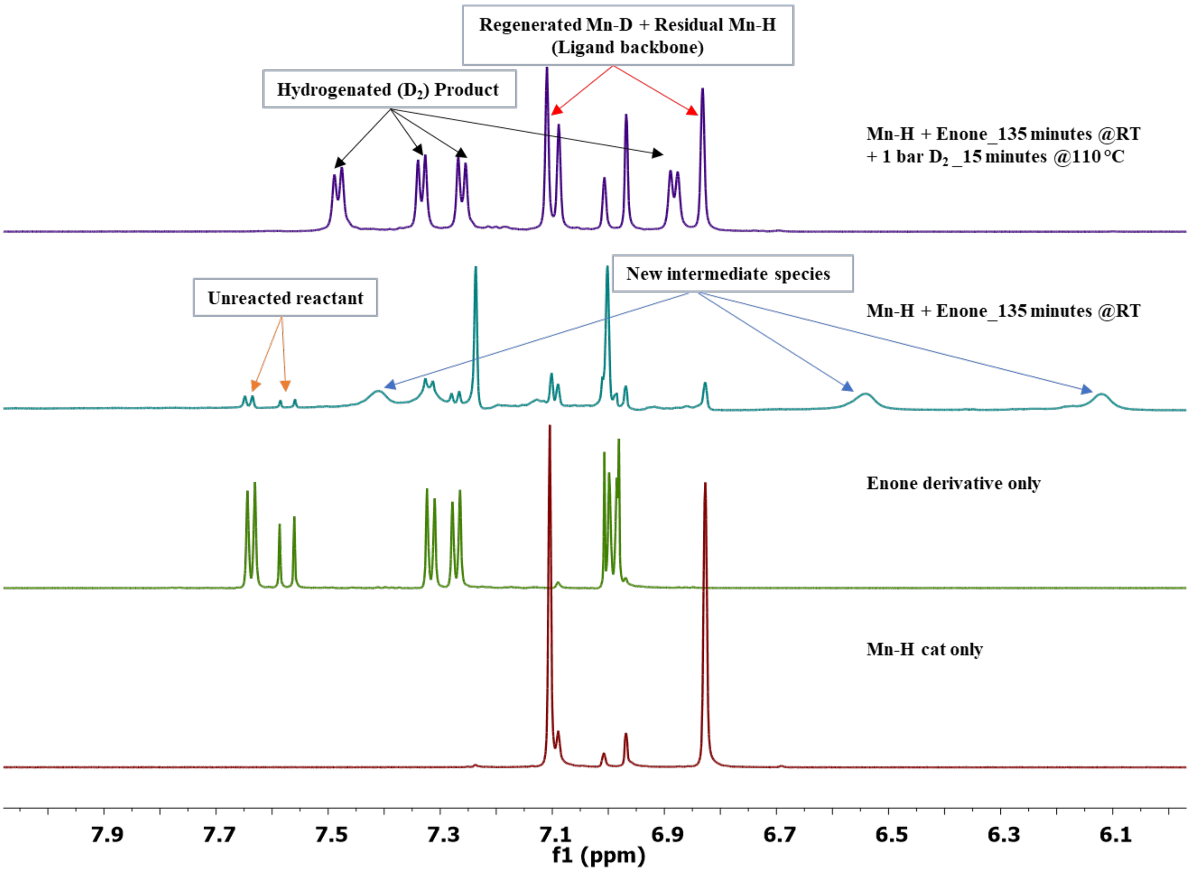

Figure S7: Stacked ^{1}H NMR (600 MHz) spectra (6 ppm to 8 ppm) at different stages during reaction recorded in toluene-d_8.

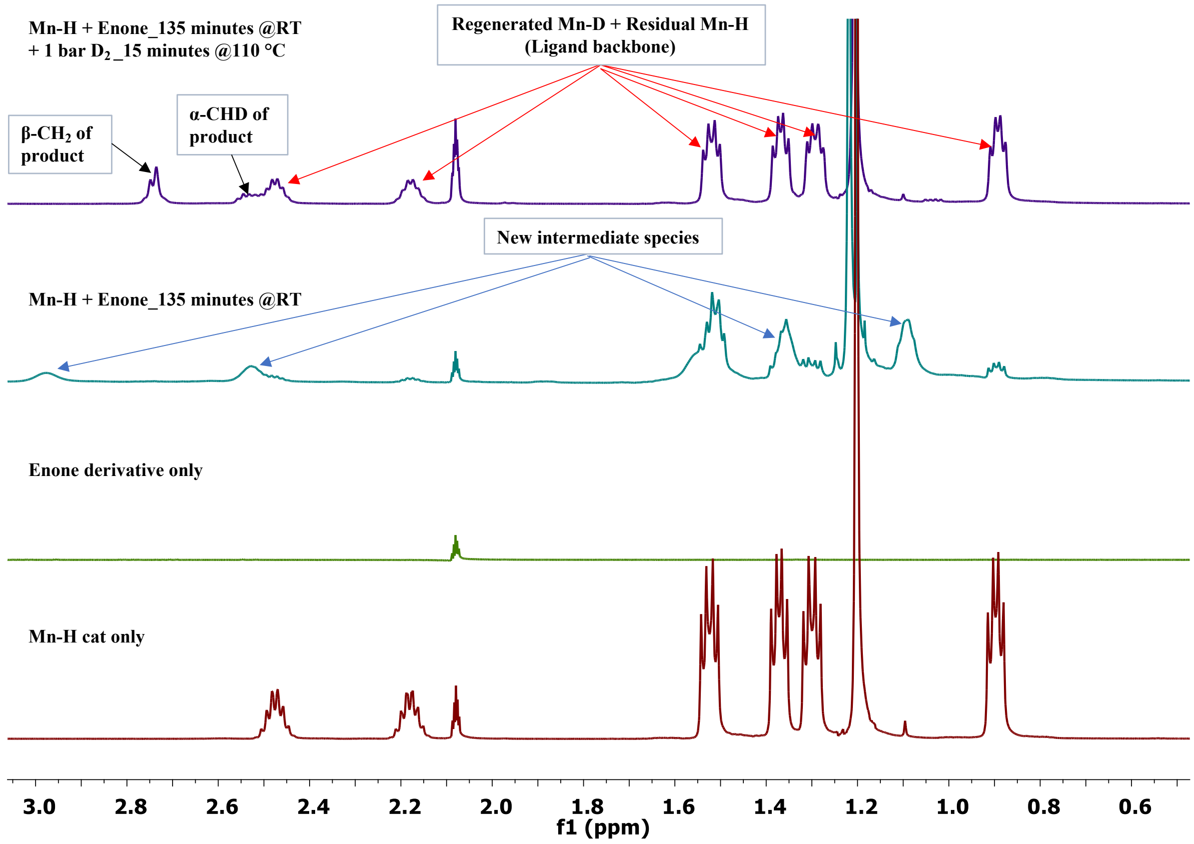

Figure S8: Stacked ^{1}H NMR (600 MHz) spectra (0.5 ppm to 3 ppm) at different stages during reaction recorded in toluene-d_8.

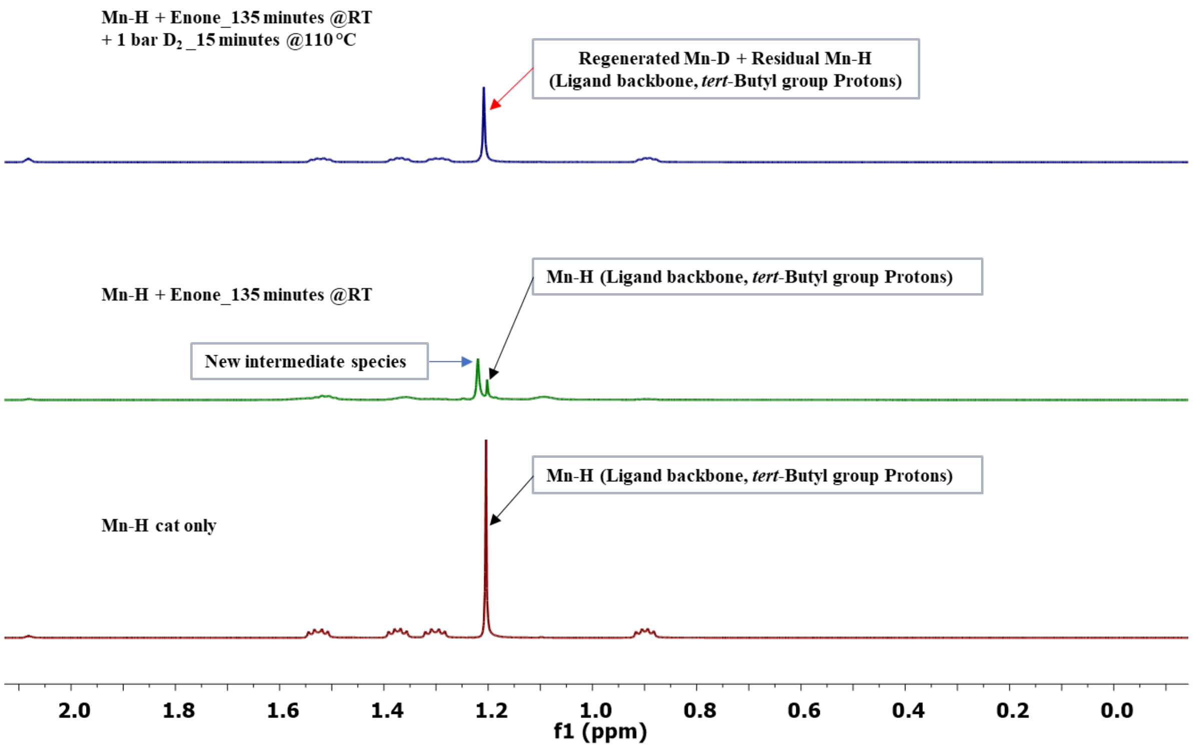

Figure S9: Stacked ^{1}H NMR (600 MHz) spectra (−0.2 ppm to 2.2 ppm) at different stages during reaction recorded in toluene-d_8.

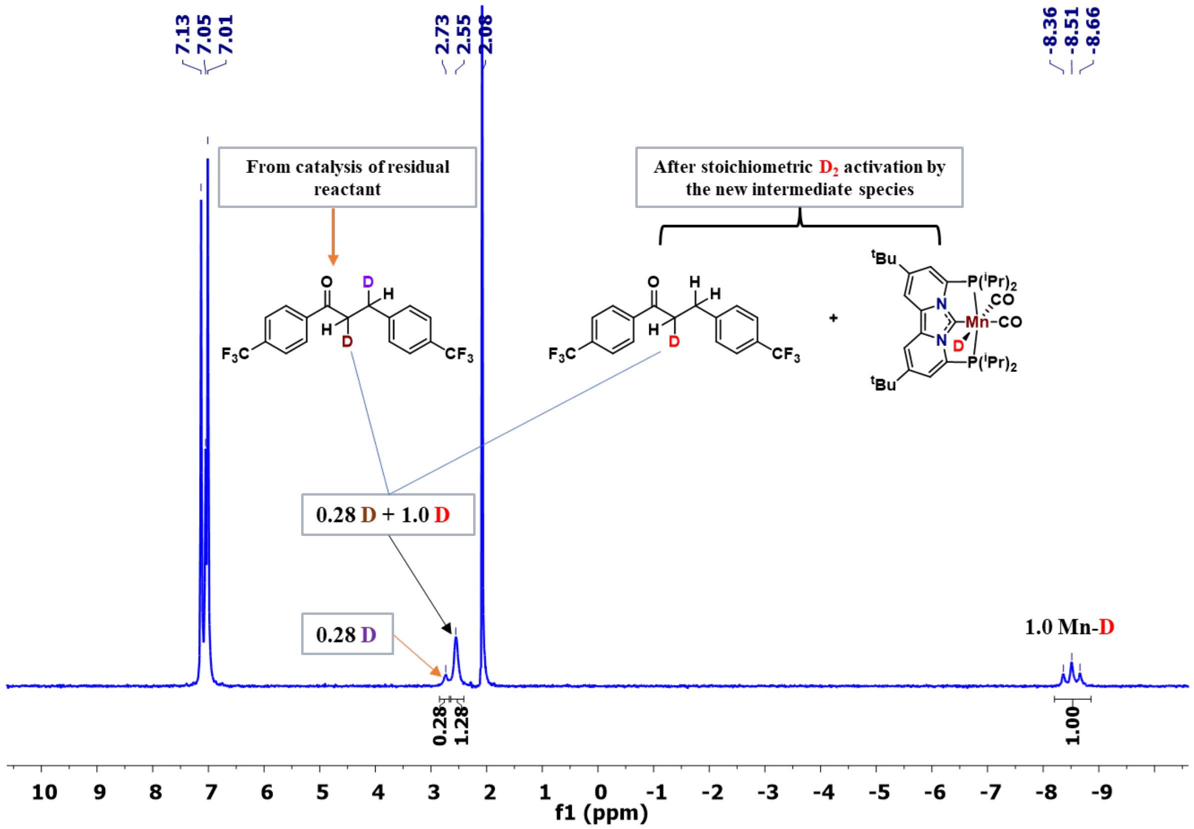

Figure S10: Deuterium (^{2}H) NMR spectrum (46 MHz, of the crude reaction mixture containing partially deuterated **6h**.

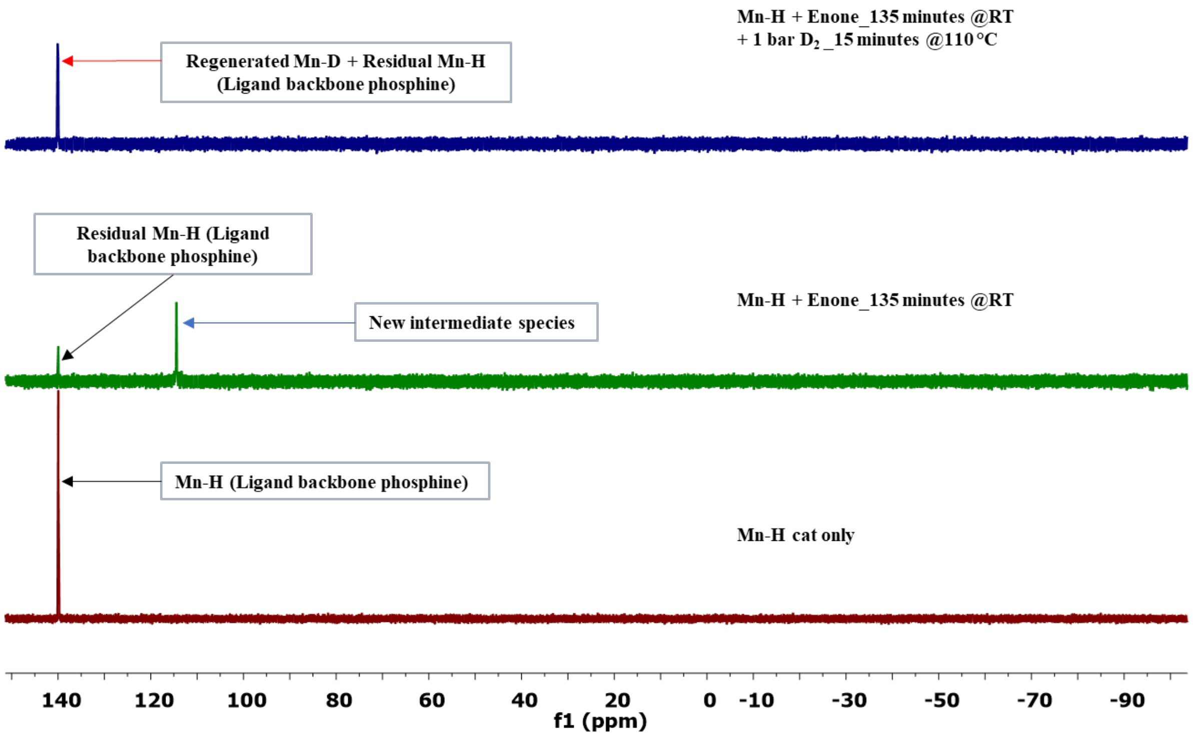

Figure S11: Stacked ^{31}P NMR (243 MHz) spectra (−100 ppm to 150 ppm) at different stages during reaction recorded in toluene-d_8.

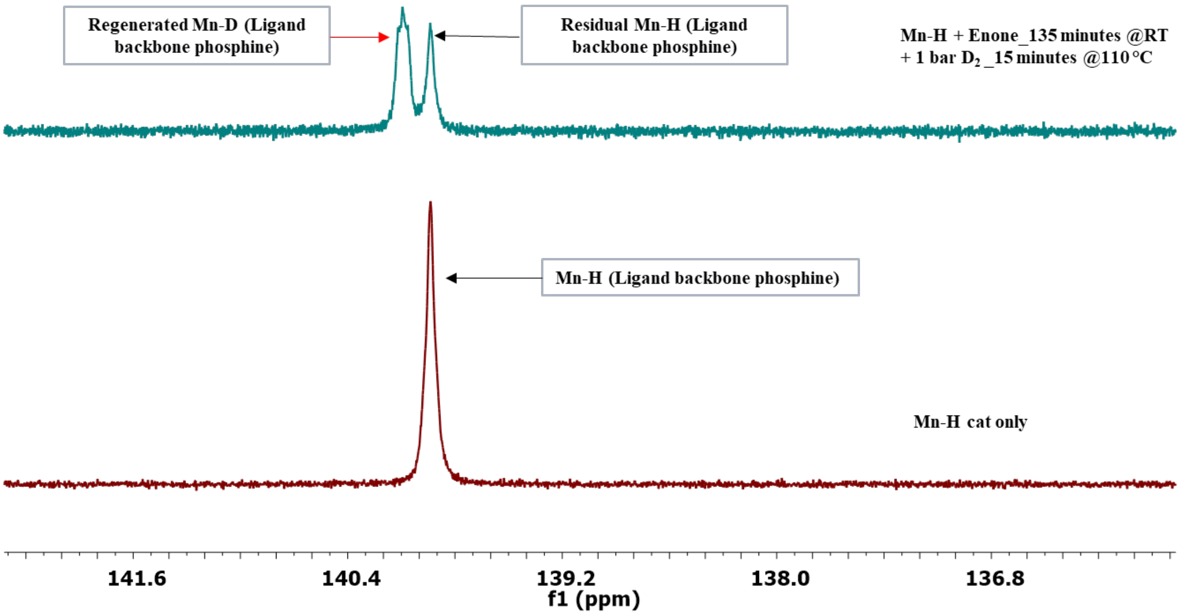

Figure S12: Stacked ^{31}P NMR (243 MHz) spectra (136 ppm to 142 ppm) at different stages during reaction recorded in toluene-d_8.

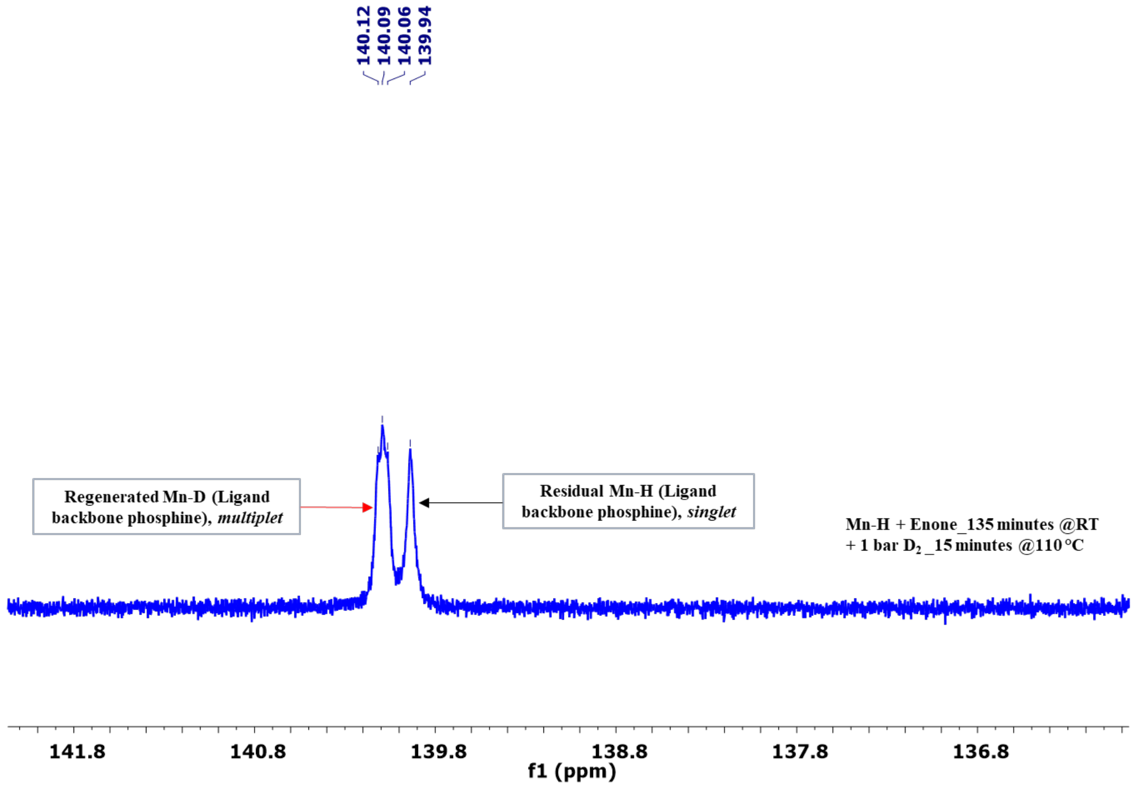

Figure S13: ^{31}P NMR (243 MHz) spectra (136 ppm to 142 ppm) of **1-D** and **1** obtained after the reaction in toluene-d$_8$.

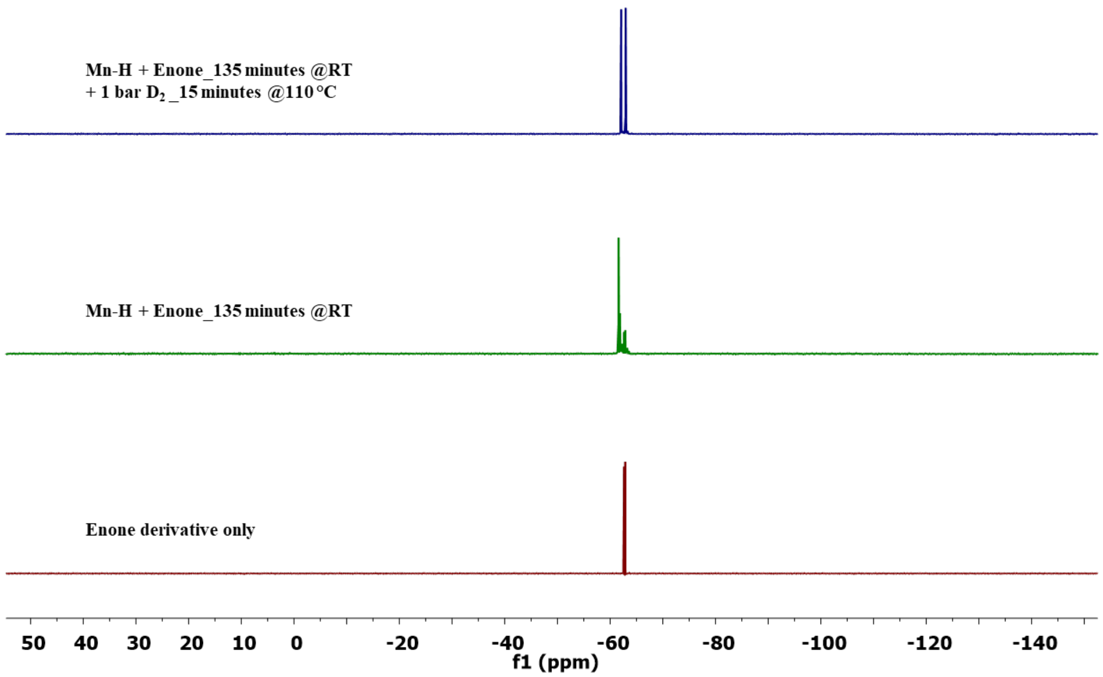

Figure S14: Stacked ^{19}F NMR (188 MHz) spectra (−150 ppm to 50 ppm) at different stages during reaction recorded in toluene-d$_8$.

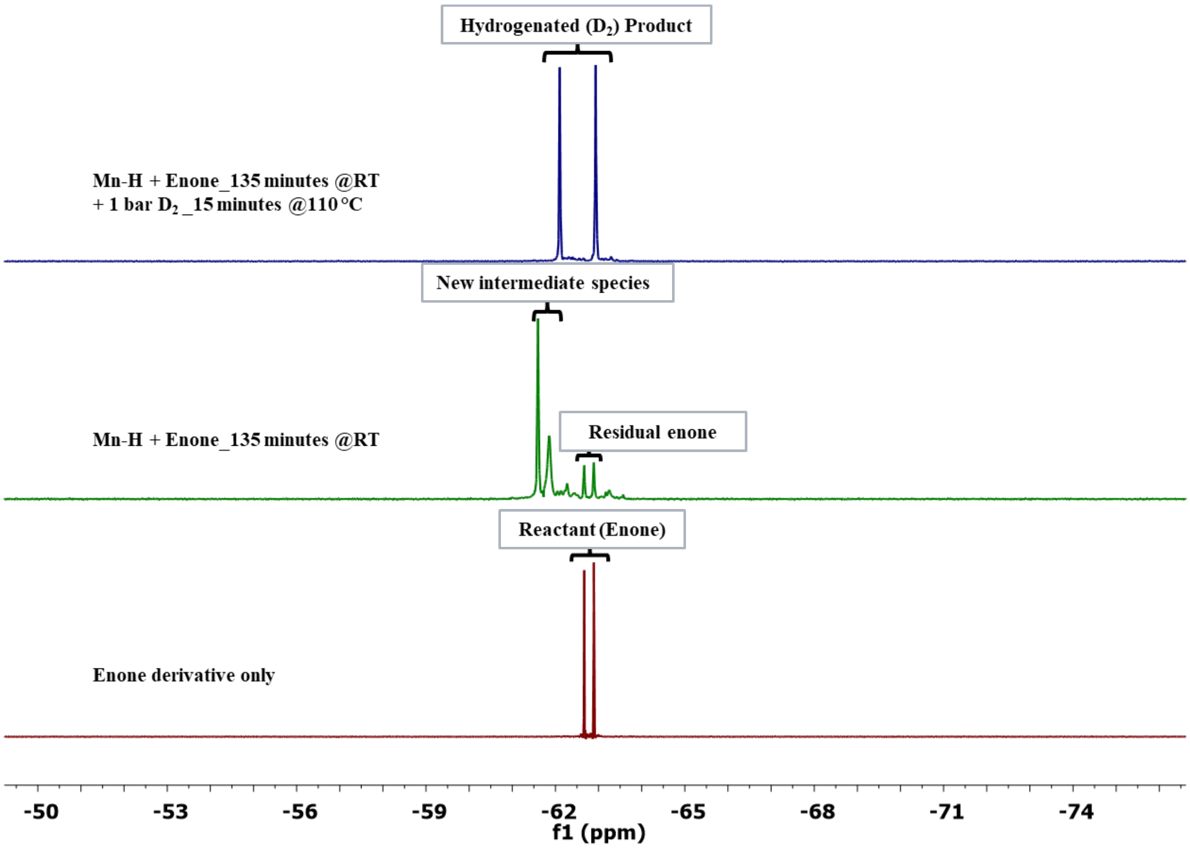

Figure S15: Stacked ^{19}F NMR (188 MHz) spectra (−75 ppm to −50 ppm) at different stages during reaction recorded in toluene-d_8.

Reaction of saturated ketone 6h with catalytic [(PC$_{NHC}$P)Mn(CO)$_2$H] in D_2 atmosphere:

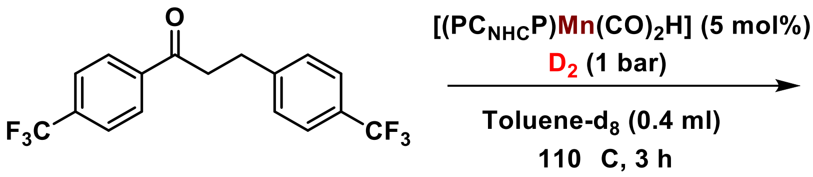

Scheme S7: Exposure of product **6h** with catalytic Mn-H catalyst in D_2.

Inside a N_2-filled glovebox, a J. Young NMR tube suitable for intermediate pressure (up to 10.5 bar) was charged with [(PC$_{NHC}$P)Mn(CO)$_2$H] (**1**, 1.81 mg, 0.0029 mmol, 0.05 equiv.), saturated ketone **6h** (20 mg, 0.058 mmol, 1 equiv.), and toluene-d$_8$ (0.4 mL). The tube was sealed and was taken out of the glovebox. Hereafter, the tube was degassed by freeze-pump-thaw cycles (3×) using Schlenk techniques and connected to an intermediate pressure deuterium gas (D_2) line. The line was purged by subsequent evacuation and filling with D_2 (3×). The tube was opened and was filled with deuterium gas at the desired pressure (1 bar). Next, the tube was sealed again and placed on a preheated oil bath at 110 °C. ^{1}H-NMR spectra were recorded immediately after charging D_2 and after 3 hours of heating at 110 °C. Both the spectra were stacked for comparison (**Figure S16**). After that, the solvent was evaporated and the deuterium (^{2}H)-NMR was recorded in 500 µL toluene and 5 µL of toluene-d$_8$ (**Figure S17**).

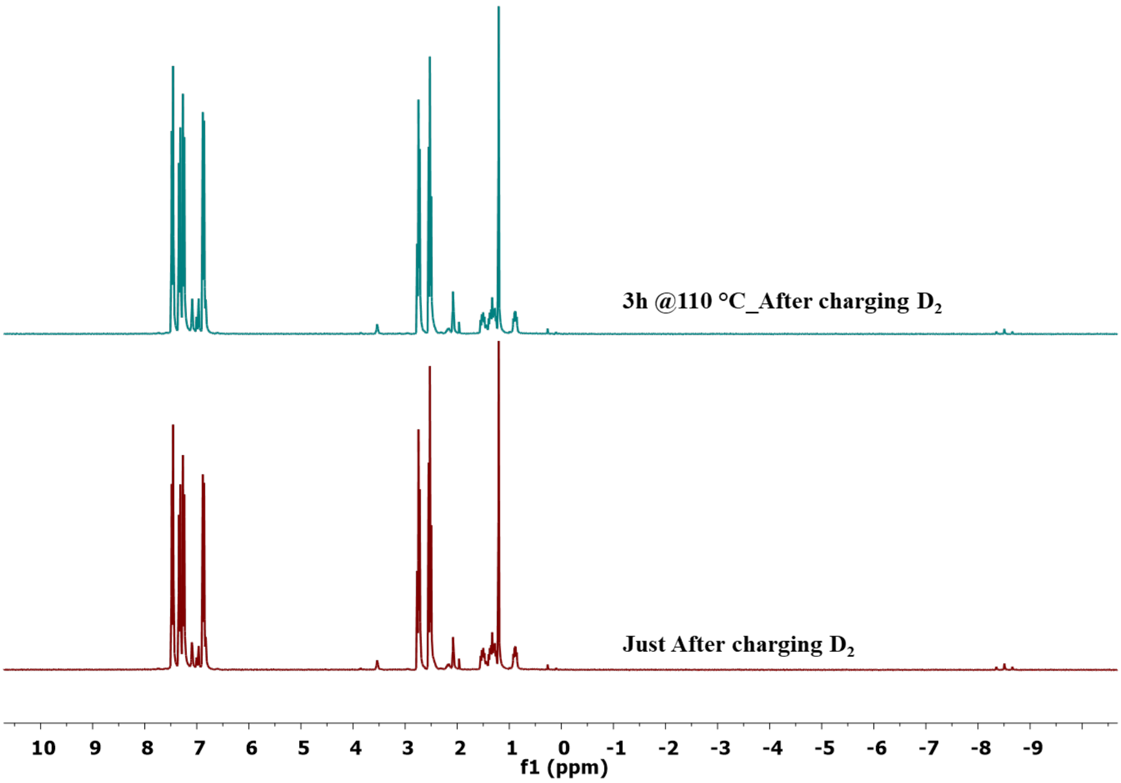

Figure S16: Stacked ^{1}H NMR (300 MHz) spectra (10 ppm to -10 ppm) at different stages during reaction recorded in toluene-d$_8$.

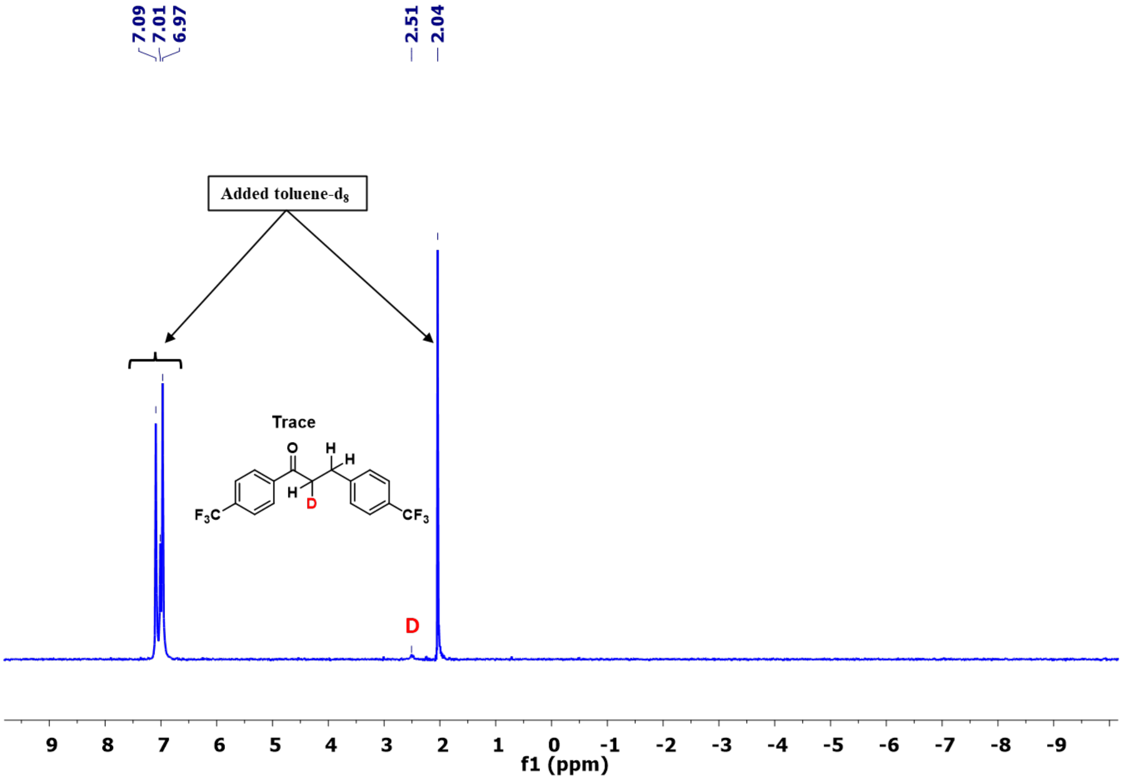

Figure S17: Deuterium (^{2}H) NMR spectrum (46 MHz) of the crude reaction mixture after completion of reaction (5 µL toluene-d$_8$ in 500 µL toluene)

Reaction of saturated ketone 6h with stoichiometric [(PC$_{NHC}$P)Mn(CO)$_2$H] in D_2 atmosphere:

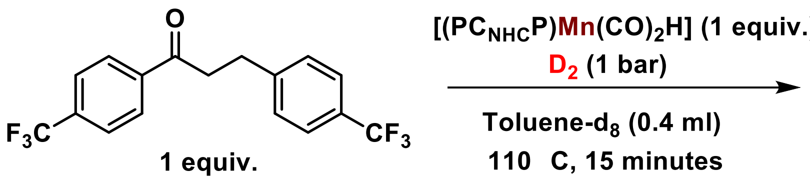

Scheme S8: Exposure of product **6h** with stoichiometric Mn-H catalyst in D_2.

Inside a N_2-filled glovebox, a J. Young NMR tube suitable for intermediate pressure (up to 10.5 bar) was charged with [(PC$_{NHC}$P)Mn(CO)$_2$H] (**1**, 9.37 mg, 0.015 mmol, 1 equiv.), saturated ketone **6h** (5.19 mg, 0.015 mmol, 1 equiv.), and toluene-d$_8$ (0.4 mL). The tube was sealed and was taken out of the glovebox. Hereafter, the tube was degassed by freeze-pump-thaw cycles (3×) using Schlenk techniques and connected to an intermediate pressure deuterium gas (D_2) line. The line was purged by subsequent evacuation and filling with D_2 (3×). The tube was opened and was filled with deuterium gas at the desired pressure (1 bar). Next, the tube was sealed again and placed on a preheated oil bath at 110 °C. ^{1}H- and ^{31}P-NMR spectra were recorded before charging D_2, immediately after charging D_2 and after 15 minutes of heating at 110 °C. All the spectra were stacked for comparison (**Figure S18 and Figure S19**). After that, the solvent was evaporated and the deuterium (^{2}H)-NMR was recorded in 500 µL toluene and 5 µL of toluene-d$_8$ (**Figure S20**).

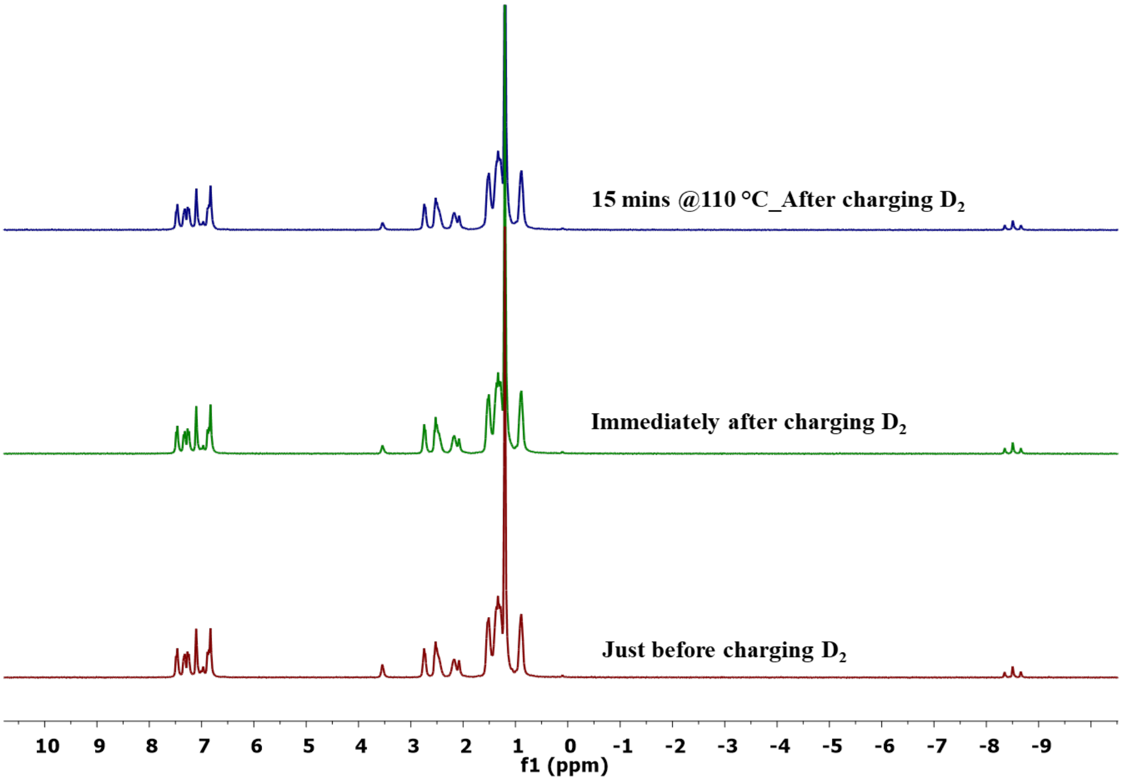

Figure S18: Stacked ^{1}H NMR (300 MHz) spectra (10 ppm to -10 ppm) at different stages during reaction recorded in toluene-d_8.

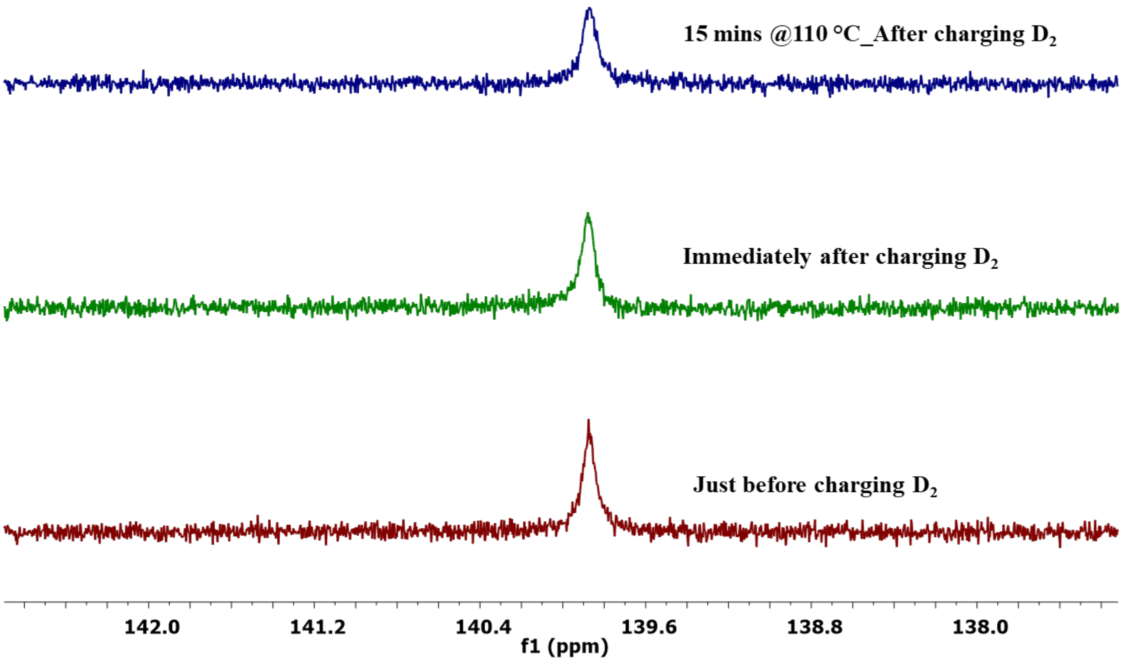

Figure S19: Stacked ^{31}P NMR (121 MHz) spectra (142 ppm to 138 ppm) at different stages during reaction recorded in toluene-d_8.

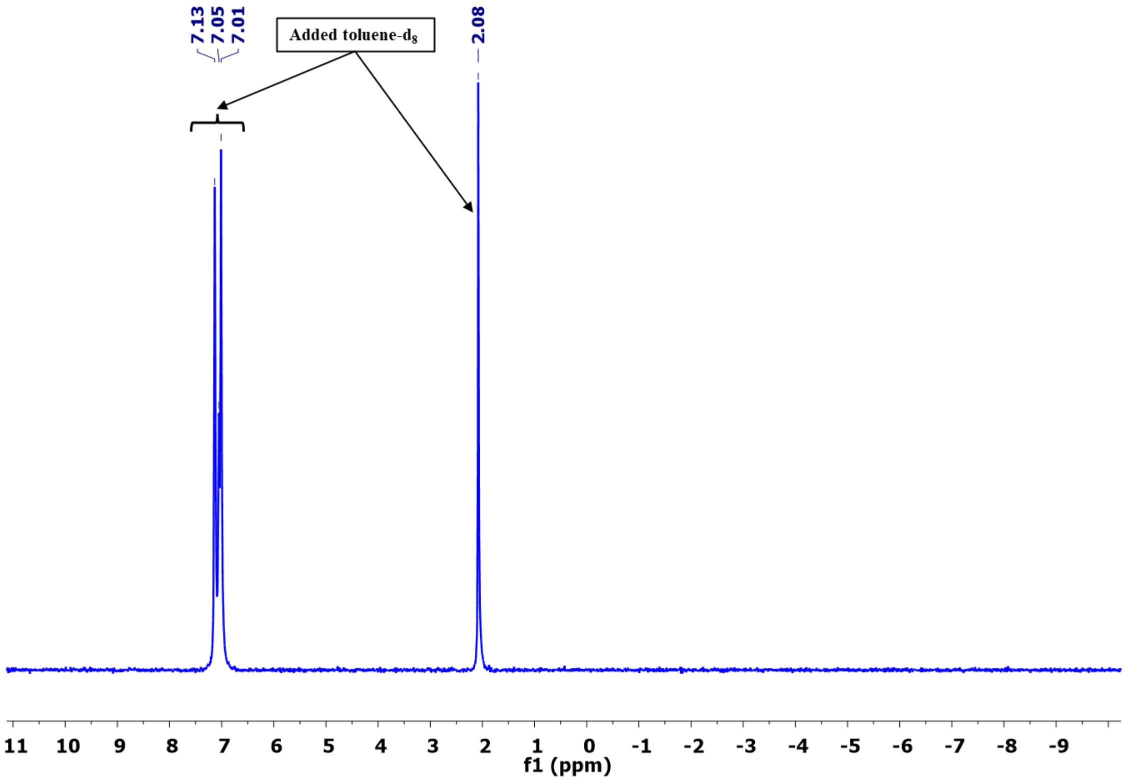

Figure S20: Deuterium (^{2}H) NMR spectrum (46 MHz) of the crude reaction mixture after completion of reaction (5 µL toluene-d_8 in 500 µL toluene)

NMR and mass spectra of selected enone substrates:

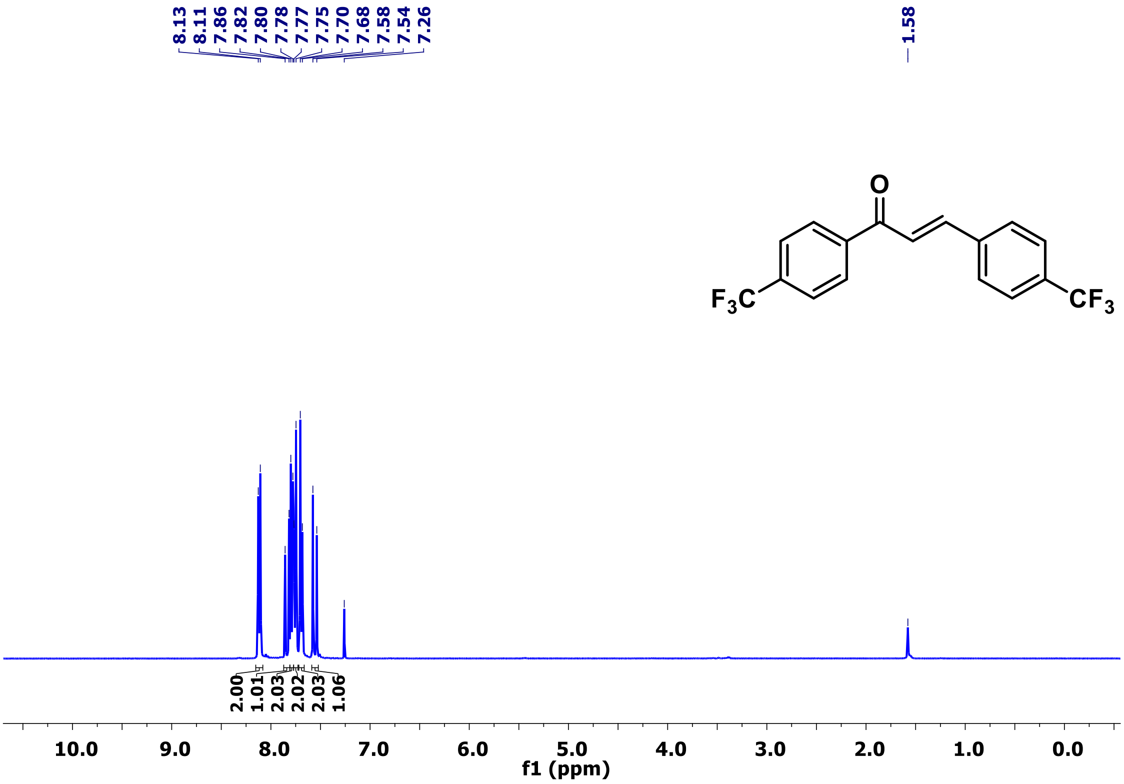

Figure S21: ^{1}H NMR spectrum (400 MHz) of **5h** in $CDCl_3$

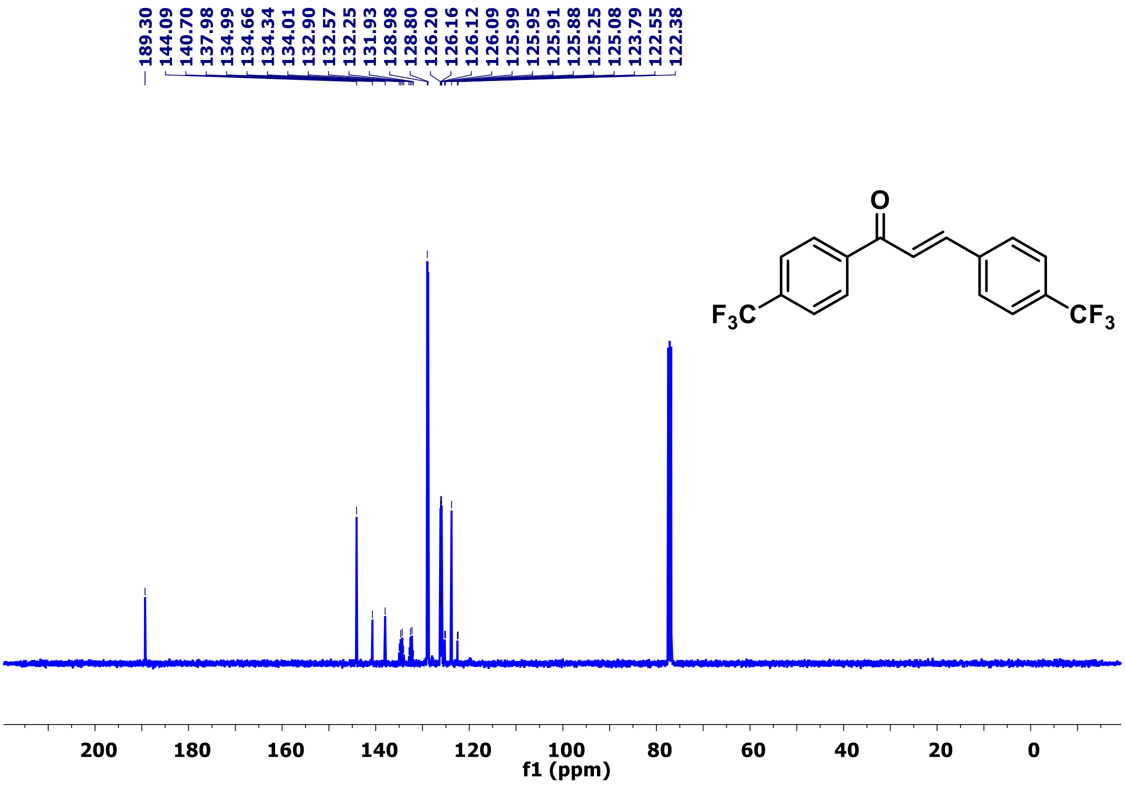

Figure S22: $^{13}C\{^{1}H\}$ NMR spectrum (101 MHz) of **5h** in $CDCl_3$

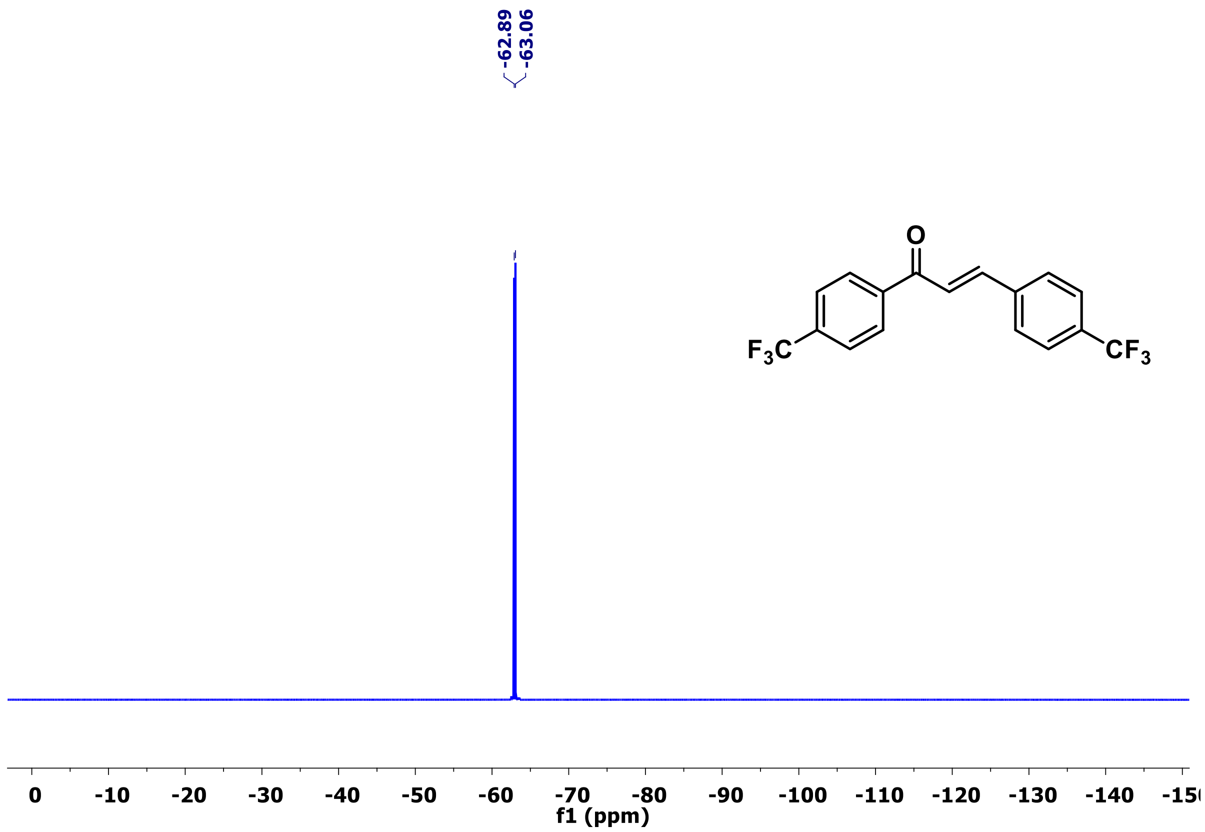

Figure S23: $^{19}F\{^{1}H\}$ NMR spectrum (377 MHz) of **5h** in $CDCl_3$

Compound Spectrum SmartFormula Report

Analysis Info

Analysis Name	D:\Data\Graham\Dr_5248000001.d	Acquisition Date	10/12/2023 15:56:16
Method	APCI_pos_SolidProbe.m	Operator	Larisa Panz
Sample Name	KD 666772	Instrument	maXis impact 282001.00128
Comment			

Acquisition Parameter

Source Type	APCI	Ion Polarity	Positive	Set Nebulizer	1.2 Bar
Focus	Active	Set Capillary	3000 V	Set Dry Heater	120 °C
Scan Begin	50 m/z	Set End Plate Offset	-500 V	Set Dry Gas	1.5 l/min
Scan End	2000 m/z	Set Charging Voltage	2000 V	Set Divert Valve	Source
		Set Corona	5000 nA	Set APCI Heater	200 °C

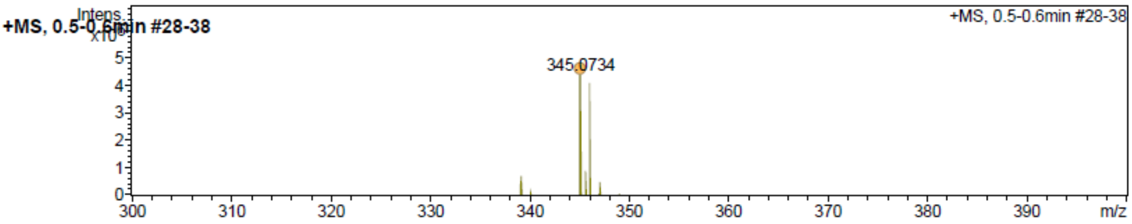

Meas. m/z	#	Ion Formula	m/z	err [ppm]	mSigma	# mSigma	Score	rdb	e¯ Conf	N-Rule	|err| [mDa]
345.0734	1	C17H11F6O	345.0709	-7.5	436.2	1	100.00	9.5	even	ok	2.6
	2	C10H11F6N6O	345.0893	46.0	467.6	2	0.00	5.5	even	ok	15.9

Figure S24: High resolution mass spectrum of **5h**

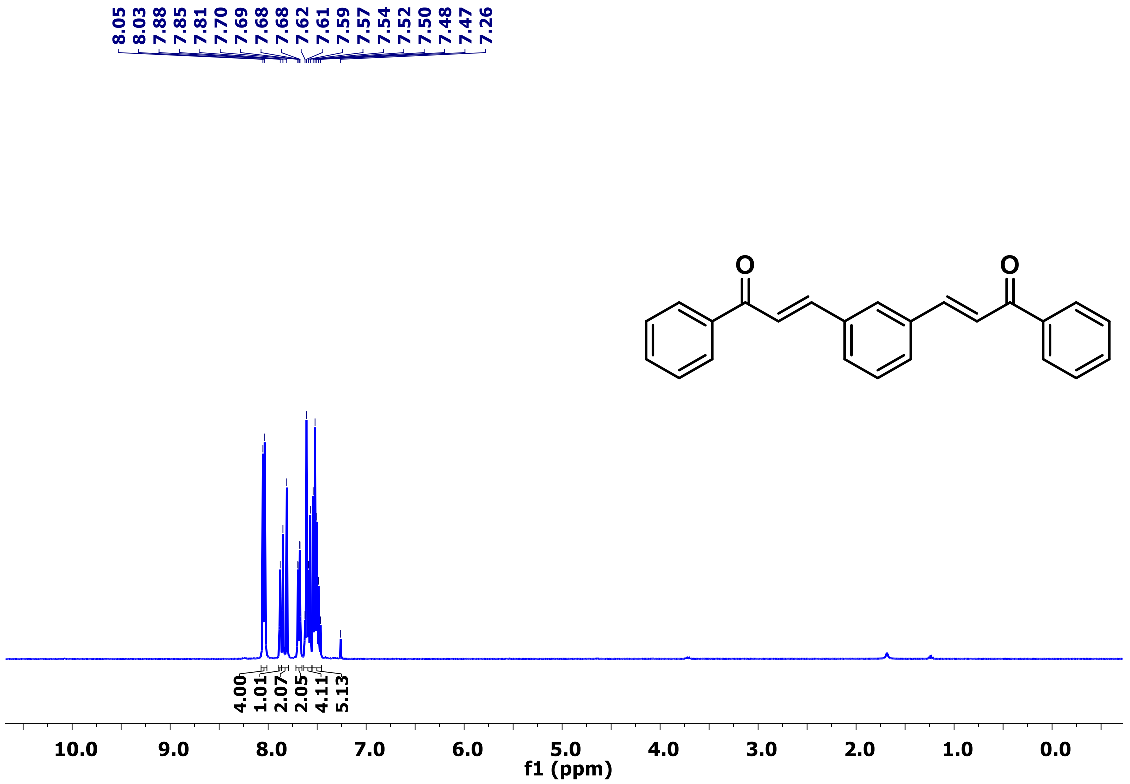

Figure S25: ^{1}H NMR spectrum (400 MHz) of **5p** in $CDCl_3$

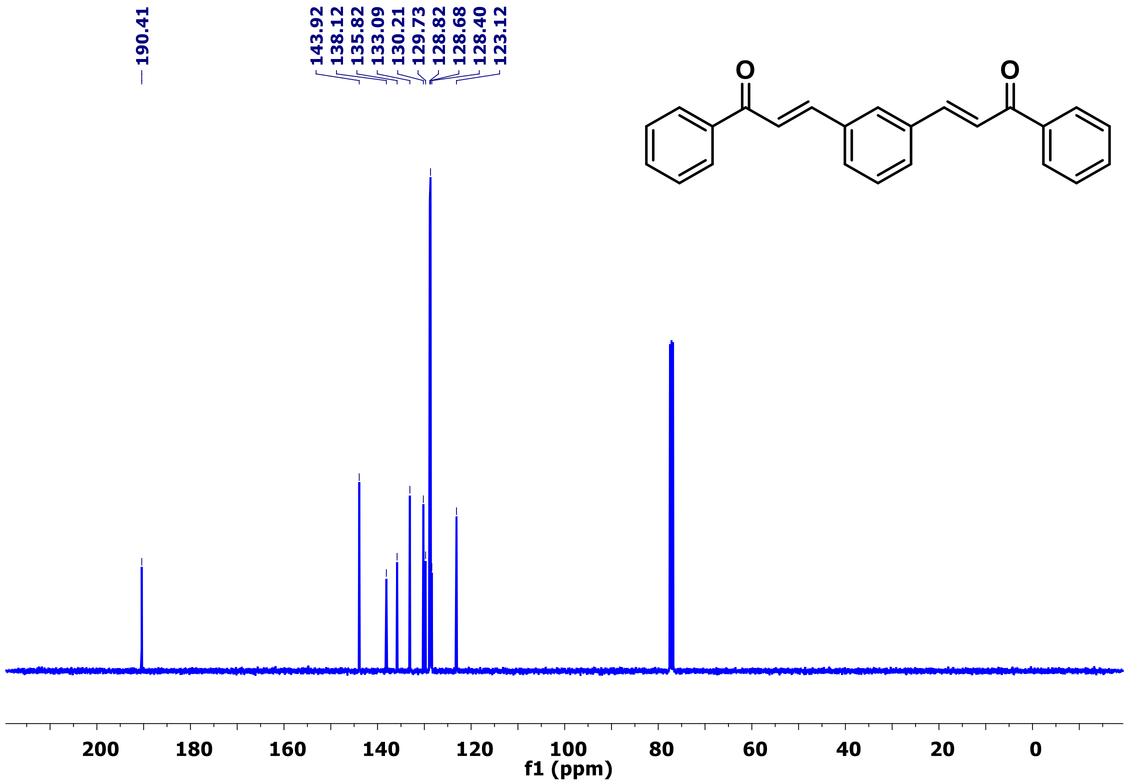

Figure S26: $^{13}C\{^{1}H\}$ NMR spectrum (101 MHz) of **5p** in $CDCl_3$

Compound Spectrum SmartFormula Report

**Analysis Info**		Acquisition Date	10/12/2023 15:52:09	
Analysis Name	D:\Data\Graham\Dr_5247000002.d			
Method	APCI_pos_SolidProbe.m	Operator	Larisa Panz	
Sample Name	KD 666770	Instrument	maXis impact	282001.00128
Comment				

Acquisition Parameter

Source Type	APCI	Ion Polarity	Positive	Set Nebulizer	1.2 Bar
Focus	Active	Set Capillary	3000 V	Set Dry Heater	120 °C
Scan Begin	50 m/z	Set End Plate Offset	-500 V	Set Dry Gas	1.5 l/min
Scan End	2000 m/z	Set Charging Voltage	2000 V	Set Divert Valve	Source
		Set Corona	5000 nA	Set APCI Heater	200 °C

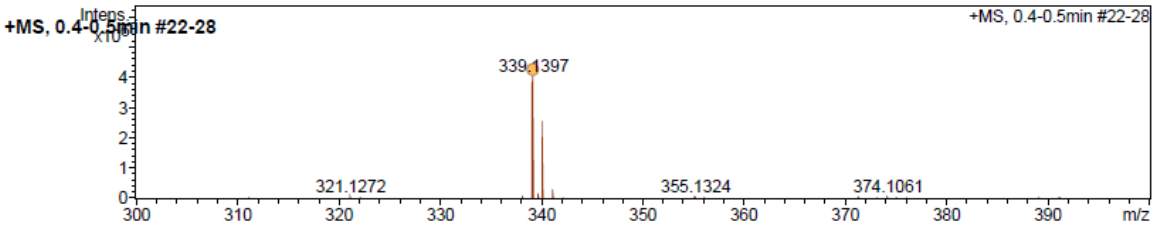

Meas. m/z	#	Ion Formula	m/z	err [ppm]	mSigma	# mSigma	Score	rdb	e⁻ Conf	N-Rule	|err| [mDa]	|err| [mDa]
339.1397	1	C24H19O2	339.1380	-5.1	218.8	1	100.00	15.5	even	ok	1.7	1.7
	2	C17H19N6O2	339.1564	49.3	250.5	2	0.00	11.5	even	ok	16.7	16.7
	3	C10H19N12O2	339.1748	103.6	282.0	3	0.00	7.5	even	ok	35.2	35.2

Figure S27: High resolution mass spectrum of **5p**

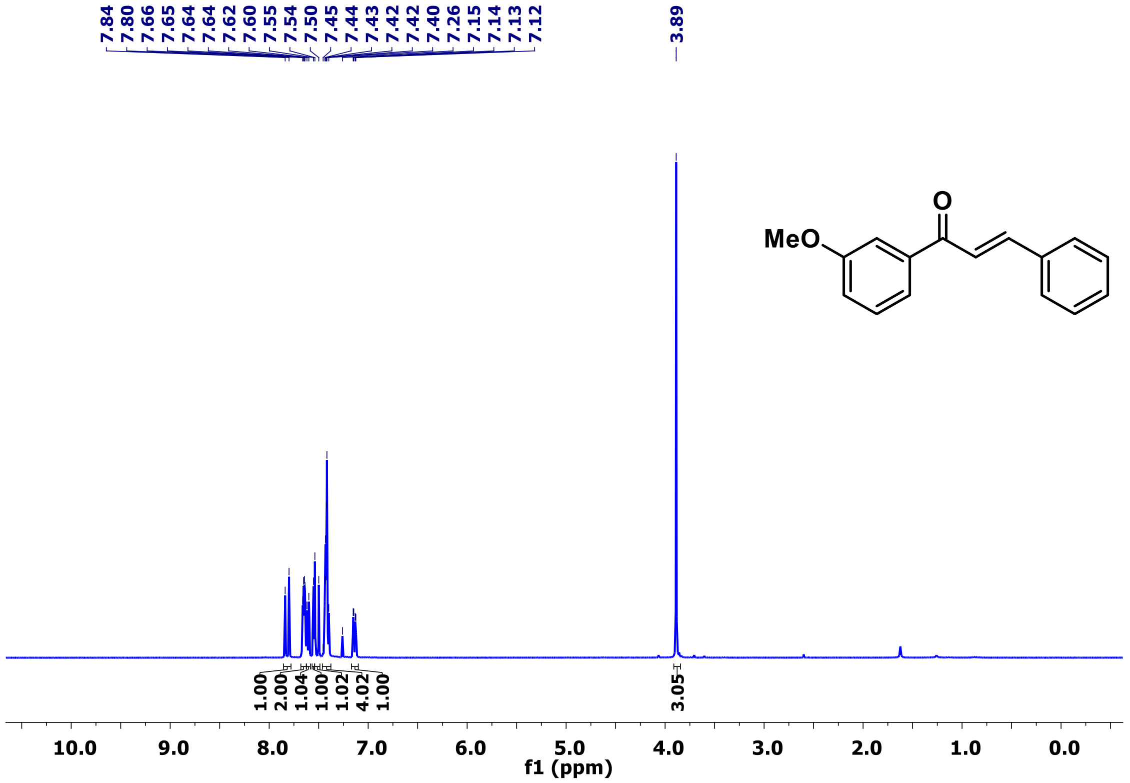

Figure S28: ^{1}H NMR spectrum (400 MHz) of **5t** in $CDCl_3$

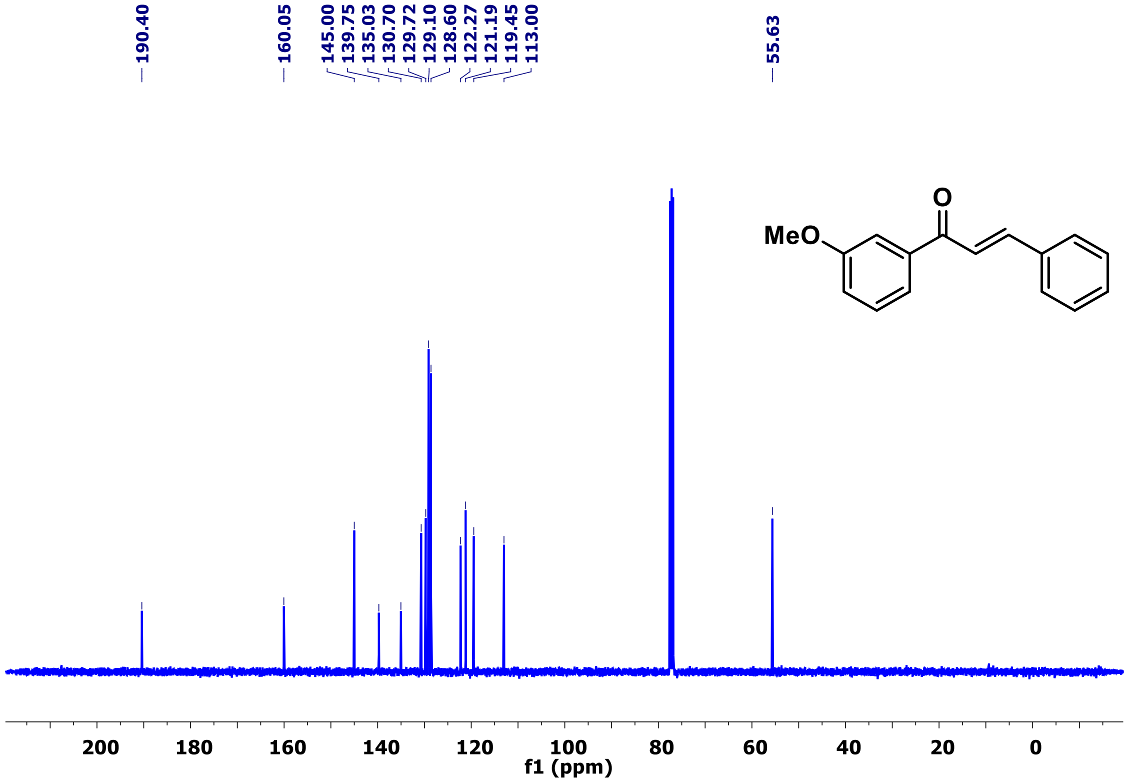

Figure S29: $^{13}C\{^{1}H\}$ NMR spectrum (101 MHz) of **5t** in $CDCl_3$

Compound Spectrum SmartFormula Report

Analysis Info

Analysis Name	D:\Data\Graham\Dr_5246000003.d	Acquisition Date	10/12/2023 15:46:07
Method	APCI_pos_SolidProbe.m	Operator	Larisa Panz
Sample Name	KD 666771	Instrument	maXis impact 282001.00128
Comment			

Acquisition Parameter

Source Type	APCI	Ion Polarity	Positive	Set Nebulizer	1.2 Bar
Focus	Active	Set Capillary	3000 V	Set Dry Heater	120 °C
Scan Begin	50 m/z	Set End Plate Offset	-500 V	Set Dry Gas	1.5 l/min
Scan End	2000 m/z	Set Charging Voltage	2000 V	Set Divert Valve	Source
		Set Corona	5000 nA	Set APCI Heater	200 °C

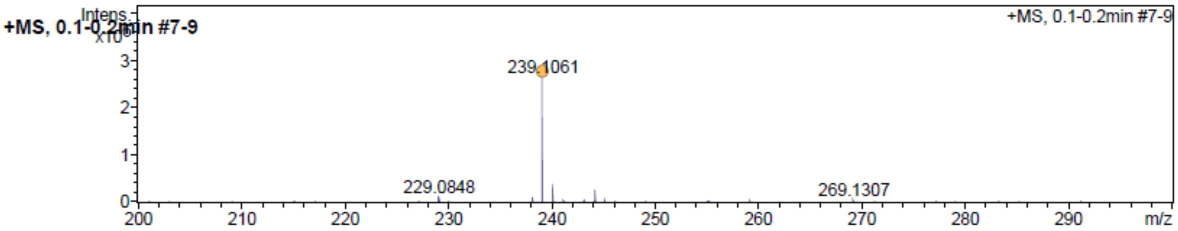

Meas. m/z	#	Ion Formula	m/z	err [ppm]	mSigma	# mSigma	Score	rdb	e⁻ Conf	N-Rule	|err| [mDa]
239.1061	1	C9H15N6O2	239.1251	79.6	14.6	1	0.00	5.5	even	ok	19.0
	2	C16H15O2	239.1067	2.4	16.8	2	100.00	9.5	even	ok	0.6

Figure S30: High resolution mass spectrum of **5t**

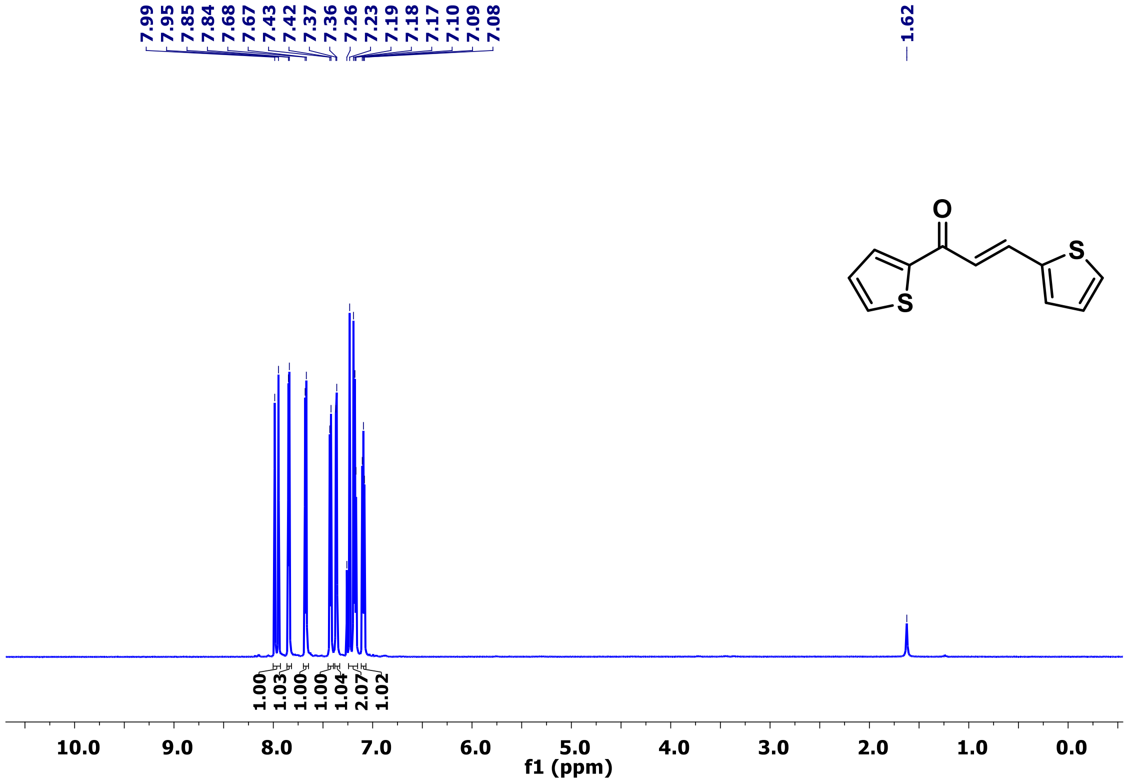

Figure S31: ^{1}H NMR spectrum (400 MHz) of **5u** in $CDCl_3$

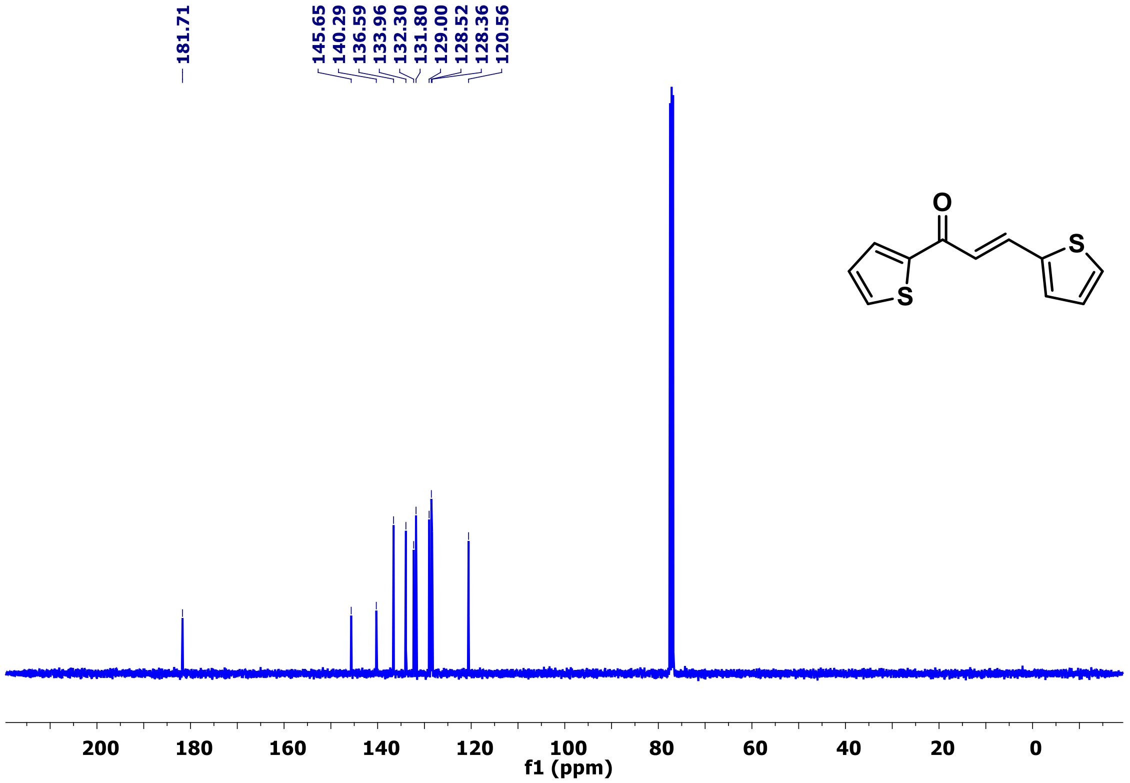

Figure S32: $^{13}C\{^{1}H\}$ NMR spectrum (101 MHz) of **5u** in $CDCl_3$

Compound Spectrum SmartFormula Report

**Analysis Info**			
Analysis Name	D:\Data\Graham\Dr_5249000001.d	Acquisition Date	10/12/2023 15:59:17
Method	APCI_pos_SolidProbe.m	Operator	Larisa Panz
Sample Name	KD 666773	Instrument	maXis impact 282001.00128
Comment			

Acquisition Parameter

Source Type	APCI	Ion Polarity	Positive	Set Nebulizer	1.2 Bar
Focus	Active	Set Capillary	3000 V	Set Dry Heater	120 °C
Scan Begin	50 m/z	Set End Plate Offset	-500 V	Set Dry Gas	1.5 l/min
Scan End	2000 m/z	Set Charging Voltage	2000 V	Set Divert Valve	Source
		Set Corona	5000 nA	Set APCI Heater	200 °C

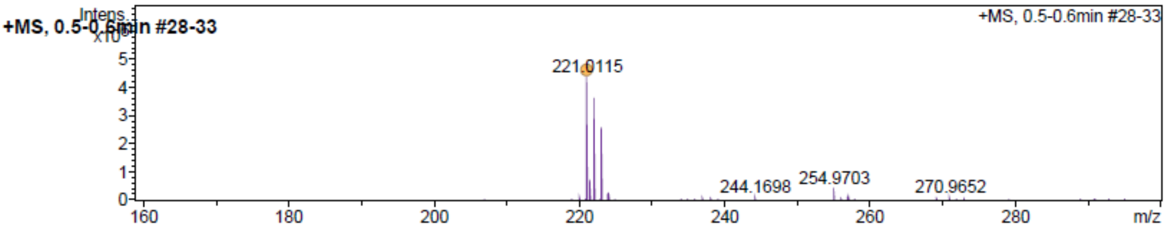

Meas. m/z	#	Ion Formula	m/z	err [ppm]	mSigma	# mSigma	Score	rdb	e⁻ Conf	N-Rule	|err| [mDa]
221.0115	1	C11H9OS2	221.0089	-11.8	425.6	1	100.00	7.5	even	ok	2.6
	2	C4H9N6OS2	221.0274	71.6	449.4	2	0.00	3.5	even	ok	15.8

Figure S33: High resolution mass spectrum of **5u**

NMR spectra of crude and isolated products:

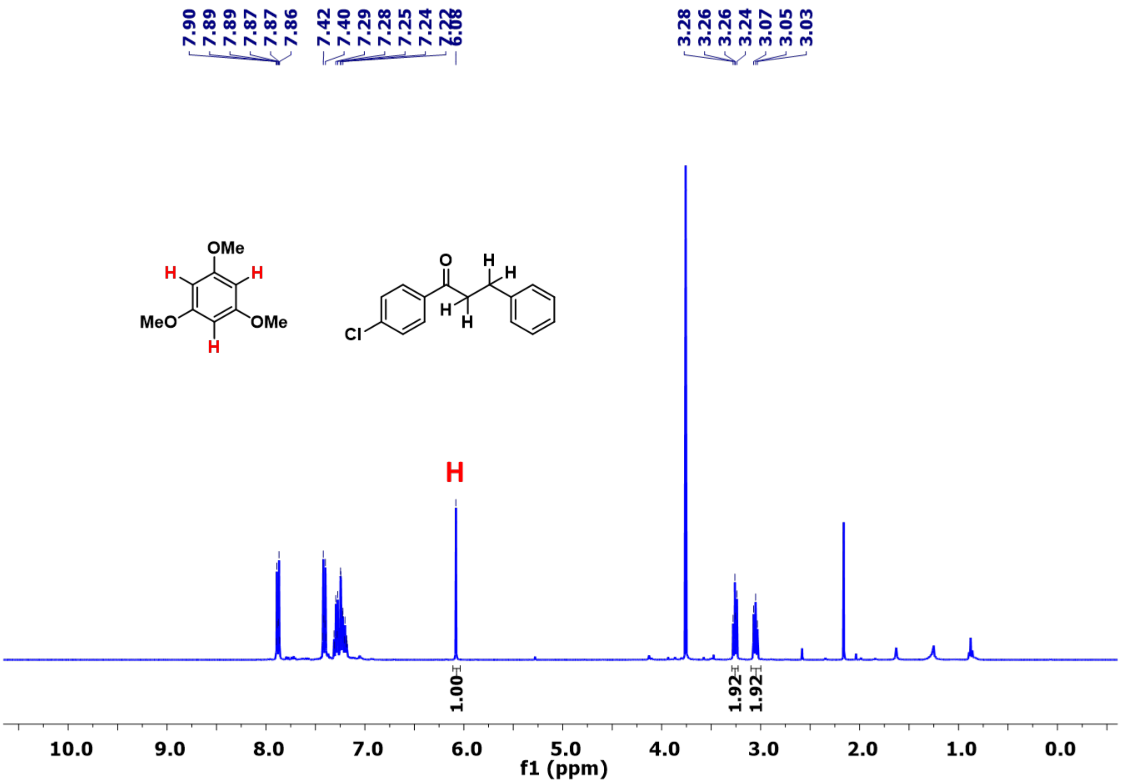

Figure S34: ^{1}H NMR spectrum (400 MHz, $CDCl_3$) of crude product mixture with 1/3 equiv. 1,3,5-trimethoxybenzene after hydrogenation of **5a**

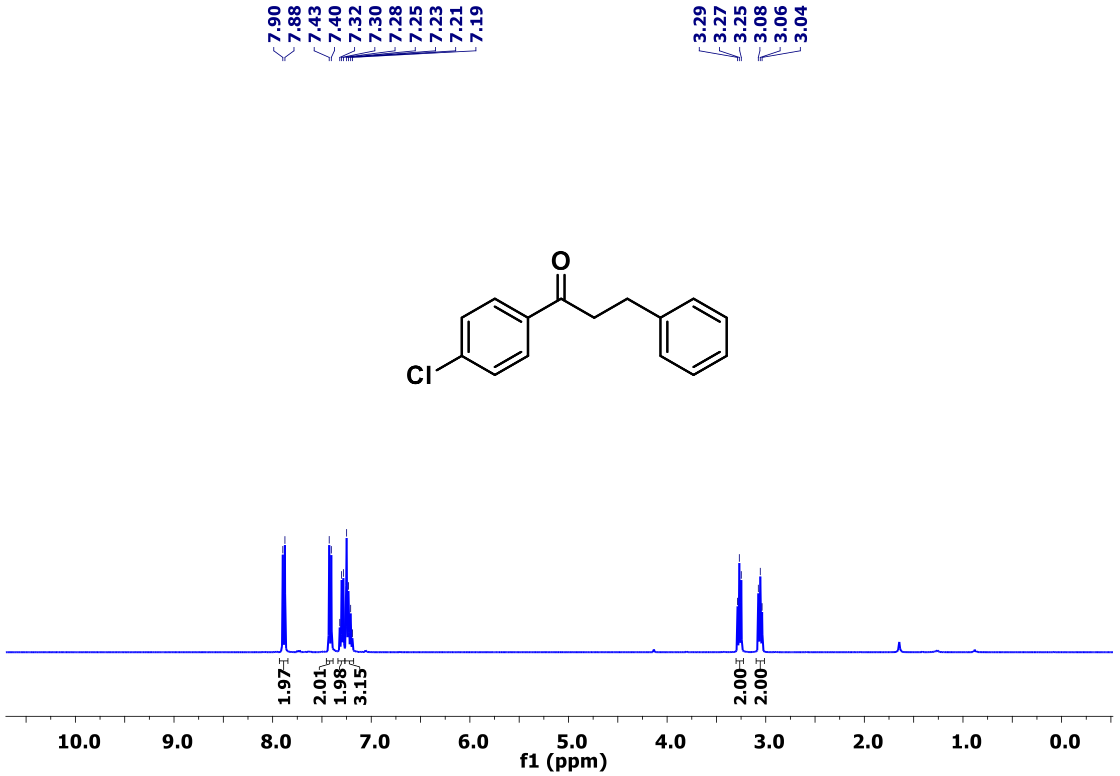

Figure S35: ^{1}H NMR spectrum (400 MHz) of **6a** in $CDCl_3$

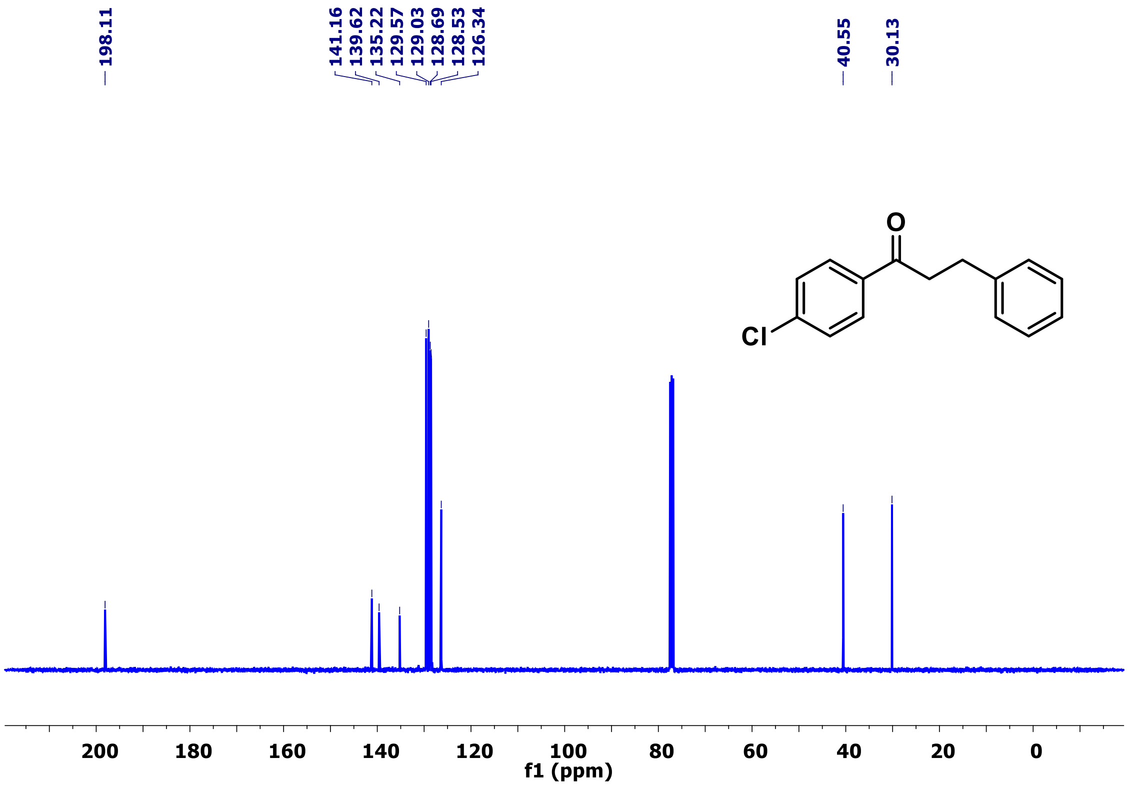

Figure S36: $^{13}C\{^{1}H\}$ NMR spectrum (101 MHz) of **6a** in $CDCl_3$

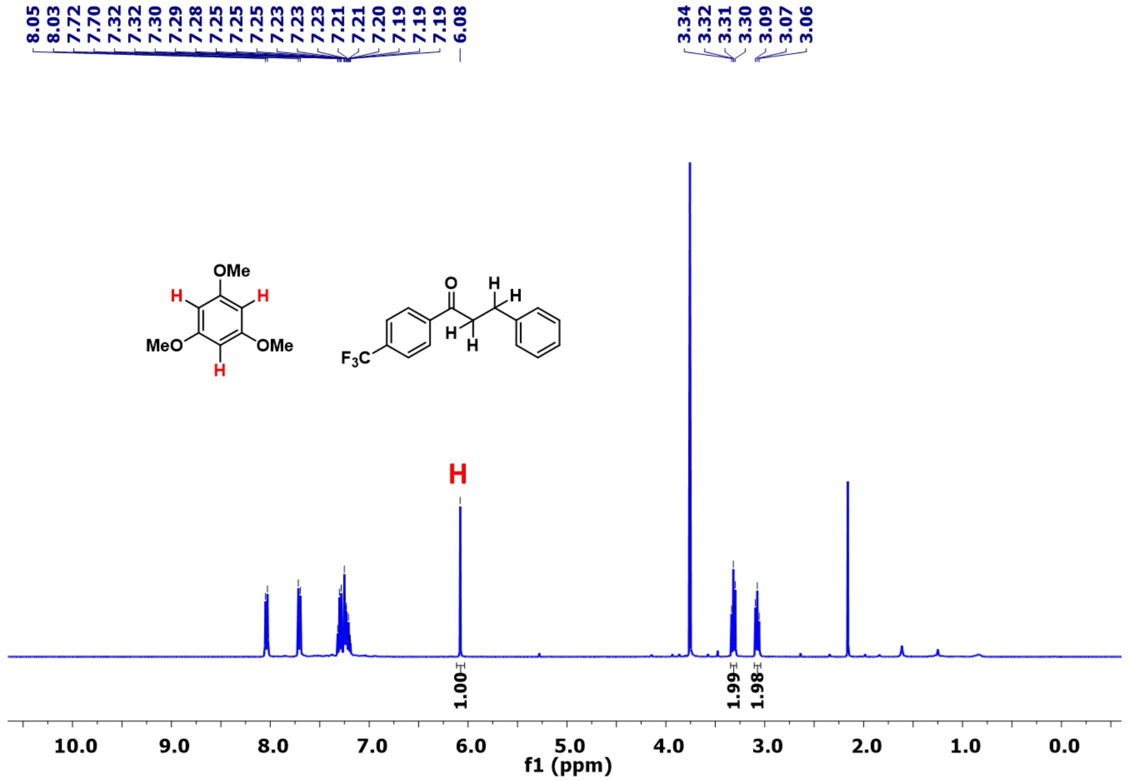

Figure S37: ^{1}H NMR spectrum (400 MHz, $CDCl_3$) of crude product mixture with 1/3 equiv. 1,3,5-trimethoxybenzene after hydrogenation of **5b**

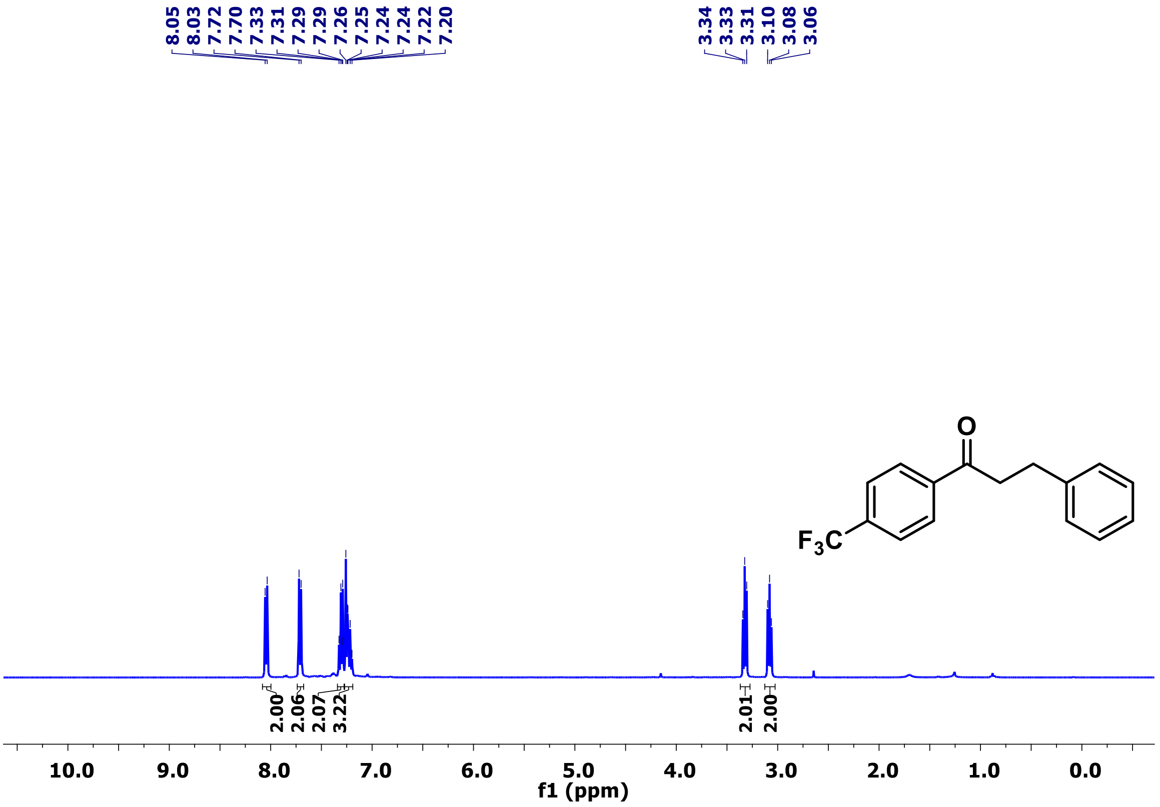

Figure S38: ^{1}H NMR spectrum (400 MHz) of **6b** in $CDCl_3$

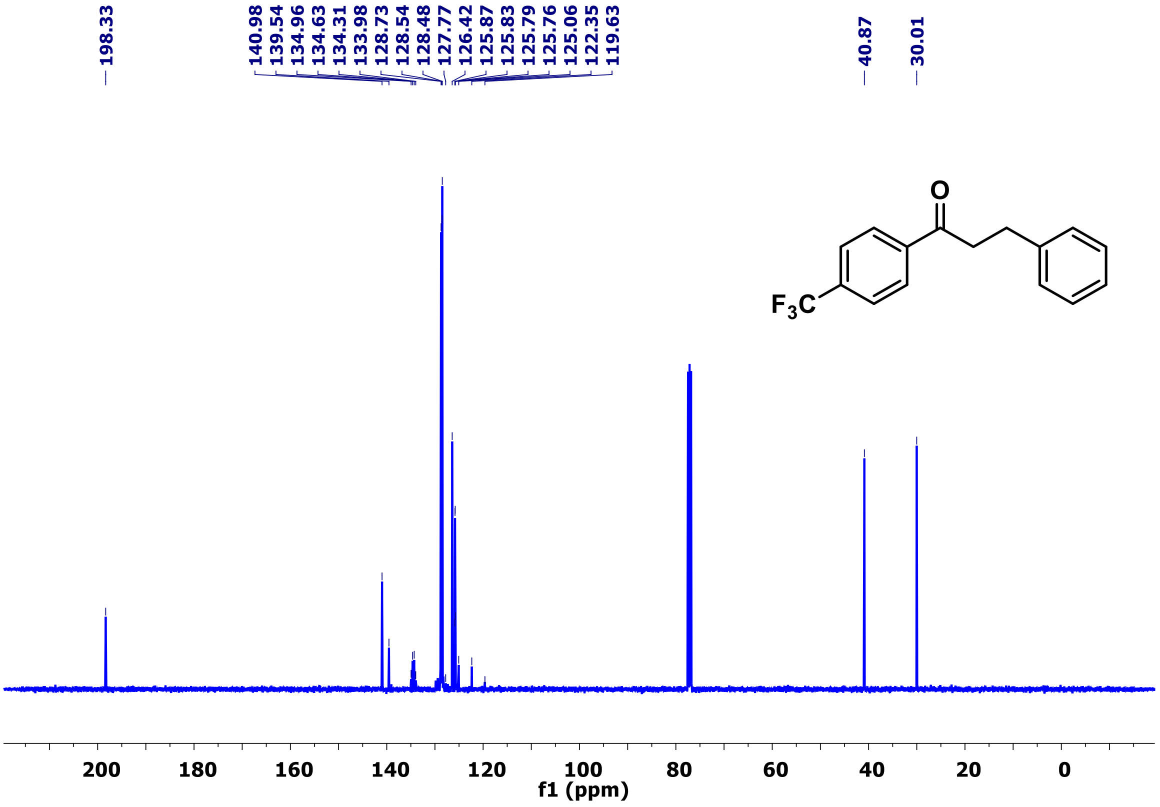

Figure S39: $^{13}C\{^{1}H\}$ NMR spectrum (101 MHz) of **6b** in $CDCl_3$

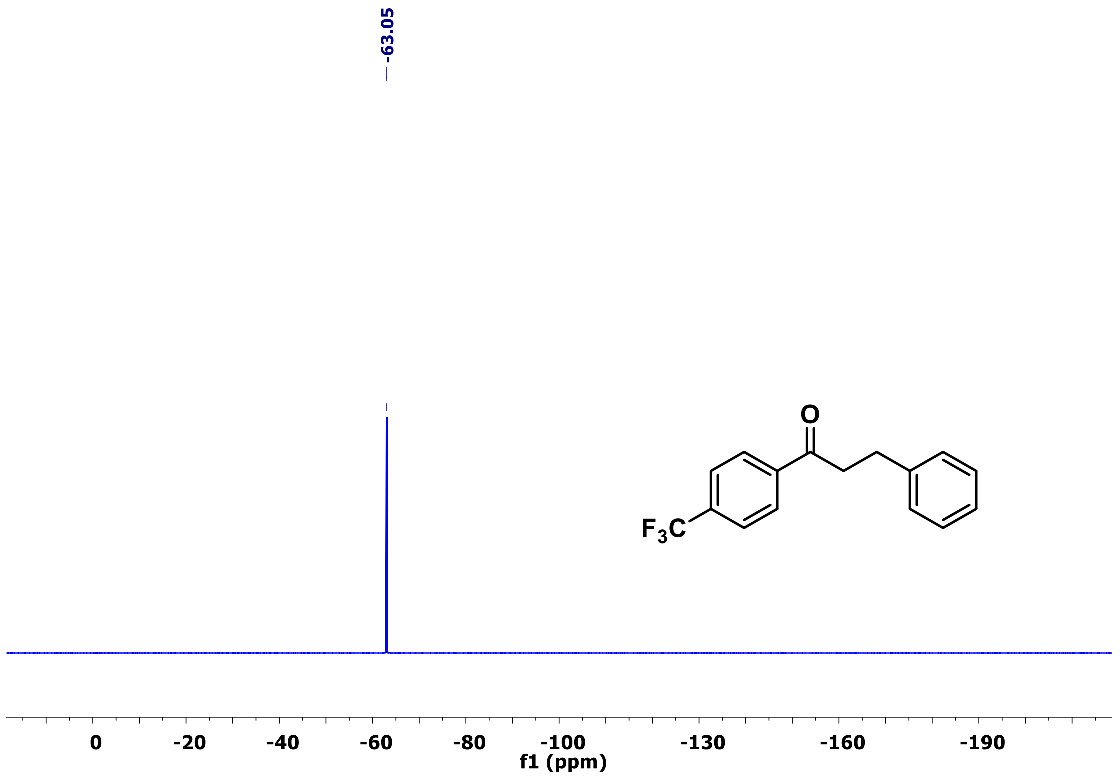

Figure S40:$^{19}F\{^{1}H\}$ NMR spectrum (377 MHz) of **6b** in $CDCl_3$

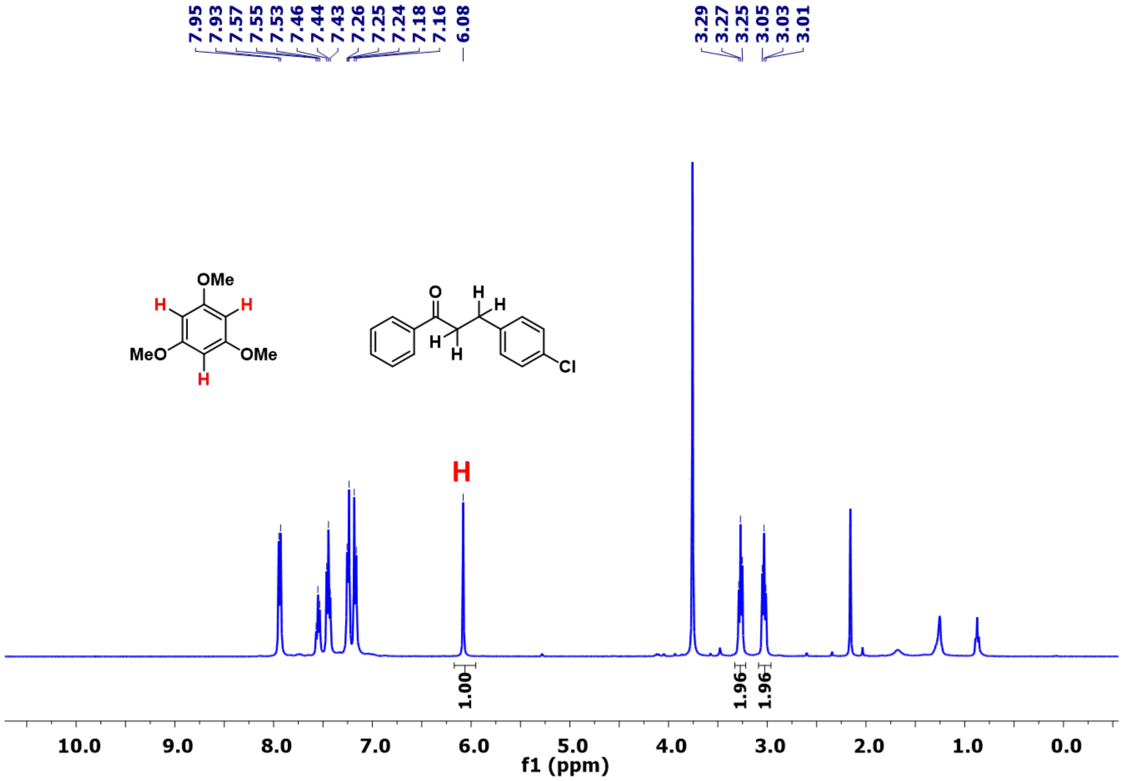

Figure S41: ^{1}H NMR spectrum (400 MHz, $CDCl_3$) of crude product mixture with 1/3 equiv. 1,3,5-trimethoxybenzene after hydrogenation of **5c**

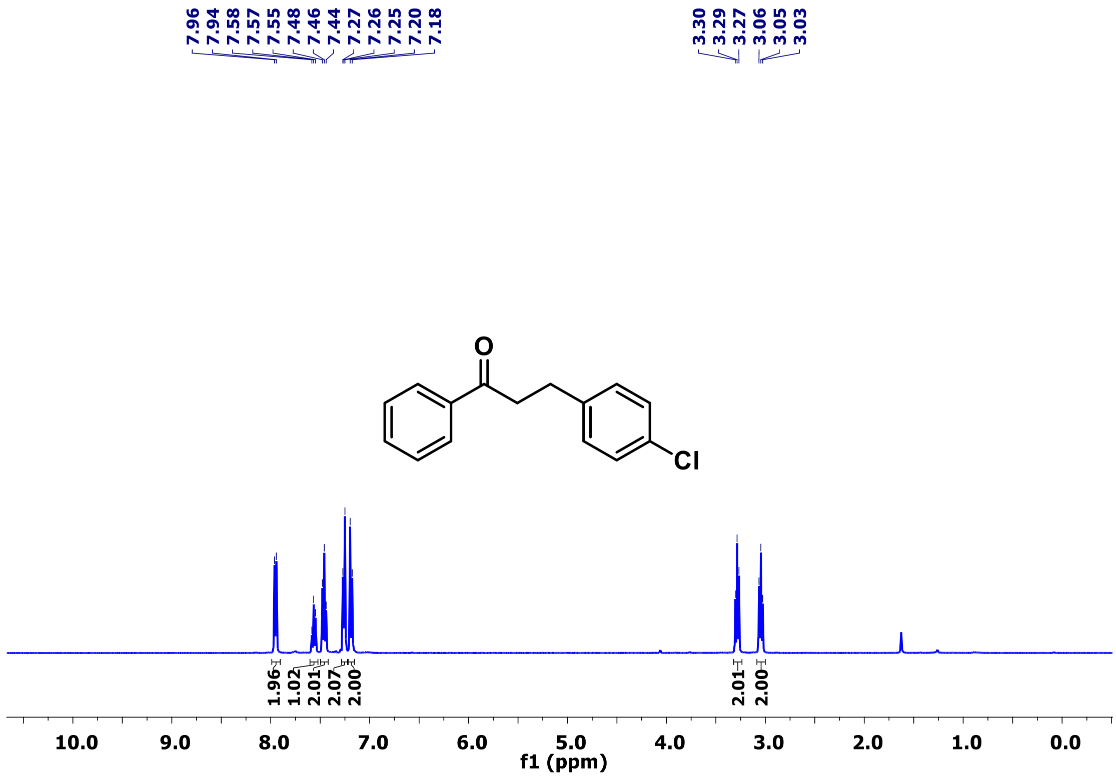

Figure S42: ^{1}H NMR spectrum (400 MHz) of **6c** in $CDCl_3$

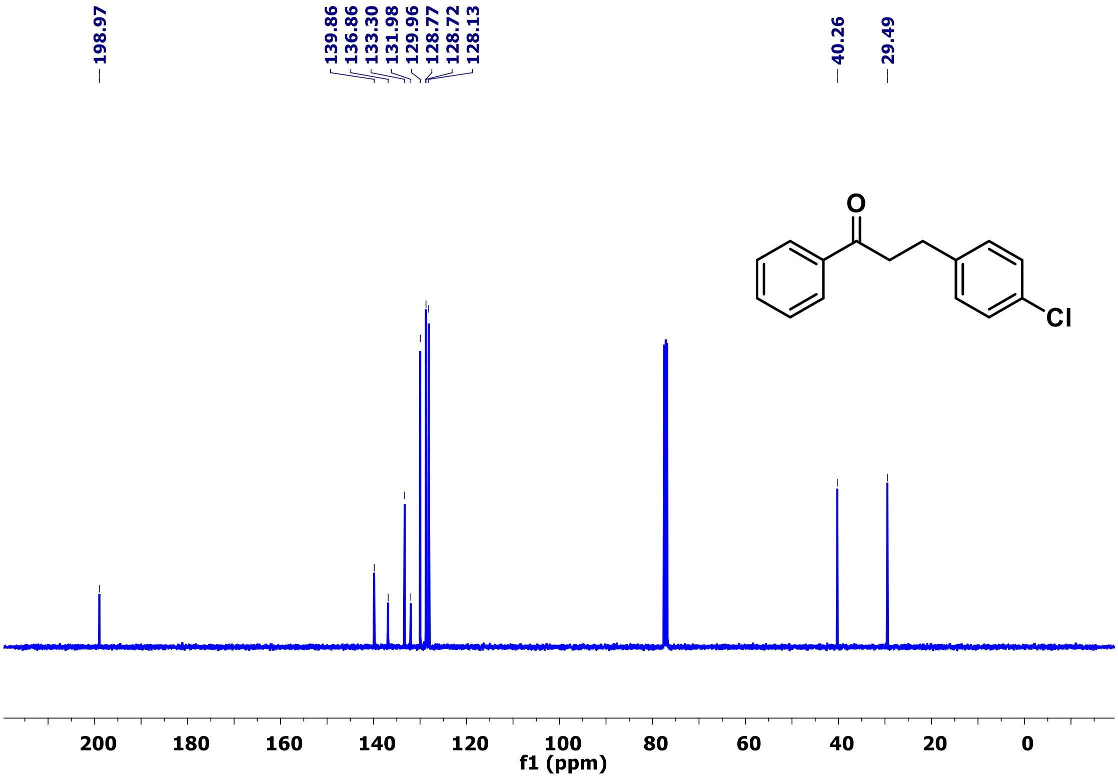

Figure S43: $^{13}C\{^{1}H\}$ NMR spectrum (101 MHz) of **6c** in $CDCl_3$

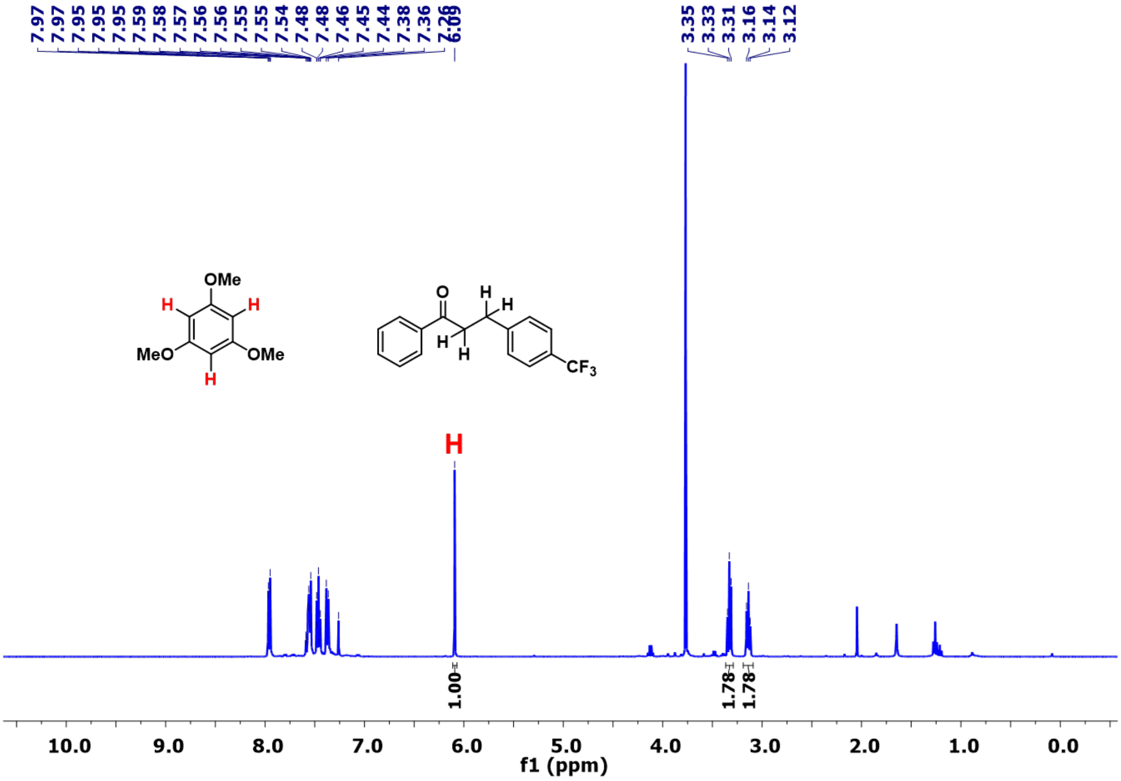

Figure S44: ^{1}H NMR spectrum (400 MHz, $CDCl_3$) of crude product mixture with 1/3 equiv. 1,3,5-trimethoxybenzene after hydrogenation of **5d**

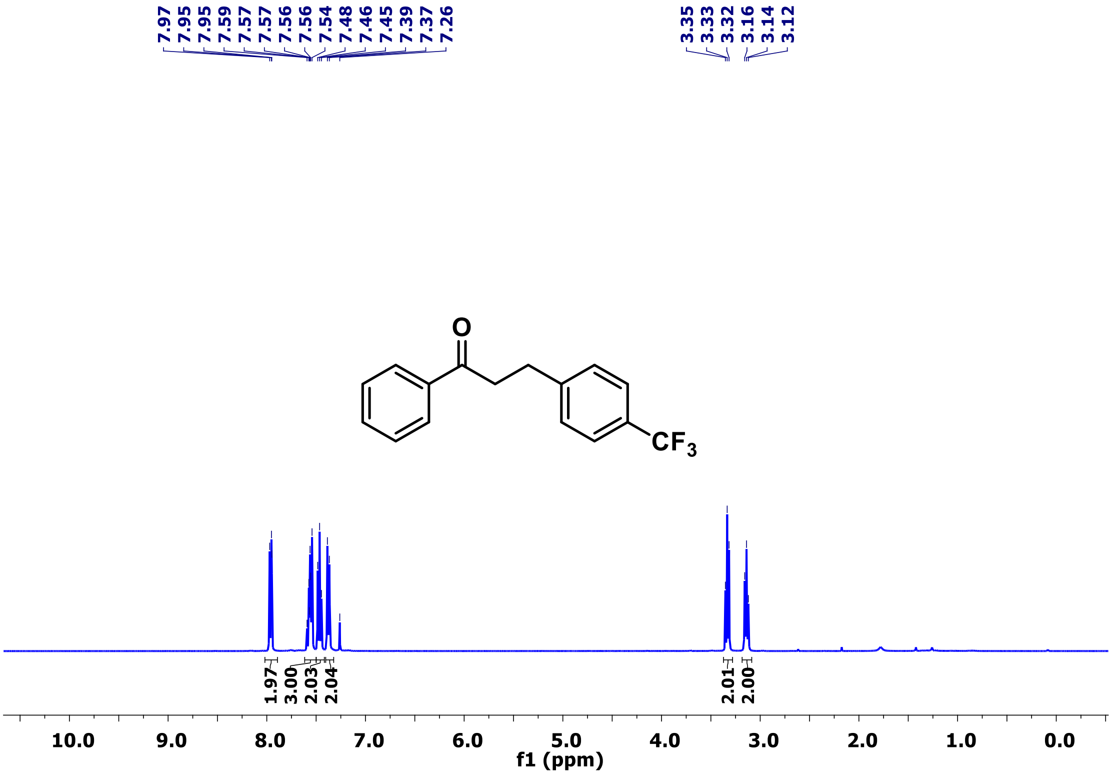

Figure S45: ^{1}H NMR spectrum (400 MHz) of **6d** in $CDCl_3$

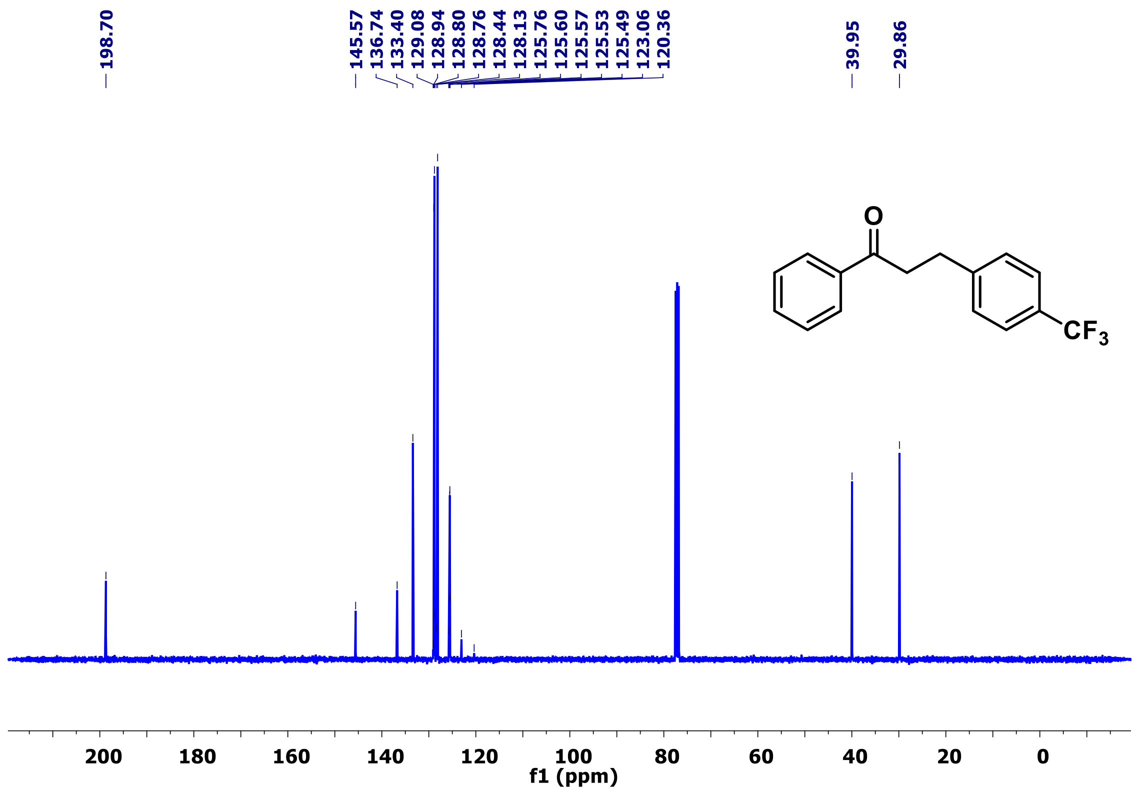

Figure S46: $^{13}C\{^{1}H\}$ NMR spectrum (101 MHz) of **6d** in $CDCl_3$

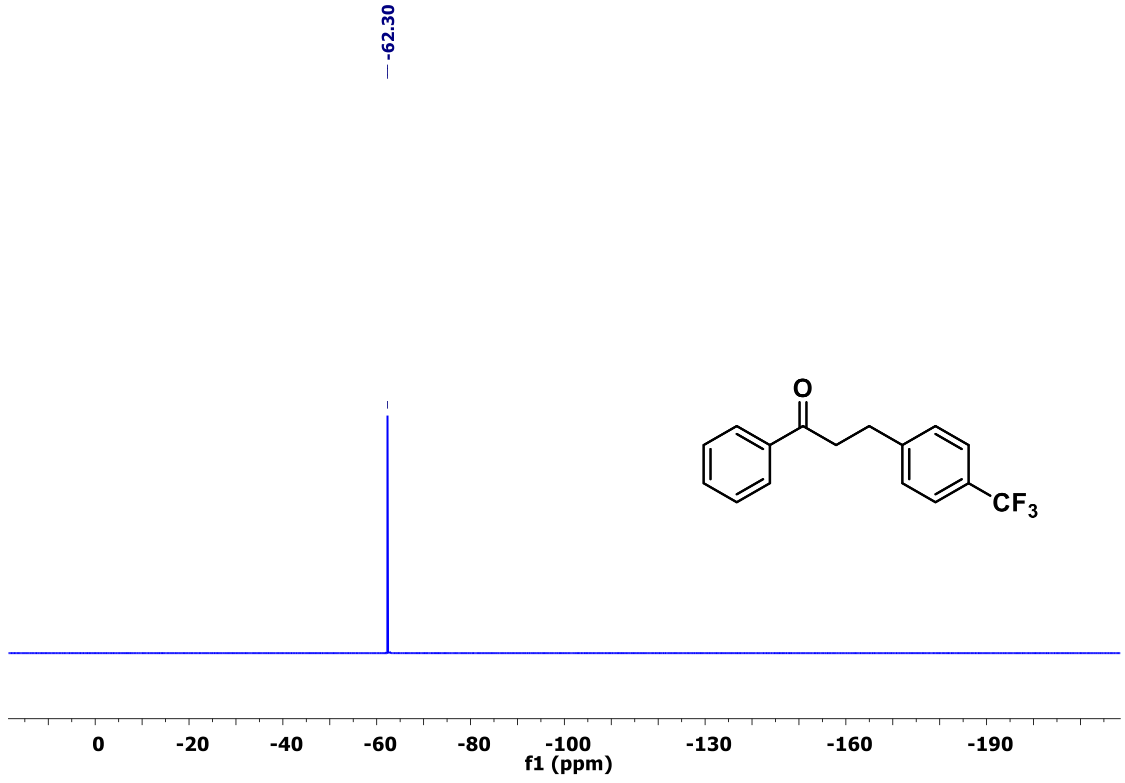

Figure S47: $^{19}F\{^{1}H\}$ NMR spectrum (377 MHz) of **6d** in $CDCl_3$

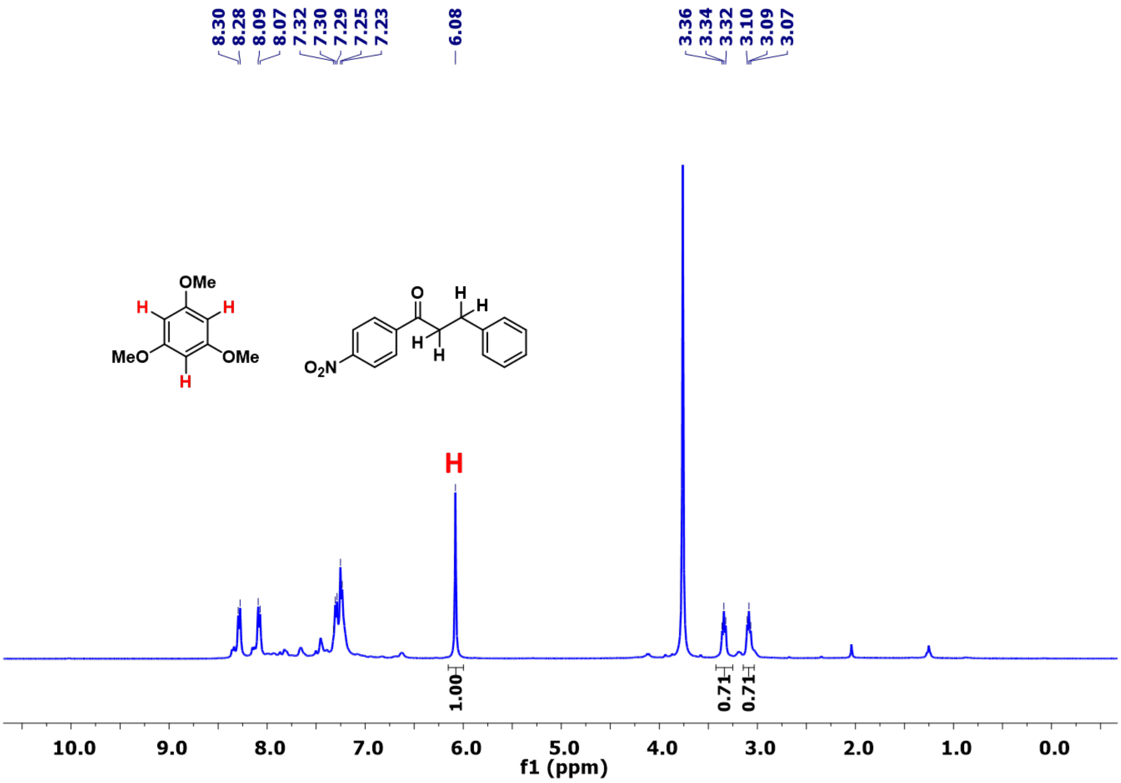

Figure S48: ^{1}H NMR spectrum (400 MHz, $CDCl_3$) of crude product mixture with 1/3 equiv. 1,3,5-trimethoxybenzene after hydrogenation of **5e**

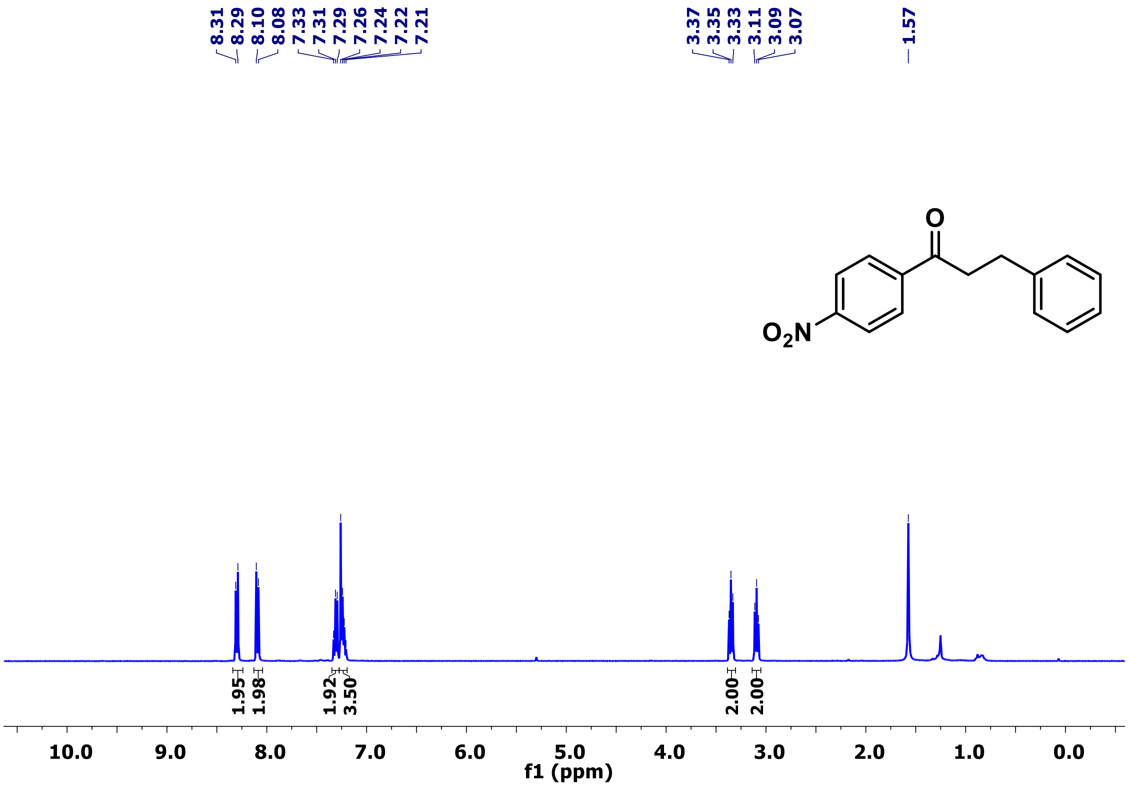

Figure S49: ^{1}H NMR spectrum (400 MHz) of **6e** in $CDCl_3$

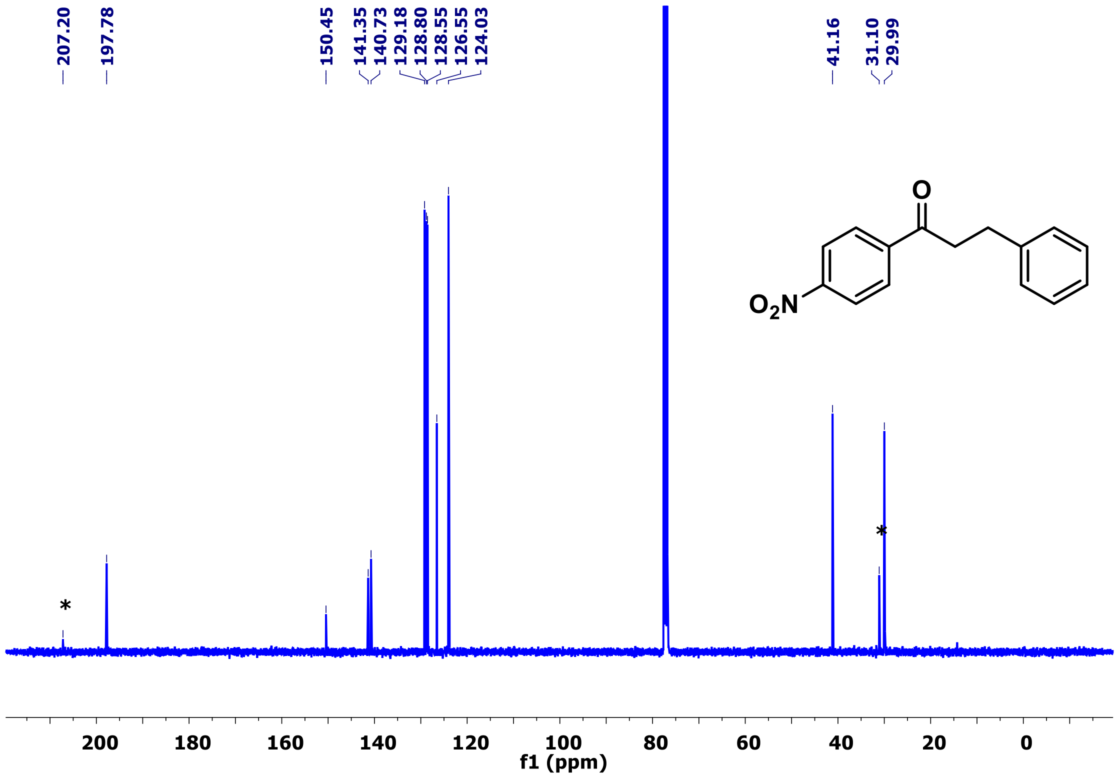

Figure S50: $^{13}C\{^1H\}$ NMR spectrum (101 MHz) of **6e** in $CDCl_3$. (* Marked peaks are from residual acetone)

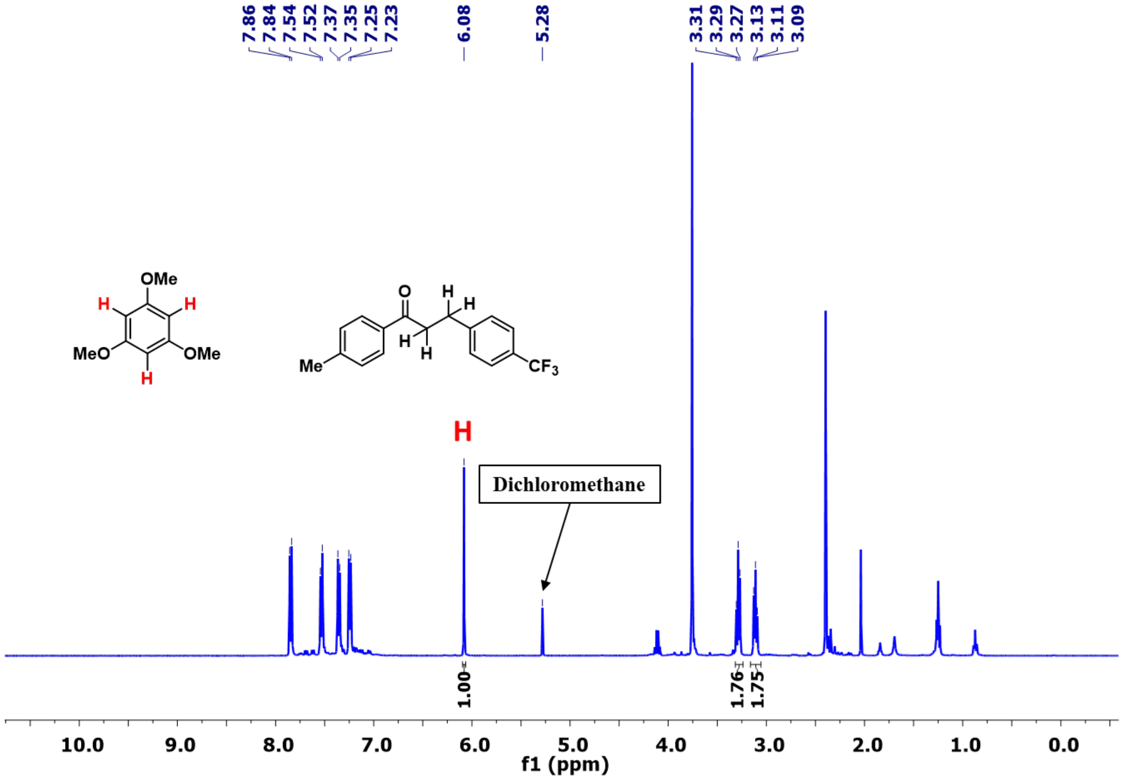

Figure S51: ^1H NMR spectrum (400 MHz, $CDCl_3$) of crude product mixture with 1/3 equiv. 1,3,5-trimethoxybenzene after hydrogenation of **5f**

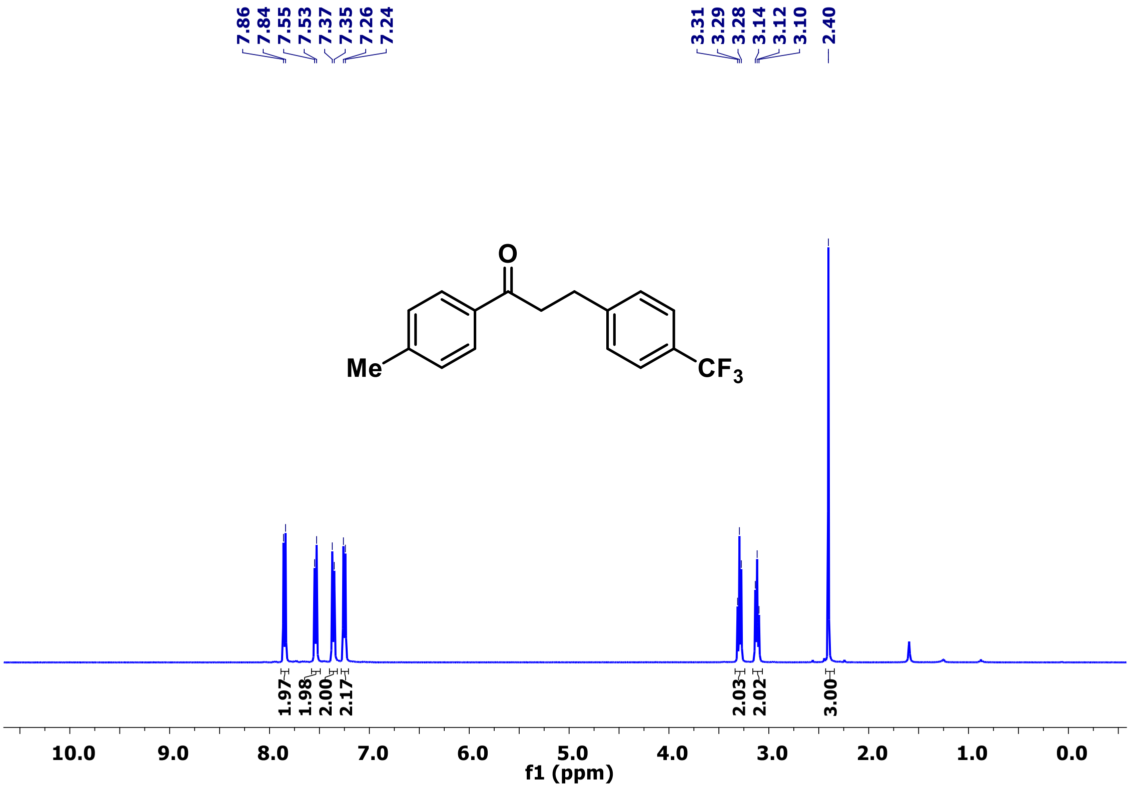

Figure S52: ^{1}H NMR spectrum (400 MHz) of **6f** in $CDCl_3$

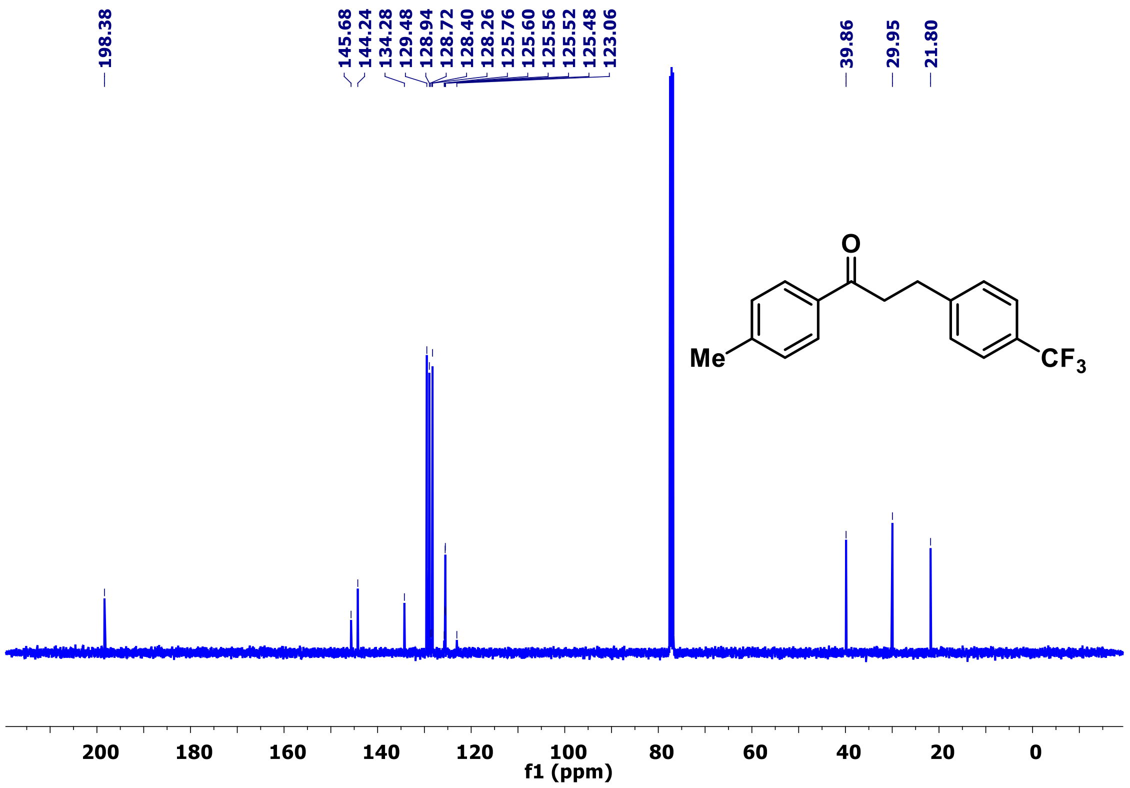

Figure S53: $^{13}C\{^1H\}$ NMR spectrum (101 MHz) of **6f** in $CDCl_3$

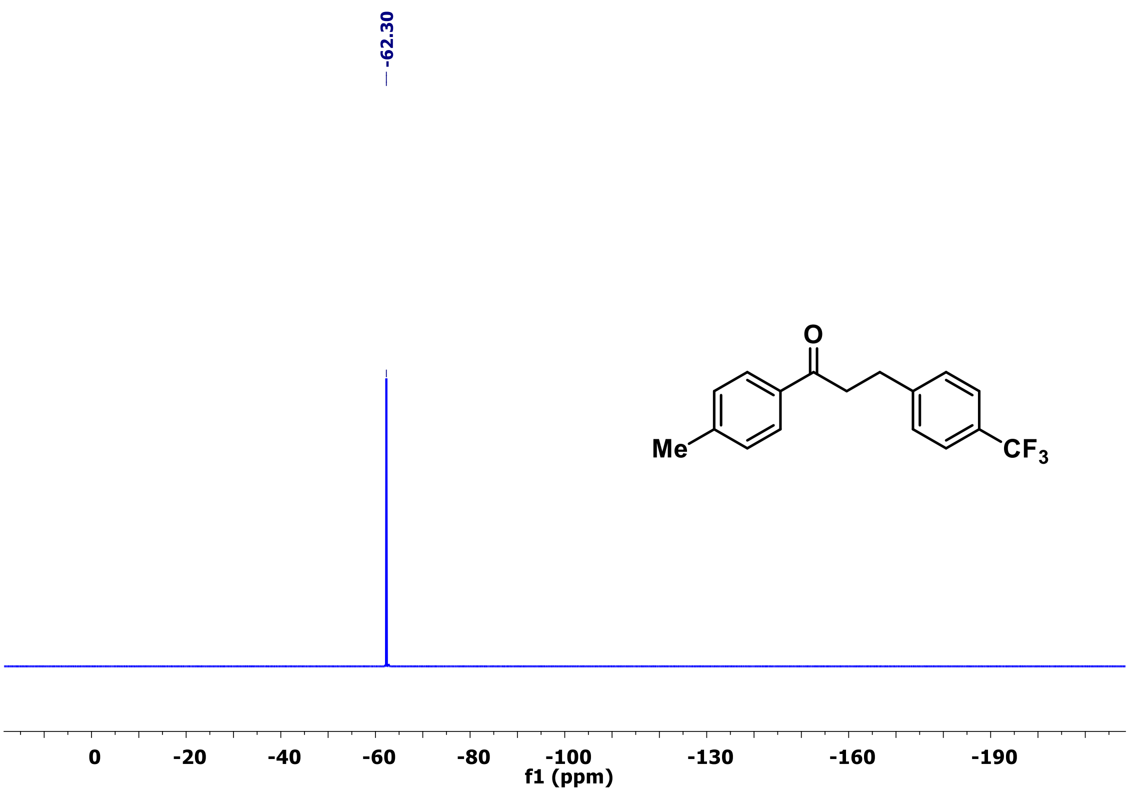

Figure S54:$^{19}F\{^{1}H\}$ NMR spectrum (377 MHz) of **6f** in $CDCl_3$

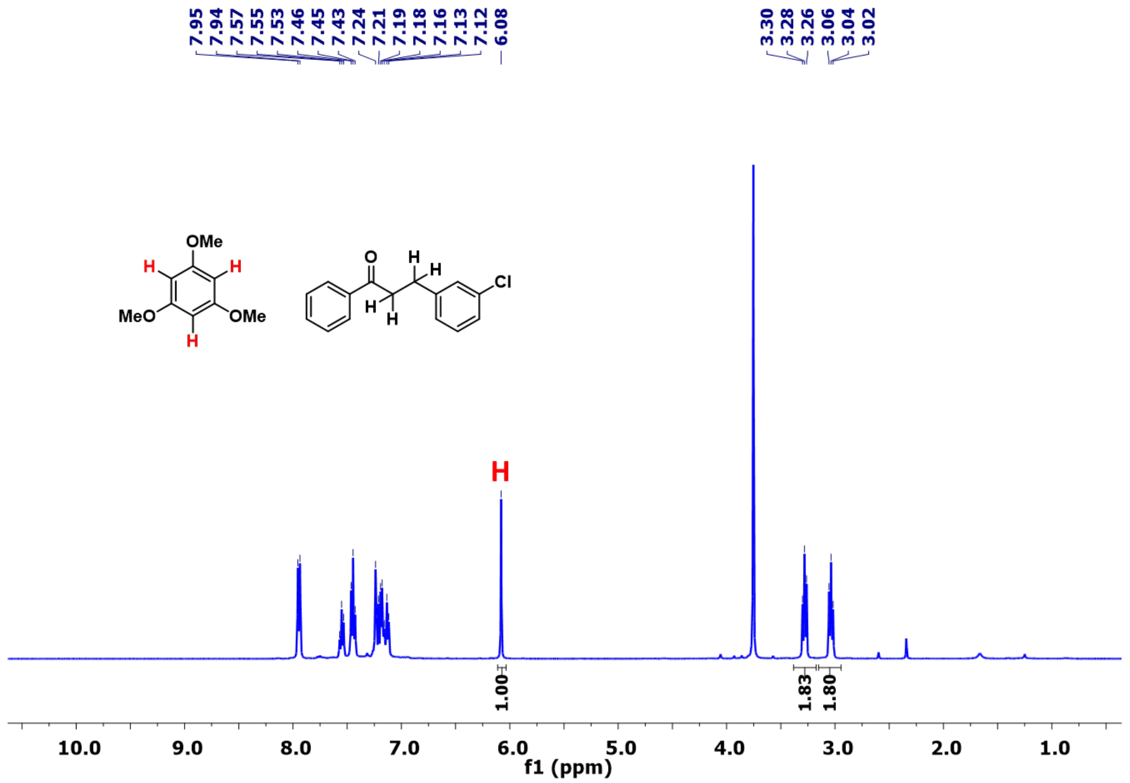

Figure S55: ^{1}H NMR spectrum (400 MHz, $CDCl_3$) of crude product mixture with 1/3 equiv. 1,3,5-trimethoxybenzene after hydrogenation of **5g**

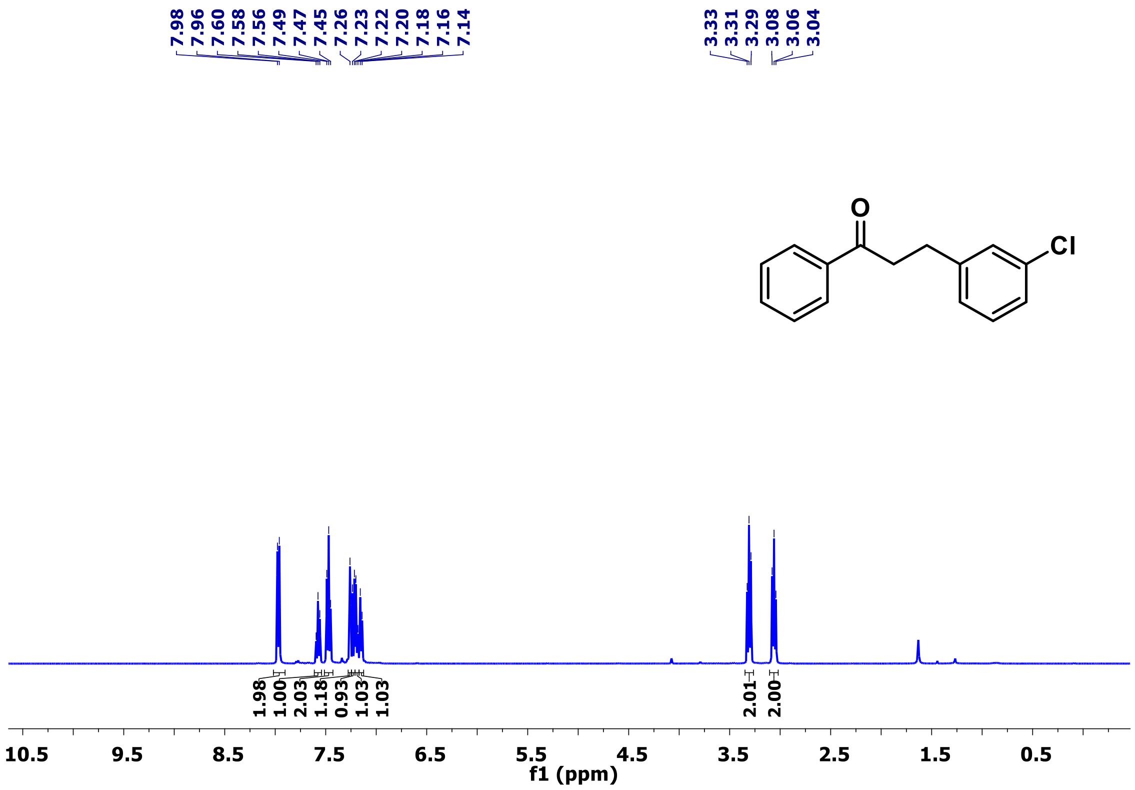

Figure S56: ^{1}H NMR spectrum (400 MHz) of **6g** in $CDCl_3$

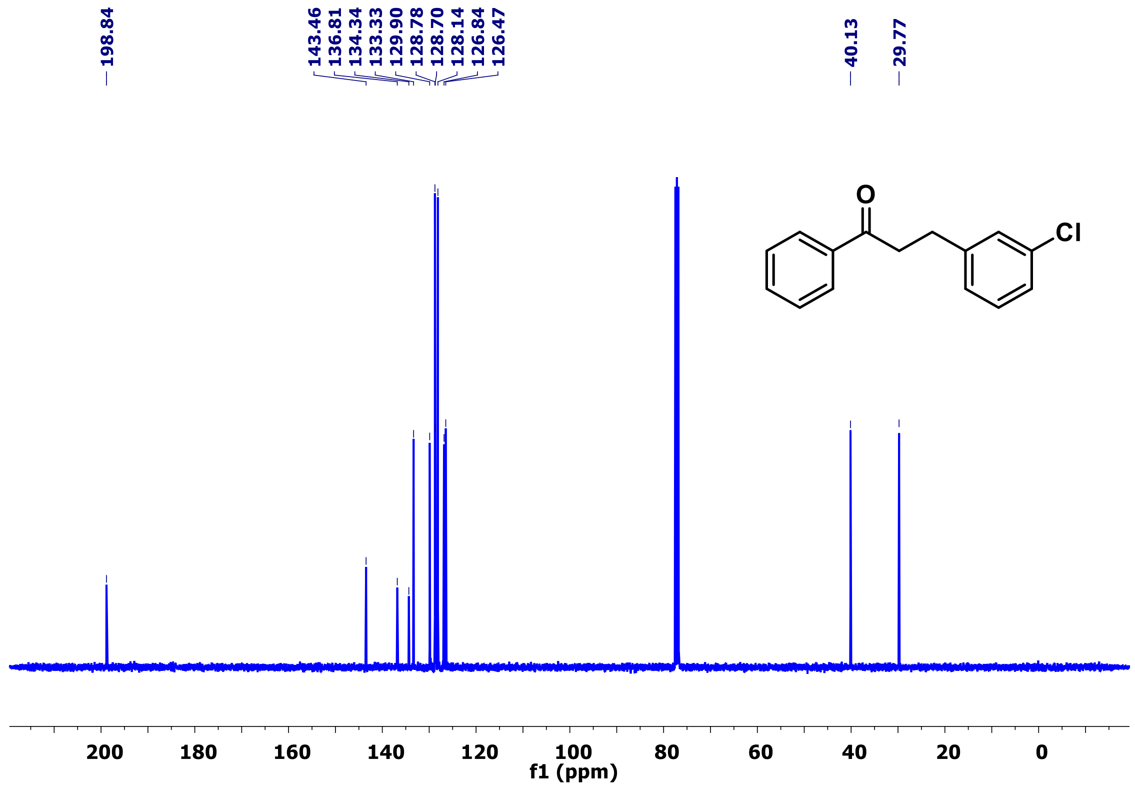

Figure S57: $^{13}C\{^{1}H\}$ NMR spectrum (101 MHz) of **6g** in $CDCl_3$

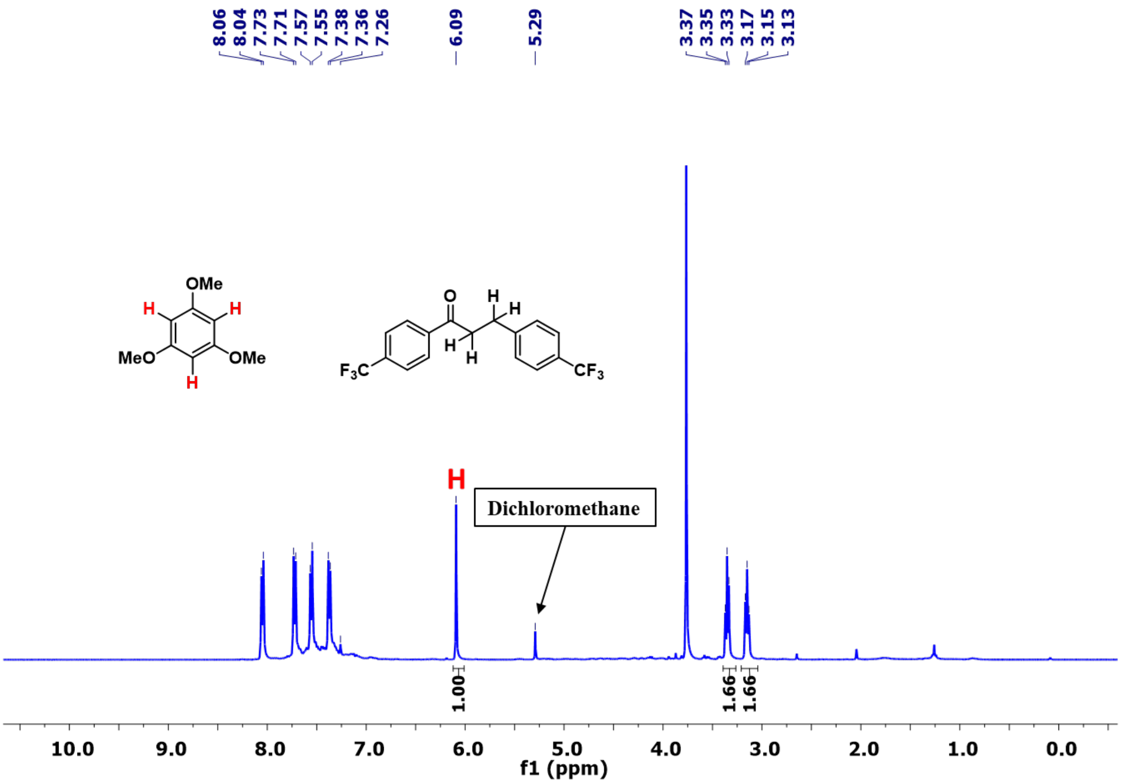

Figure S58: ^{1}H NMR spectrum (400 MHz, $CDCl_3$) of crude product mixture with 1/3 equiv. 1,3,5-trimethoxybenzene after hydrogenation of **5h**

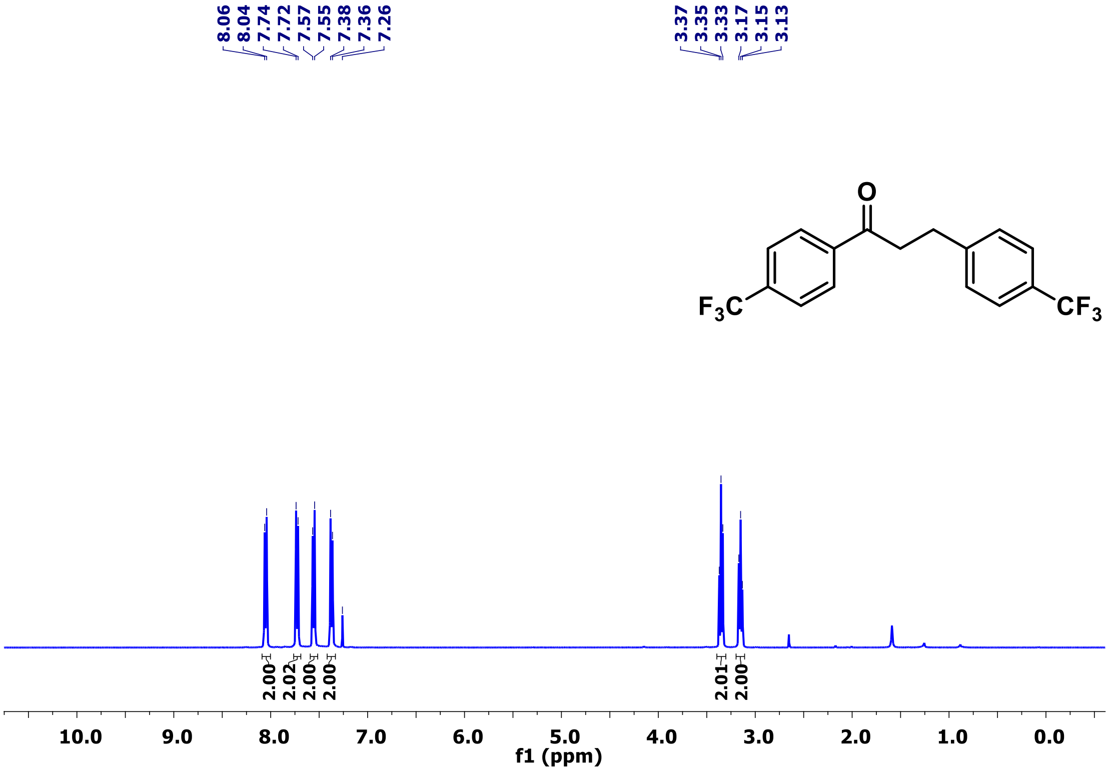

Figure S59: ^{1}H NMR spectrum (400 MHz) of **6h** in $CDCl_3$

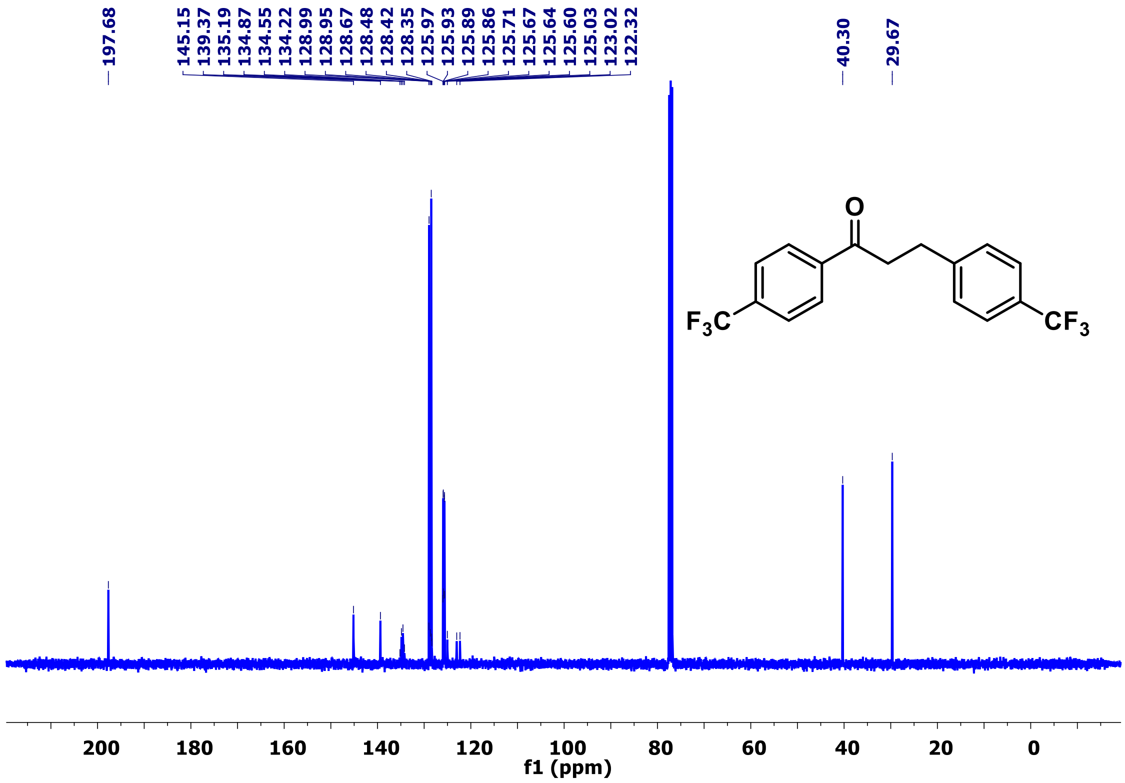

Figure S60: $^{13}C\{^{1}H\}$ NMR spectrum (101 MHz) of **6h** in $CDCl_3$

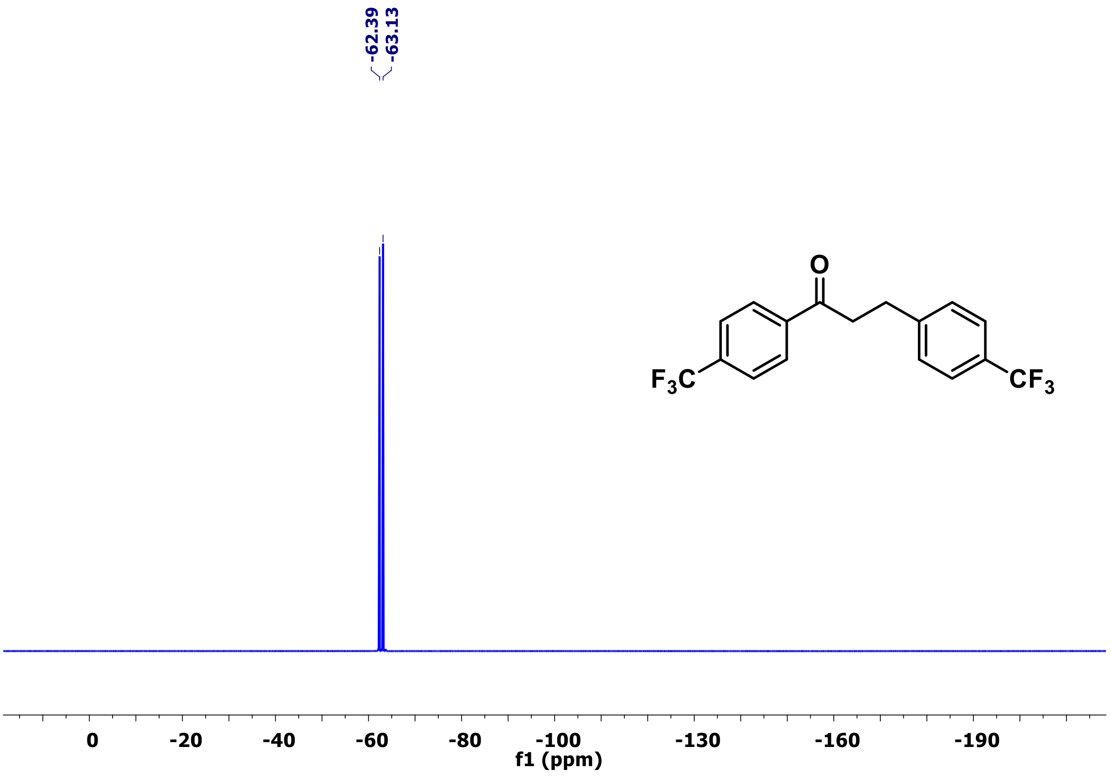

Figure S61: $^{19}F\{^{1}H\}$ NMR spectrum (377 MHz) of **6h** in $CDCl_3$

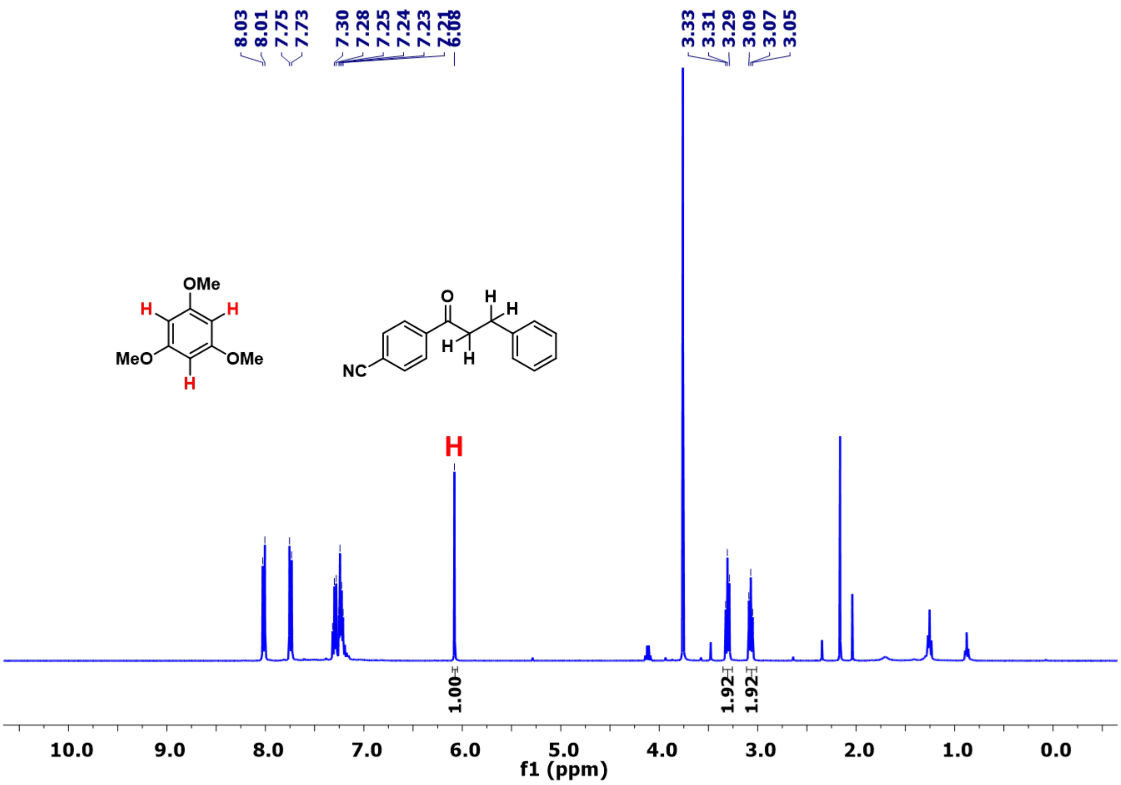

Figure S62: ^{1}H NMR spectrum (400 MHz, $CDCl_3$) of crude product mixture with 1/3 equiv. 1,3,5-trimethoxybenzene after hydrogenation of **5i**

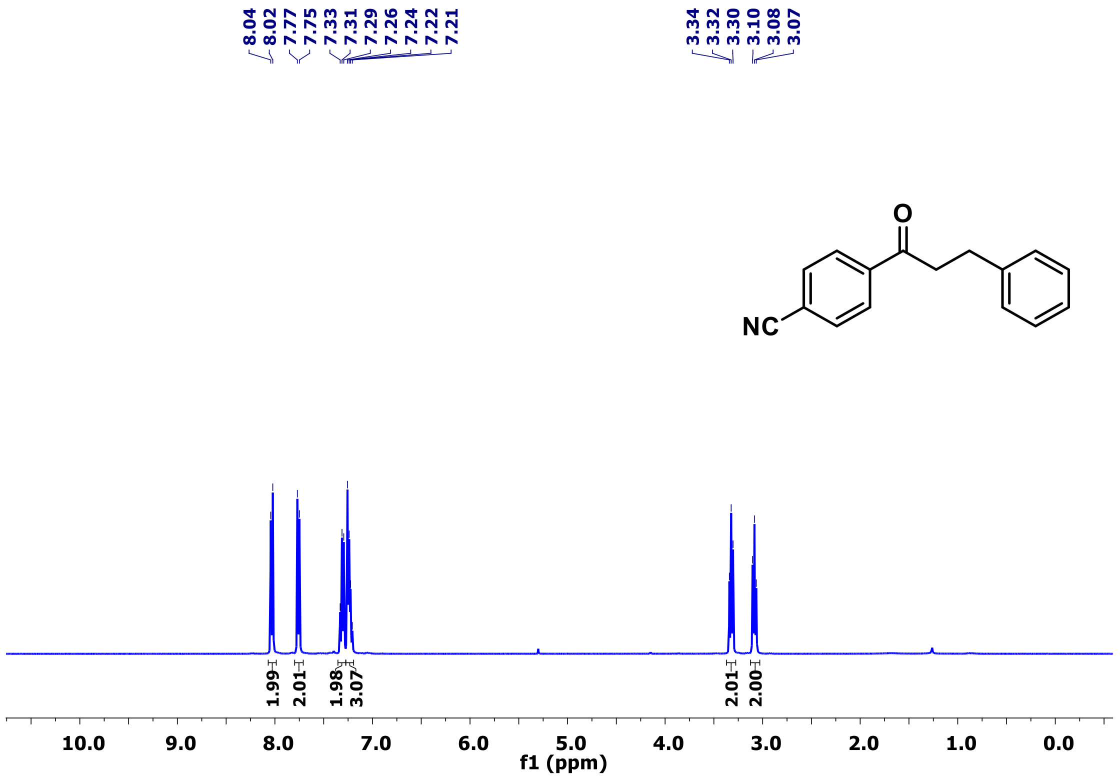

Figure S63: ^{1}H NMR spectrum (400 MHz) of **6i** in $CDCl_3$

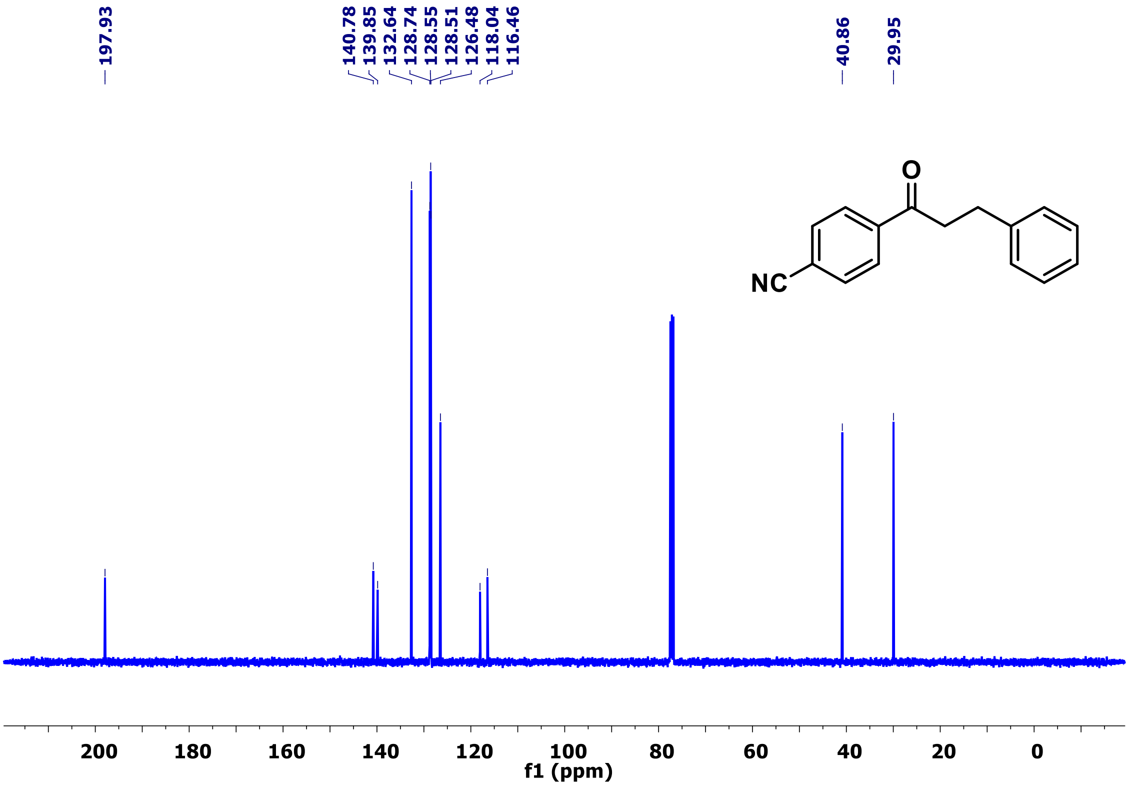

Figure S64: $^{13}C\{^{1}H\}$ NMR spectrum (101 MHz) of **6i** in $CDCl_3$

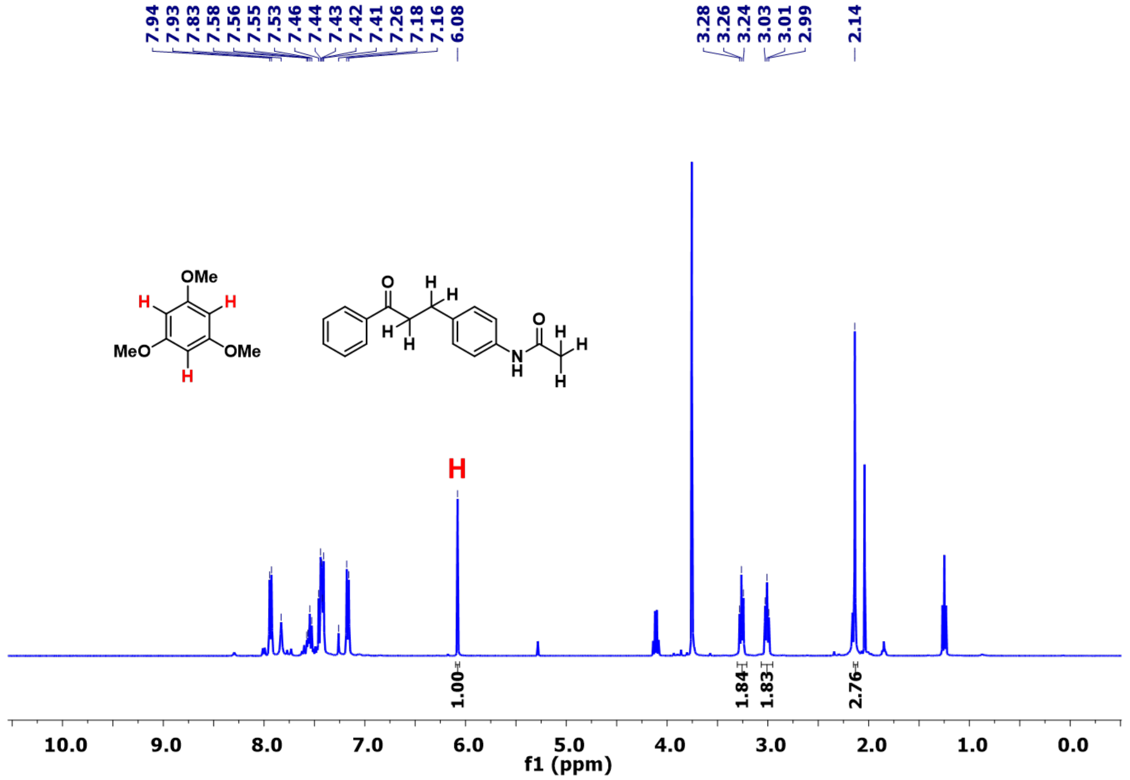

Figure S65: ^{1}H NMR spectrum (400 MHz, $CDCl_3$) of crude product mixture with 1/3 equiv. 1,3,5-trimethoxybenzene after hydrogenation of **5j**

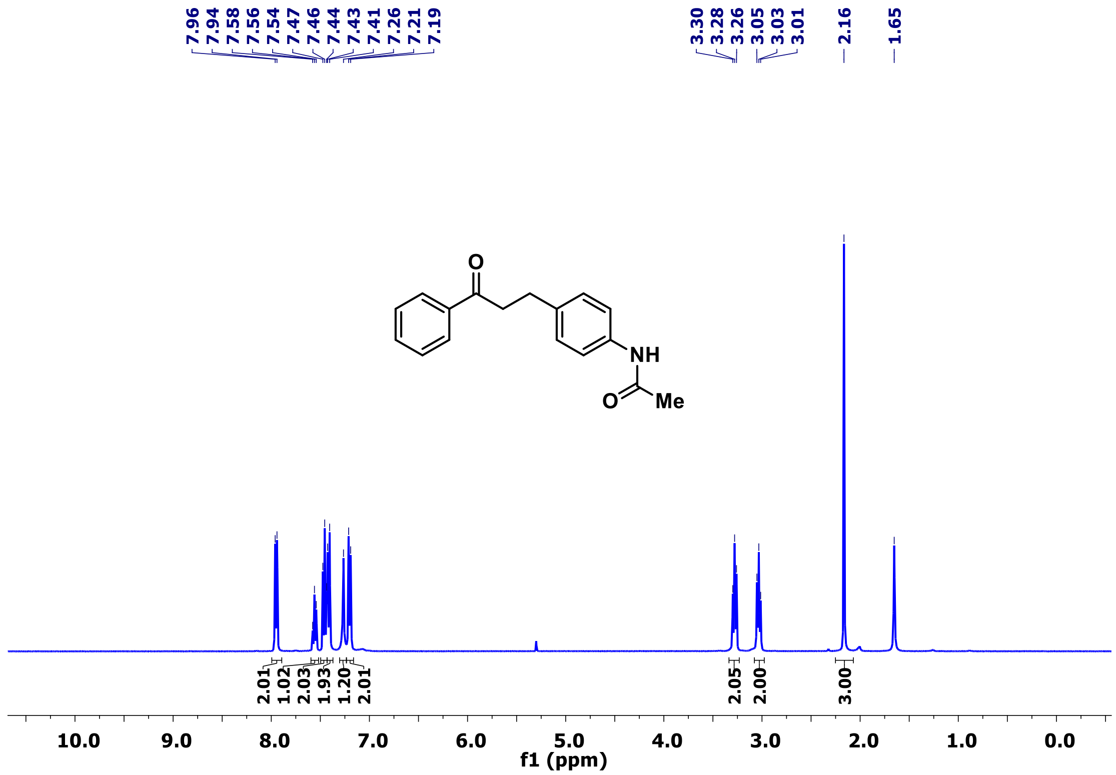

Figure S66: ^{1}H NMR spectrum (400 MHz) of **6j** in $CDCl_3$

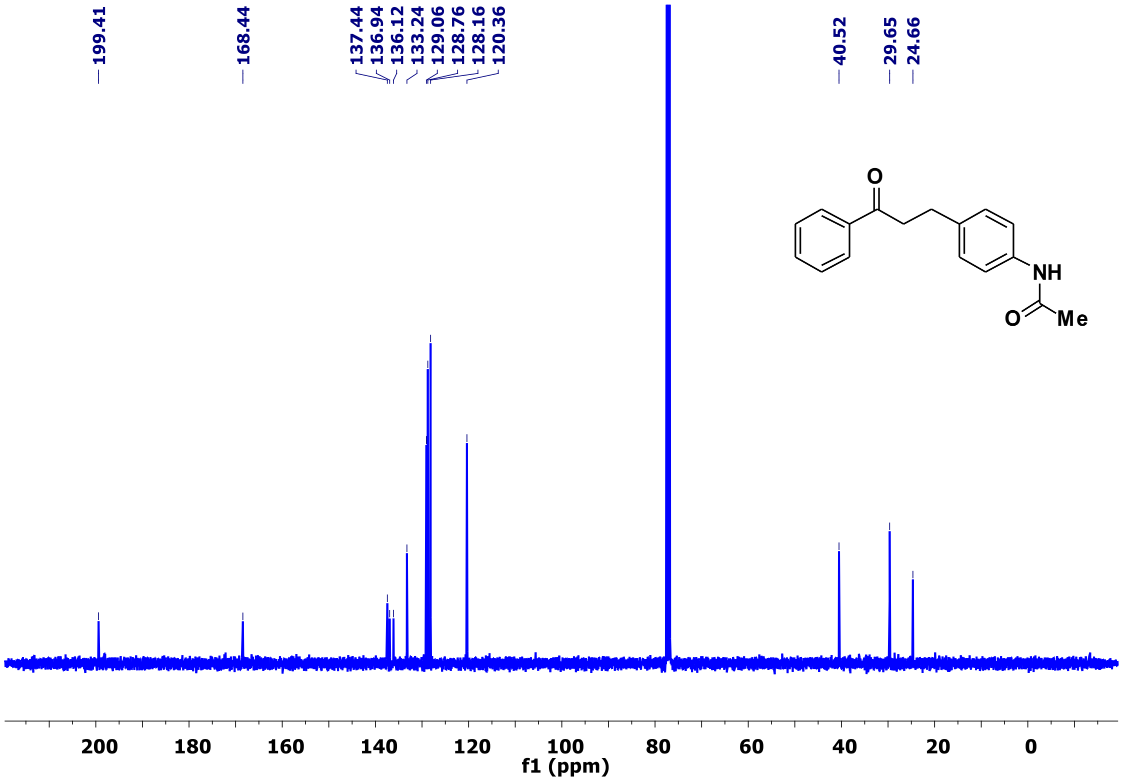

Figure S67: $^{13}C\{^{1}H\}$ NMR spectrum (101 MHz) of **6j** in $CDCl_3$

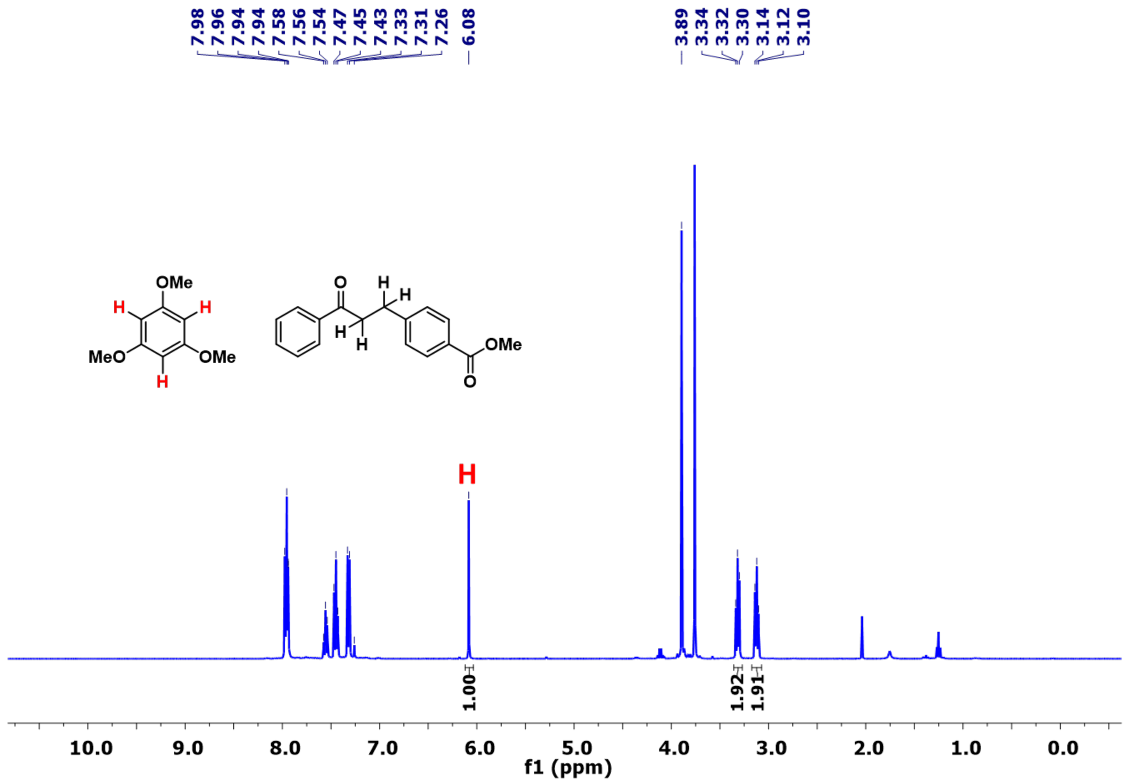

Figure S68: ^{1}H NMR spectrum (400 MHz, $CDCl_3$) of crude product mixture with 1/3 equiv. 1,3,5-trimethoxybenzene after hydrogenation of **5k**

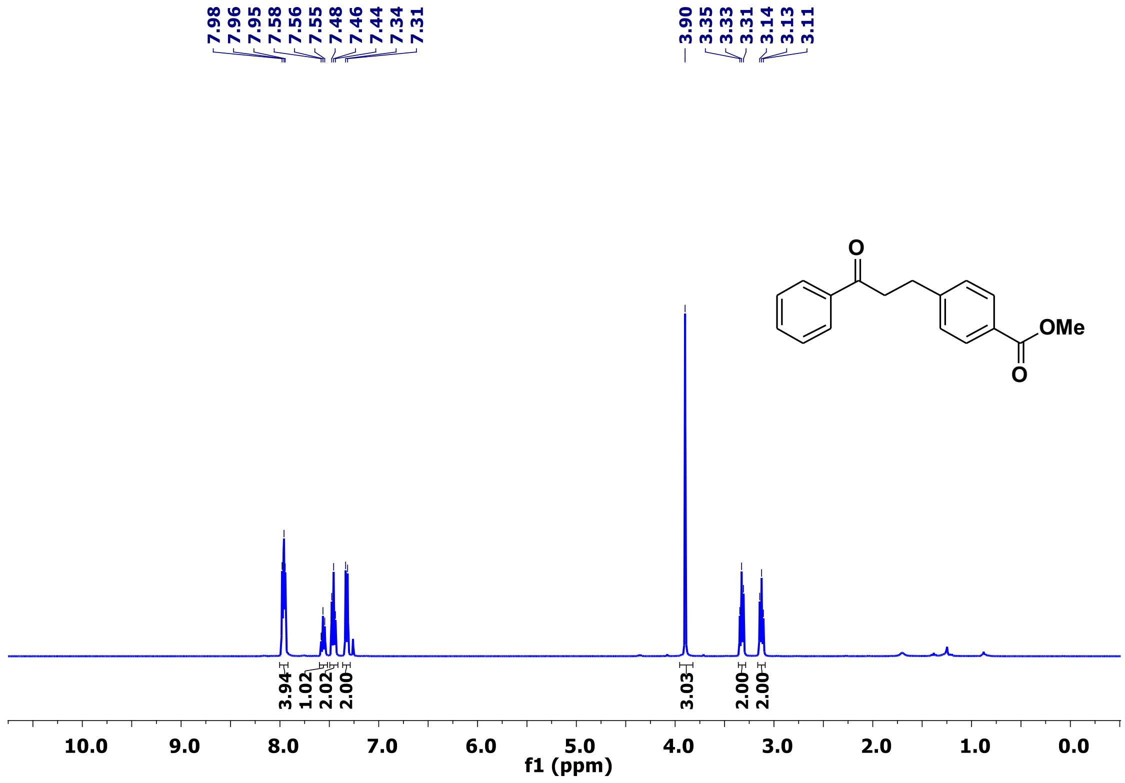

Figure S69: ^{1}H NMR spectrum (400 MHz) of **6k** in $CDCl_3$

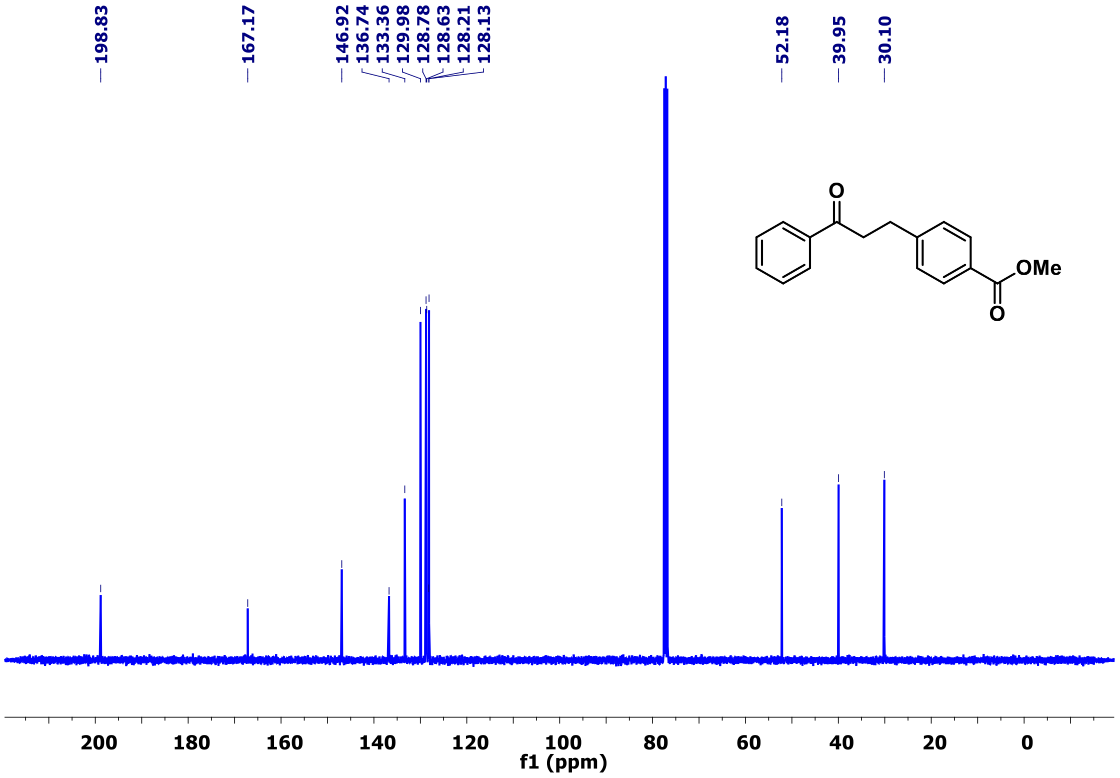

Figure S70: $^{13}C\{^{1}H\}$ NMR spectrum (101 MHz) of **6k** in $CDCl_3$

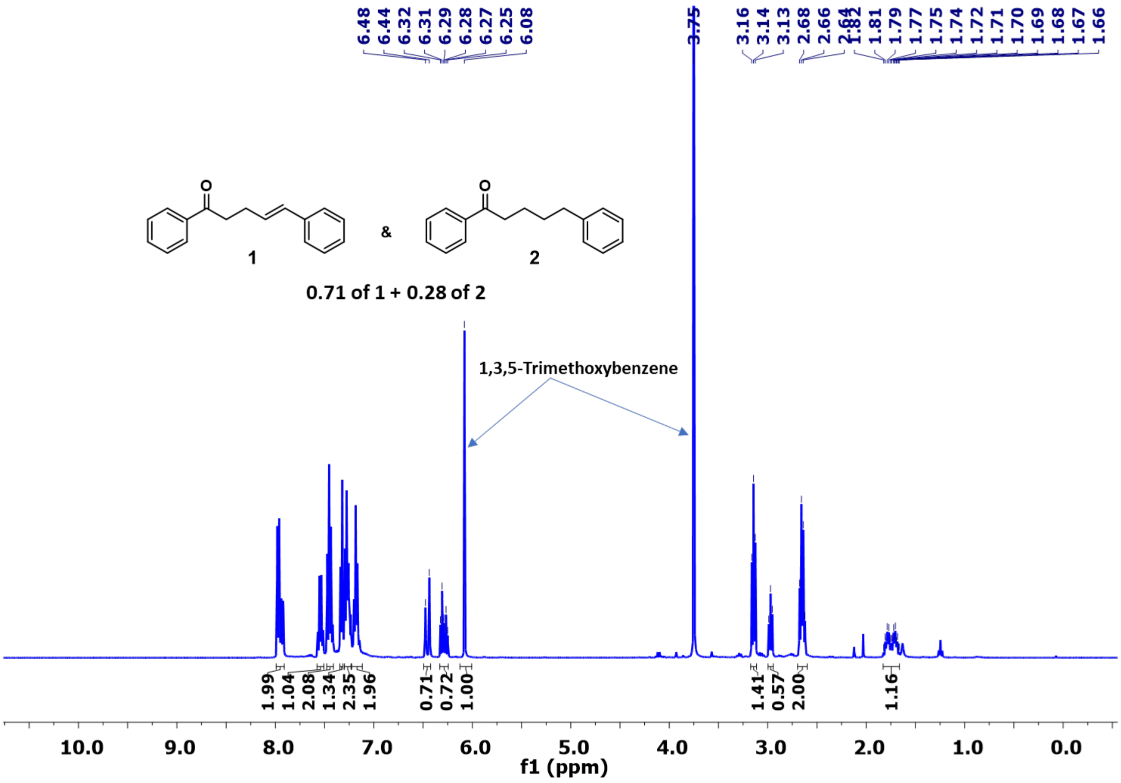

Figure S71: ^{1}H NMR spectrum (400 MHz, $CDCl_3$) of crude product mixture with 1/3 equiv. 1,3,5-trimethoxybenzene after hydrogenation of **5l**

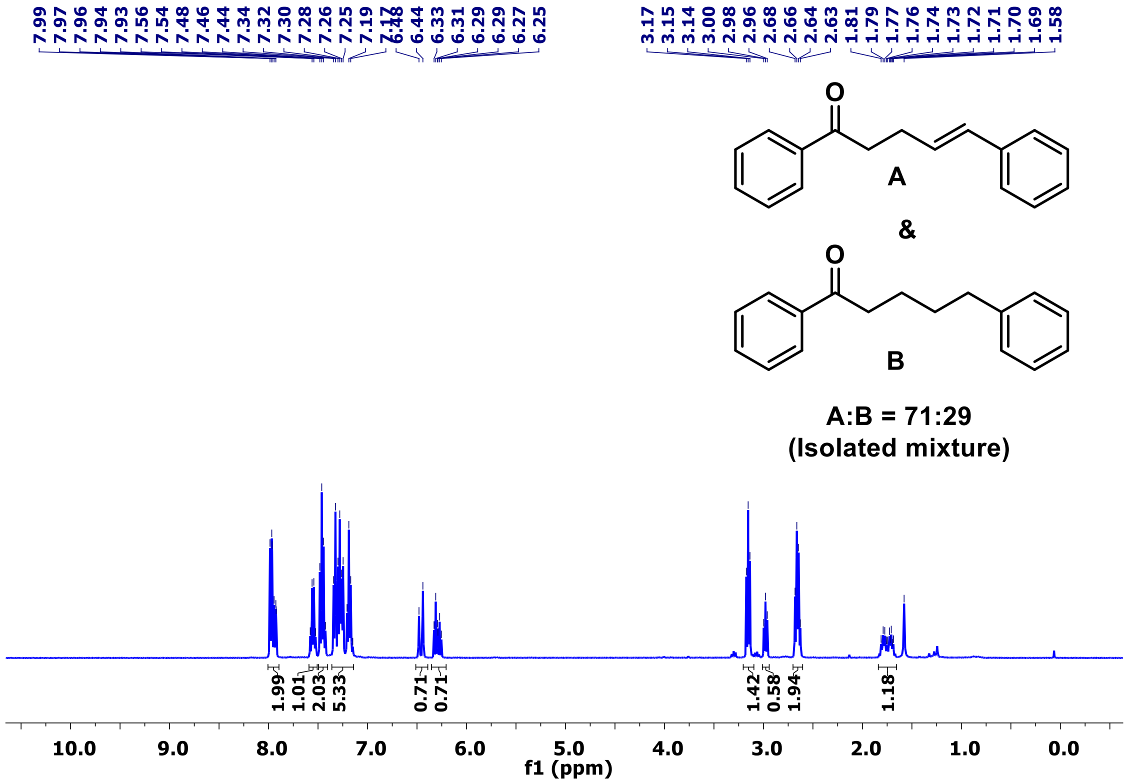

Figure S72: ^{1}H NMR spectrum (400 MHz) of isolated product mixture **6l** in $CDCl_3$

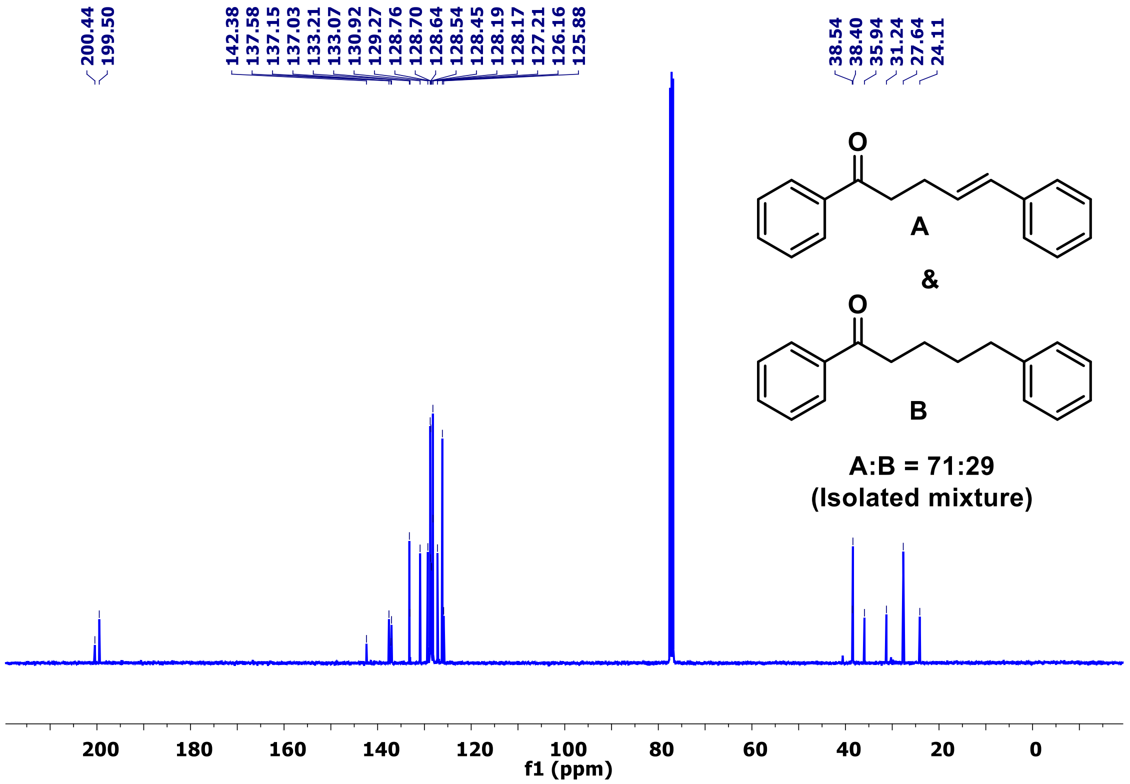

Figure S73: $^{13}C\{^{1}H\}$ NMR spectrum (101 MHz) of isolated product mixture **6l** in $CDCl_3$

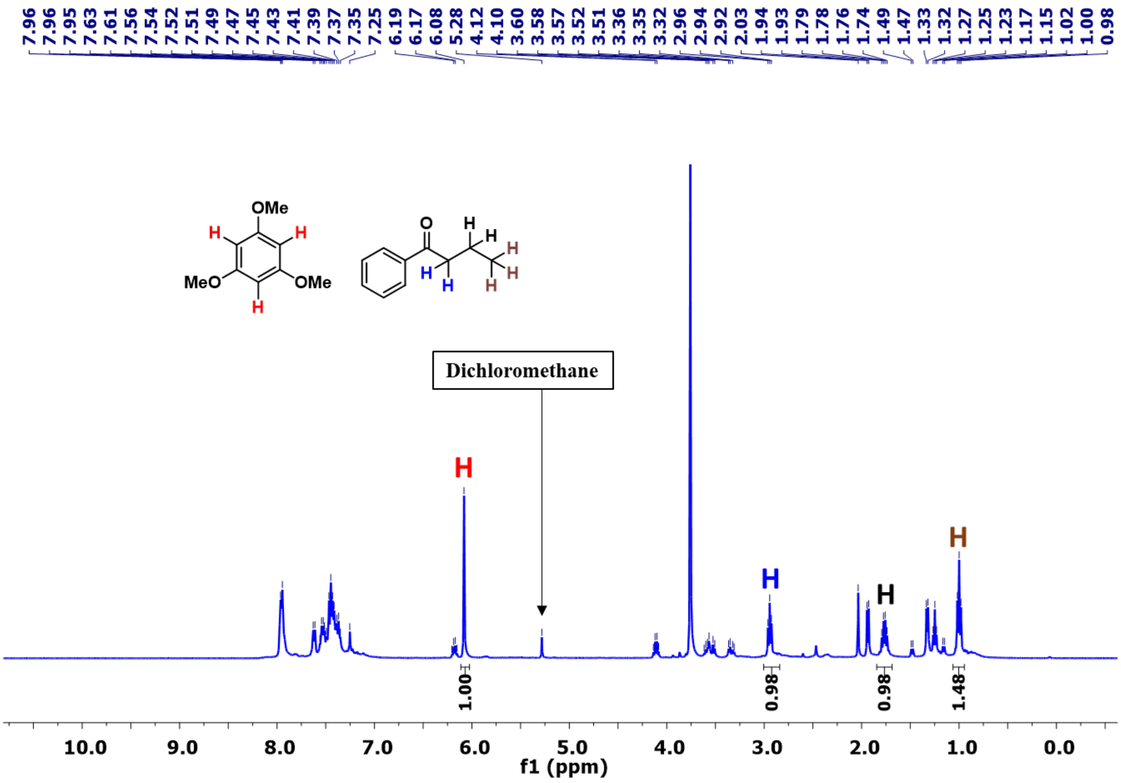

Figure S74: ^{1}H NMR spectrum (400 MHz, $CDCl_3$) of crude product mixture with 1/3 equiv. 1,3,5-trimethoxybenzene after hydrogenation of **5m**

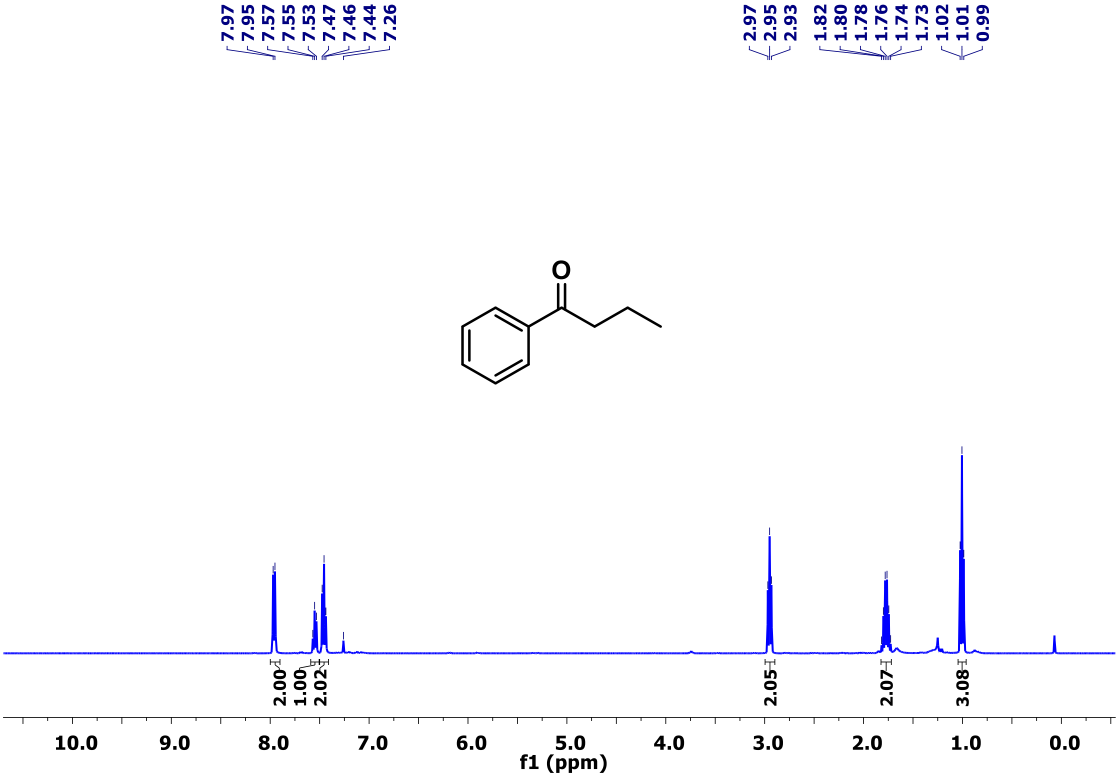

Figure S75: ^{1}H NMR spectrum (400 MHz) of **6m** in $CDCl_3$

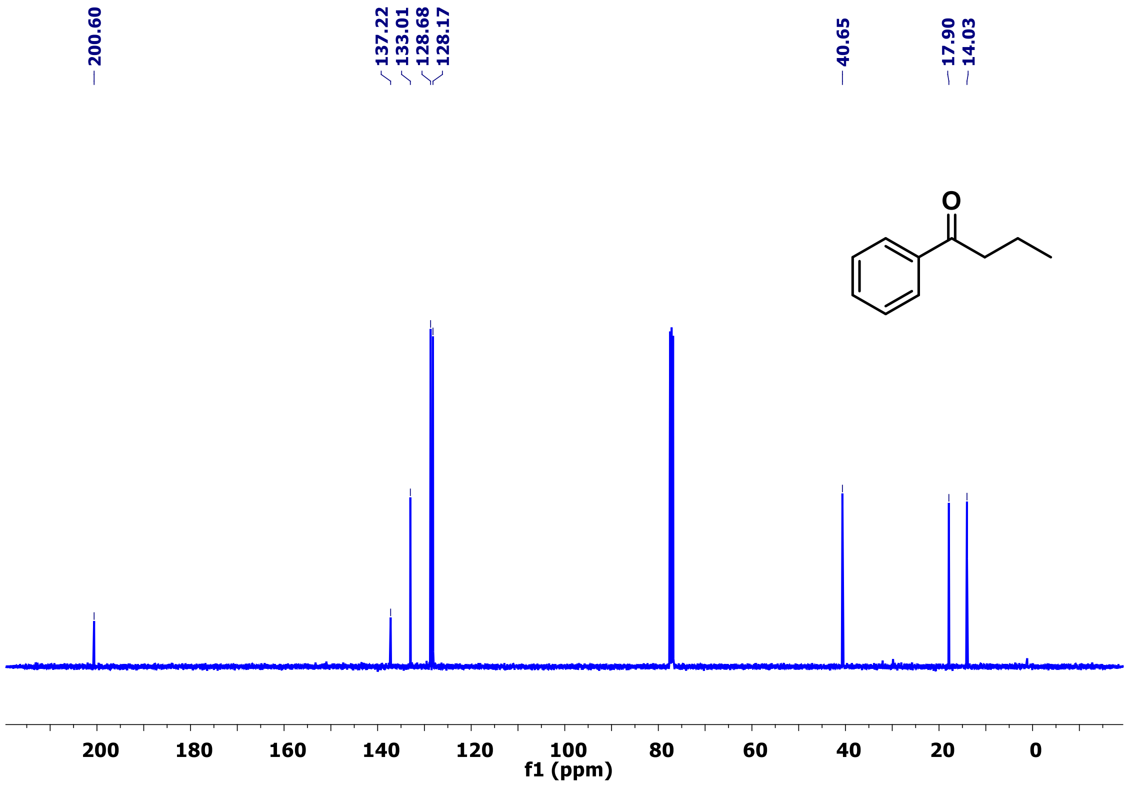

Figure S76: $^{13}C\{^{1}H\}$ NMR spectrum (101 MHz) of **6m** in $CDCl_3$

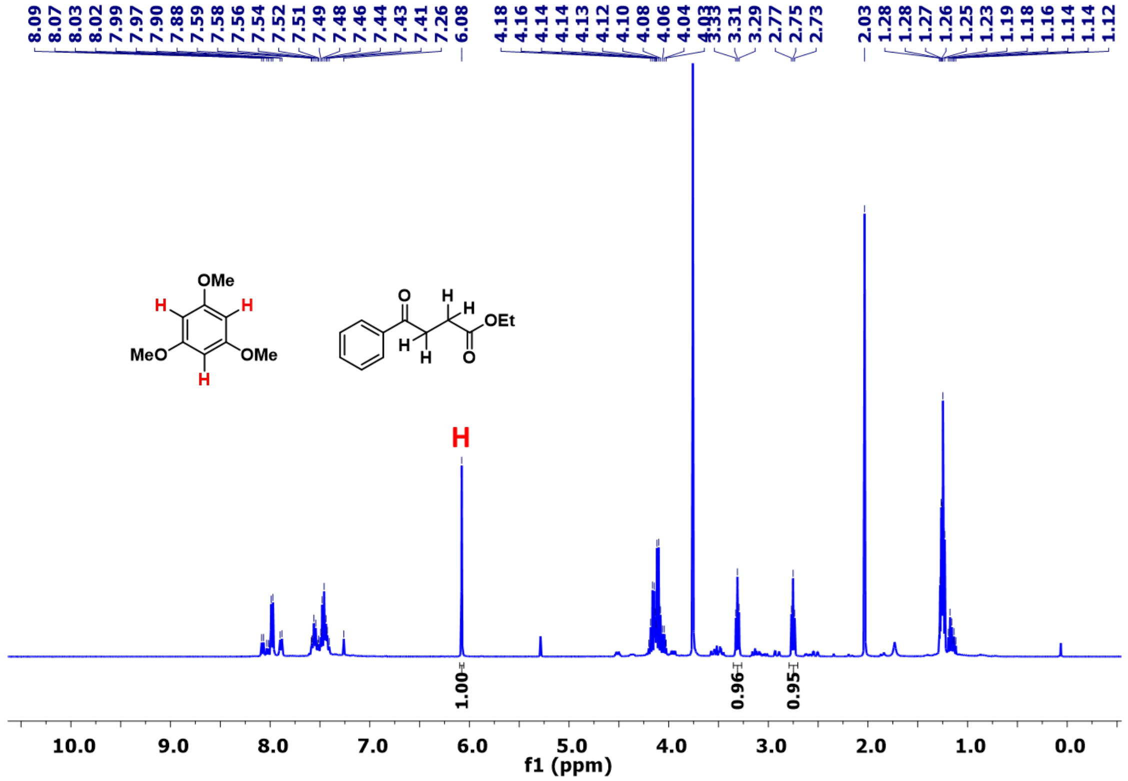

Figure S77: ^{1}H NMR spectrum (400 MHz, $CDCl_3$) of crude product mixture with 1/3 equiv. 1,3,5-trimethoxybenzene after hydrogenation of **5n**

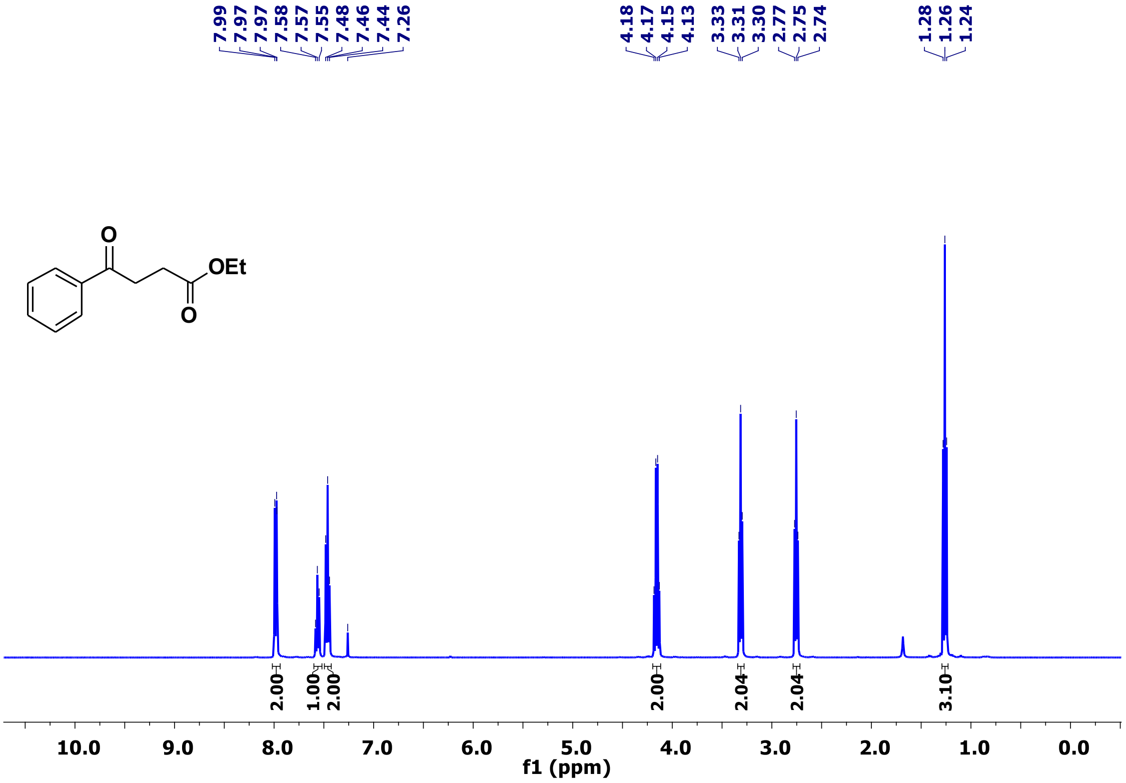

Figure S78: ^{1}H NMR spectrum (400 MHz) of **6n** in $CDCl_3$

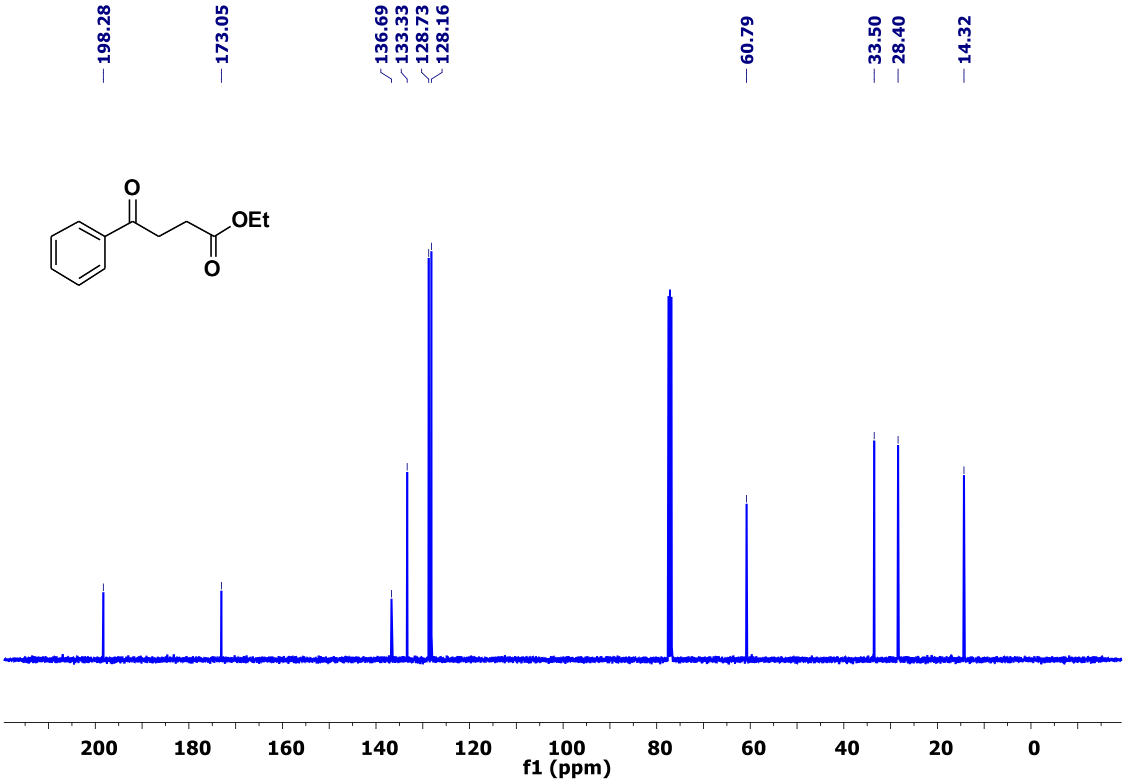

Figure S79: $^{13}C\{^{1}H\}$ NMR spectrum (101 MHz) of **6n** in $CDCl_3$

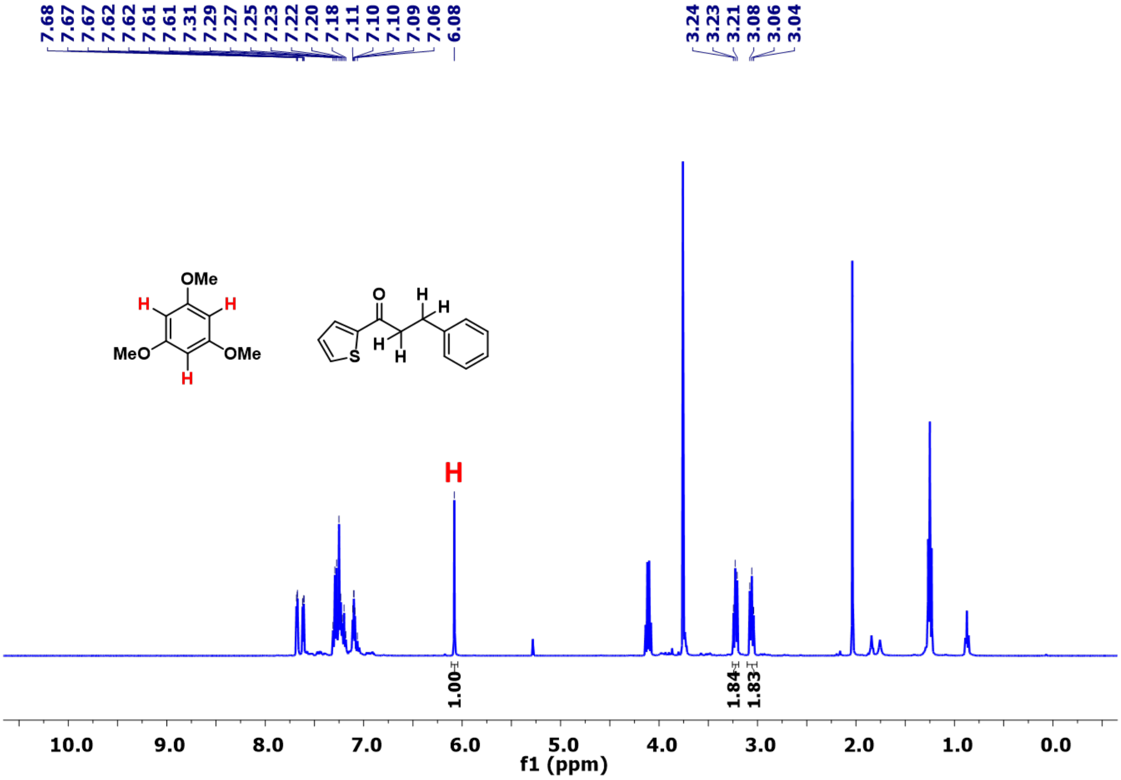

Figure S80: ^{1}H NMR spectrum (400 MHz, $CDCl_3$) of crude product mixture with 1/3 equiv. 1,3,5-trimethoxybenzene after hydrogenation of **5o**

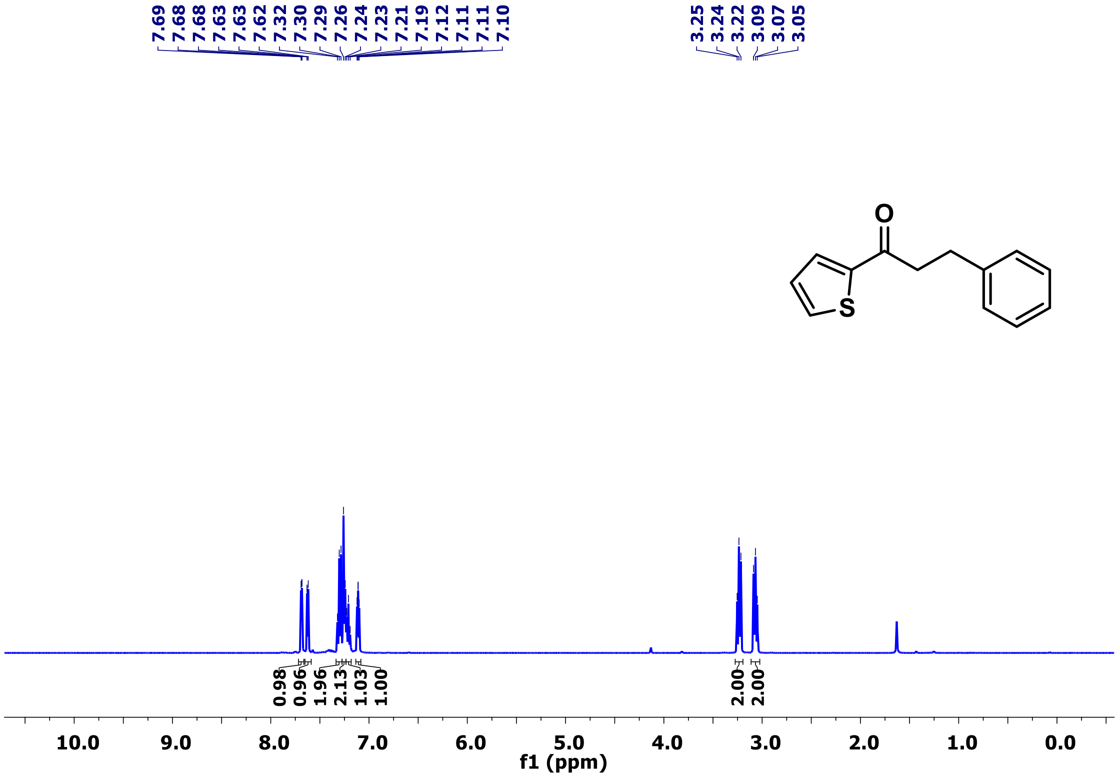

Figure S81: ^{1}H NMR spectrum (400 MHz) of **6o** in $CDCl_3$

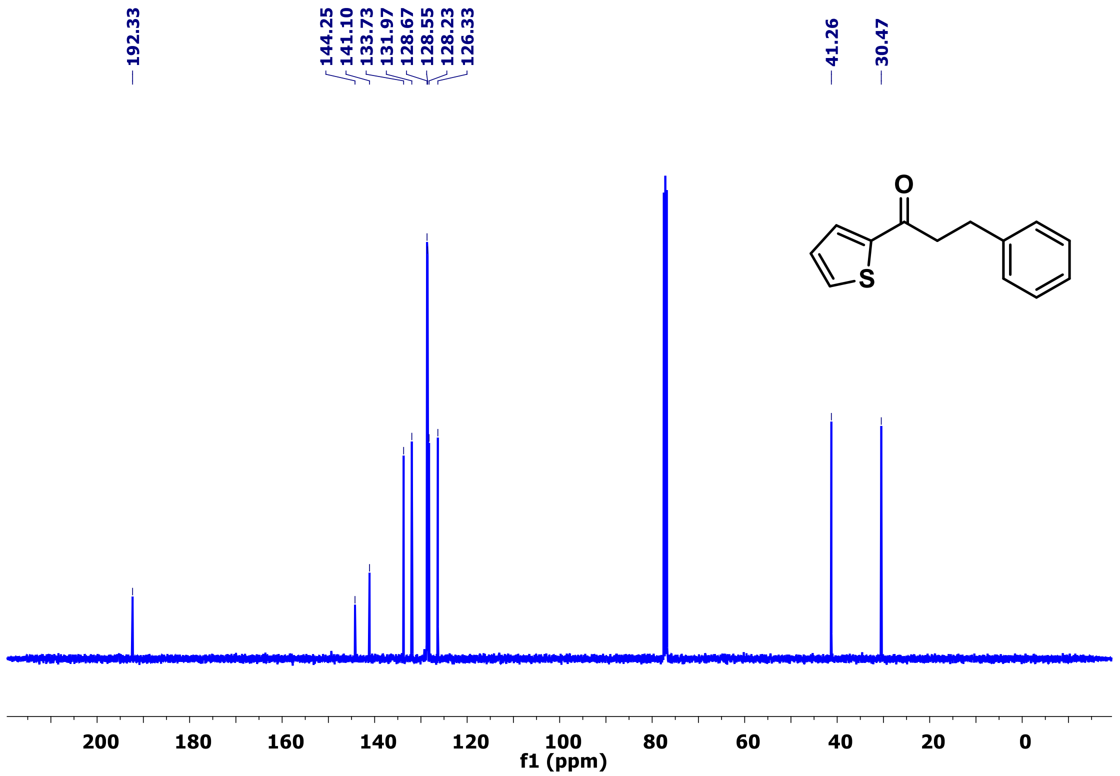

Figure S82: $^{13}C\{^1H\}$ NMR spectrum (101 MHz) of **6o** in $CDCl_3$

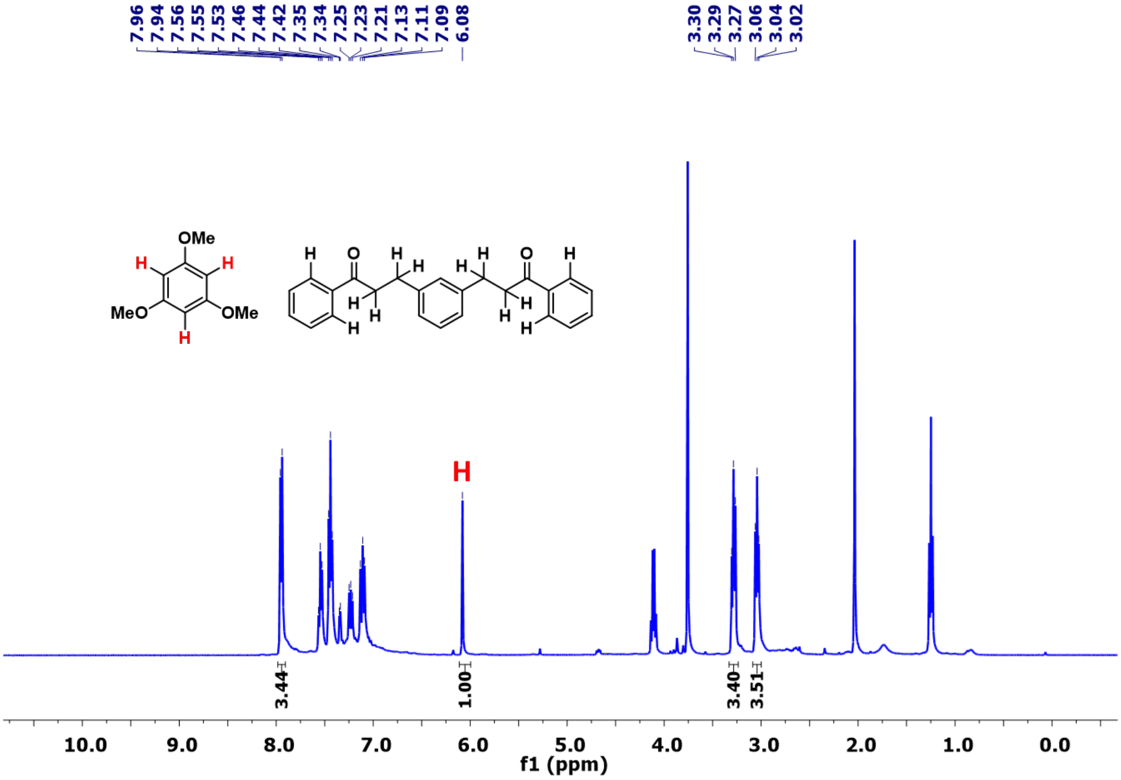

Figure S83: ^1H NMR spectrum (400 MHz, $CDCl_3$) of crude product mixture with 1/3 equiv. 1,3,5-trimethoxybenzene after hydrogenation of **5p**

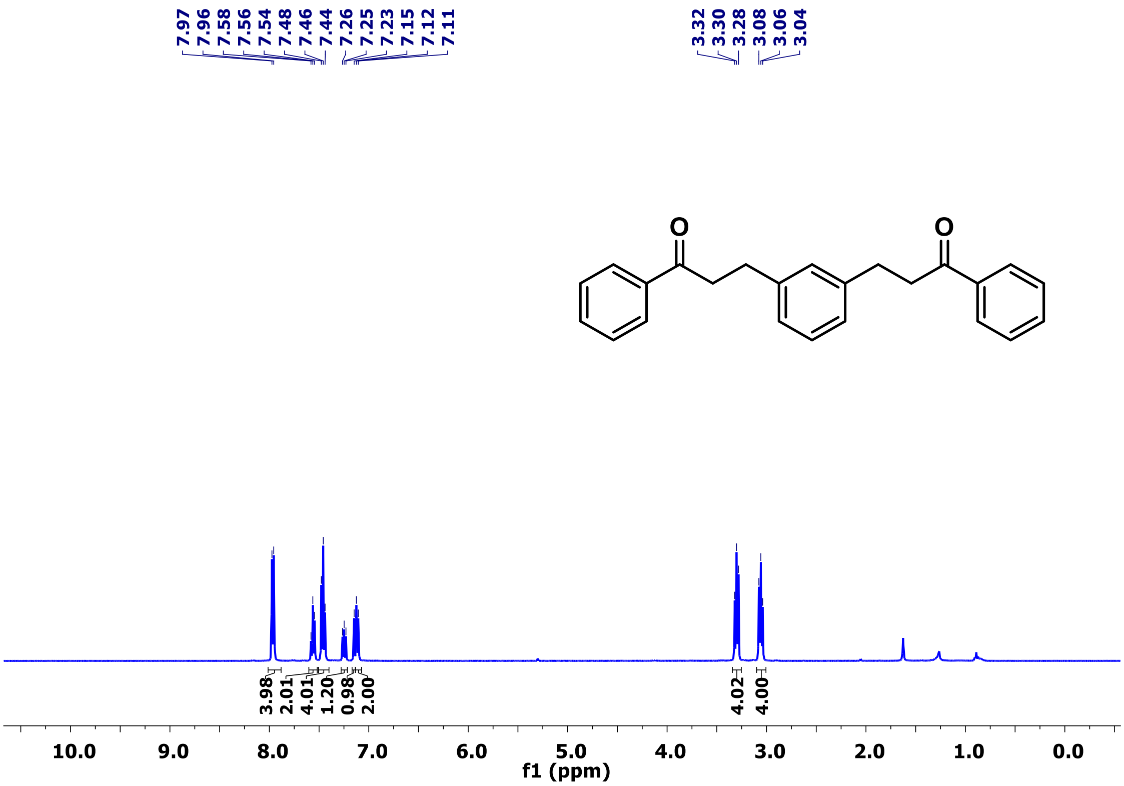

Figure S84: ^{1}H NMR spectrum (400 MHz) of **6p** in $CDCl_3$

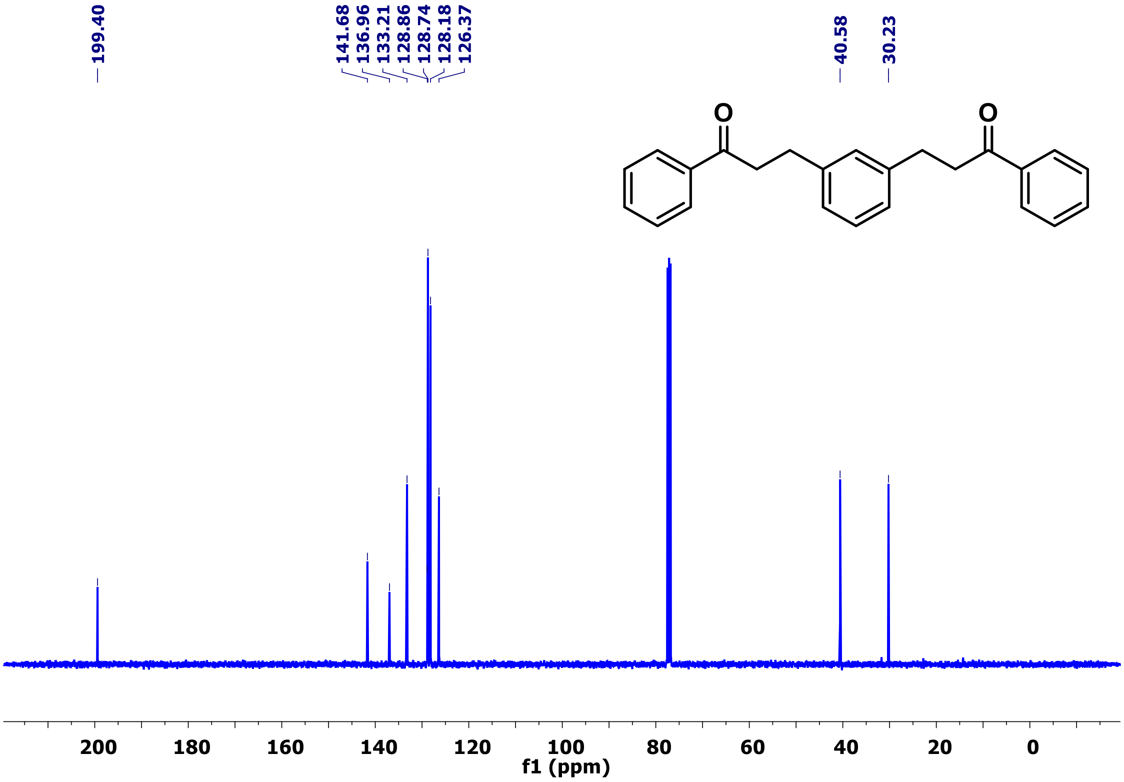

Figure S85: $^{13}C\{^{1}H\}$ NMR spectrum (101 MHz) of **6p** in $CDCl_3$

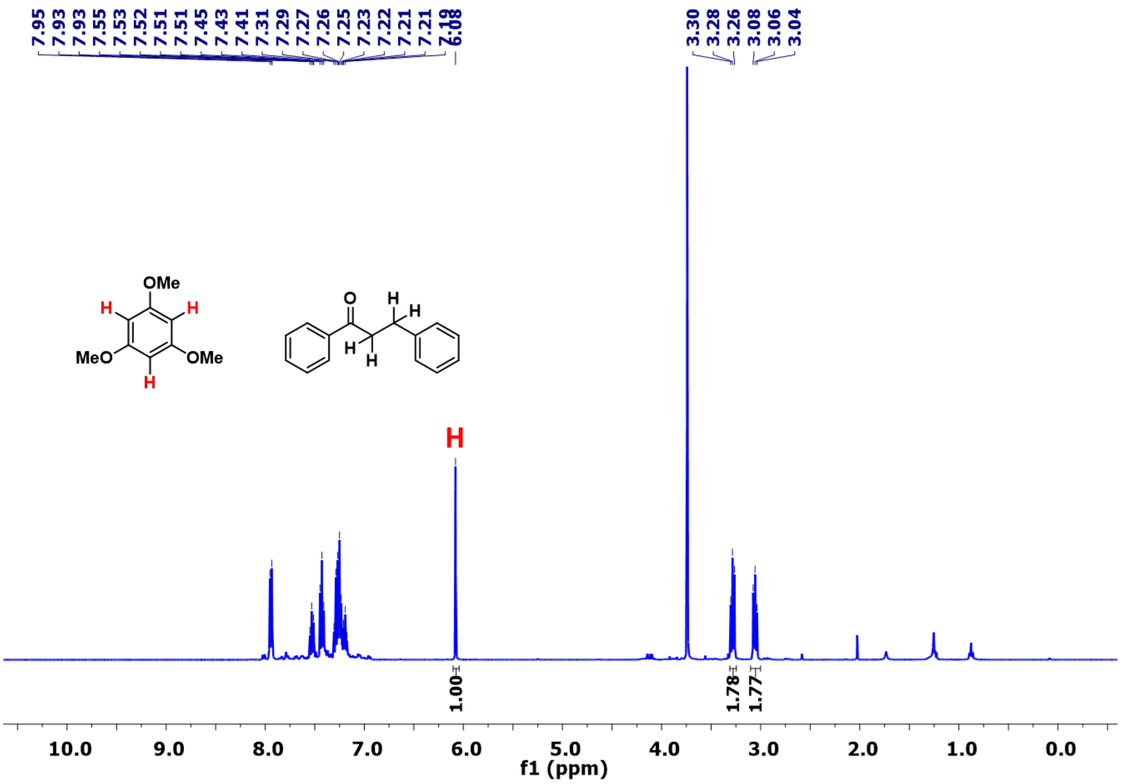

Figure S86: ^{1}H NMR spectrum (400 MHz, $CDCl_3$) of crude product mixture with 1/3 equiv. 1,3,5-trimethoxybenzene after hydrogenation of **5q**

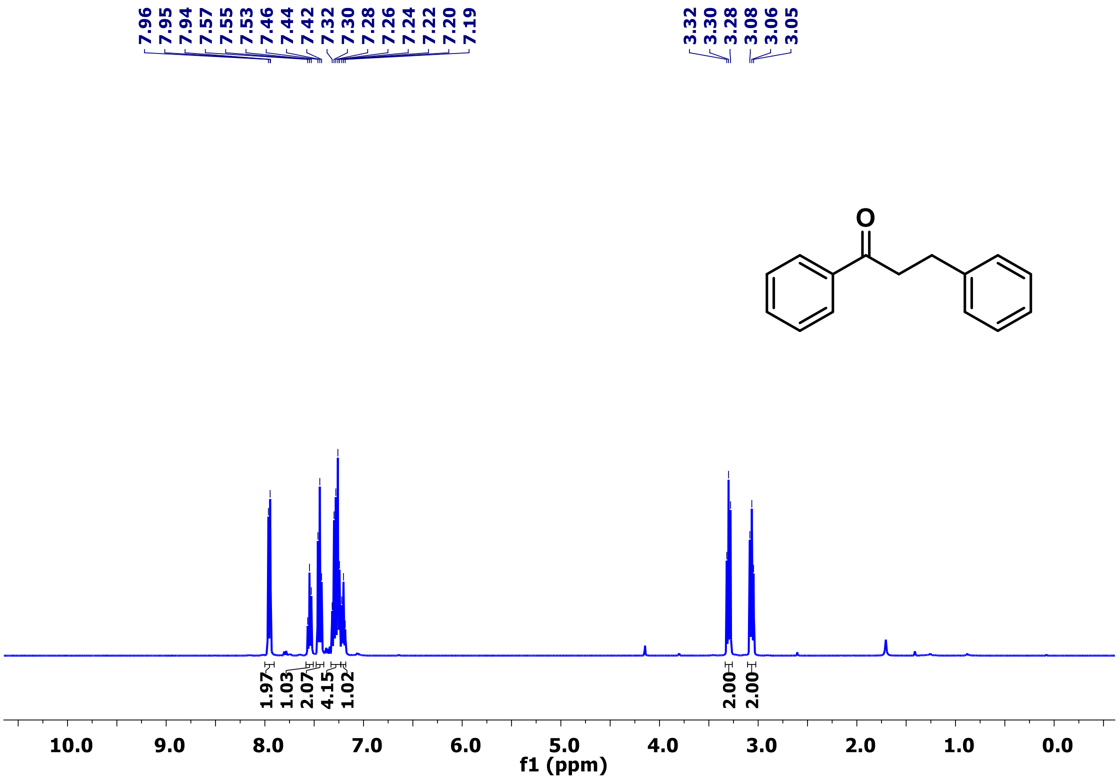

Figure S87: ^{1}H NMR spectrum (400 MHz) of **6q** in $CDCl_3$

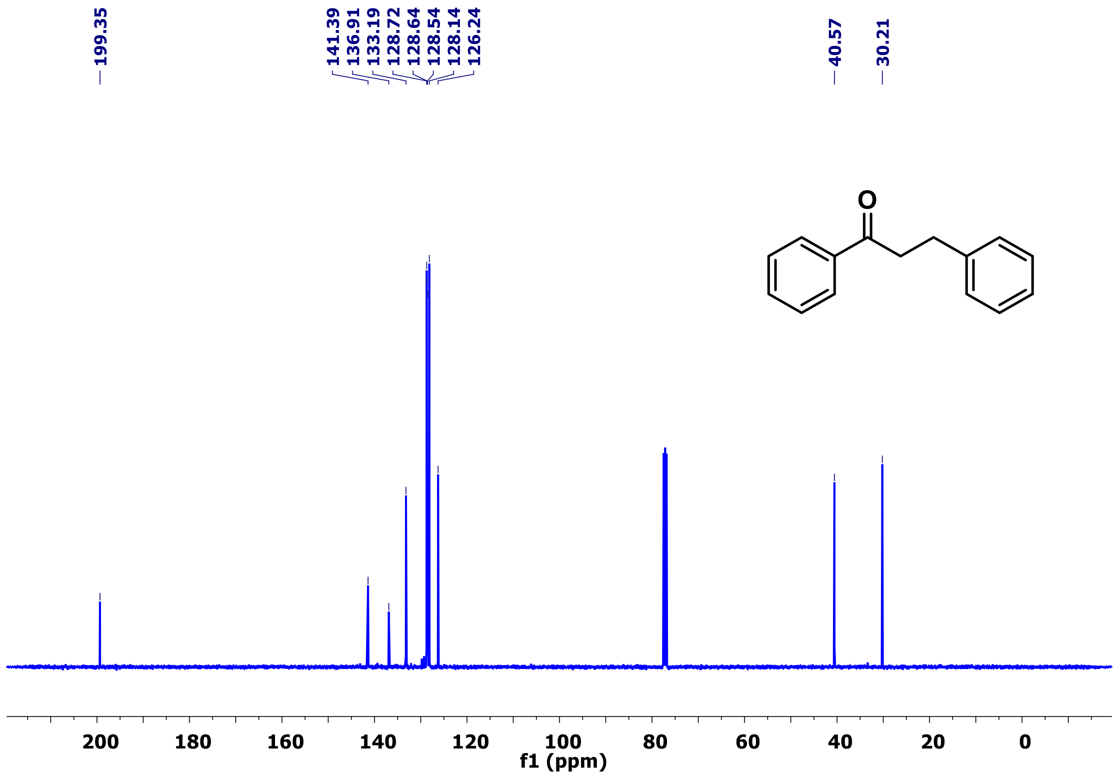

Figure S88: $^{13}C\{^{1}H\}$ NMR spectrum (101 MHz) of **6q** in $CDCl_3$

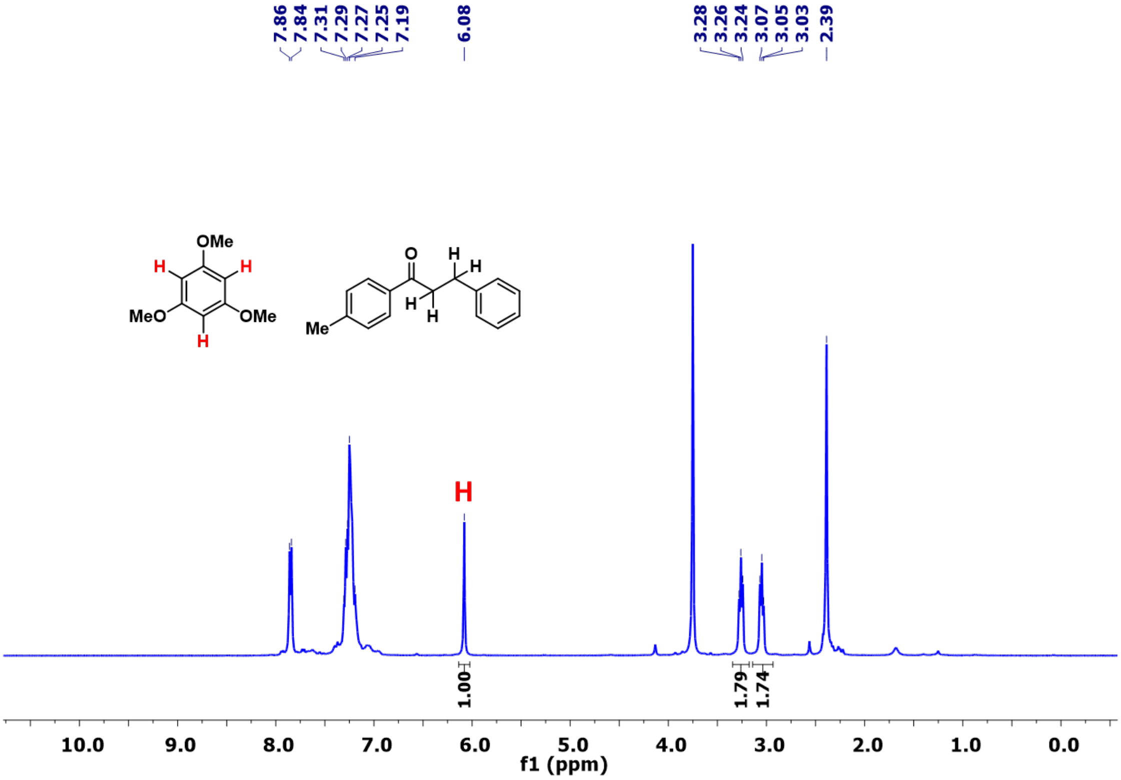

Figure S89: ^{1}H NMR spectrum (400 MHz, $CDCl_3$) of crude product mixture with 1/3 equiv. 1,3,5-trimethoxybenzene after hydrogenation of **5r**

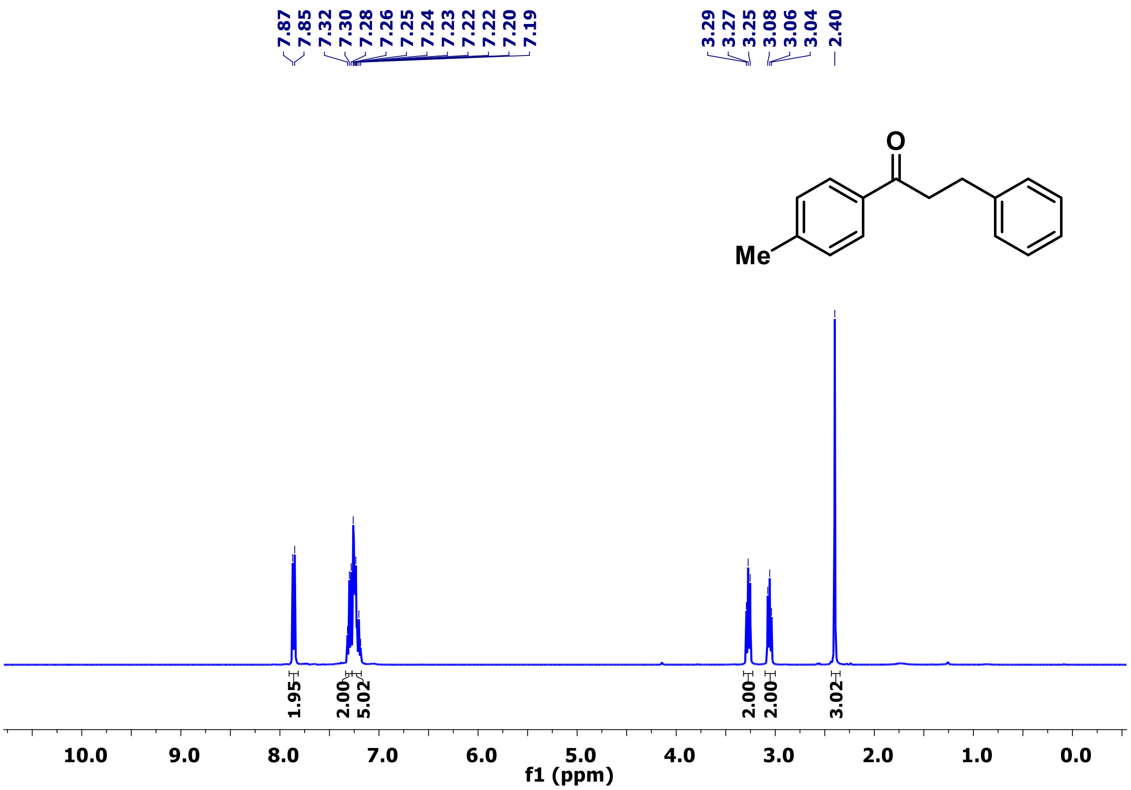

Figure S90: ^{1}H NMR spectrum (400 MHz) of **6r** in $CDCl_3$

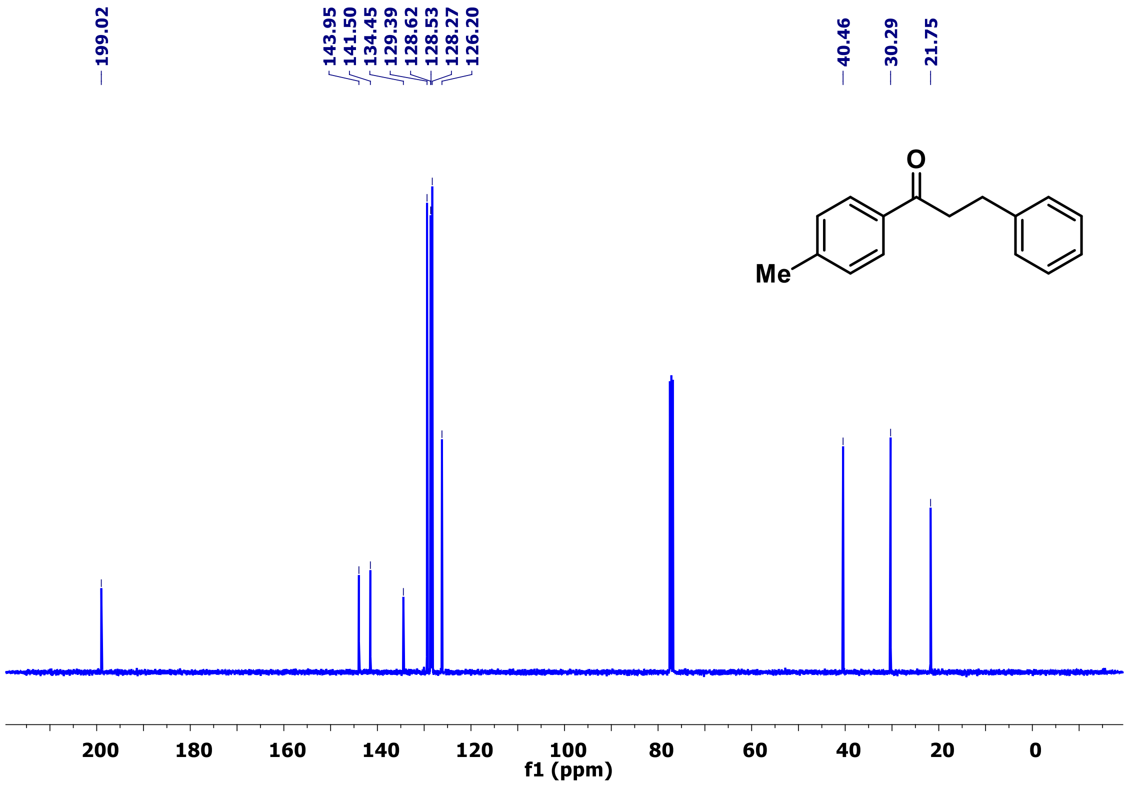

Figure S91: $^{13}C\{^{1}H\}$ NMR spectrum (101 MHz) of **6r** in $CDCl_3$

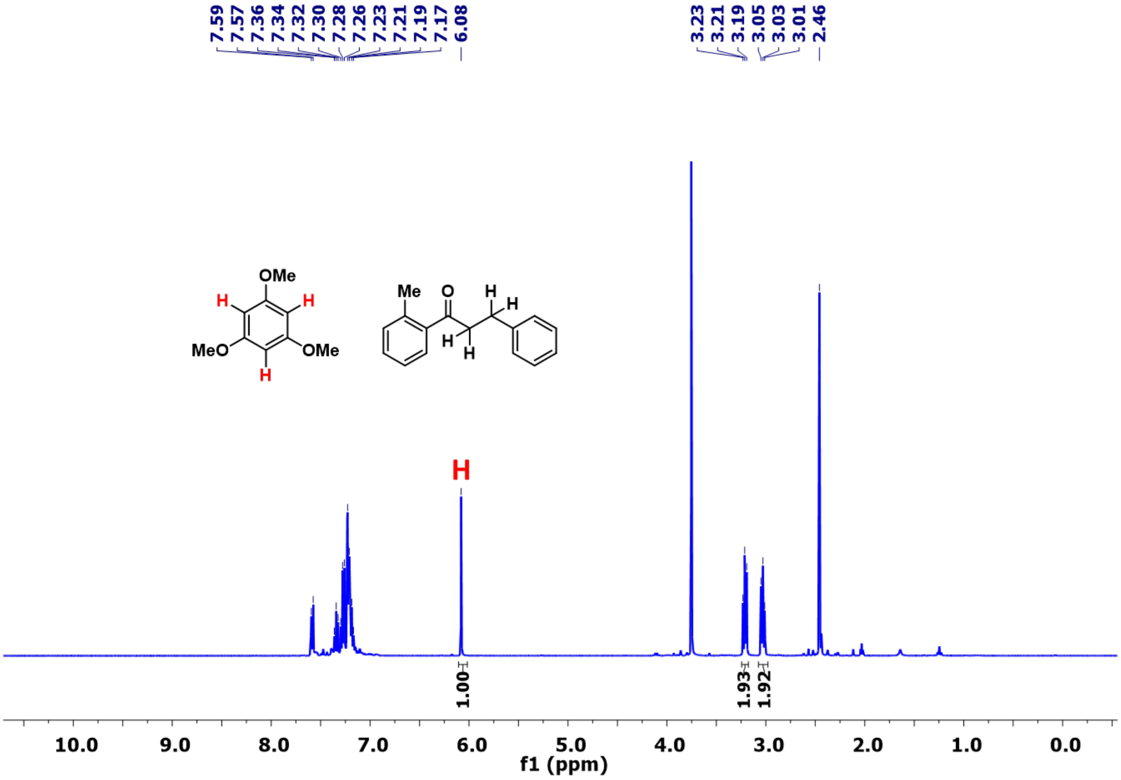

Figure S92: ^{1}H NMR spectrum (400 MHz, $CDCl_3$) of crude product mixture with 1/3 equiv. 1,3,5-trimethoxybenzene after hydrogenation of **5s**

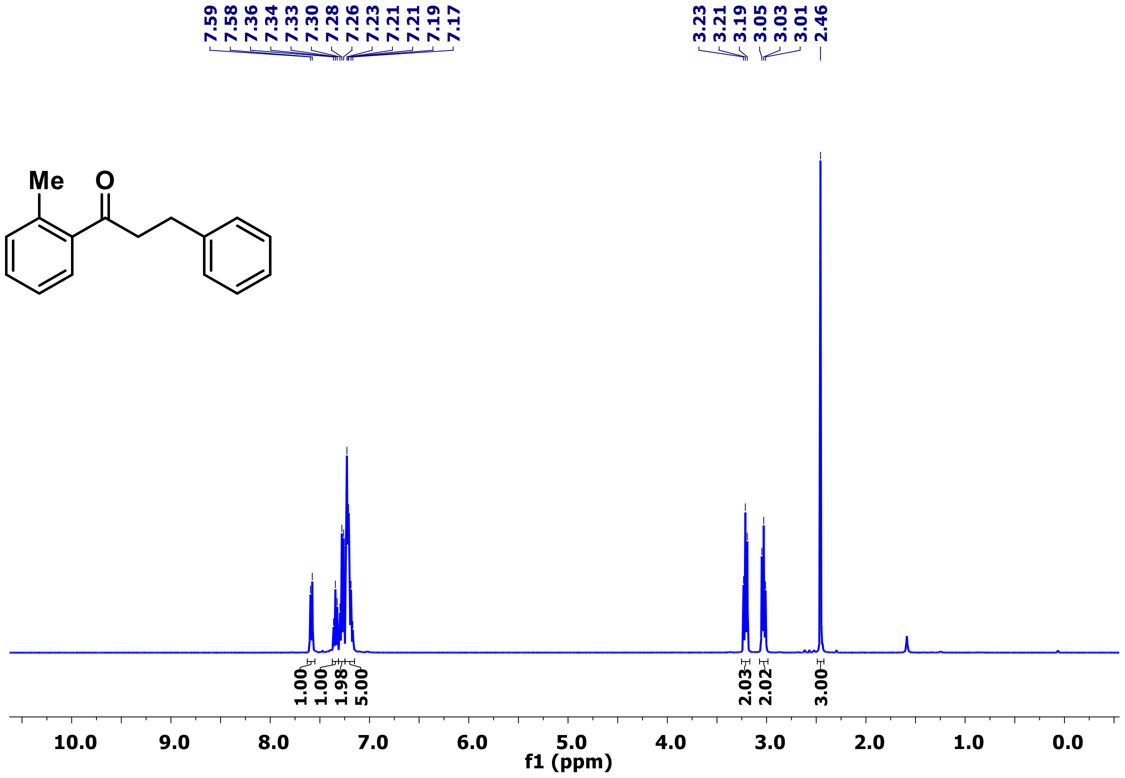

Figure S93: ^{1}H NMR spectrum (400 MHz) of **6s** in $CDCl_3$

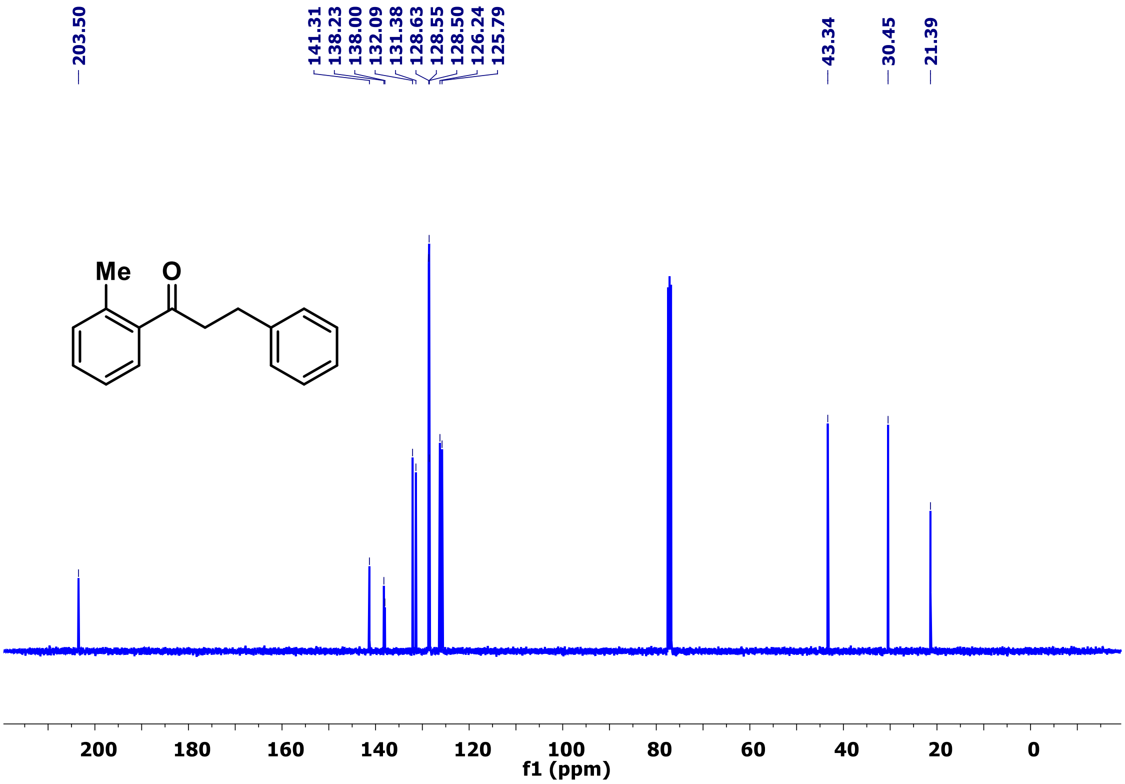

Figure S94: $^{13}C\{^{1}H\}$ NMR spectrum (101 MHz) of **6s** in $CDCl_3$

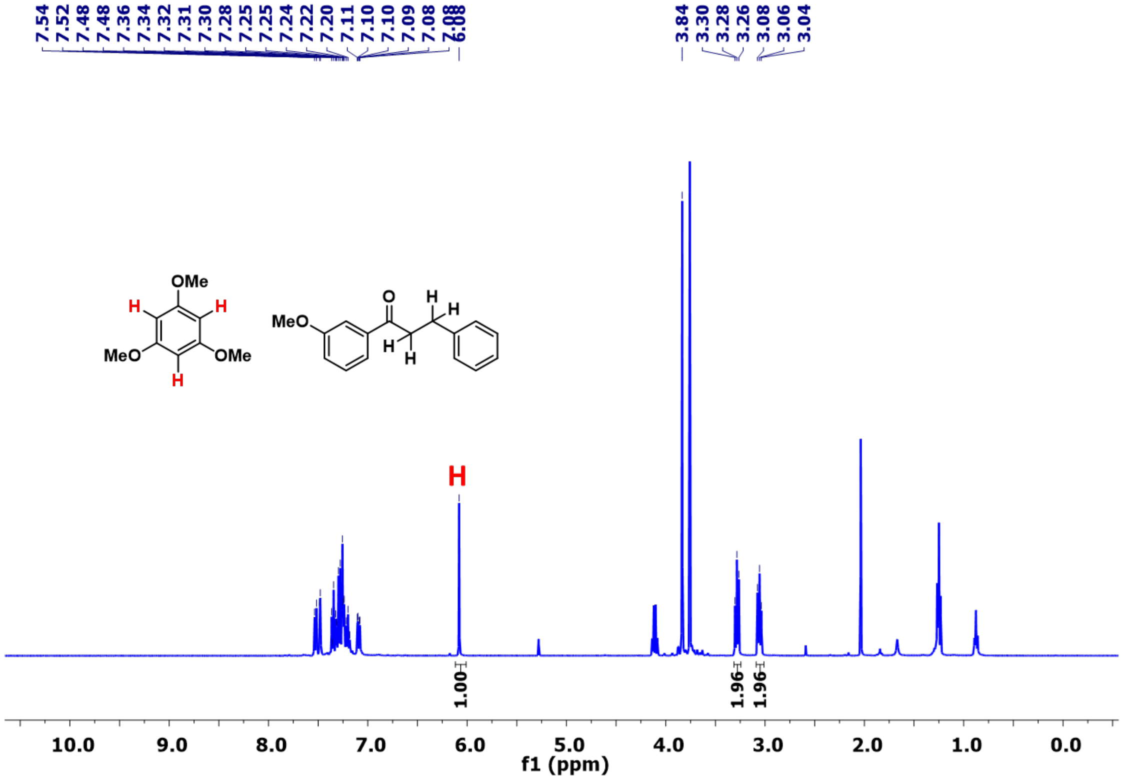

Figure S95: ^{1}H NMR spectrum (400 MHz, $CDCl_3$) of crude product mixture with 1/3 equiv. 1,3,5-trimethoxybenzene after hydrogenation of **5t**

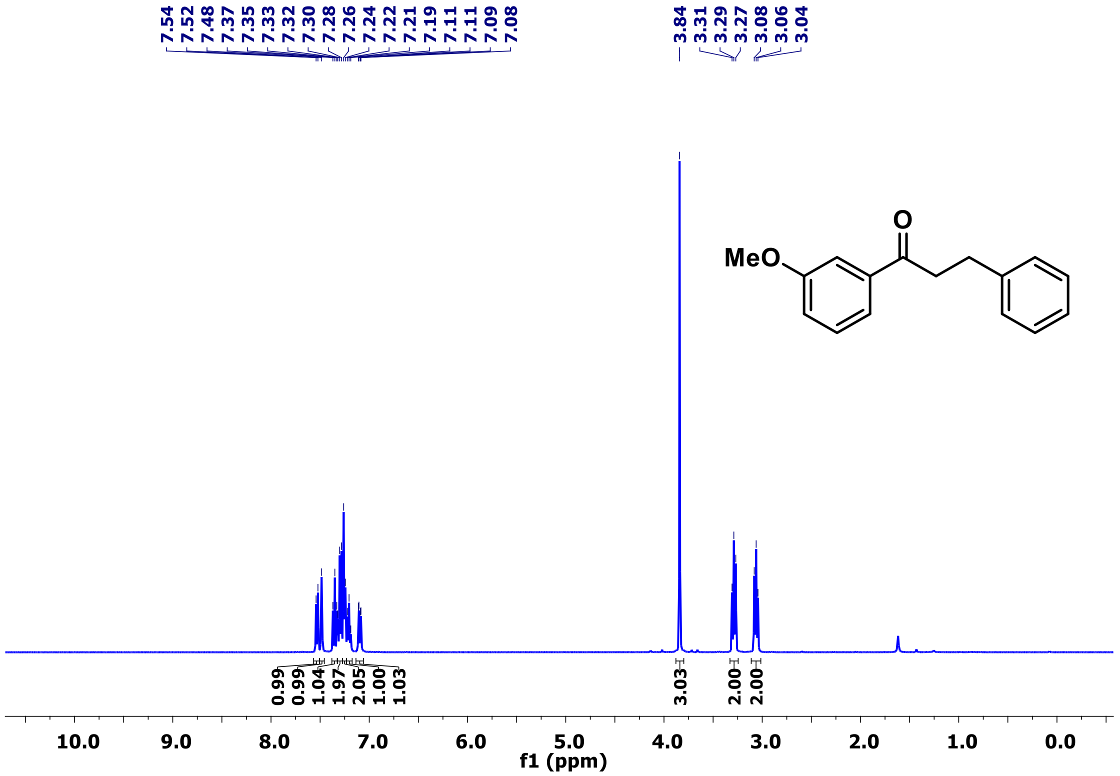

Figure S96: ^{1}H NMR spectrum (400 MHz) of **6t** in $CDCl_3$

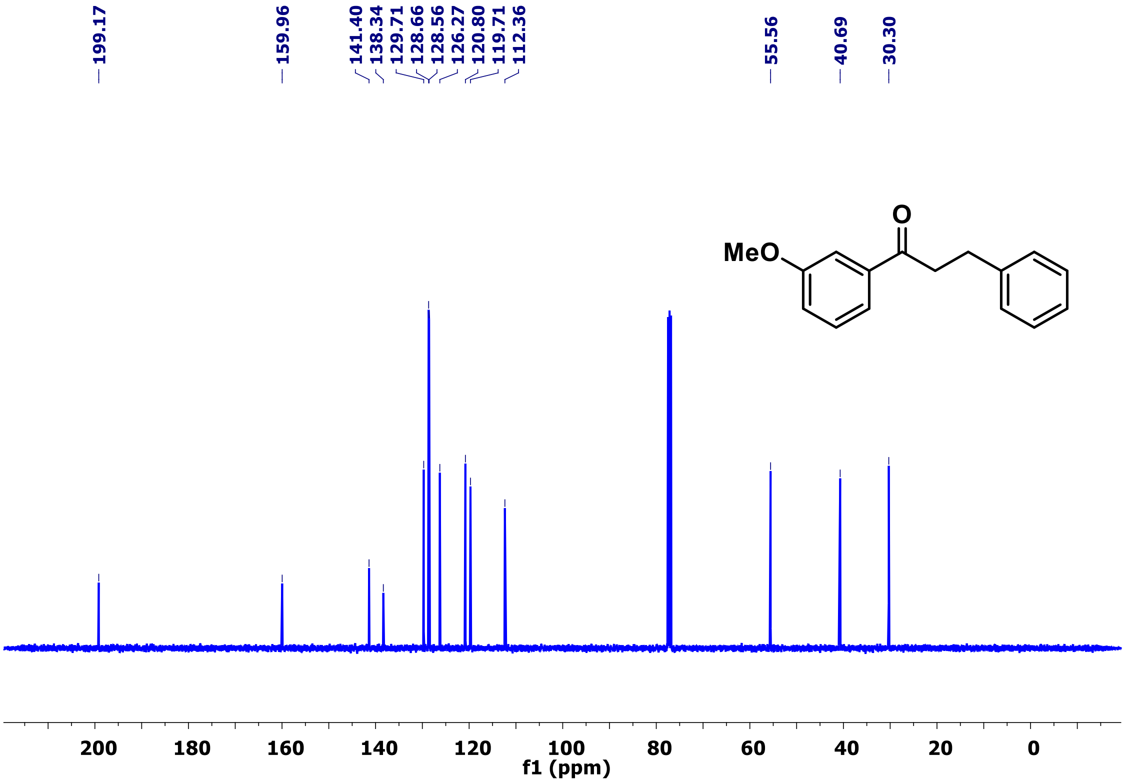

Figure S97: $^{13}C\{^{1}H\}$ NMR spectrum (101 MHz) of **6t** in $CDCl_3$

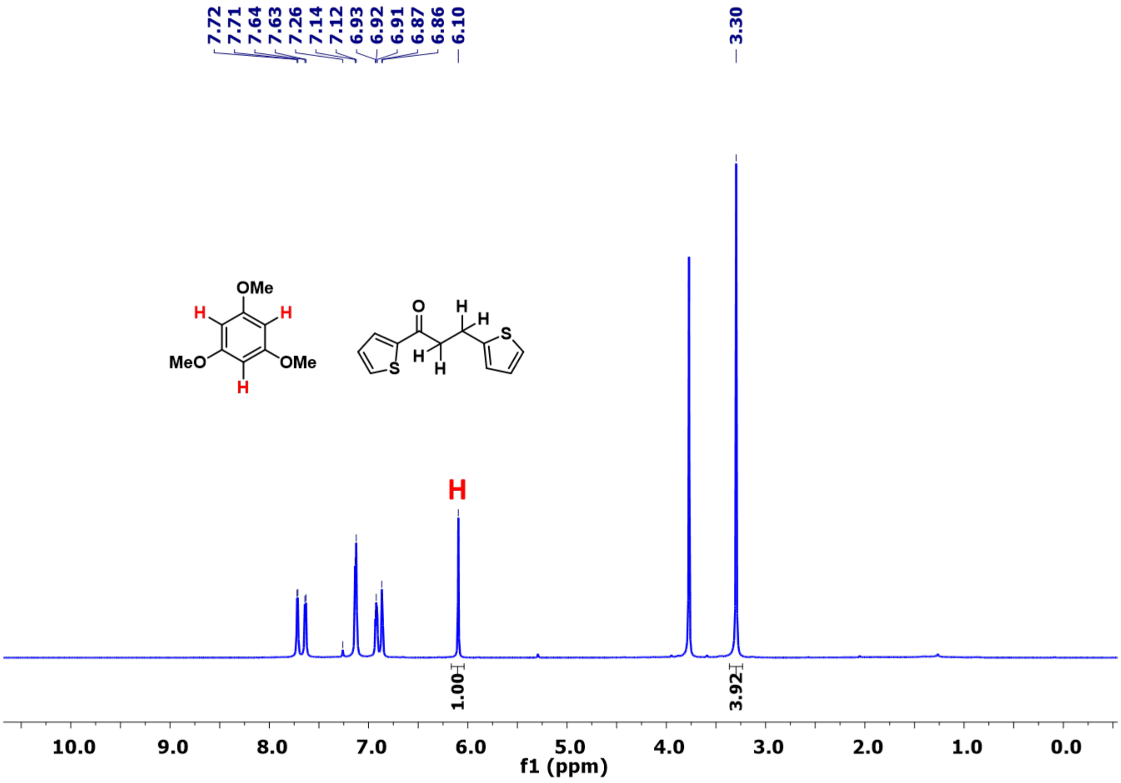

Figure S98: ^{1}H NMR spectrum (400 MHz, $CDCl_3$) of crude product mixture with 1/3 equiv. 1,3,5-trimethoxybenzene after hydrogenation of **5u**

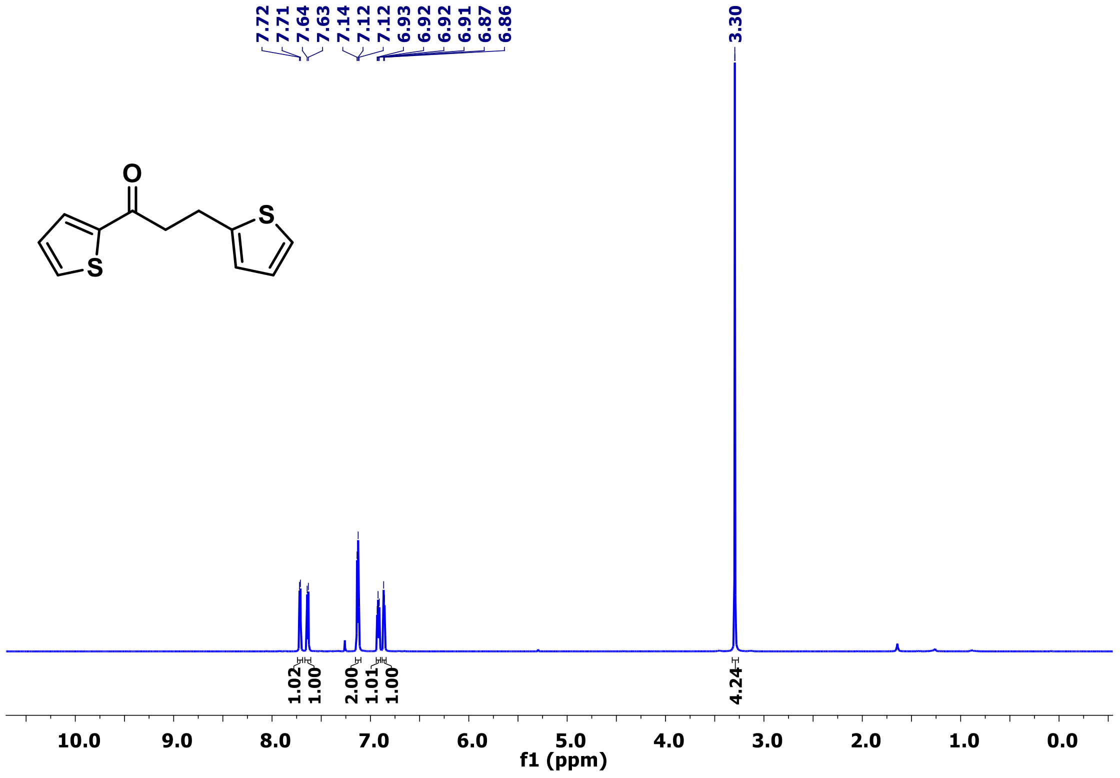

Figure S99: ^{1}H NMR spectrum (400 MHz) of **6u** in $CDCl_3$

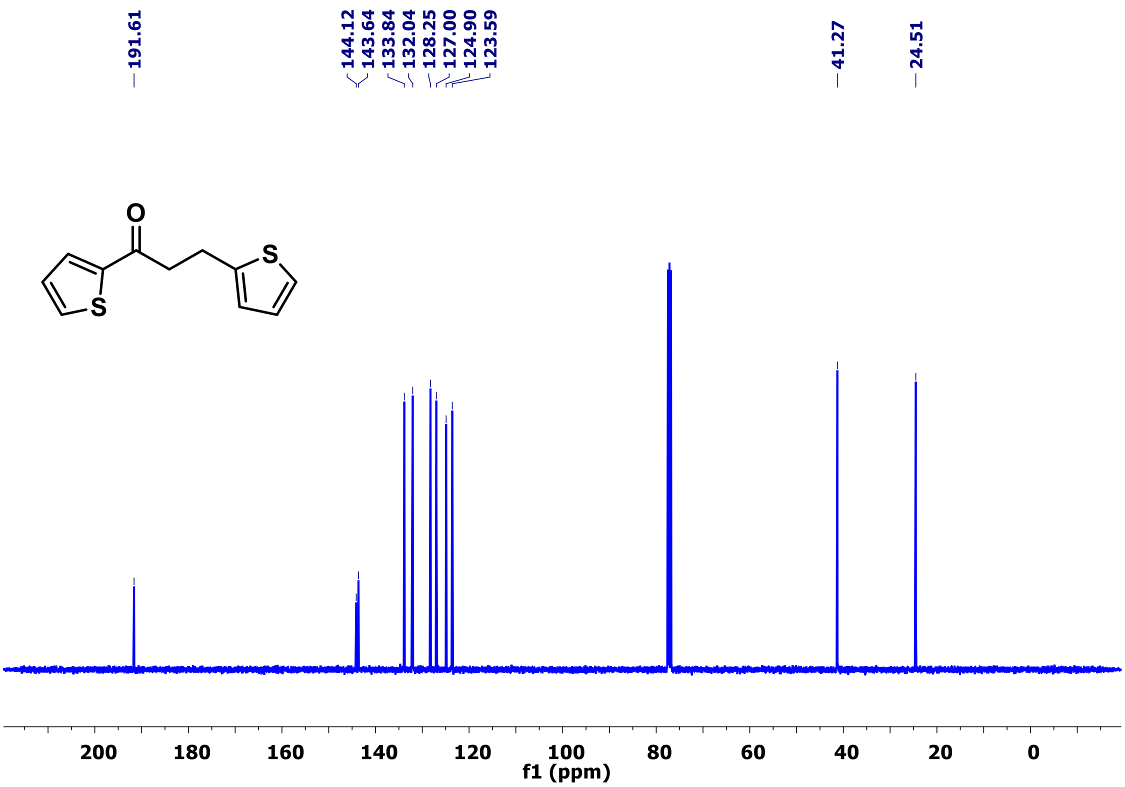

Figure S100: $^{13}C\{^{1}H\}$ NMR spectrum (101 MHz) of **6u** in $CDCl_3$

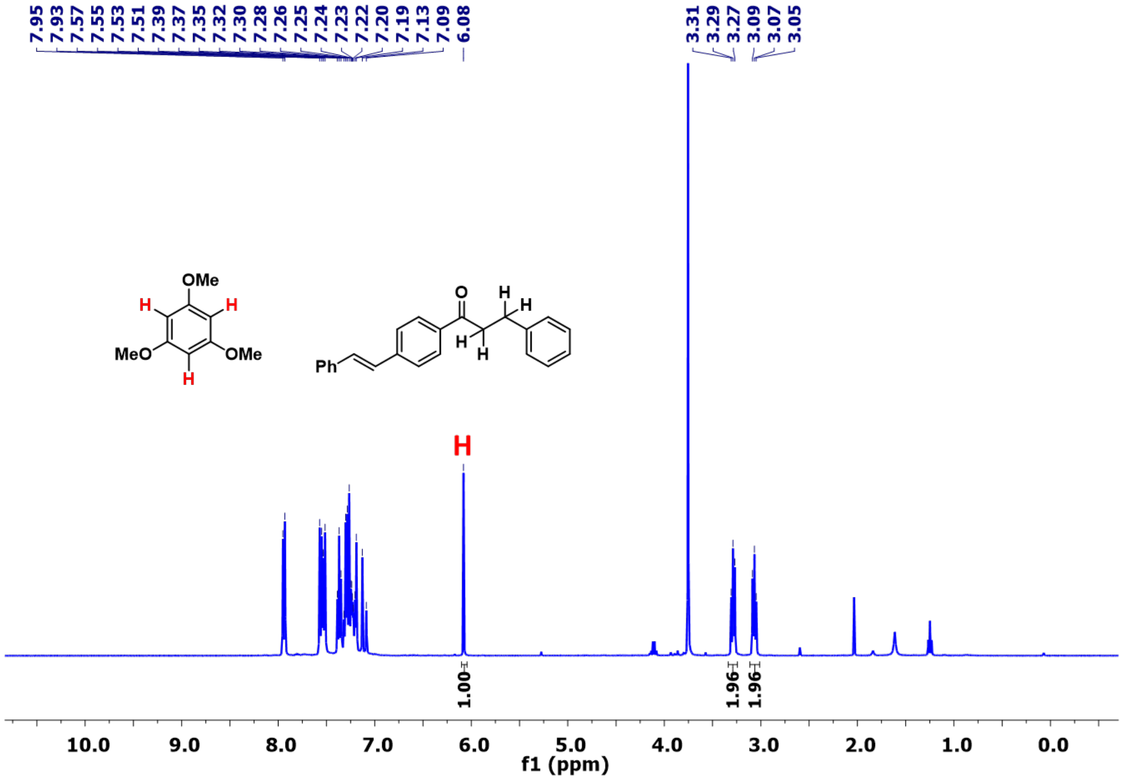

Figure S101: ^{1}H NMR spectrum (400 MHz, $CDCl_3$) of crude product mixture with 1/3 equiv. 1,3,5-trimethoxybenzene after hydrogenation of **5v**

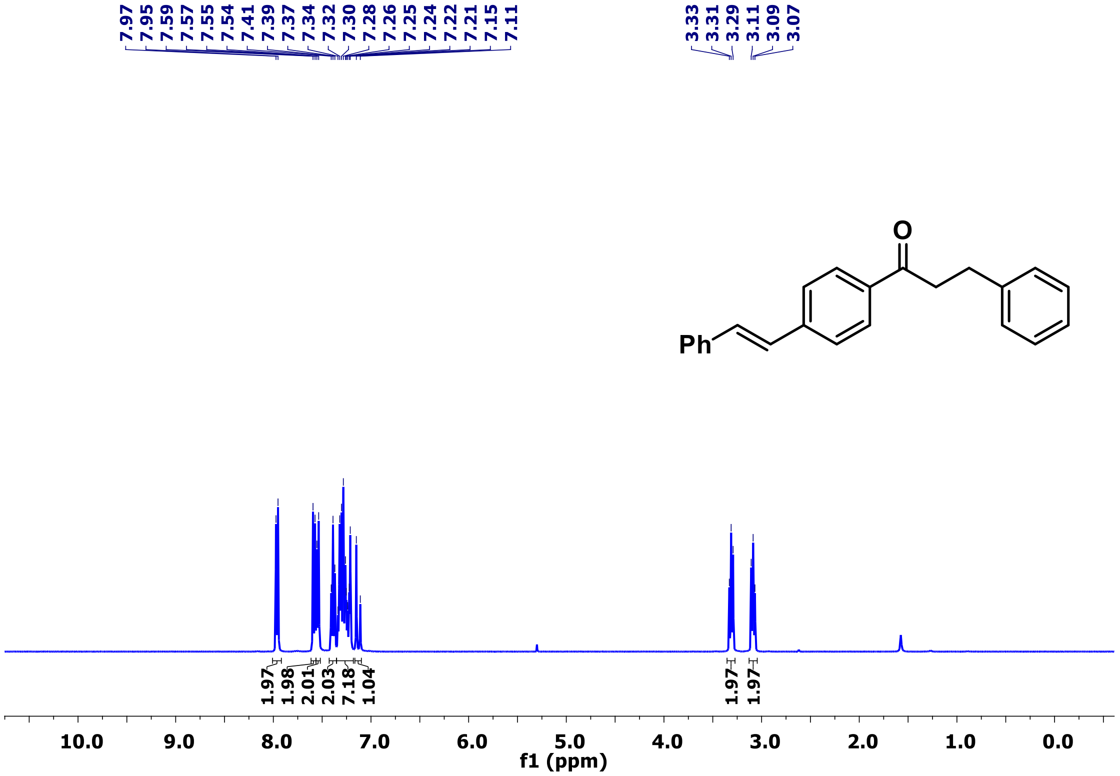

Figure S102: ^{1}H NMR spectrum (400 MHz) of **6v** in $CDCl_3$

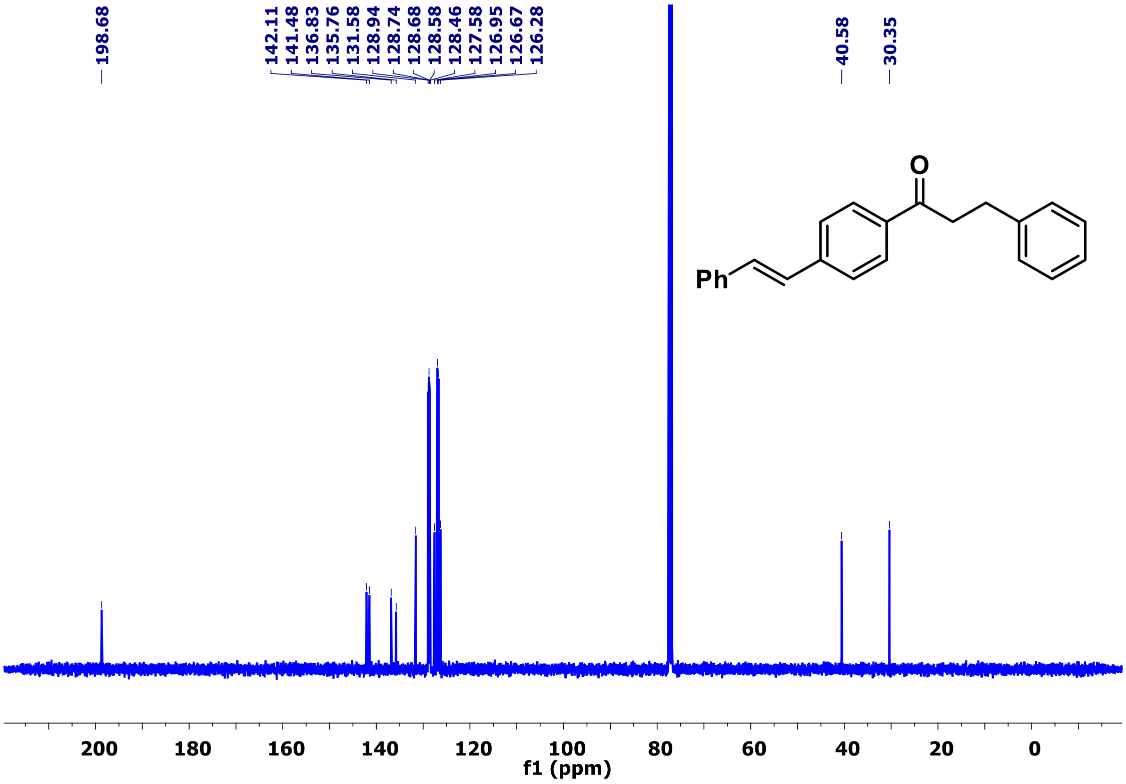

Figure S103: $^{13}C\{^{1}H\}$ NMR spectrum (101 MHz) of **6v** in $CDCl_3$

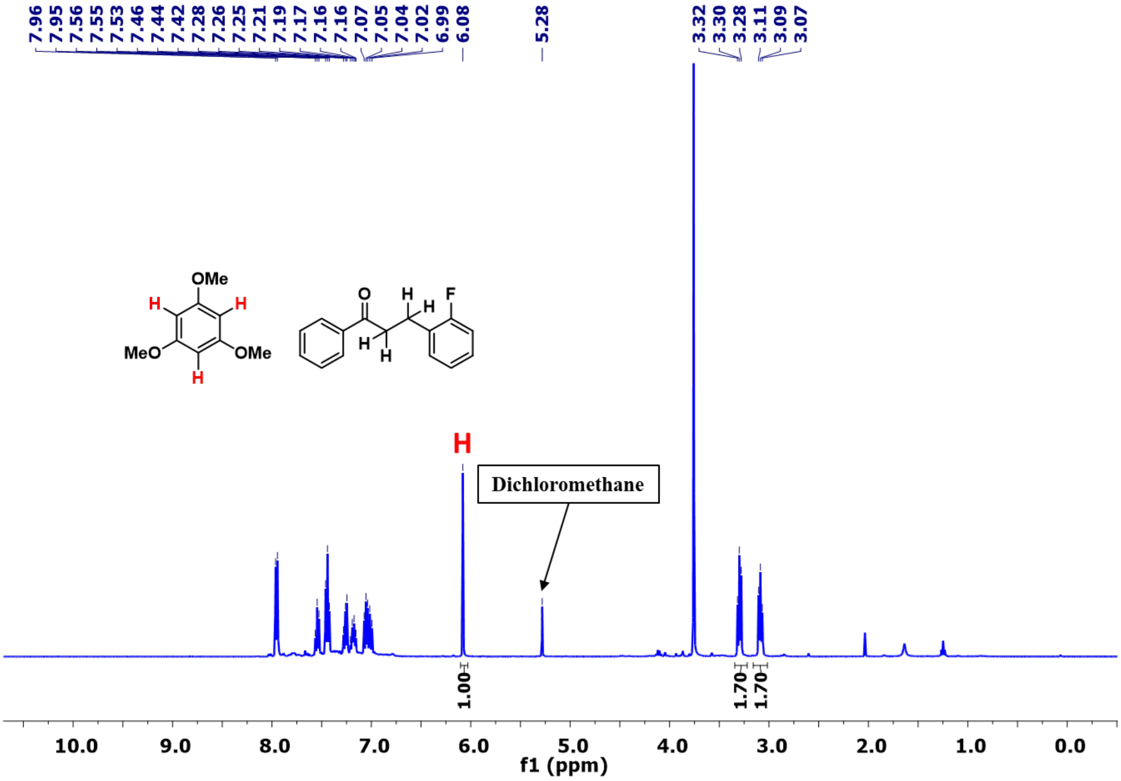

Figure S104: ^{1}H NMR spectrum (400 MHz, $CDCl_3$) of crude product mixture with 1/3 equiv. 1,3,5-trimethoxybenzene after hydrogenation of **5w**

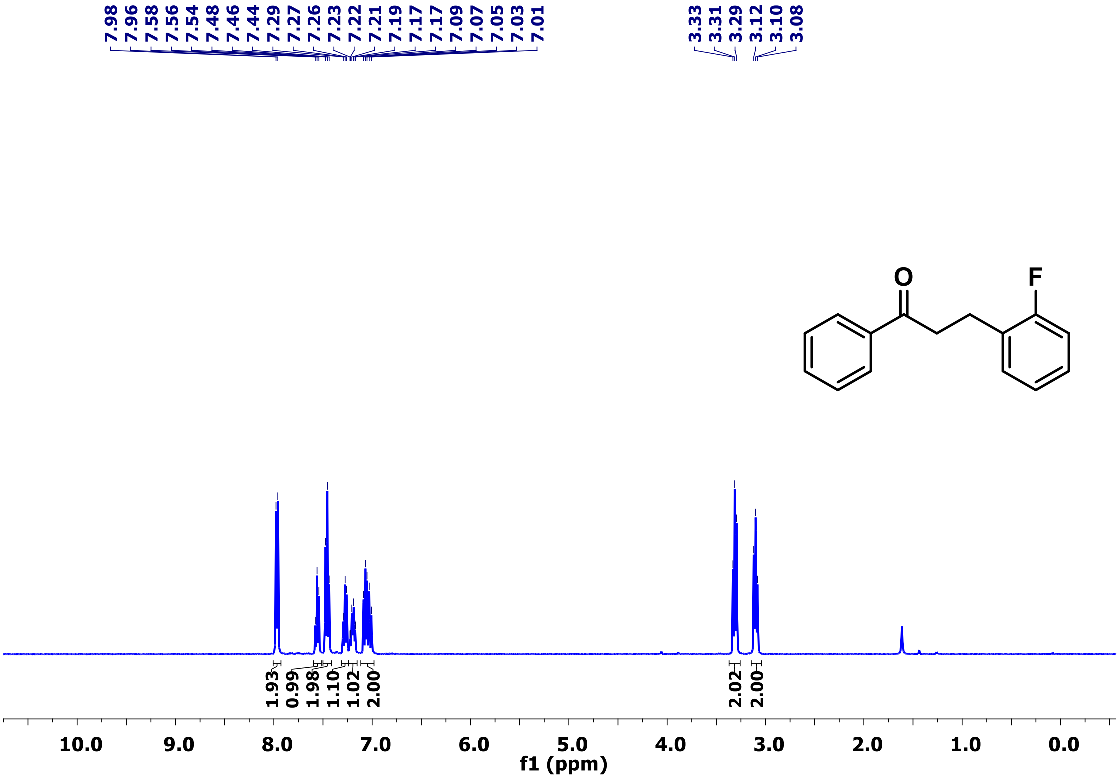

Figure S105: ^{1}H NMR spectrum (400 MHz) of **6w** in $CDCl_3$

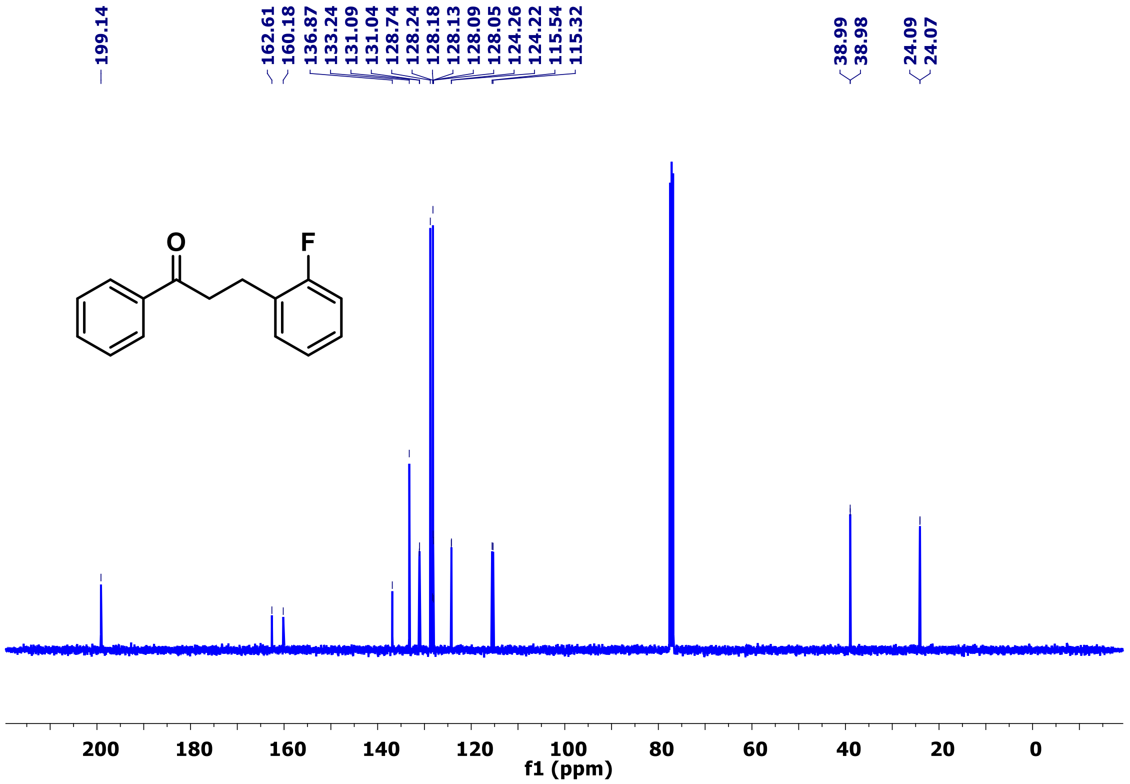

Figure S106: $^{13}C\{^1H\}$ NMR spectrum (101 MHz) of **6w** in $CDCl_3$

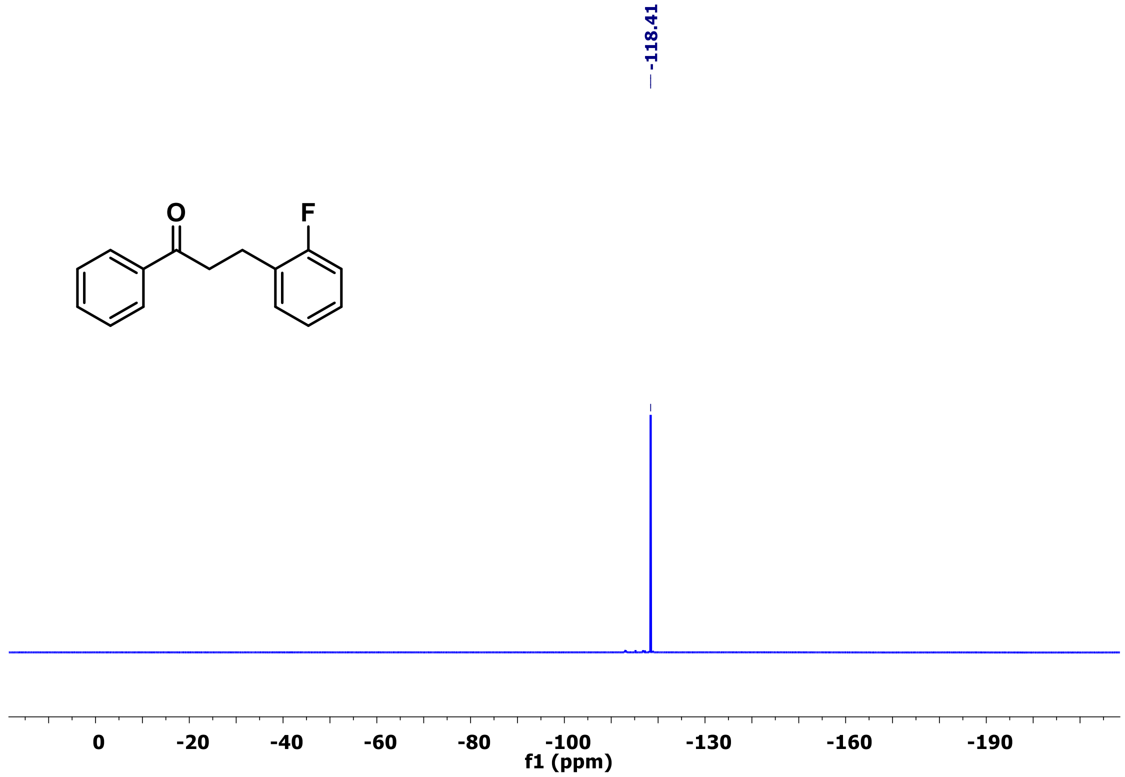

Figure S107: $^{19}F\{^1H\}$ NMR spectrum (377 MHz) of **6w** in $CDCl_3$

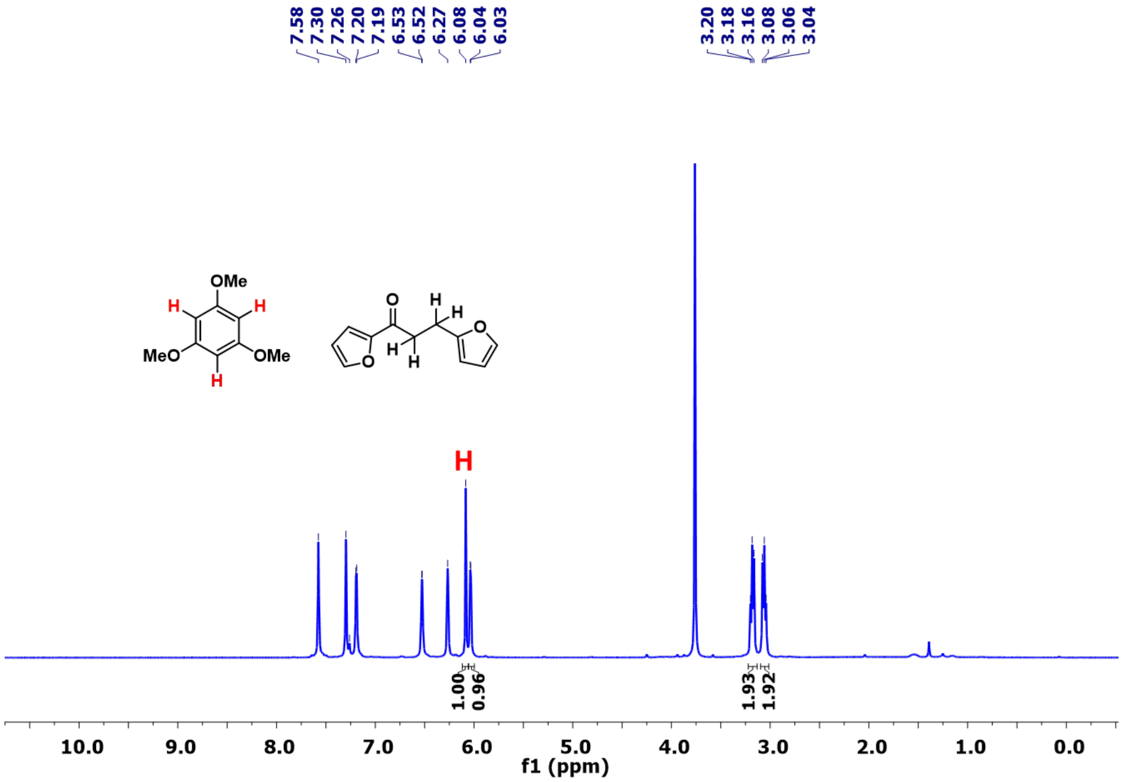

Figure S108: ^{1}H NMR spectrum (400 MHz, $CDCl_3$) of crude product mixture with 1/3 equiv. 1,3,5-trimethoxybenzene after hydrogenation of **5x**

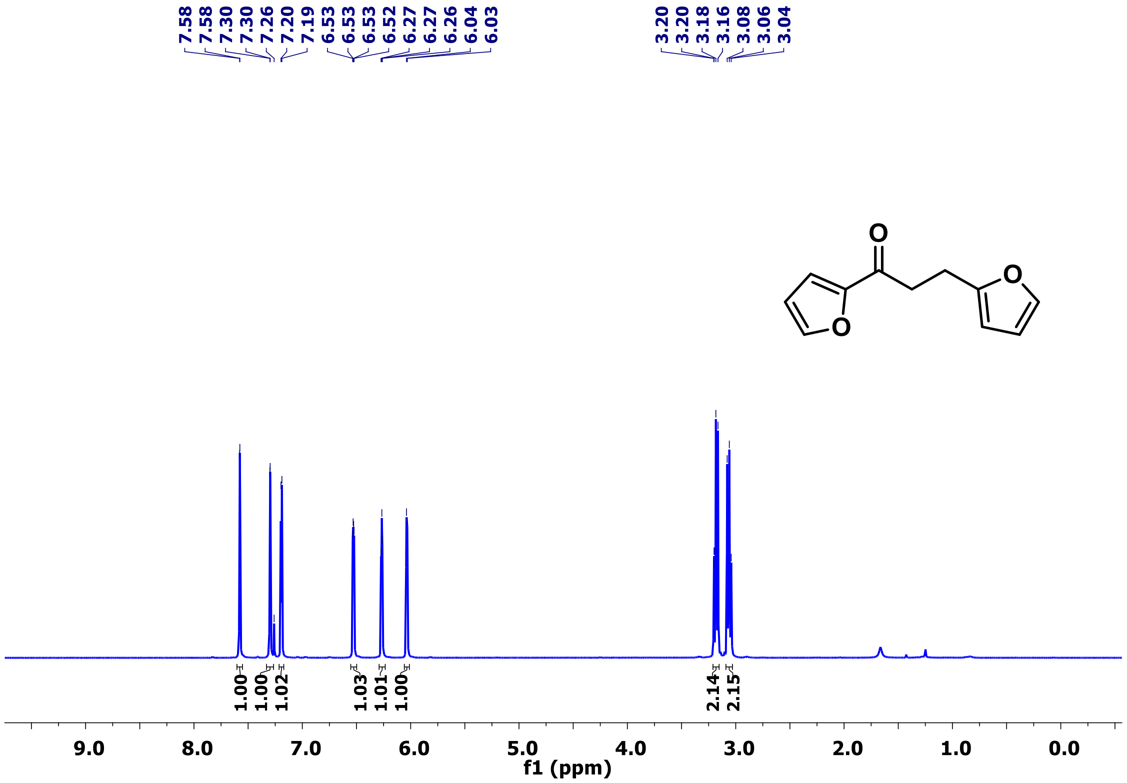

Figure S109: ^{1}H NMR spectrum (400 MHz) of **6x** in $CDCl_3$

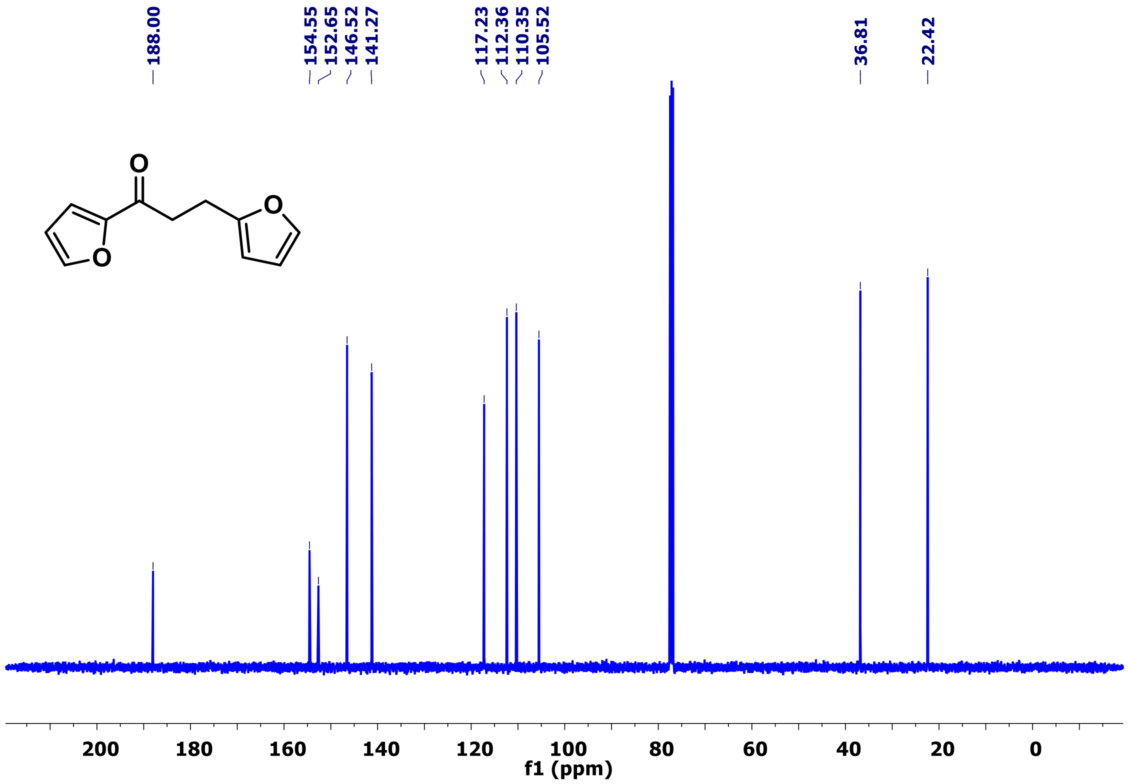

Figure S110: $^{13}C\{^{1}H\}$ NMR spectrum (101 MHz) of **6x** in $CDCl_3$

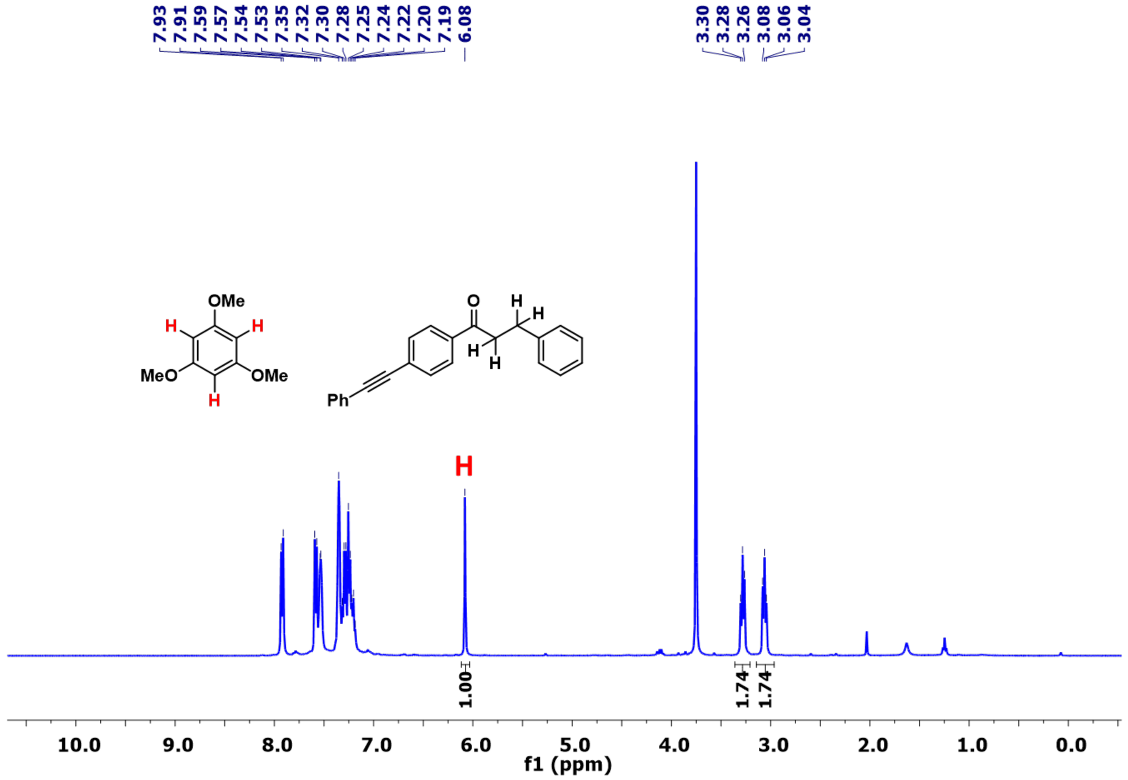

Figure S111: ^{1}H NMR spectrum (400 MHz, $CDCl_3$) of crude product mixture with 1/3 equiv. 1,3,5-trimethoxybenzene after hydrogenation of **5y**

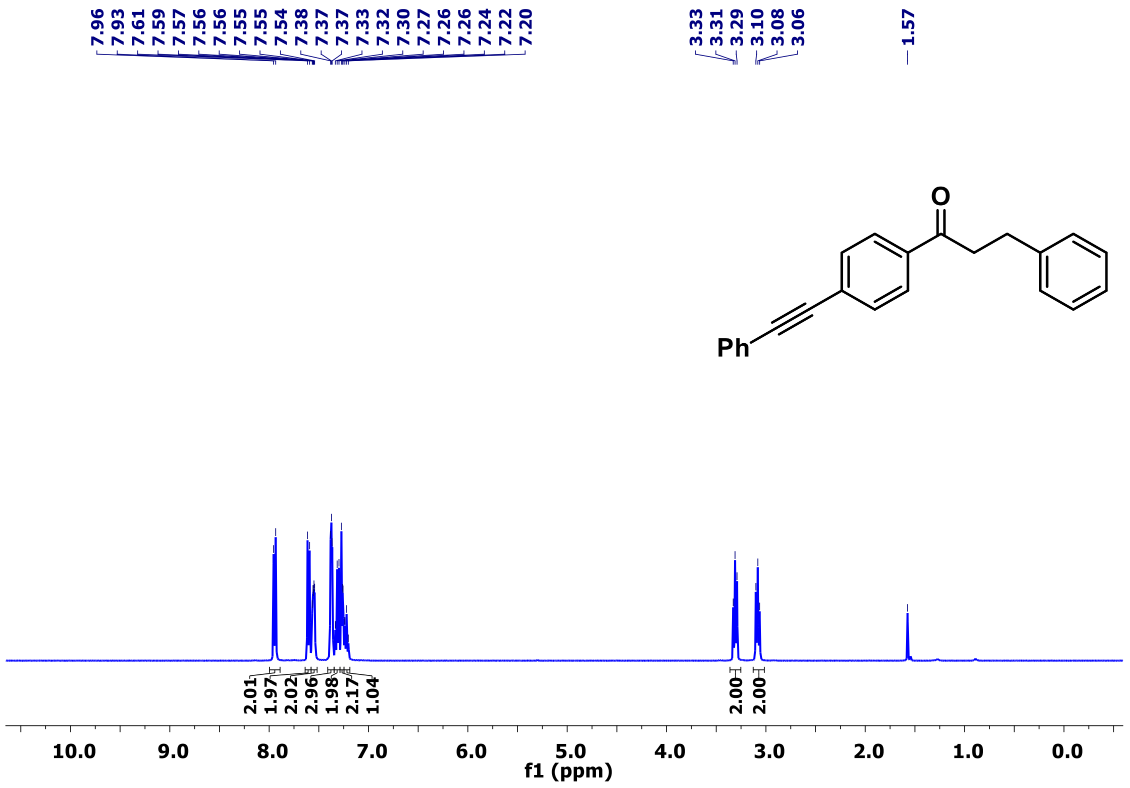

Figure S112: ^{1}H NMR spectrum (400 MHz) of **6y** in $CDCl_3$

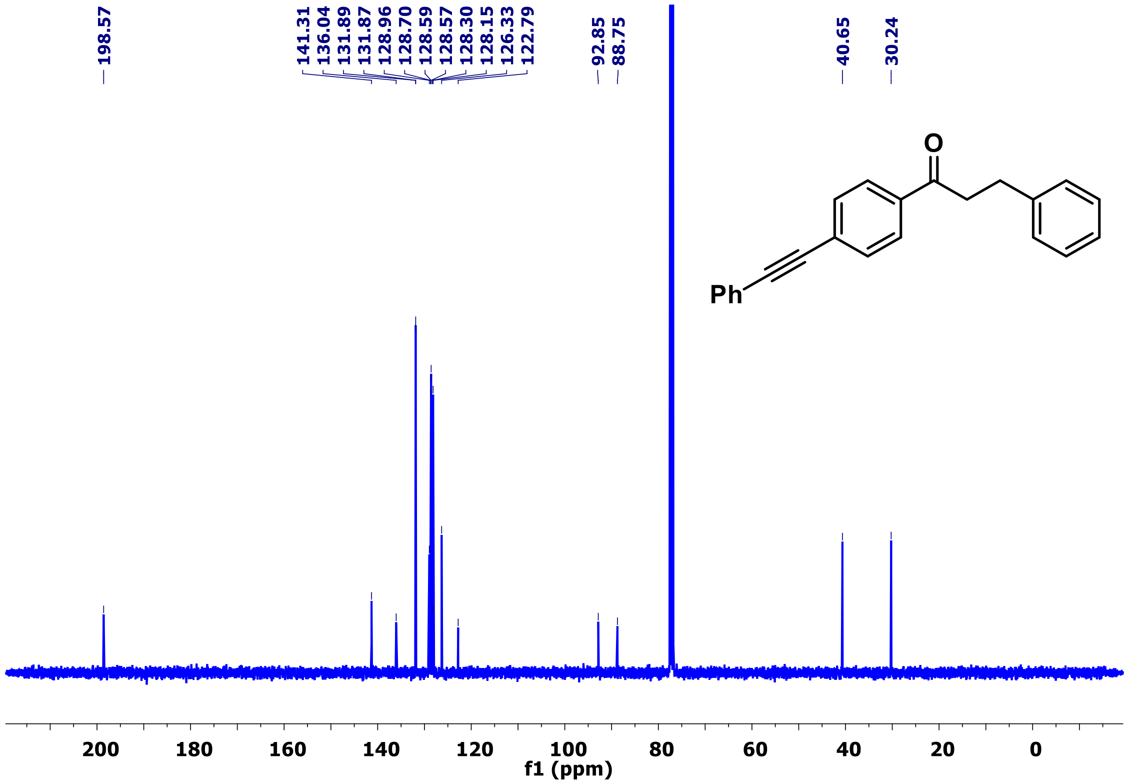

Figure S113: $^{13}C\{^{1}H\}$ NMR spectrum (101 MHz) of **6y** in $CDCl_3$

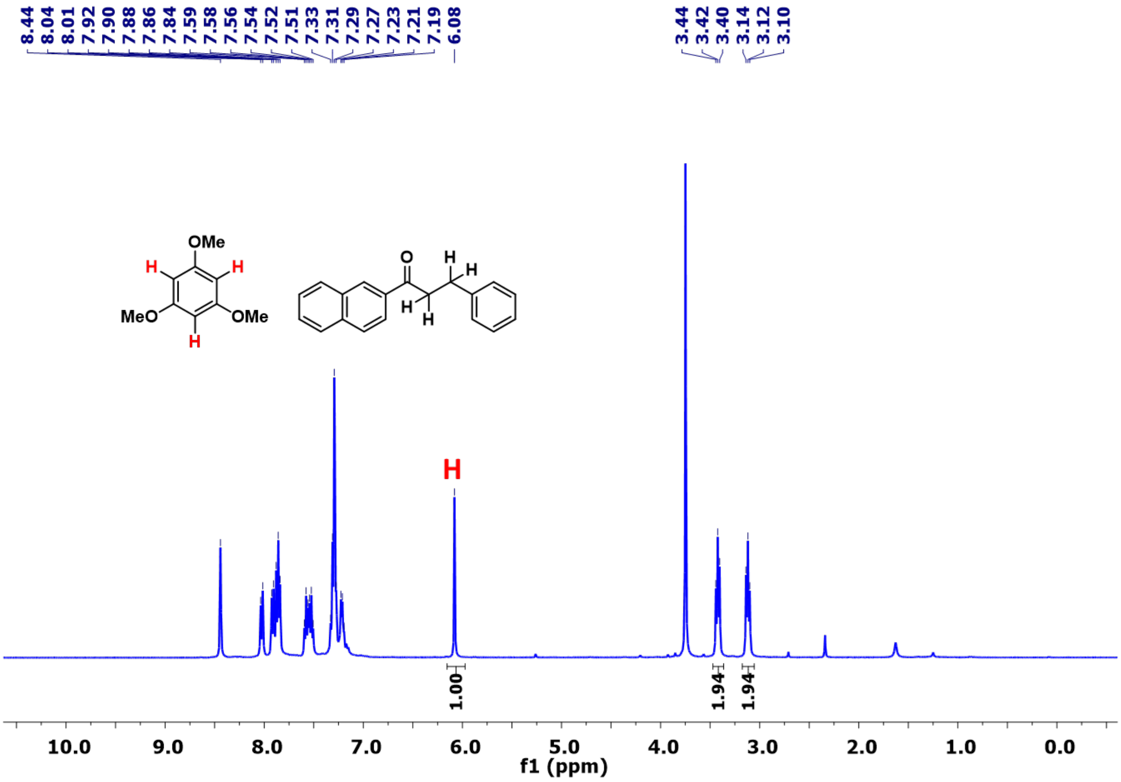

Figure S114: ^{1}H NMR spectrum (400 MHz, $CDCl_3$) of crude product mixture with 1/3 equiv. 1,3,5-trimethoxybenzene after hydrogenation of **5z**

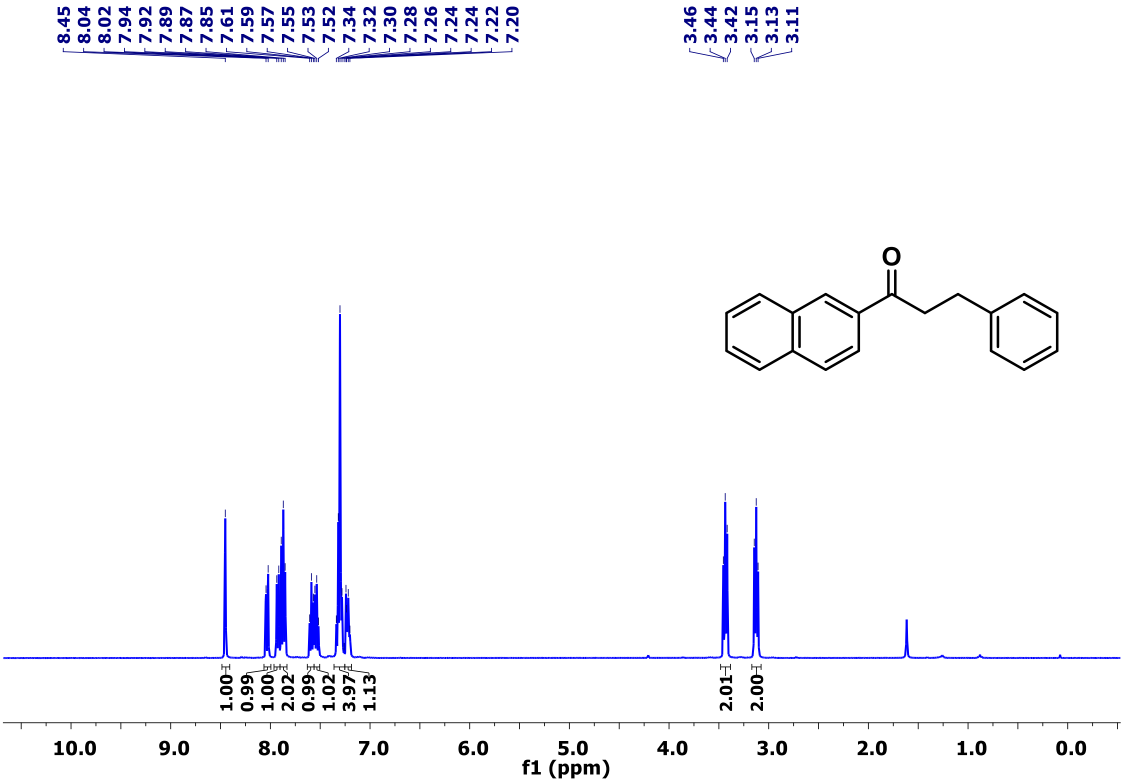

Figure S115: ^{1}H NMR spectrum (400 MHz) of **6z** in $CDCl_3$

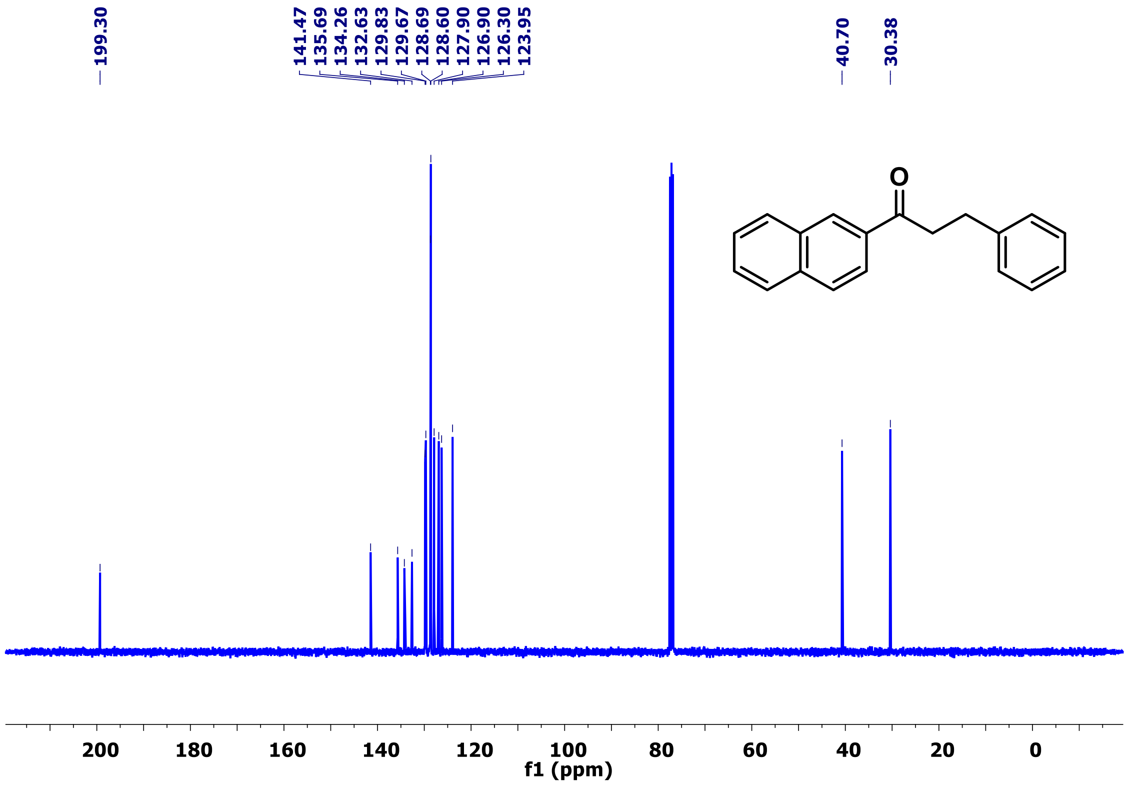

Figure S116: $^{13}C\{^{1}H\}$ NMR spectrum (101 MHz) of **6z** in $CDCl_3$

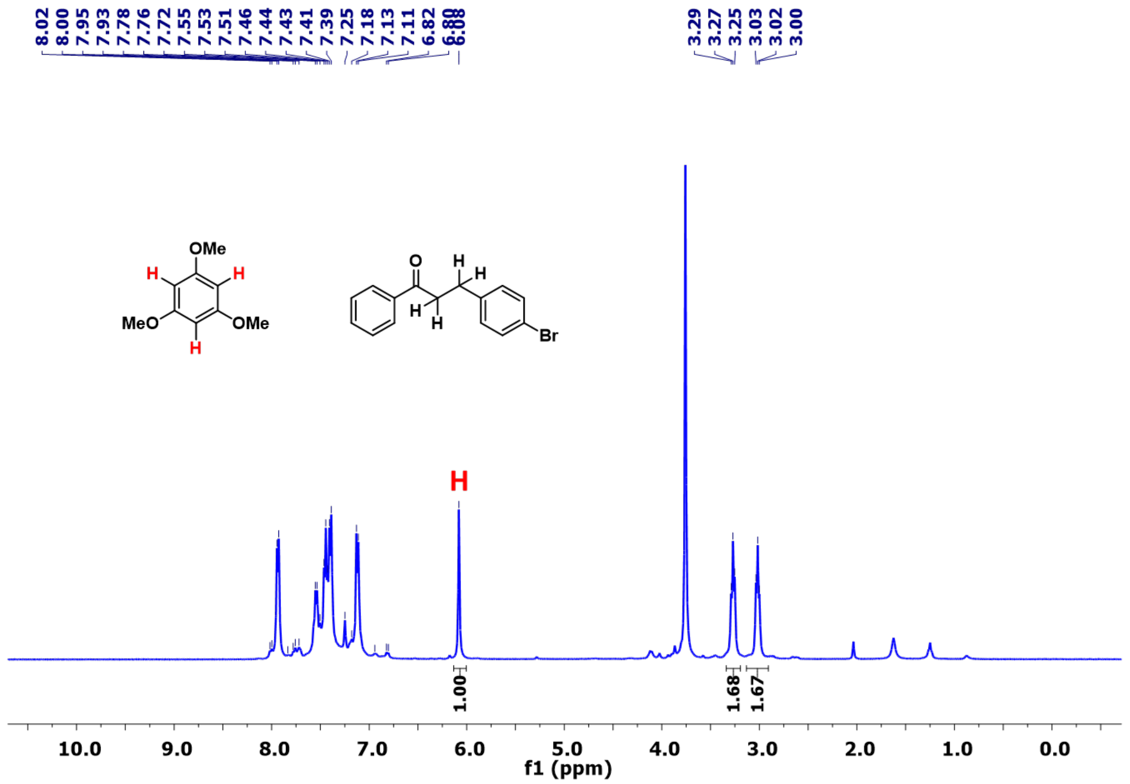

Figure S117: ^{1}H NMR spectrum (400 MHz, $CDCl_3$) of crude product mixture with 1/3 equiv. 1,3,5-trimethoxybenzene after hydrogenation of **5aa**

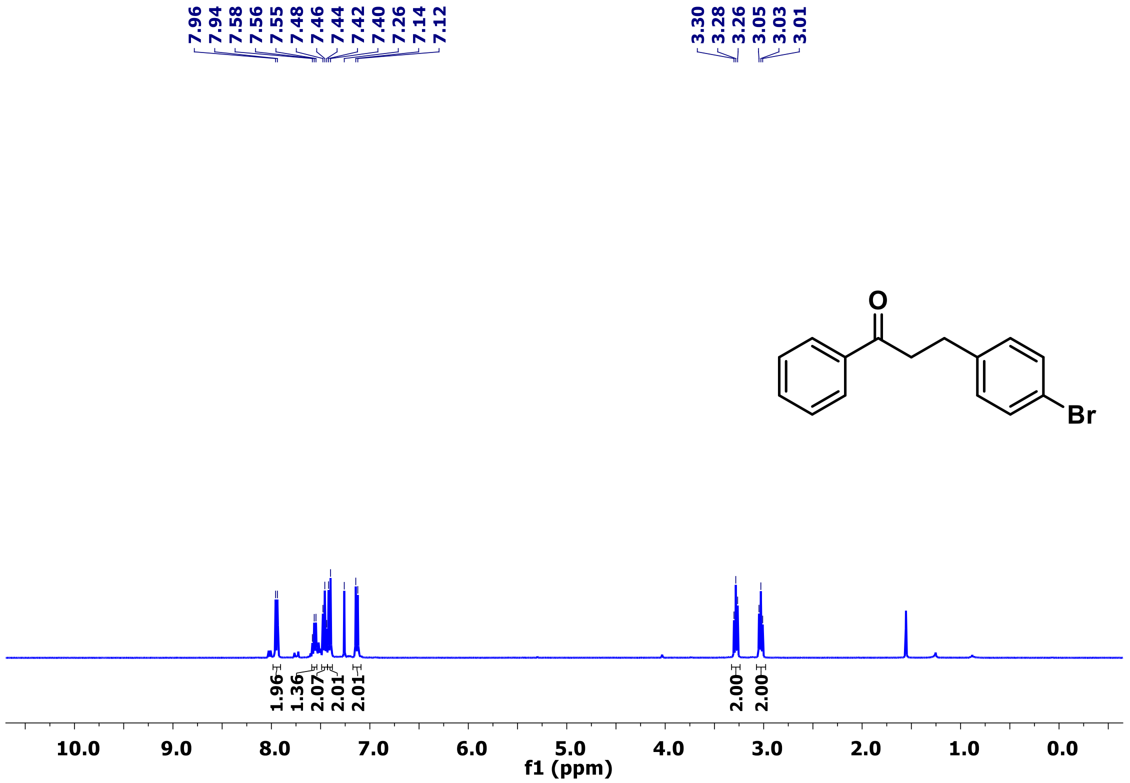

Figure S118: ^{1}H NMR spectrum (400 MHz) of **6aa** in $CDCl_3$

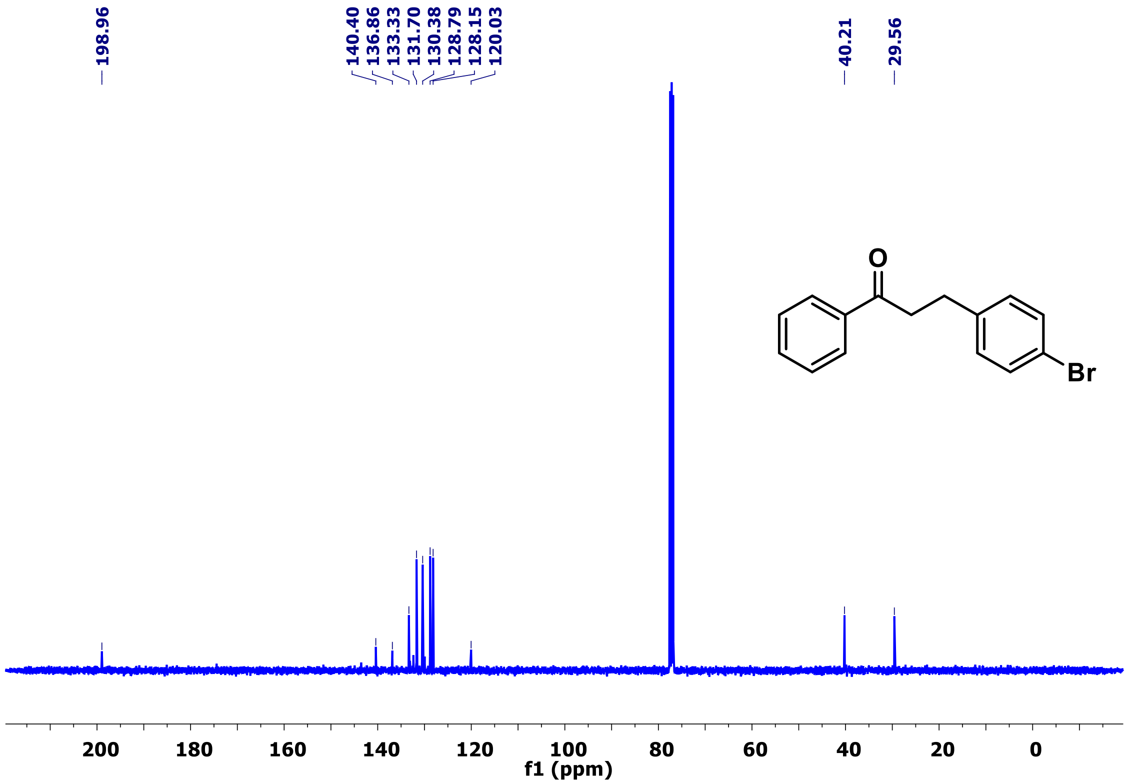

Figure S119: $^{13}C\{^{1}H\}$ NMR spectrum (101 MHz) of **6aa** in $CDCl_3$

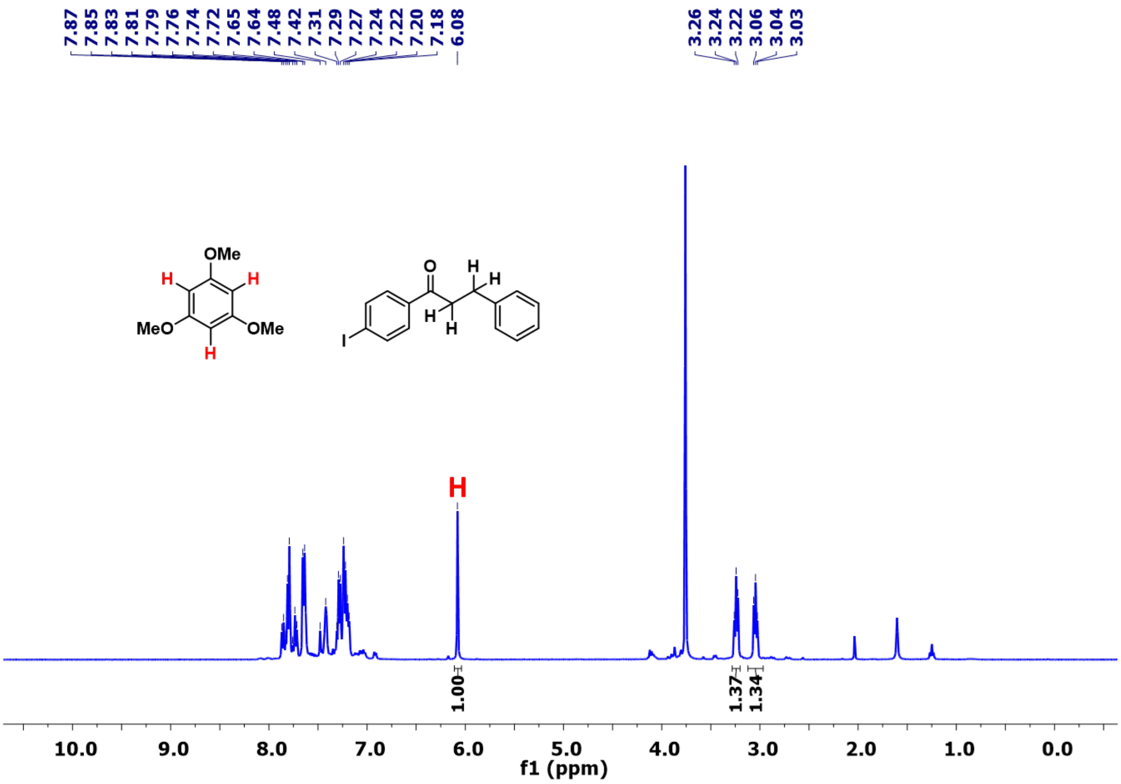

Figure S120: ^{1}H NMR spectrum (400 MHz, $CDCl_3$) of crude product mixture with 1/3 equiv. 1,3,5-trimethoxybenzene after hydrogenation of **5ab**

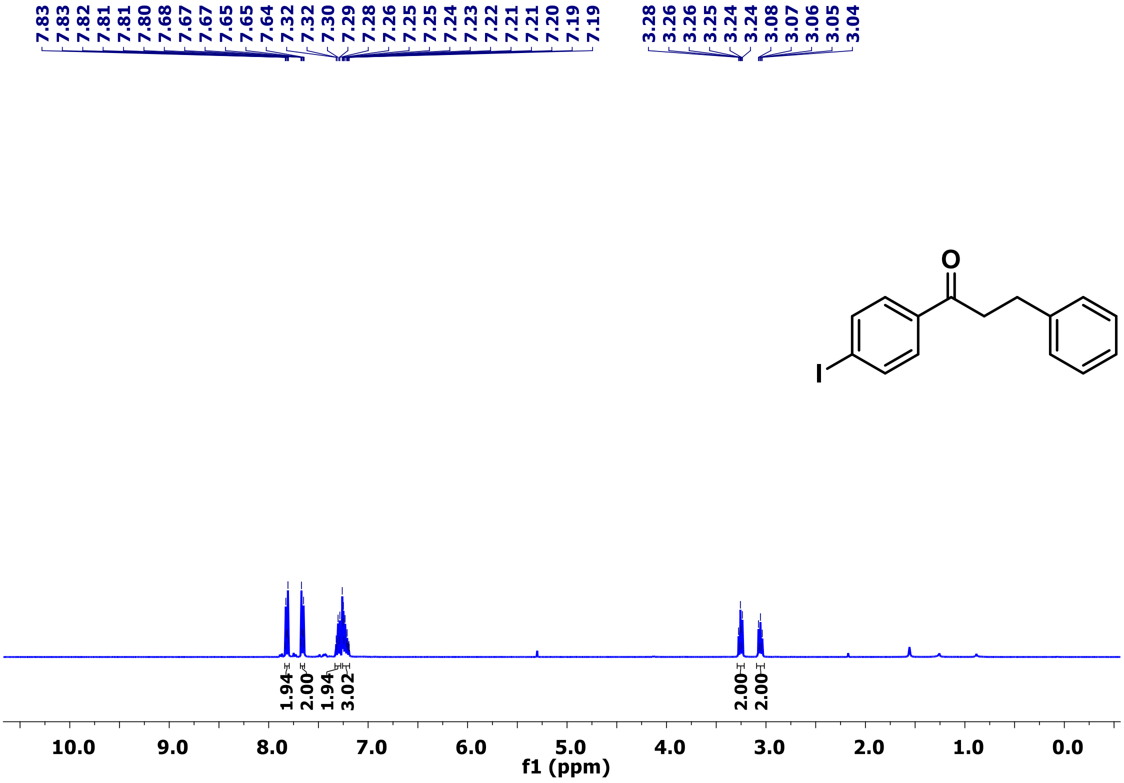

Figure S121: ^{1}H NMR spectrum (400 MHz) of **6ab** in $CDCl_3$

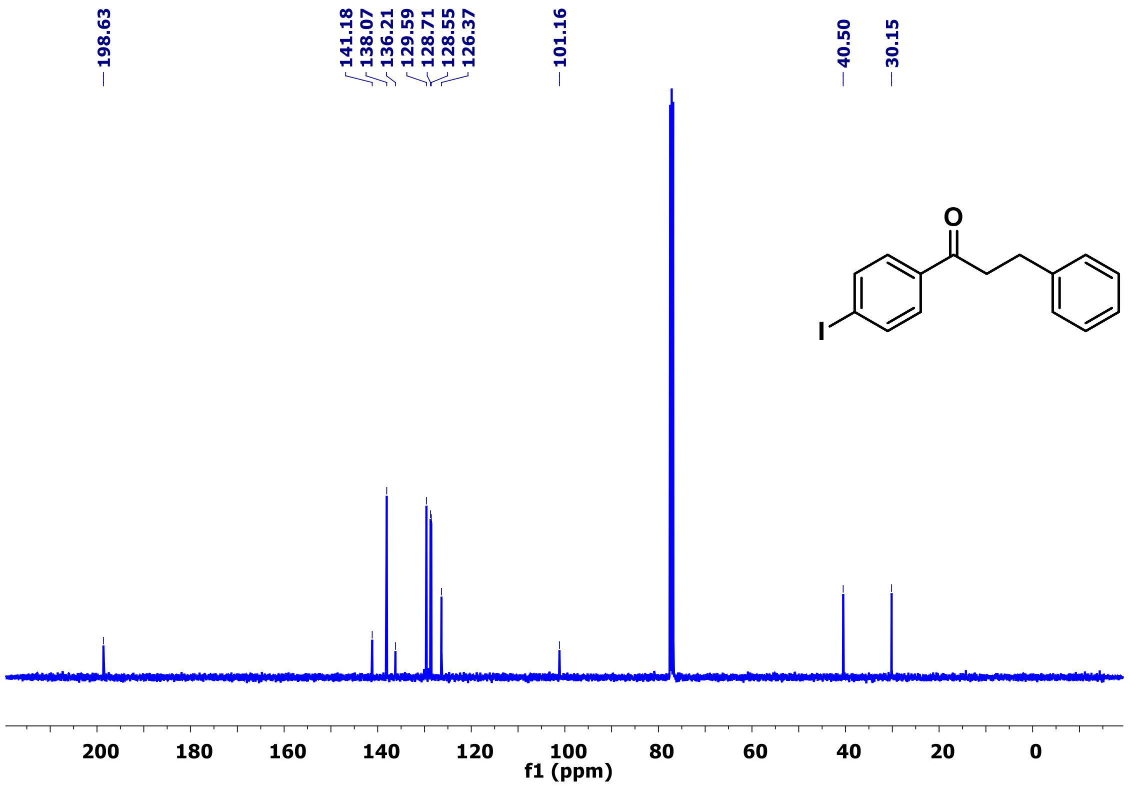

Figure S122: $^{13}C\{^{1}H\}$ NMR spectrum (101 MHz) of **6ab** in $CDCl_3$

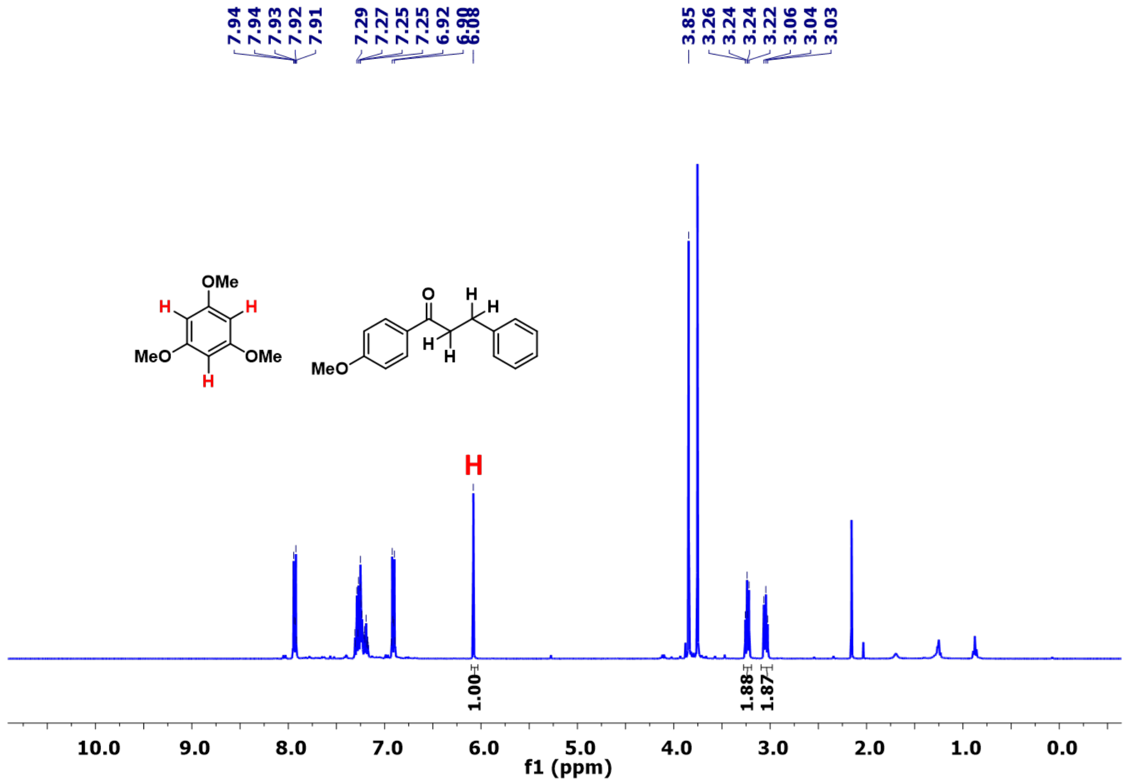

Figure S123: ^{1}H NMR spectrum (400 MHz, $CDCl_3$) of crude product mixture with 1/3 equiv. 1,3,5-trimethoxybenzene after hydrogenation of **5ac**

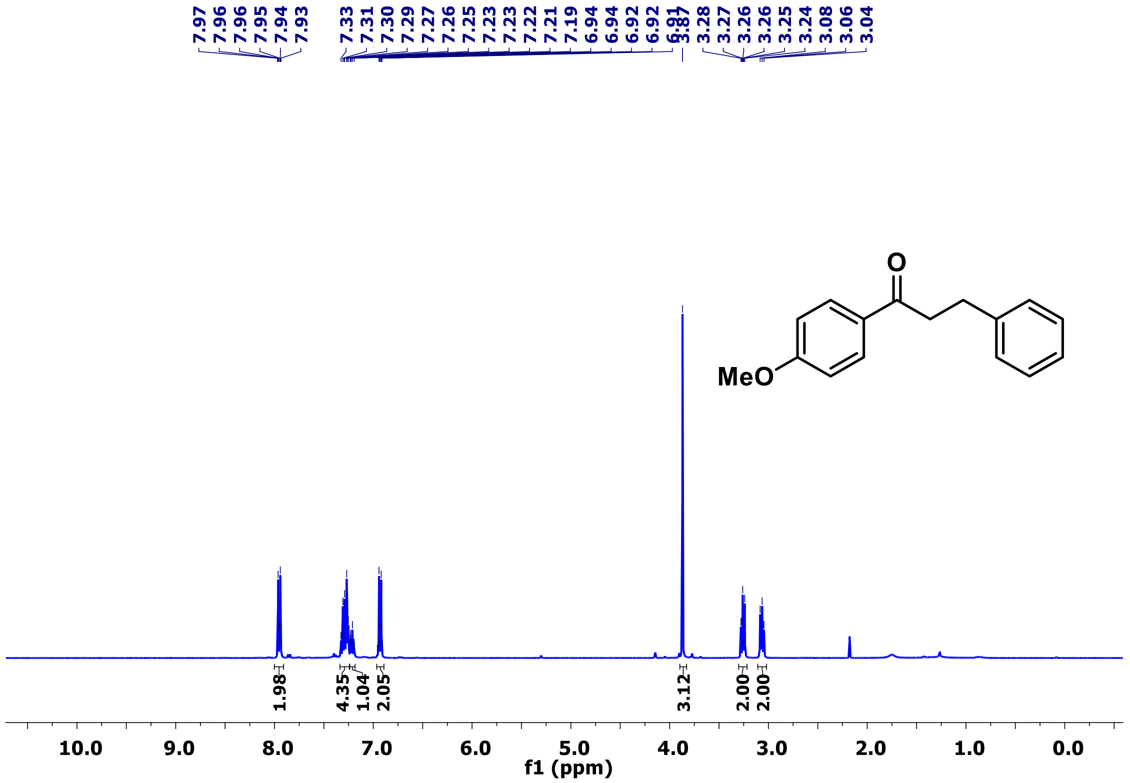

Figure S124: ^{1}H NMR spectrum (400 MHz) of **6ac** in $CDCl_3$

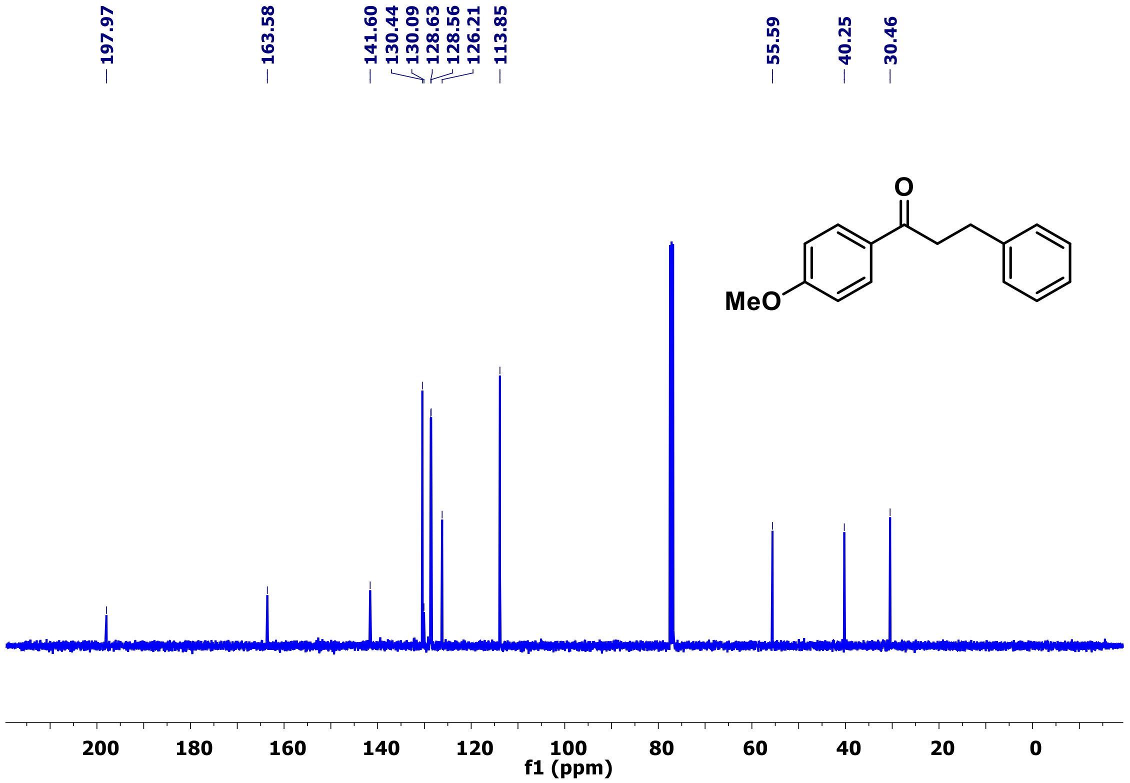

Figure S125: $^{13}C\{^{1}H\}$ NMR spectrum (101 MHz) of **6ac** in $CDCl_3$

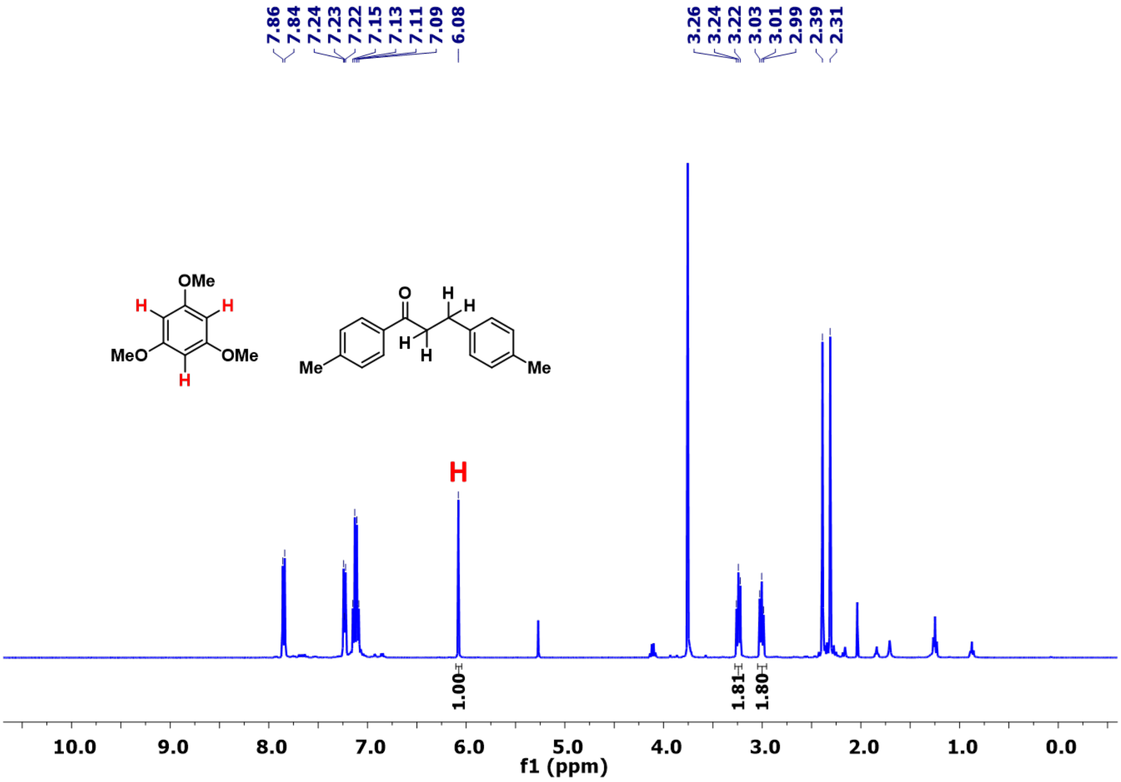

Figure S126: ^{1}H NMR spectrum (400 MHz, $CDCl_3$) of crude product mixture with 1/3 equiv. 1,3,5-trimethoxybenzene after hydrogenation of **5ad**

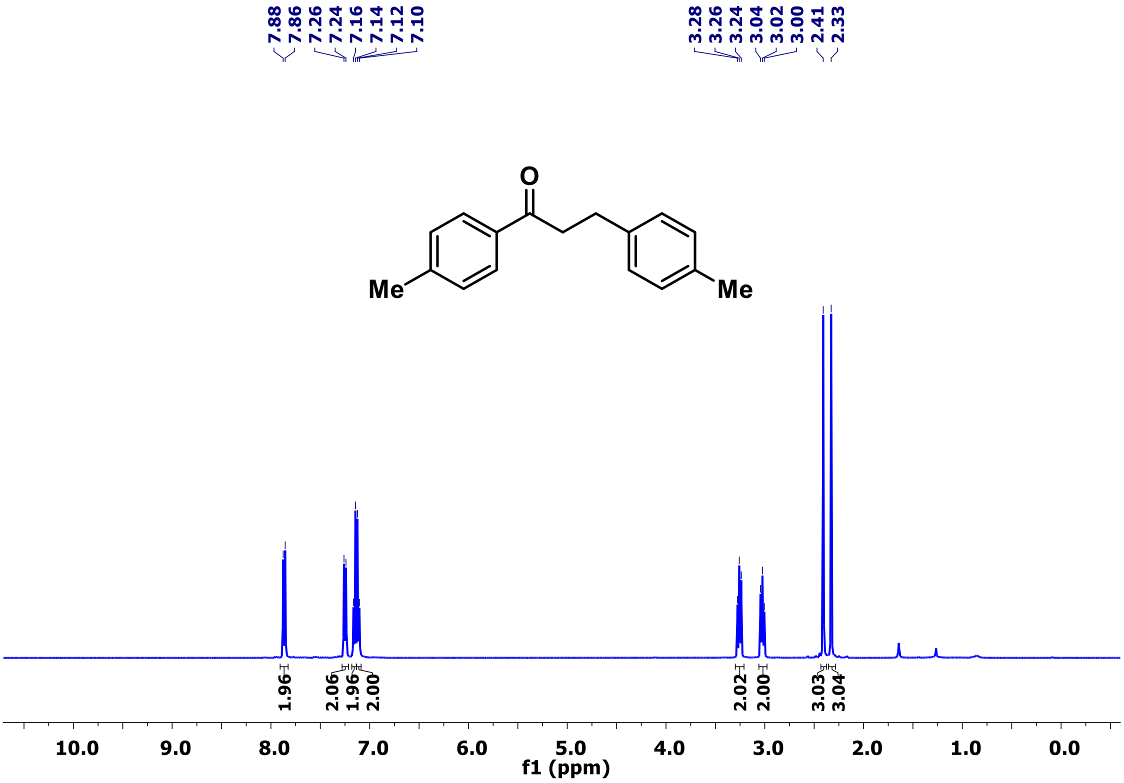

Figure S127: ^{1}H NMR spectrum (400 MHz) of **6ad** in $CDCl_3$

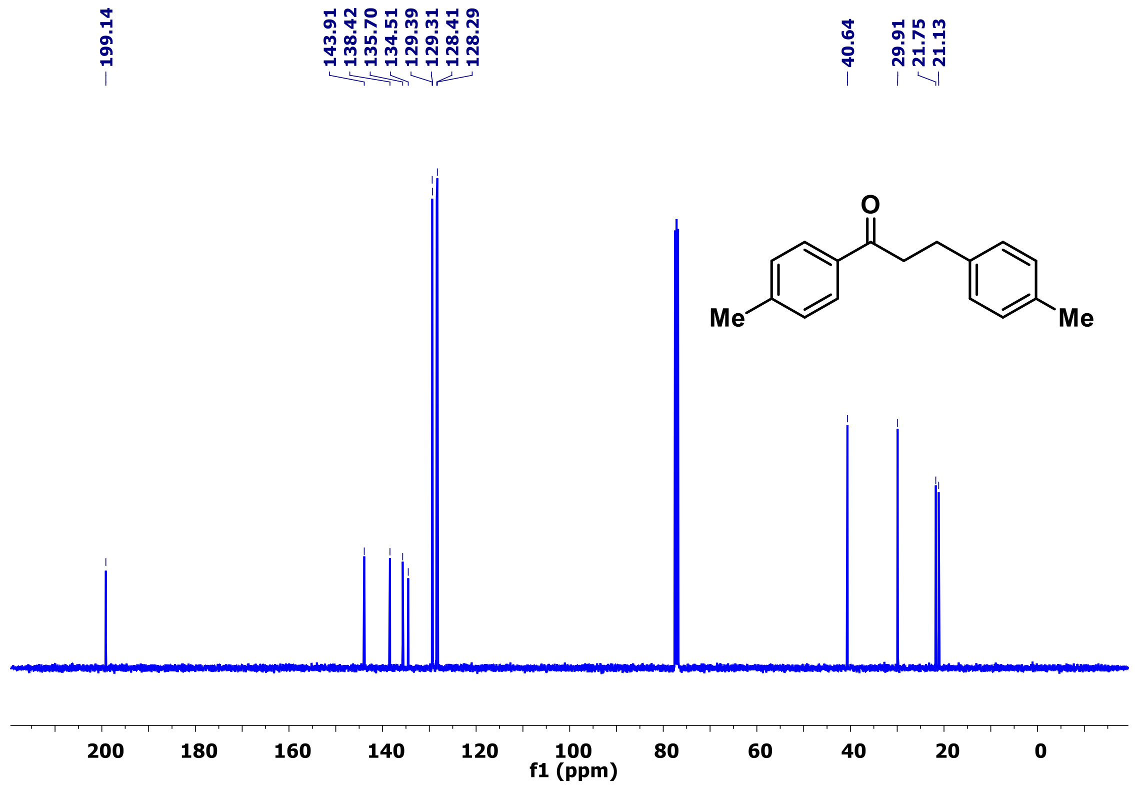

Figure S128: $^{13}C\{^{1}H\}$ NMR spectrum (101 MHz) of **6ad** in $CDCl_3$

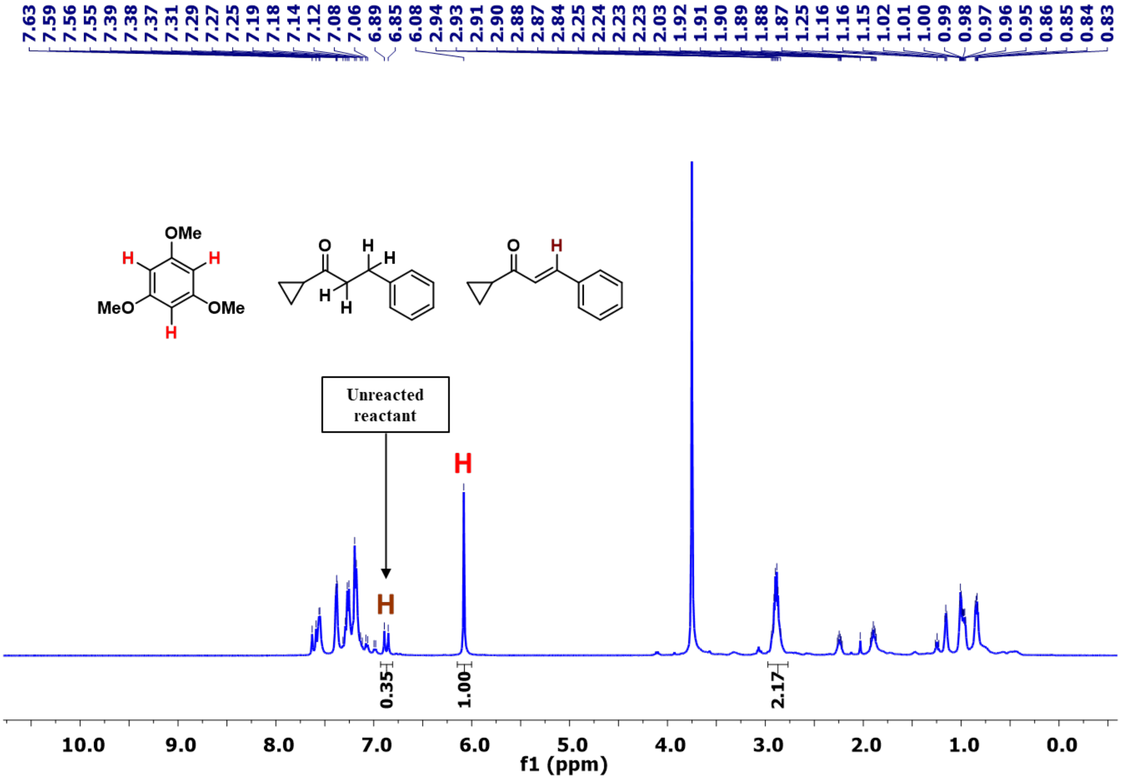

Figure S129: ^{1}H NMR spectrum (400 MHz, $CDCl_3$) of crude product mixture with 1/3 equiv. 1,3,5-trimethoxybenzene after hydrogenation of **5ae**.

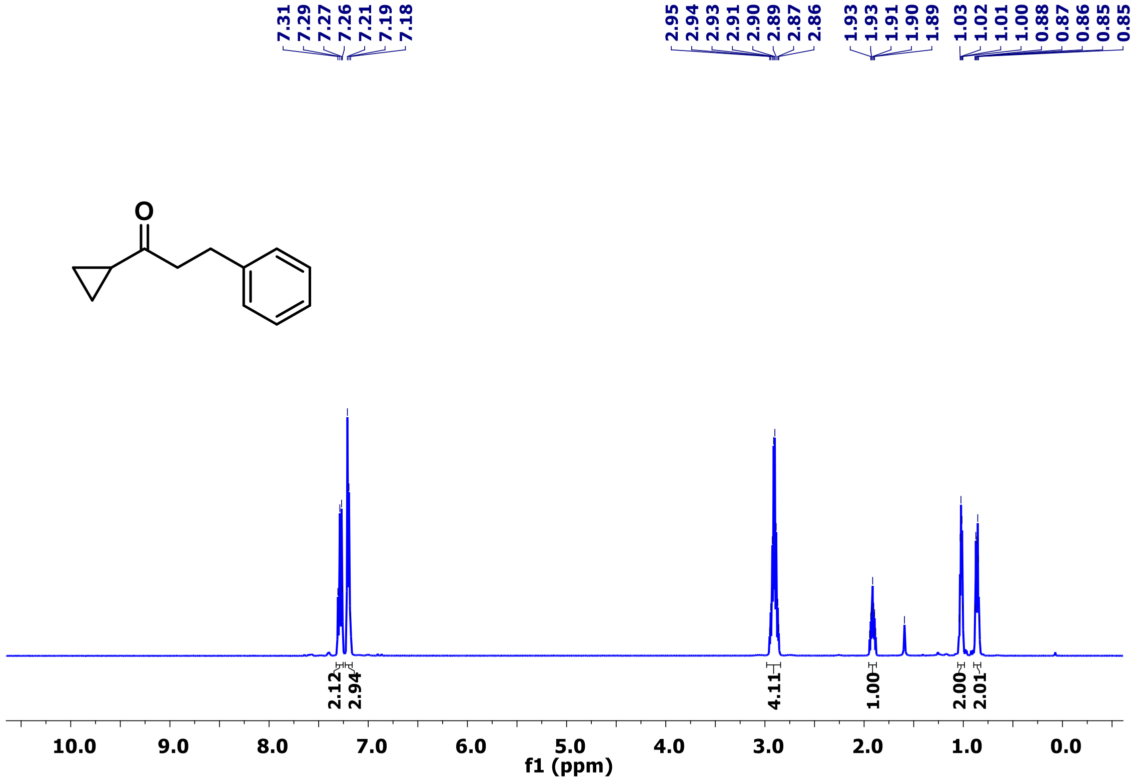

Figure S130: ^{1}H NMR spectrum (400 MHz) of **6ae** in $CDCl_3$

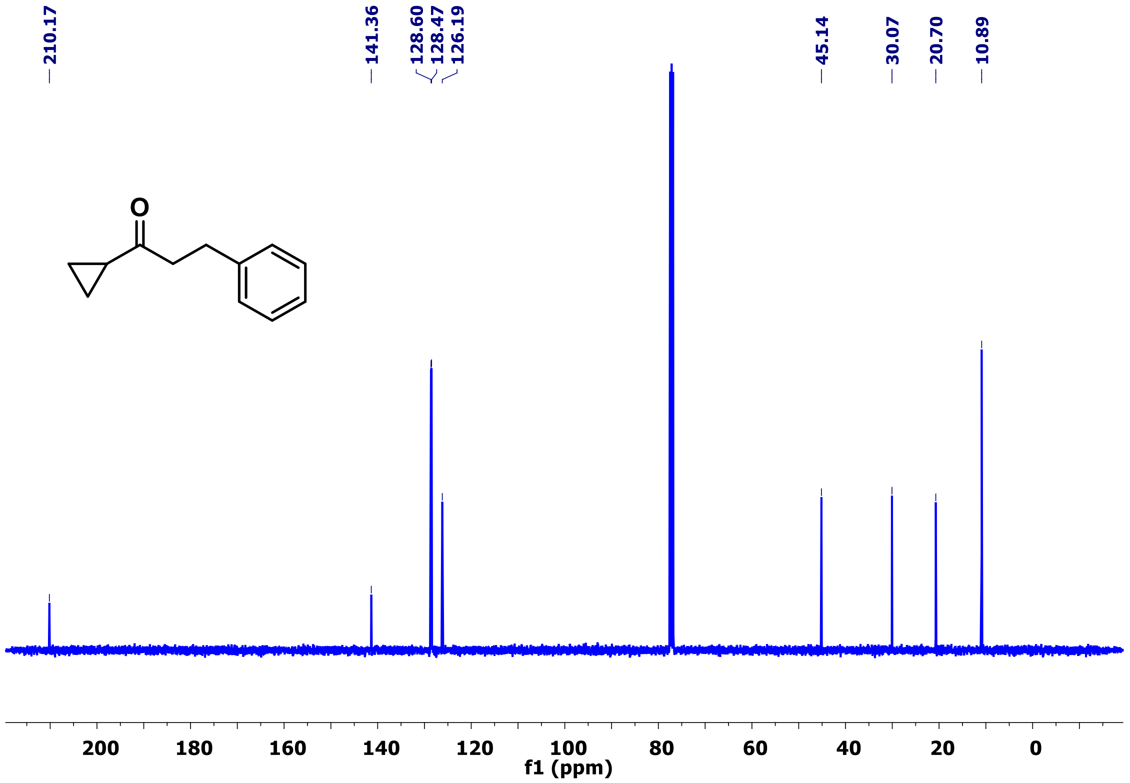

Figure S131: $^{13}C\{^{1}H\}$ NMR spectrum (101 MHz) of **6ae** in $CDCl_3$

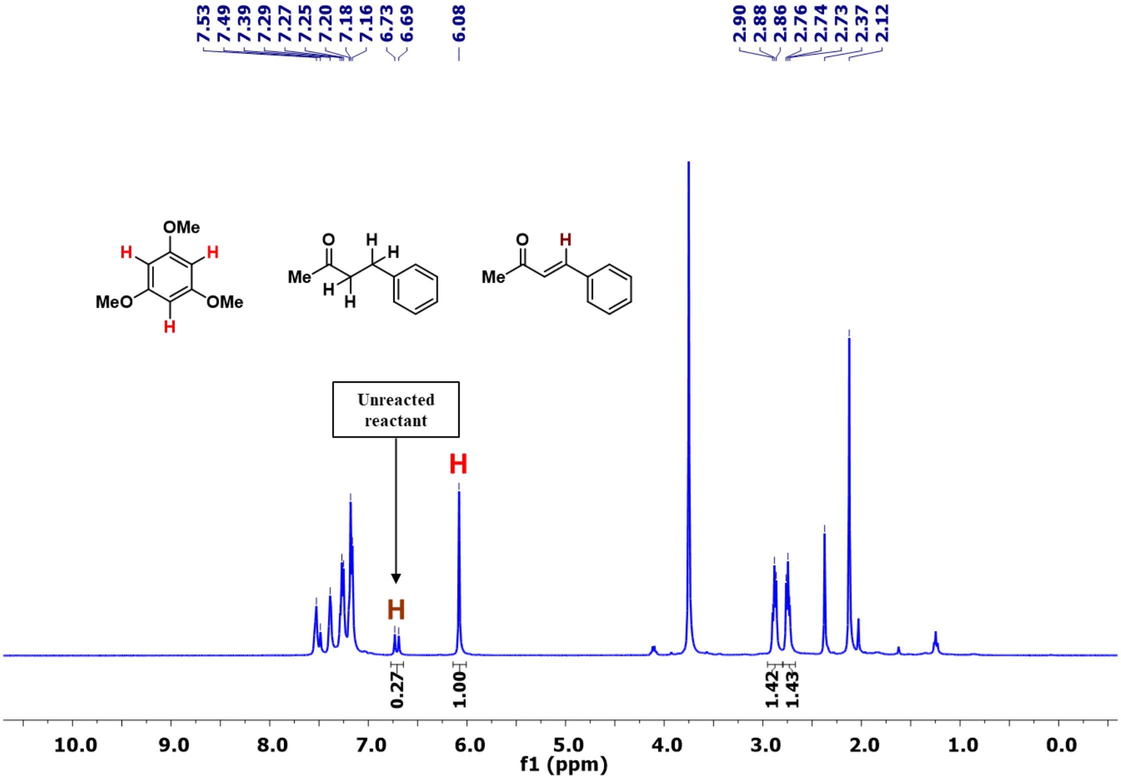

Figure S132: ^{1}H NMR spectrum (400 MHz, $CDCl_3$) of crude product mixture with 1/3 equiv. 1,3,5-trimethoxybenzene after hydrogenation of **5af**.

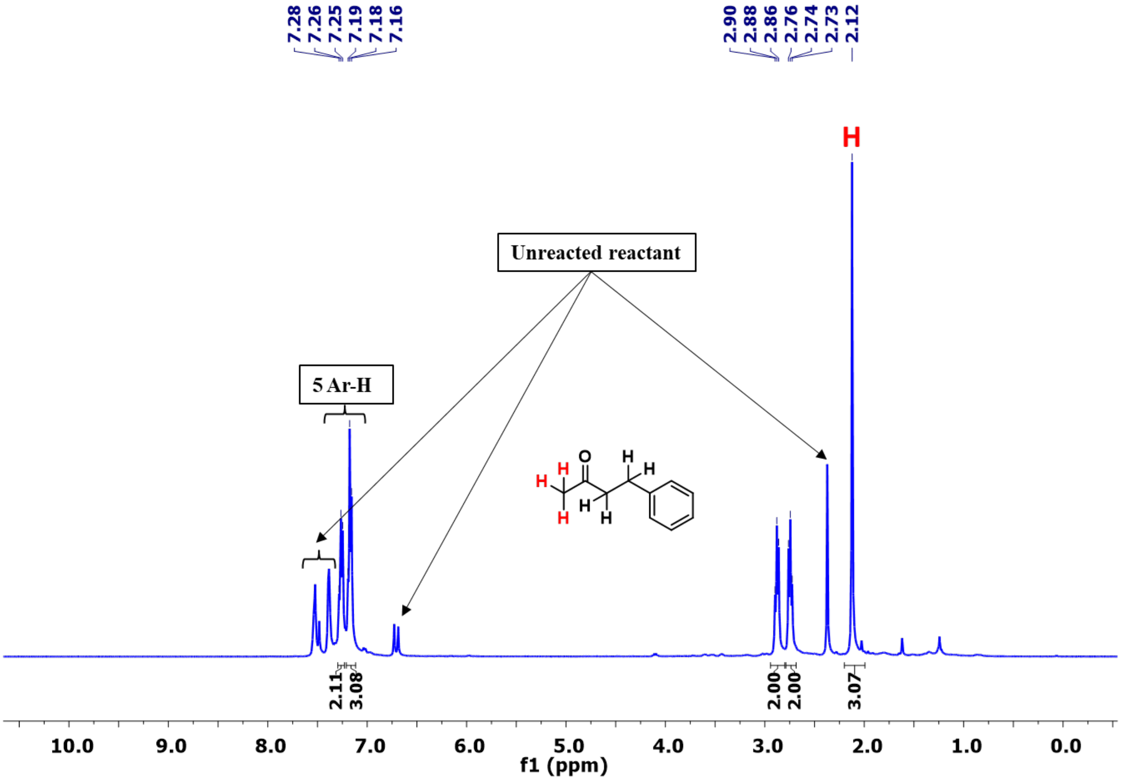

Figure S133: ^{1}H NMR spectrum (400 MHz) of crude product mixture **6af** in $CDCl_3$

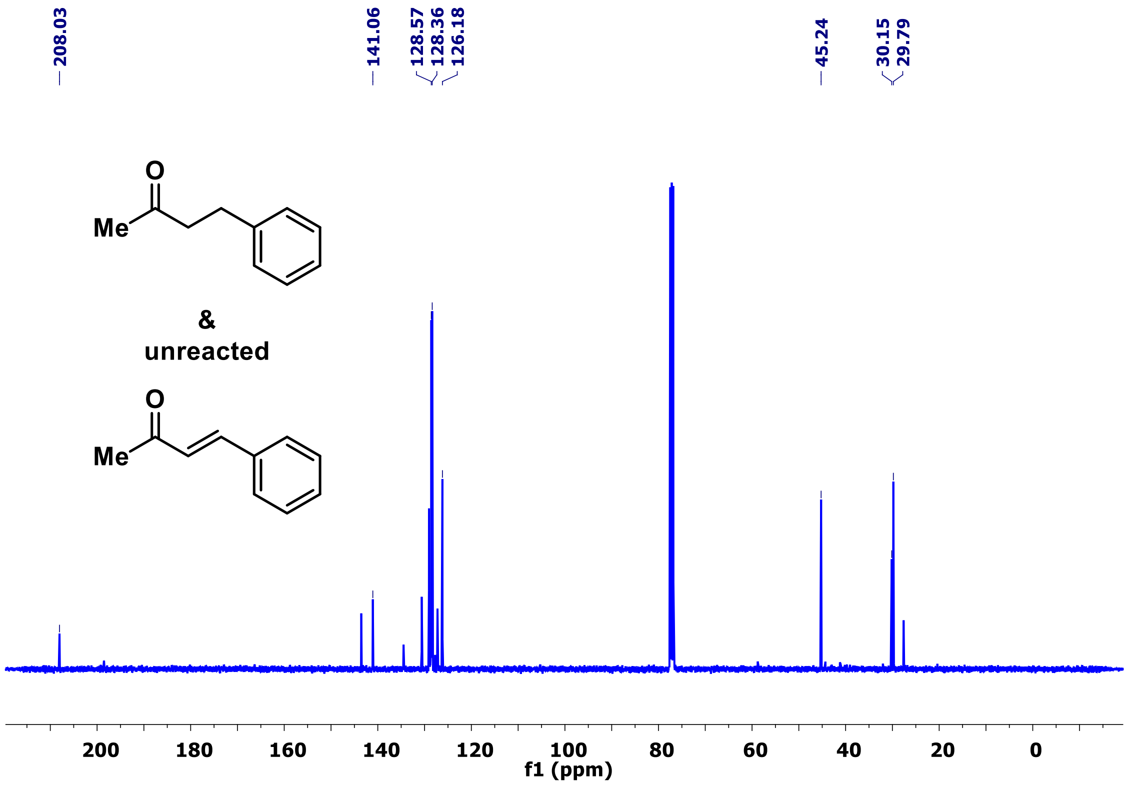

Figure S134: $^{13}C\{^{1}H\}$ NMR spectrum (101 MHz) of crude product mixture **6af** in $CDCl_3$. Unpicked peaks are of the remaining starting material.

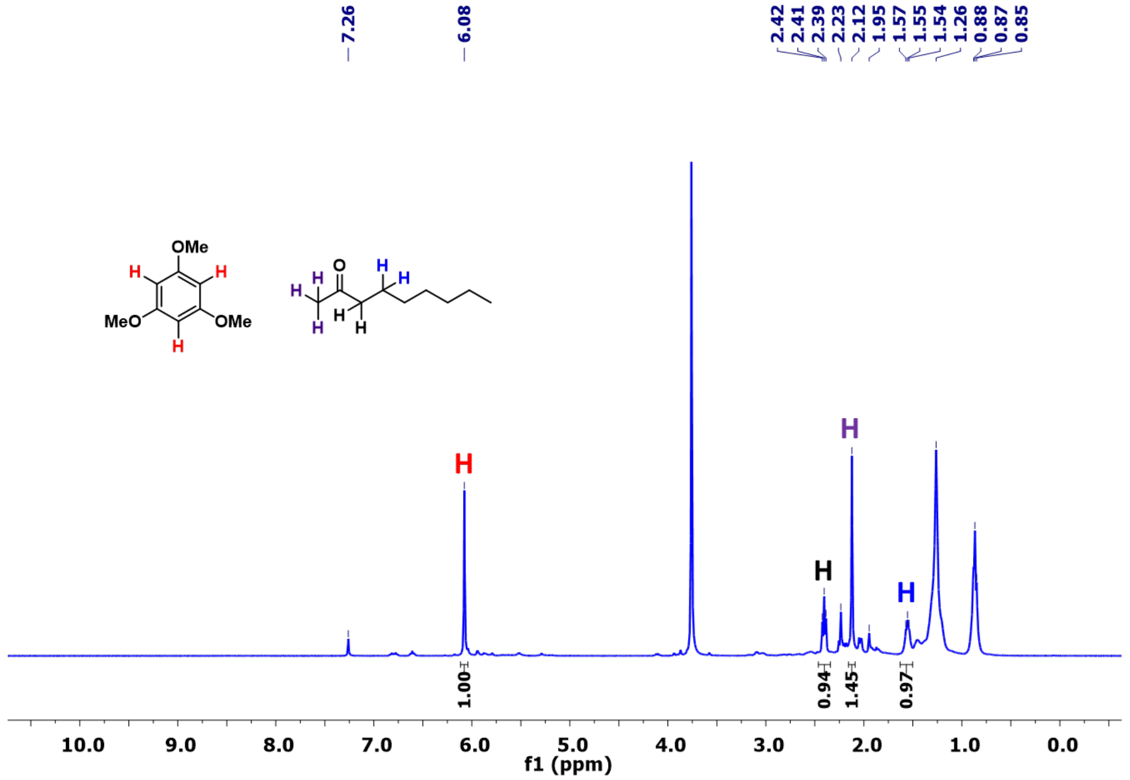

Figure S135: ^{1}H NMR spectrum (400 MHz, $CDCl_3$) of crude product mixture with 1/3 equiv. 1,3,5-trimethoxybenzene after hydrogenation of **5ah**.

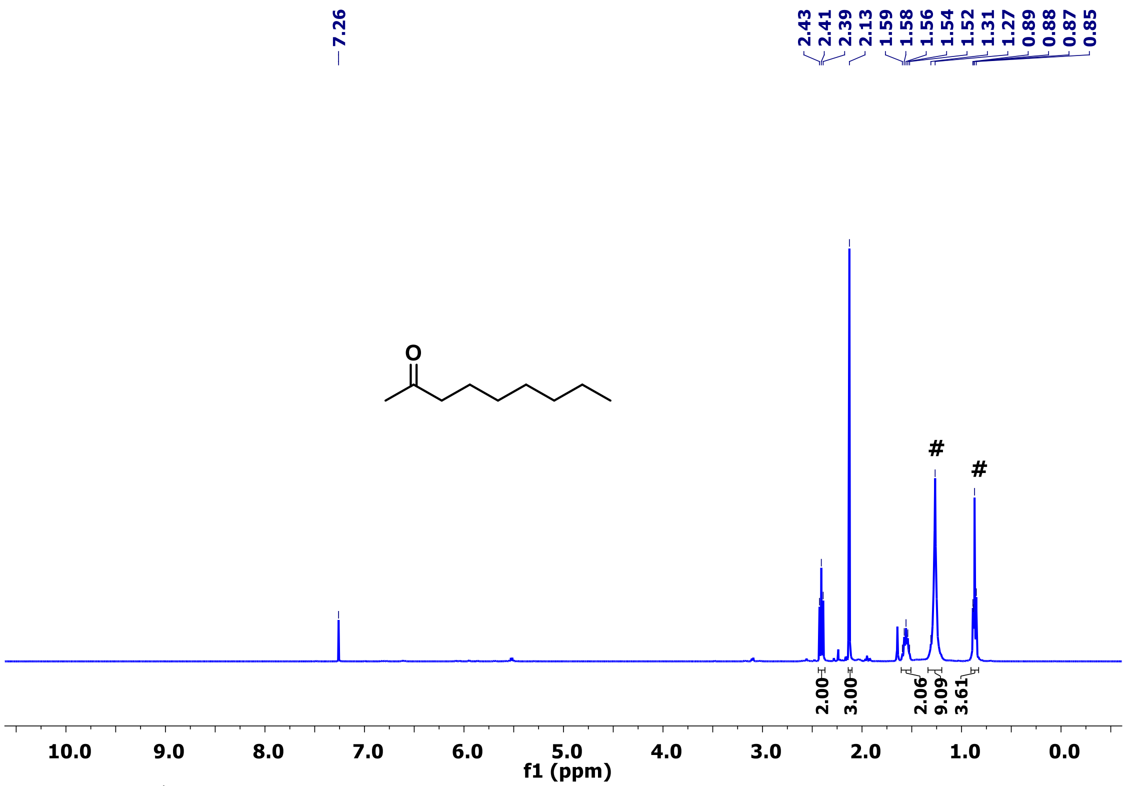

Figure S136: ^{1}H NMR spectrum (400 MHz) of **6ah** in $CDCl_3$. Trace residual n-hexane resonances are merged with # marked peaks.

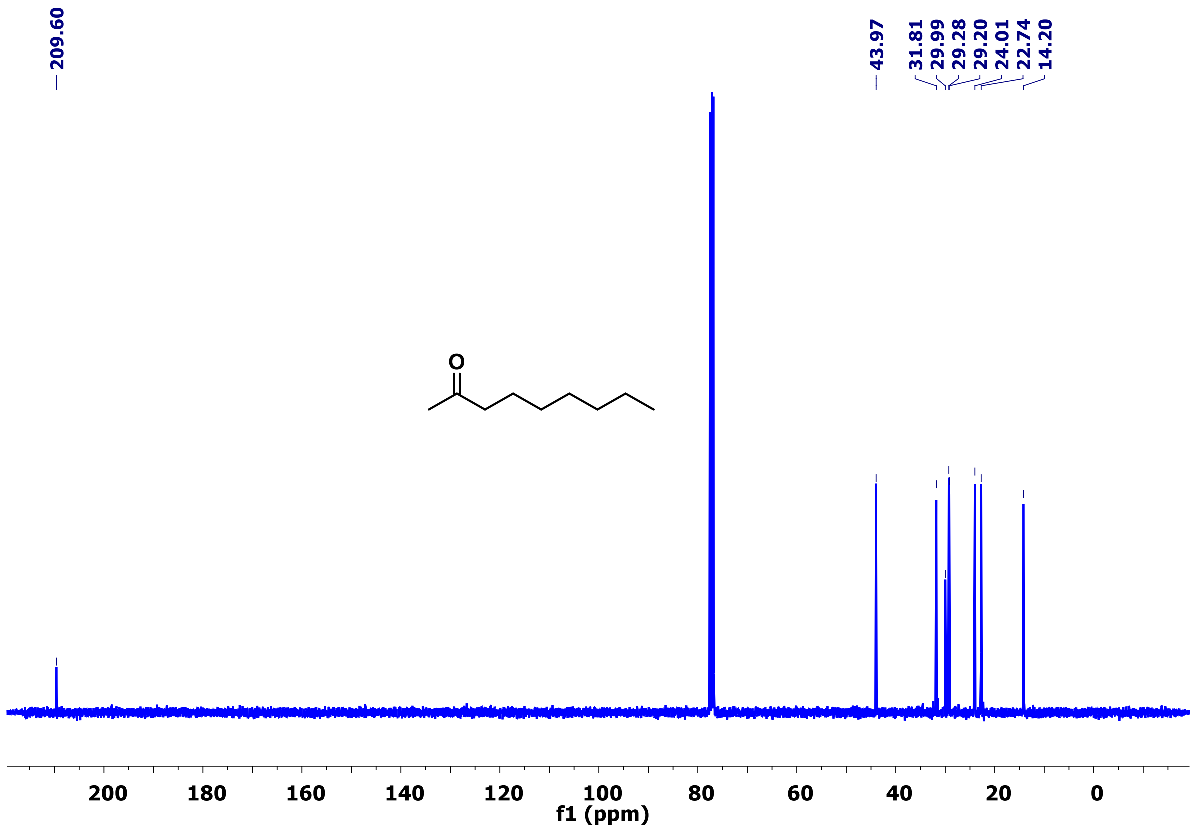

Figure S137: $^{13}C\{^{1}H\}$ NMR spectrum (101 MHz) of **6ah** in $CDCl_3$

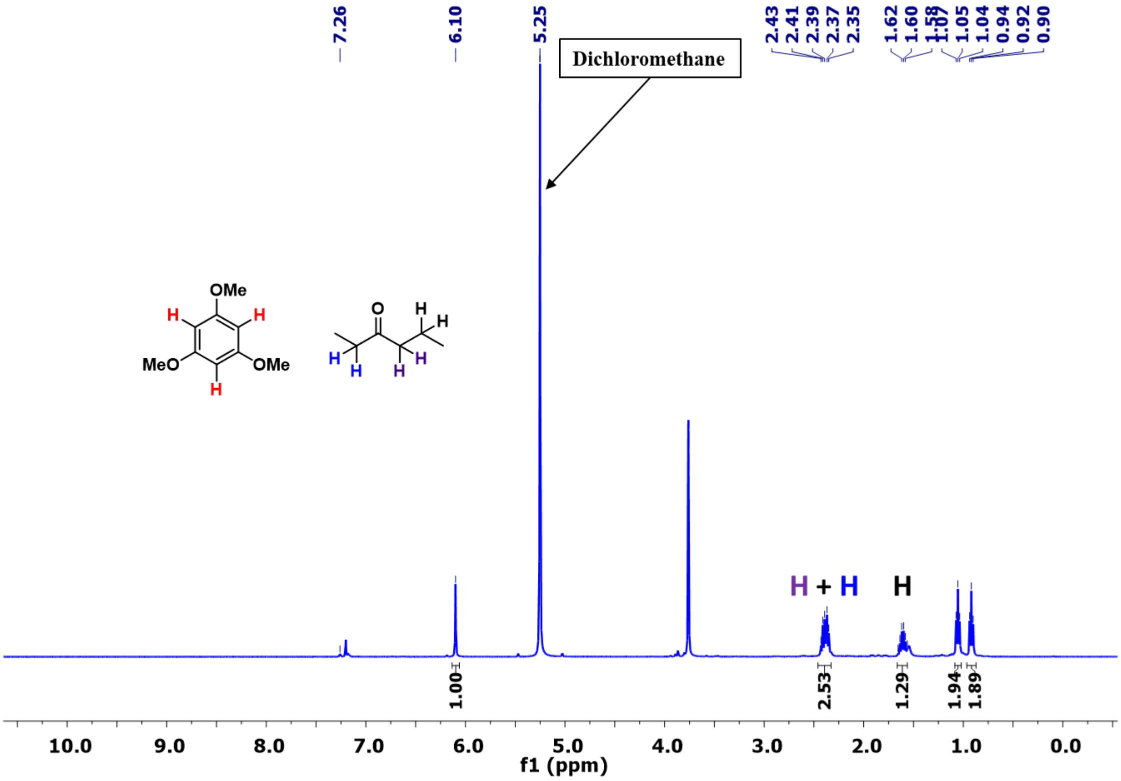

Figure S138: ^{1}H NMR spectrum (400 MHz, $CDCl_3$) of crude product mixture with 1/3 equiv. 1,3,5-trimethoxybenzene after hydrogenation of **5ai**.

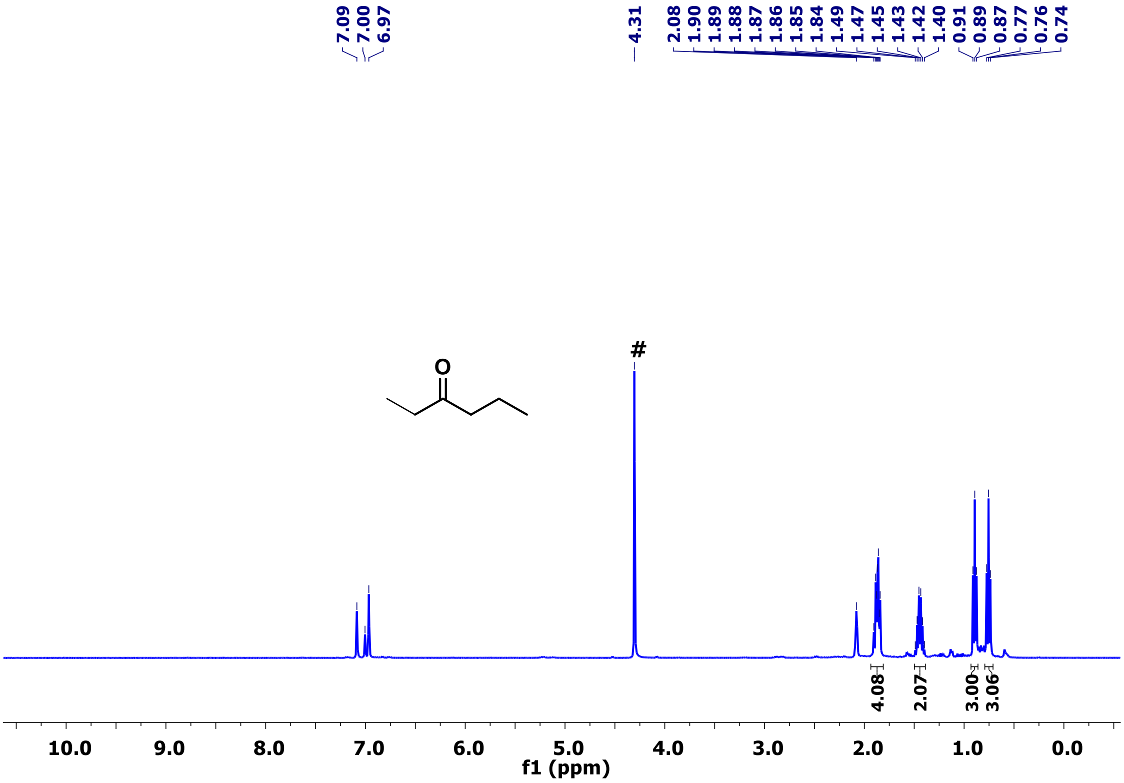

Figure S139: ^{1}H NMR spectrum (400 MHz) of **6ai** in Toluene-d_8. # marked peak corresponds to residual dichloromethane in the product mixture.

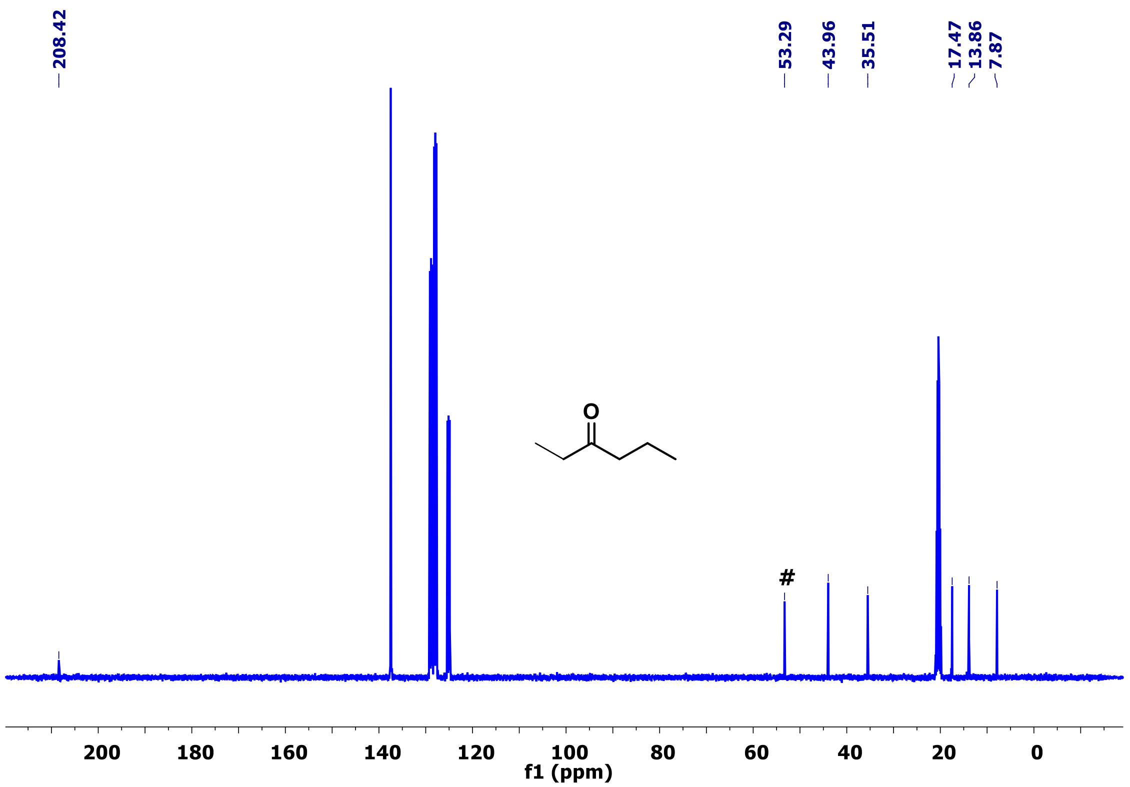

Figure S140: $^{13}C\{^{1}H\}$ NMR spectrum (101 MHz) of **6ai** in Toluene-d_8. # marked peak corresponds to residual dichloromethane in the product mixture.

Yield analysis of product 6ag by Gas Chromatography:

Determination of response factor (R_f) from GC calibration graph:
Five known samples containing 1:1 cyclohexanone and internal standard dodecane were prepared. The concentrations of both cyclohexanone and dodecane in those five standard solutions were 1 mM, 2 mM, 3 mM, 4 mM, and 5 mM. Ethyl acetate was used as solvent to prepare these solutions. Hereafter, the samples were run (**Figure S143** to **Figure S147**) and the area under cyclohexanone (t_R = 5.38 mins) was plotted against the area of dodecane (t_R = 8.03 mins).

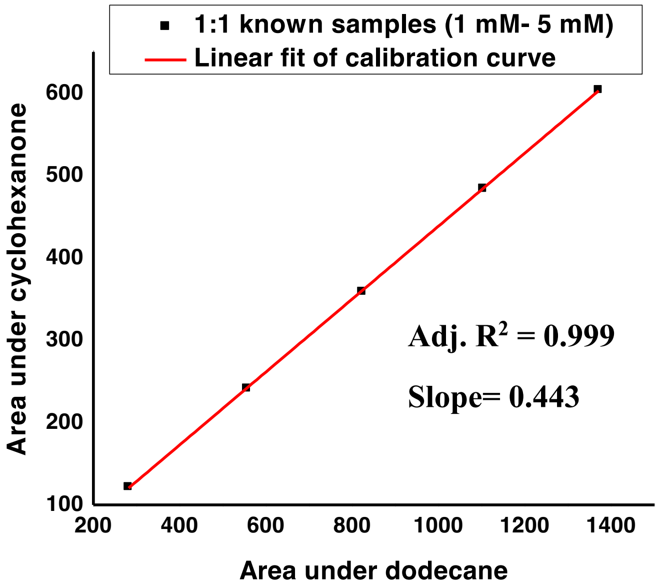

Figure S141: GC calibration graph of cyclohexanone with dodecane as internal standard

Calculation of yield from the crude reaction mixture:
After separation of organic products from catalyst following general procedure of hydrogenation of enones, 45.4 µL (0.2 mmol) dodecane was added and the total volume of the solution was made to 40 mL.

Now, $area_{analyte}/mmol_{analyte} = R_f * area_{Int.\ stand.}/mmol_{Int.\ stand.}$

Therefore, $mmol_{analyte} = area_{analyte} * mmol_{Int.\ stand.}/R_f * area_{\ Int.\ stand.}$

= 359.46 * 0.2./0.44 * 1407.25 (R_f = slope of the calibration graph = 0.44, and areas from **Figure S142**)

= 0.1161

Therefore, % of yield = 0.1161 *100/0.2

= 58.05%

```
Data File C:\CHEM32\1\DATA\TEREZA\2023-12-07-TIME-15-25-28\206B0601.D
Sample Name: Crude mixr

    =====================================================================
    Acq. Operator   : SYSTEM                          Seq. Line :   6
    Acq. Instrument : GC7820                           Location : Vial 206
    Injection Date  : 07/12/2023 17:23:49                   Inj :   1
                                                     Inj Volume : 1 µl
    Different Inj Volume from Sequence !     Actual Inj Volume : 5 µl
    Acq. Method     : C:\CHEM32\1\DATA\TEREZA\2023-12-07-TIME-15-25-28\50-15-300NEW.M
    Last changed    : 07/12/2023 15:25:28 by SYSTEM
    Analysis Method : C:\CHEM32\1\METHODS\80-10-300.M
    Last changed    : 07/12/2023 18:19:46 by SYSTEM
                      (modified after loading)
    Additional Info : Peak(s) manually integrated
```

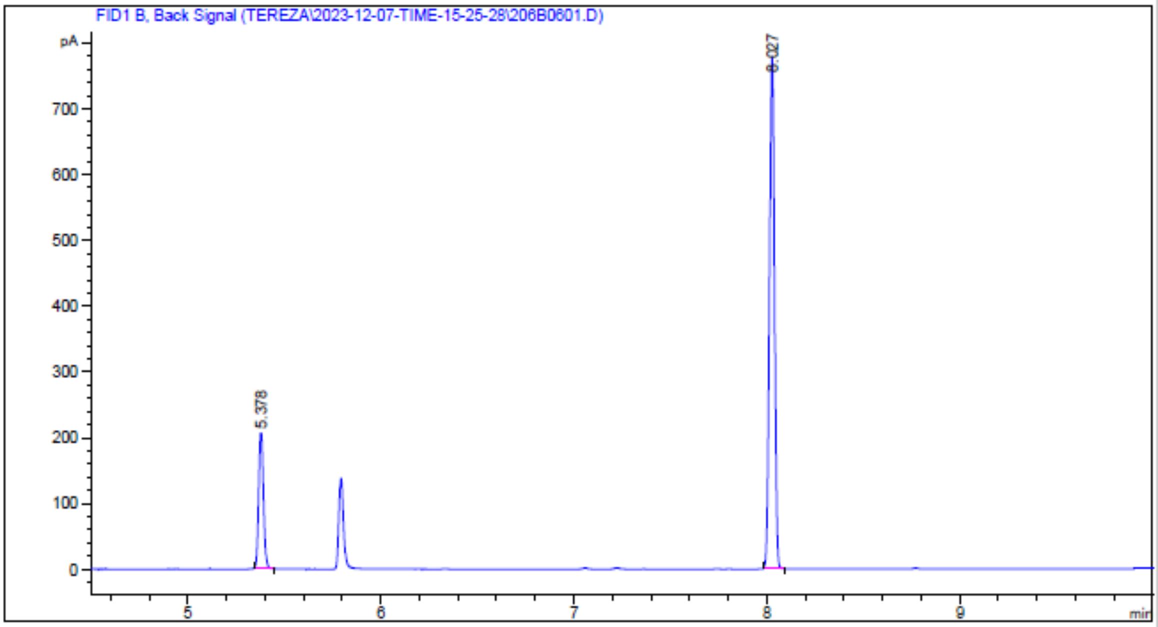


```
    =====================================================================
                             Area Percent Report
    =====================================================================

    Sorted By             :      Signal
    Multiplier            :      1.0000
    Dilution              :      1.0000
    Do not use Multiplier & Dilution Factor with ISTDs


    Signal 1: FID1 B, Back Signal

    Peak RetTime Type  Width     Area      Height     Area
      #   [min]        [min]   [pA*s]      [pA]        %
    ----|-------|----|-------|----------|----------|--------|
       1   5.378 BB    0.0273  359.45758  206.38971 20.34614
       2   8.027 BB    0.0291 1407.25427  776.67072 79.65386

    Totals :                  1766.71185  983.06042
```

Figure S142: Gas chromatography spectrum of crude reaction mixture after hydrogenation of 5ae (0.2 mmol dodecane as internal standard)

Data File C:\CHEM32\1\DATA\TEREZA\2023-12-07-TIME-15-25-28\201B0101.D
Sample Name: 1 mM

===
Acq. Operator : SYSTEM Seq. Line : 1
Acq. Instrument : GC7820 Location : Vial 201
Injection Date : 07/12/2023 15:28:15 Inj : 1
Inj Volume : 1 µl
Different Inj Volume from Sequence ! Actual Inj Volume : 5 µl
Acq. Method : C:\CHEM32\1\DATA\TEREZA\2023-12-07-TIME-15-25-28\50-15-300NEW.M
Last changed : 07/12/2023 15:25:28 by SYSTEM
Analysis Method : C:\CHEM32\1\METHODS\80-10-300.M
Last changed : 07/12/2023 18:11:29 by SYSTEM
(modified after loading)
Additional Info : Peak(s) manually integrated

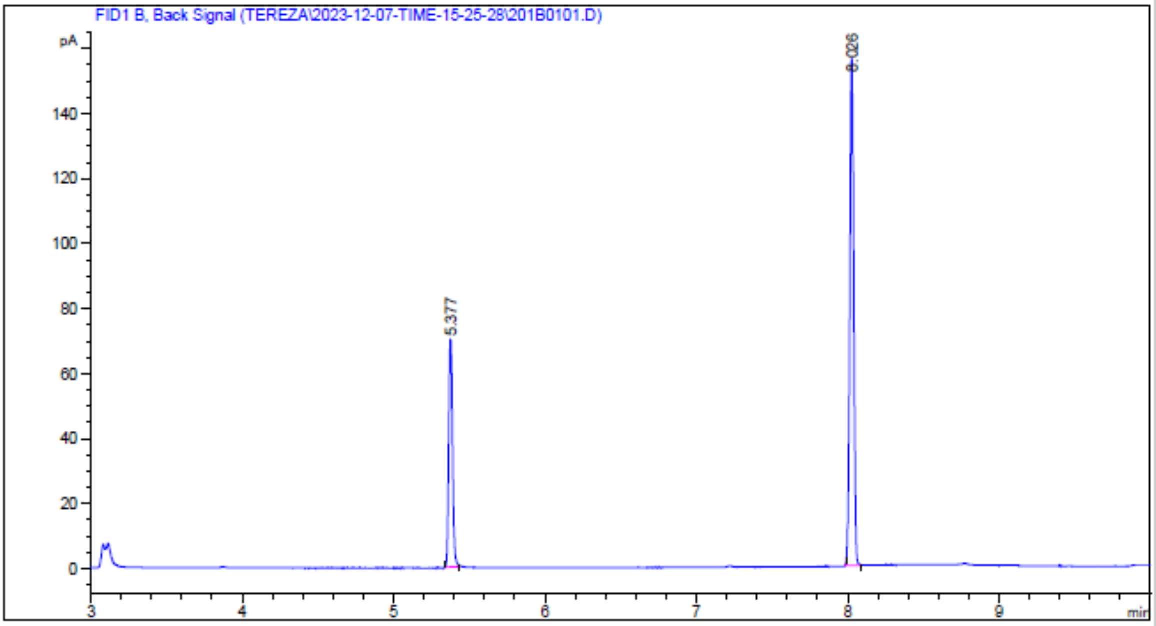

===
Area Percent Report
===

Sorted By : Signal
Multiplier : 1.0000
Dilution : 1.0000
Do not use Multiplier & Dilution Factor with ISTDs

Signal 1: FID1 B, Back Signal

Peak #	RetTime [min]	Type	Width [min]	Area [pA*s]	Height [pA]	Area %
1	5.377	BB	0.0274	121.57303	69.61977	30.16206
2	8.026	BB	0.0291	281.49310	155.62616	69.83794

Totals : 403.06613 225.24593

Figure S143: Gas chromatography spectrum of standard sample containing 1 mM cyclohexanone and 1 mM dodecane

```
Data File C:\CHEM32\1\DATA\TEREZA\2023-12-07-TIME-15-25-28\202B0201.D
Sample Name: 2 mM

    =====================================================================
    Acq. Operator   : SYSTEM                          Seq. Line :   2
    Acq. Instrument : GC7820                           Location : Vial 202
    Injection Date  : 07/12/2023 15:51:10                   Inj :   1
                                                    Inj Volume : 1 µl
    Different Inj Volume from Sequence !     Actual Inj Volume : 5 µl
    Acq. Method     : C:\CHEM32\1\DATA\TEREZA\2023-12-07-TIME-15-25-28\50-15-300NEW.M
    Last changed    : 07/12/2023 15:25:28 by SYSTEM
    Analysis Method : C:\CHEM32\1\METHODS\80-10-300.M
    Last changed    : 07/12/2023 18:11:29 by SYSTEM
                      (modified after loading)
    Additional Info : Peak(s) manually integrated
```

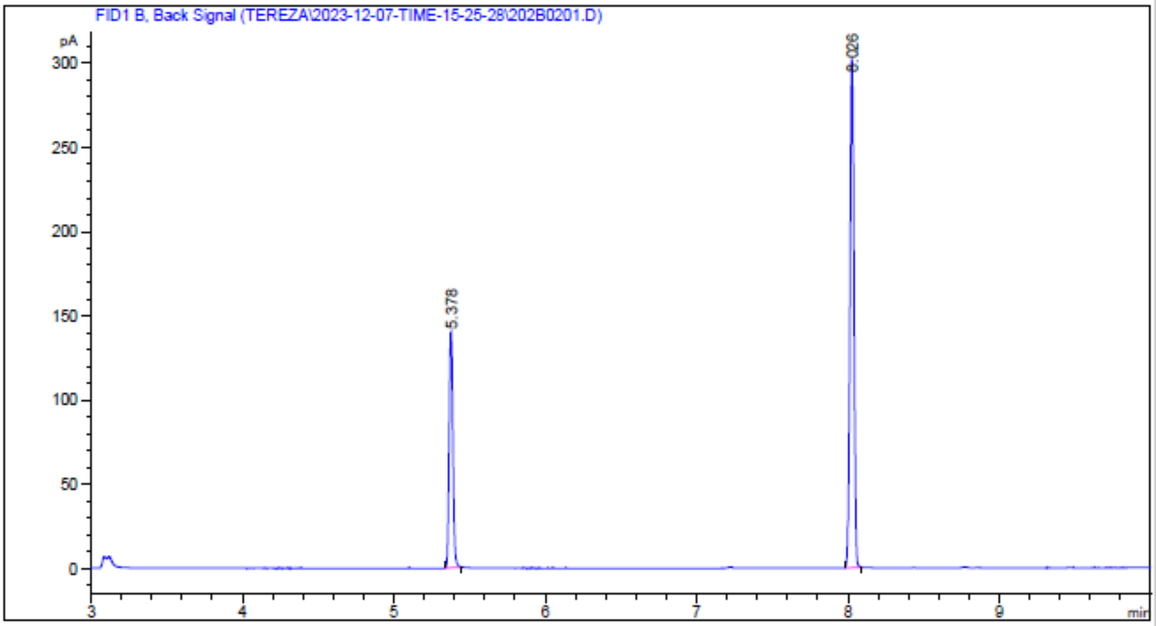


```
    =====================================================================
                         Area Percent Report
    =====================================================================

    Sorted By             :      Signal
    Multiplier            :      1.0000
    Dilution              :      1.0000
    Do not use Multiplier & Dilution Factor with ISTDs


    Signal 1: FID1 B, Back Signal

    Peak RetTime Type  Width     Area      Height     Area
      #   [min]        [min]   [pA*s]      [pA]         %
    ----|-------|----|-------|----------|----------|--------|
       1   5.378 BB    0.0272  241.18365  139.38747 30.25987
       2   8.026 BB    0.0295  555.85754  301.75519 69.74013

    Totals :                   797.04120  441.14265
```

Figure S144: Gas chromatography spectrum of standard sample containing 2 mM cyclohexanone and 2 mM dodecane

```
Data File C:\CHEM32\1\DATA\TEREZA\2023-12-07-TIME-15-25-28\203B0301.D
Sample Name: 3 mM

    =====================================================================
    Acq. Operator   : SYSTEM                            Seq. Line :    3
    Acq. Instrument : GC7820                             Location : Vial 203
    Injection Date  : 07/12/2023 16:14:11                     Inj :    1
                                                   Inj Volume : 1 µl
    Different Inj Volume from Sequence !     Actual Inj Volume : 5 µl
    Acq. Method     : C:\CHEM32\1\DATA\TEREZA\2023-12-07-TIME-15-25-28\50-15-300NEW.M
    Last changed    : 07/12/2023 15:25:28 by SYSTEM
    Analysis Method : C:\CHEM32\1\METHODS\80-10-300.M
    Last changed    : 07/12/2023 18:11:29 by SYSTEM
                      (modified after loading)
    Additional Info : Peak(s) manually integrated
```

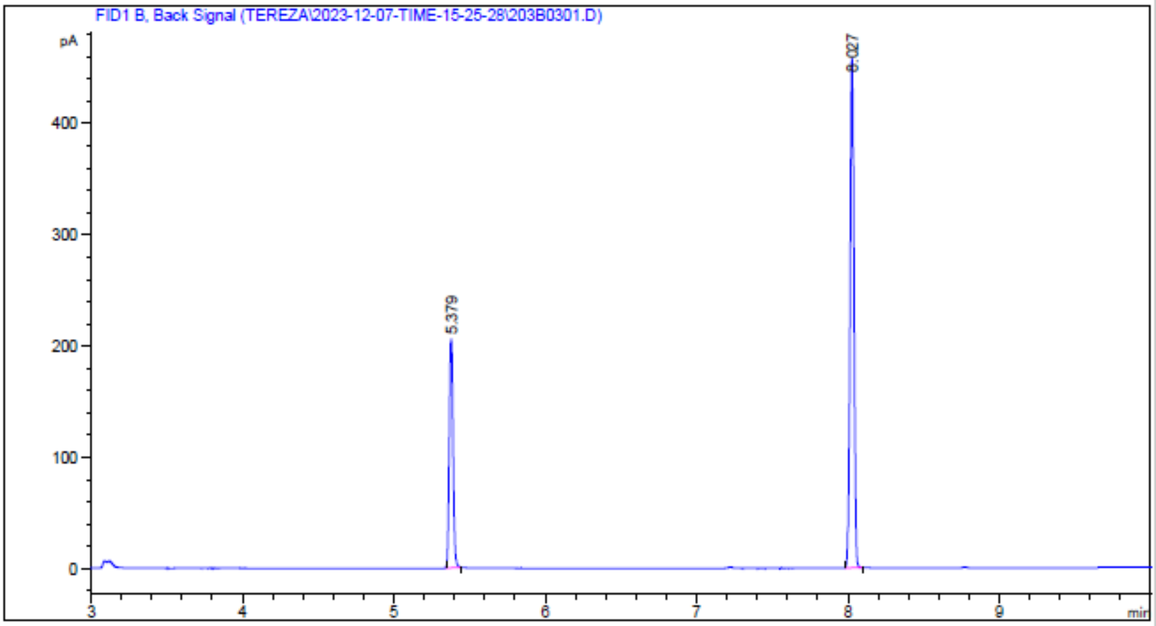


```
    =====================================================================
                             Area Percent Report
    =====================================================================

    Sorted By             :      Signal
    Multiplier            :      1.0000
    Dilution              :      1.0000
    Do not use Multiplier & Dilution Factor with ISTDs


    Signal 1: FID1 B, Back Signal

    Peak RetTime Type  Width     Area      Height     Area
      #   [min]        [min]   [pA*s]      [pA]         %
    ----|-------|----|-------|----------|----------|--------|
       1   5.379 BB    0.0273  358.78851  205.69531 30.35716
       2   8.027 BB    0.0290  823.10248  457.22656 69.64284

    Totals :                  1181.89099  662.92188
```

Figure S145: Gas chromatography spectrum of standard sample containing 3 mM cyclohexanone and 3 mM dodecane

```
Data File C:\CHEM32\1\DATA\TEREZA\2023-12-07-TIME-15-25-28\204B0401.D
Sample Name: 4 mM

    =====================================================================
    Acq. Operator   : SYSTEM                          Seq. Line :   4
    Acq. Instrument : GC7820                           Location : Vial 204
    Injection Date  : 07/12/2023 16:37:25                   Inj :   1
                                                     Inj Volume : 1 µl
    Different Inj Volume from Sequence !      Actual Inj Volume : 5 µl
    Acq. Method     : C:\CHEM32\1\DATA\TEREZA\2023-12-07-TIME-15-25-28\50-15-300NEW.M
    Last changed    : 07/12/2023 15:25:28 by SYSTEM
    Analysis Method : C:\CHEM32\1\METHODS\80-10-300.M
    Last changed    : 07/12/2023 18:11:29 by SYSTEM
                      (modified after loading)
    Additional Info : Peak(s) manually integrated
```

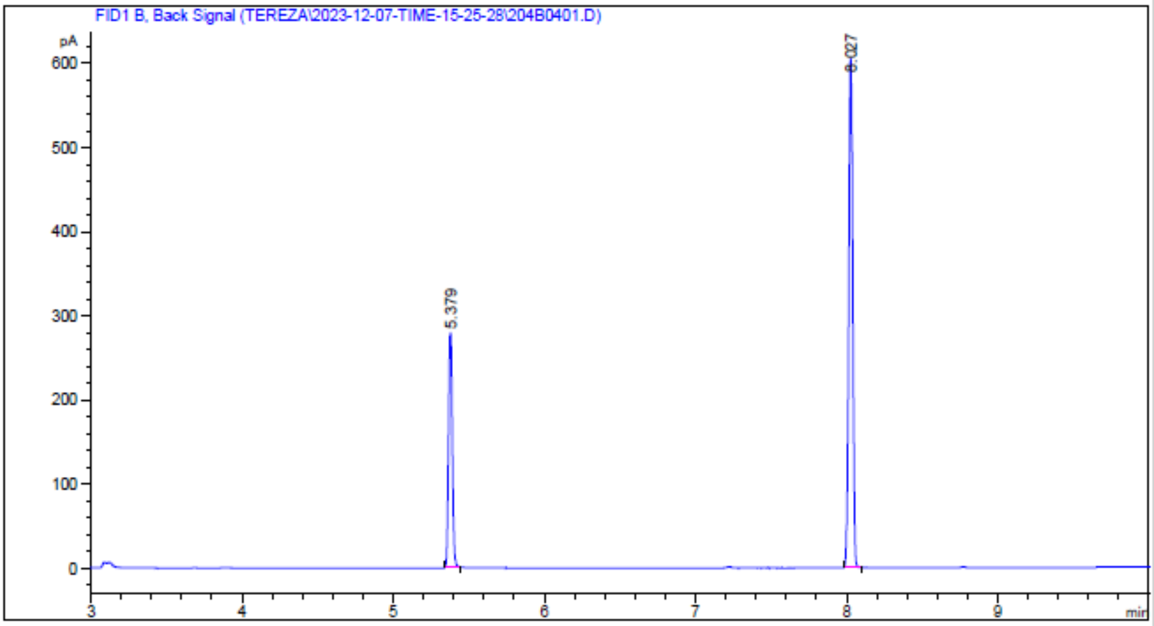


```
    =====================================================================
                             Area Percent Report
    =====================================================================

    Sorted By             :      Signal
    Multiplier            :      1.0000
    Dilution              :      1.0000
    Do not use Multiplier & Dilution Factor with ISTDs


    Signal 1: FID1 B, Back Signal

    Peak RetTime Type  Width     Area      Height     Area
      #   [min]        [min]    [pA*s]      [pA]        %
    ----|-------|----|-------|----------|----------|--------|
       1   5.379 BB    0.0272  483.98773  279.35880 30.49256
       2   8.027 BB    0.0293 1103.24463  605.64539 69.50744

    Totals :                   1587.23236  885.00418
```

Figure S146: Gas chromatography spectrum of standard sample containing 4 mM cyclohexanone and 4 mM dodecane

Data File C:\CHEM32\1\DATA\TEREZA\2023-12-07-TIME-15-25-28\205B0501.D
Sample Name: 5 mM

===

Acq. Operator : SYSTEM Seq. Line : 5
Acq. Instrument : GC7820 Location : Vial 205
Injection Date : 07/12/2023 17:00:35 Inj : 1
Inj Volume : 1 µl
Different Inj Volume from Sequence ! Actual Inj Volume : 5 µl
Acq. Method : C:\CHEM32\1\DATA\TEREZA\2023-12-07-TIME-15-25-28\50-15-300NEW.M
Last changed : 07/12/2023 15:25:28 by SYSTEM
Analysis Method : C:\CHEM32\1\METHODS\80-10-300.M
Last changed : 07/12/2023 18:11:29 by SYSTEM
(modified after loading)
Additional Info : Peak(s) manually integrated

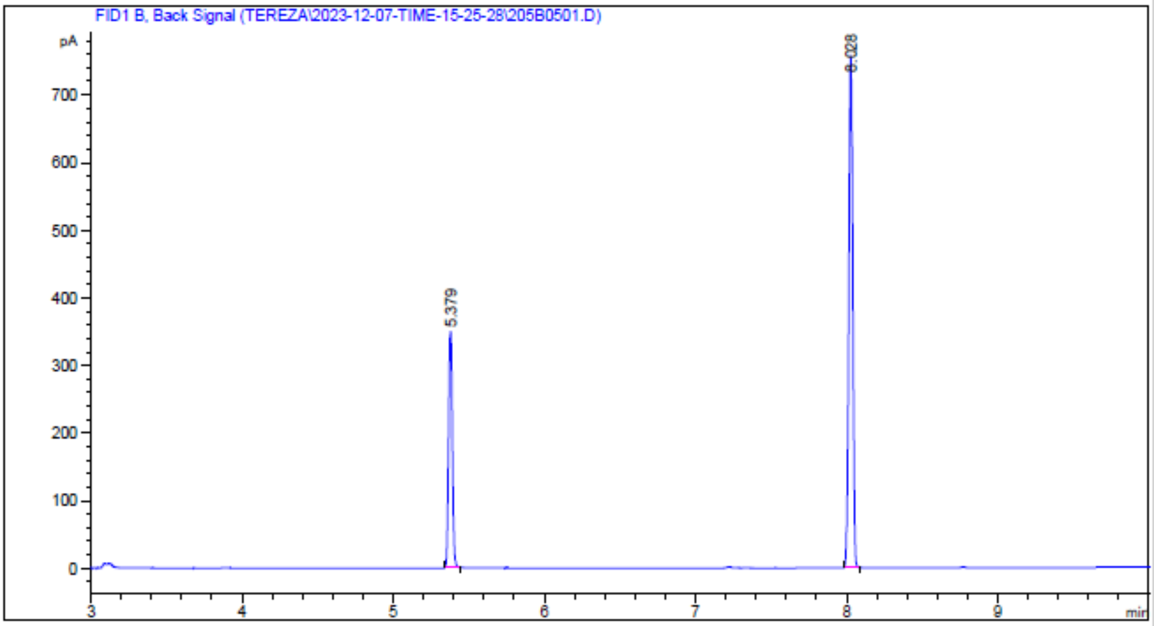

===

Area Percent Report

===

Sorted By : Signal
Multiplier : 1.0000
Dilution : 1.0000
Do not use Multiplier & Dilution Factor with ISTDs

Signal 1: FID1 B, Back Signal

Peak #	RetTime [min]	Type	Width [min]	Area [pA*s]	Height [pA]	Area %
1	5.379	BB	0.0272	603.89996	349.30713	30.57726
2	8.028	BB	0.0283	1371.09729	751.27643	69.42274

Totals : 1974.99725 1100.58356

Figure S147: Gas chromatography spectrum of standard sample containing 5 mM cyclohexanone and 5 mM dodecane

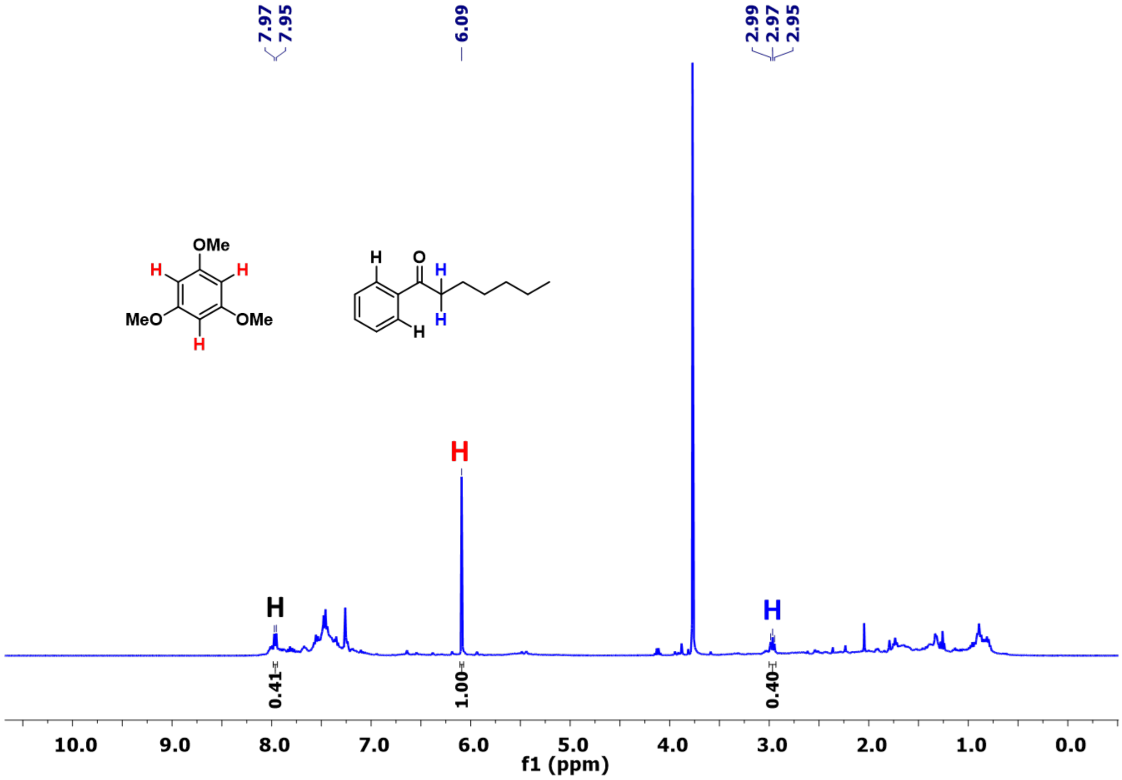

Figure S148: ^{1}H NMR spectrum (400 MHz, $CDCl_3$) of crude product mixture with 1/3 equiv. 1,3,5-trimethoxybenzene after hydrogenation of **(2*E*,4*E*)-1-phenylhepta-2,4-dien-1-one**.

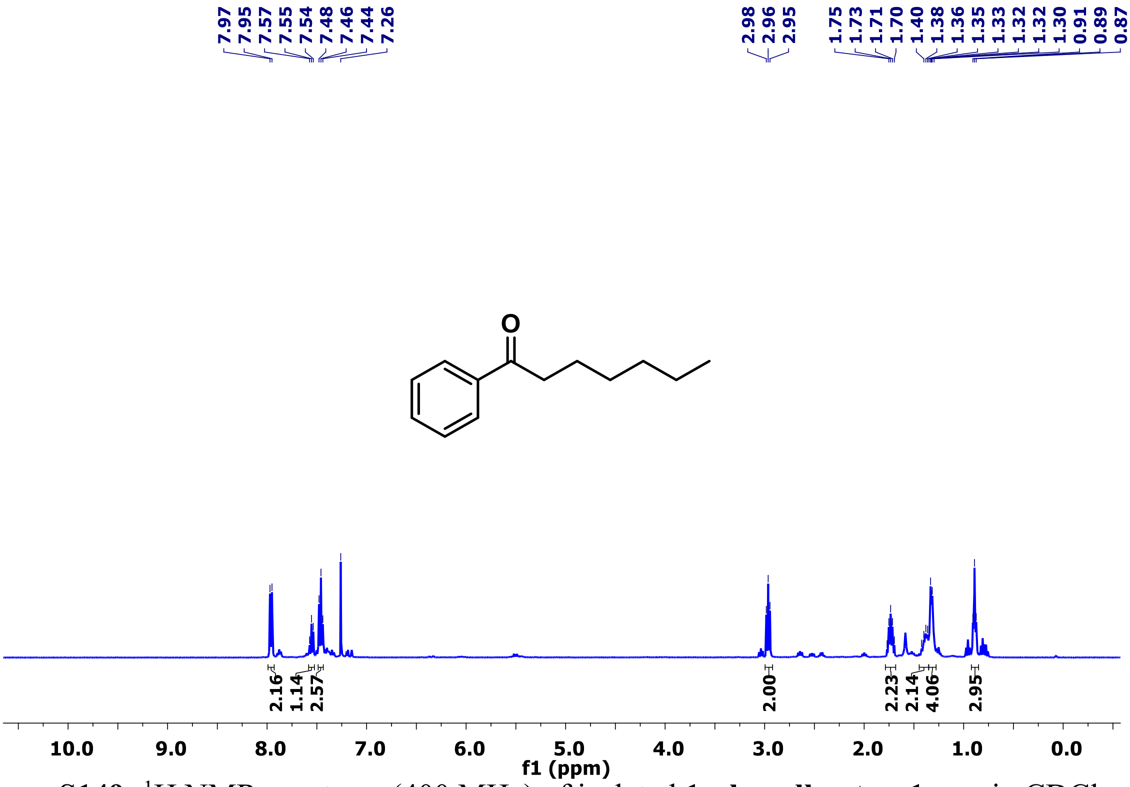

Figure S149: ^{1}H NMR spectrum (400 MHz) of isolated **1-phenylheptan-1-one** in $CDCl_3$

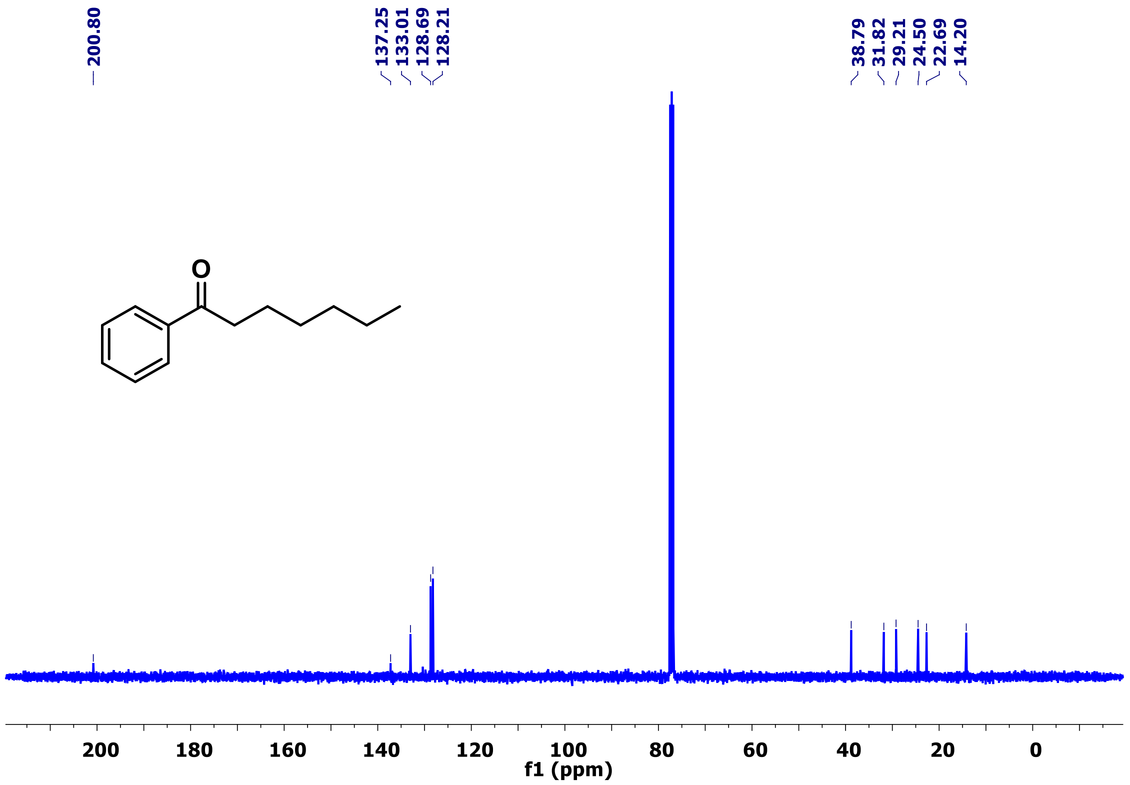

Figure S150: $^{13}C\{^{1}H\}$ NMR spectrum (101 MHz) of **1-phenylheptan-1-one** in $CDCl_3$. The NMR data was in accordance with the literature.[23]

References

[1] R. Thenarukandiyil, R. Kamte, S. Garhwal, P. Effnert, N. Fridman, G. de Ruiter, *Organometallics* **2023**, *42*, 62-71.

[2] aS. Senguttuvan, S. Nagarajan, *Journal of Heterocyclic Chemistry* **2009**, *46*, 1346-1348; bA. Sau, D. Panja, S. Dey, R. Kundu, S. Kundu, *Journal of Catalysis* **2022**, *414*, 225-235.

[3] K. Nicholson, T. Langer, S. P. Thomas, *Organic Letters* **2021**, *23*, 2498-2504.

[4] S. Fekete, T. Patonay, A. M. S. Silva, J. A. S. Cavaleiro, *ARKIVOC* **2012**, *2012*, 210-225.

[5] L. Huang, M. Rudolph, F. Rominger, A. S. K. Hashmi, *Angewandte Chemie International Edition* **2016**, *55*, 4808-4813.

[6] A. B. Shabade, D. M. Sharma, P. Bajpai, R. G. Gonnade, K. Vanka, B. Punji, *Chemical Science* **2022**, *13*, 13764-13773.

[7] N. Garg, H. P. Somasundharam, P. Dahiya, B. Sundararaju, *Chemical Communications* **2022**, *58*, 9930-9933.

[8] M. K. Barman, A. Jana, B. Maji, *Advanced Synthesis & Catalysis* **2018**, *360*, 3233-3238.

[9] H. Qin, J. Yang, K. Yan, Y. Xue, M. Zhang, X. Sun, J. Wen, H. Wang, *Advanced Synthesis & Catalysis* **2021**, *363*, 2104-2109.

[10] Z. Luo, X. Zhang, Z.-Q. Liu, C.-M. Hong, Q.-H. Li, T.-L. Liu, *Organic Letters* **2022**, *24*, 8072-8076.

[11] D. Bhattacharyya, B. K. Sarmah, S. Nandi, H. K. Srivastava, A. Das, *Organic Letters* **2021**, *23*, 869-875.

[12] X. Zhu, C. Ye, Y. Li, H. Bao, *Chem. Eur. J.* **2017**, *23*, 10254-10258.

[13] V. K. Chenniappan, S. Silwal, R. J. Rahaim, *ACS Catalysis* **2018**, *8*, 4539-4544.

[14] G.-Z. Wang, R. Shang, W.-M. Cheng, Y. Fu, *Organic Letters* **2015**, *17*, 4830-4833.

[15] J. Ma, N. Wang, F. Li, *Asian Journal of Organic Chemistry* **2014**, *3*, 940-947.

[16] P. Colbon, J. Ruan, M. Purdie, J. Xiao, *Organic Letters* **2010**, *12*, 3670-3673.

[17] J. Yang, Y. W. Seto, N. Yoshikai, *ACS Catalysis* **2015**, *5*, 3054-3057.

[18] X. Li, X. Shao, X. Zhang, Q. Zhao, H. Lai, B. Cui, Z. Shao, M. Zhao, *Organic & Biomolecular Chemistry* **2022**, *20*, 6542-6546.

[19] G. Zhang, J. Wu, H. Zeng, S. Zhang, Z. Yin, S. Zheng, *Organic Letters* **2017**, *19*, 1080-1083.

[20] D. Shen, D. L. Poole, C. C. Shotton, A. F. Kornahrens, M. P. Healy, T. J. Donohoe, *Angewandte Chemie International Edition* **2015**, *54*, 1642-1645.

[21] M. Benohoud, S. Tuokko, P. M. Pihko, *Chemistry – A European Journal* **2011**, *17*, 8404-8413.

[22] M. Gatto, A. Del Zotto, J. Segato, D. Zuccaccia, *Organometallics* **2018**, *37*, 4685-4691.

[23] W. Kong, C. Yu, H. An, Q. Song, *Organic Letters* **2018**, *20*, 349-352.
